# Catalytic Stereoconvergent Synthesis of Homochiral β-CF_3_, β-SCF_3_, and β-OCF_3_ Benzylic Alcohols

**DOI:** 10.1021/acsorginorgau.2c00019

**Published:** 2022-06-08

**Authors:** Andrej Emanuel Cotman, Pavel A. Dub, Maša Sterle, Matic Lozinšek, Jaka Dernovšek, Živa Zajec, Anamarija Zega, Tihomir Tomašič, Dominique Cahard

**Affiliations:** † Faculty of Pharmacy, University of Ljubljana, Aškerčeva cesta 7, SI-1000 Ljubljana, Slovenia; ‡ Chemistry Division, Los Alamos National Laboratory, Los Alamos, New Mexico 87545, United States; § Jožef Stefan Institute, Jamova cesta 39, SI-1000 Ljubljana, Slovenia; ∥ CNRS UMR 6014 COBRA, Normandie Université, 76821 Mont Saint Aignan, France

**Keywords:** adaptive crystals, asymmetric catalysis, density functional calculations, drug design, fluorine, hydrogenation, kinetic resolution, ruthenium

## Abstract

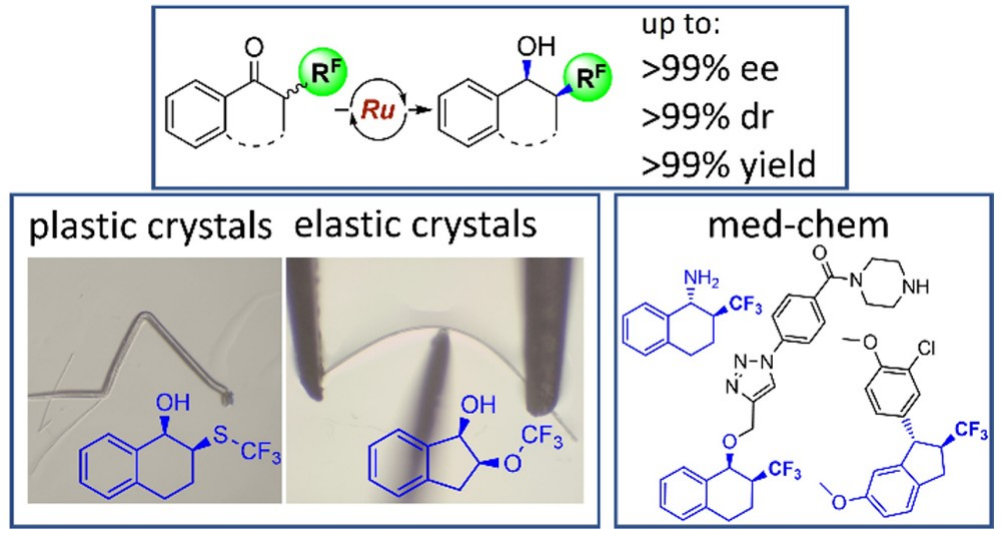

We describe an efficient catalytic strategy for enantio- and diastereoselective synthesis of homochiral β-CF_3_, β-SCF_3_, and β-OCF_3_ benzylic alcohols. The approach is based on dynamic kinetic resolution (DKR) with Noyori–Ikariya asymmetric transfer hydrogenation leading to simultaneous construction of two contiguous stereogenic centers with up to 99.9% ee, up to 99.9:0.1 dr, and up to 99% isolated yield. The origin of the stereoselectivity and racemization mechanism of DKR is rationalized by density functional theory calculations. Applicability of the previously inaccessible chiral fluorinated alcohols obtained by this method in two directions is further demonstrated: As building blocks for pharmaceuticals, illustrated by the synthesis of heat shock protein 90 inhibitor with in vitro anticancer activity, and in particular, needle-shaped crystals of representative stereopure products that exhibit either elastic or plastic flexibility, which opens the door to functional materials based on mechanically responsive chiral molecular crystals.

## Introduction

Fluorine atoms profoundly influence the properties of bioactive molecules on multiple levels, which results in half of blockbuster drugs and one-third of newly approved drugs being fluoro-pharmaceuticals.^1−6^ Other fast-growing market segments are those of fluorinated materials for use in the electronics industry and in energy storage^7,8^ and of fluorine-containing agrochemicals.^9^ Organofluorine chemistry is essentially man-made, as only a dozen fluorinated natural products have been identified on Earth.^10^ The consideration of new fluorinated chemotypes for advanced applications therefore inevitably follows the availability of the synthetic methods to access the relevant moieties. Outstanding progress was achieved in the preparation of a plethora of synthetic fluorine compounds.^11^ A less developed area that is highly challenging but very rewarding is the asymmetric synthesis of stereogenic fluorinated molecules.^12−15^ In this context, we embarked on the asymmetric construction of chiral carbon atoms featuring a fluorinated motif with emphasis on the trifluoromethyl group C*–CF_3_ and its heteroatomic homologues C*–SCF_3_ and C*–OCF_3_.

**Figure 1:**
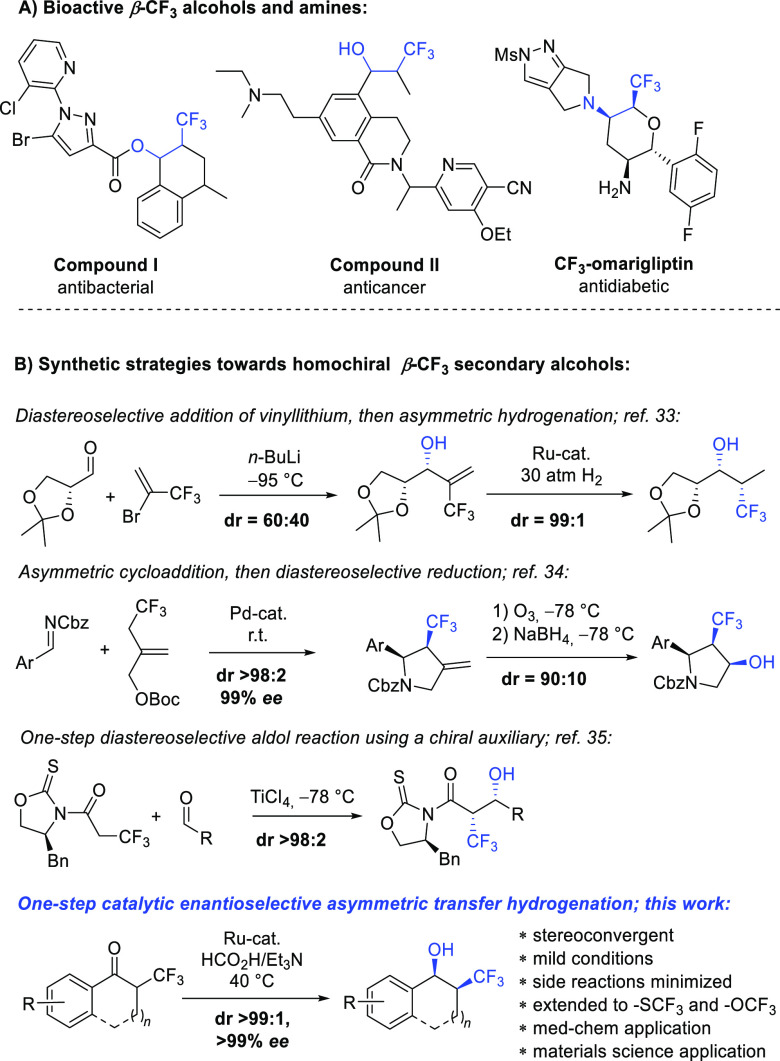
(A) Bioactive compounds with β-CF_3_ alcohol or amine motifs. (B) Synthetic strategies toward homochiral β-CF_3_ secondary alcohols.

In particular, β-CF_3_-substituted alcohols and amines are emerging structural motifs in medicinal chemistry (Figure 1A). For example, compound **I**, prepared as a mixture of stereoisomers, exhibits antibacterial activity,^16^ and racemic compound **II** is an inhibitor of WD repeat-containing protein 5, which is overexpressed in some types of cancer.^17^ Stereochemically defined trifluoromethylated omarigliptin exhibits better pharmacokinetic and pharmacodynamic profiles compared to the parent drug molecule and is clinically evaluated as a super-long-acting antidiabetic.^18^

Surprisingly, however, there are no preceding literature reports on the asymmetric synthesis of the model 2-CF_3_-1-indanol **2a**, its amino analogue, or their higher homologues. Nonasymmetric approaches toward such cyclic benzo-fused β-trifluoromethyl alcohols or amines have received significant attention in the past decade and are based on photoredox, electrochemical, or transition-metal-catalyzed oxy-trifluoromethylation^19−26^ or amino-trifluoromethylation^27−32^ of the corresponding olefins. There are only a handful of literature reports on the synthesis of the homochiral β-trifluoromethyl secondary alcohol motif (Figure 1B). The synthetic strategies are based on a two-step arrangement of the contiguous stereocenters employing diastereoselective addition of nucleophilic vinyllithium followed by substrate-controlled olefin hydrogenation,^33^ or asymmetric cycloaddition followed by NaBH_4_ reduction of the ketone intermediate.^34^ A single-step approach via diastereoselective aldol or Reformatsky reactions using a chiral auxiliary has been described,^35,36^ but to the best of our knowledge no single-step catalytic enantioselective access to this class of molecules has ever been reported.

Dynamic kinetic resolution based on Noyori–Ikariya transfer hydrogenation (DKR-ATH) seemed like a fitting synthetic strategy for addressing the challenging simultaneous control of both chiral centers of the target compound class.^37−41^ DKR-ATH is a robust method for stereoconvergent access to enantiomerically pure secondary alcohols with multiple contiguous chiral centers starting from the readily available racemic α-substituted ketones,^42−50^ including fluorinated examples.^51−58^ This approach to β-CF_3_ alcohols would involve in situ epimerization of α-CF_3_ ketones via an enol or enolate-anion intermediate. Specifically, α-CF_3_ enolates have been associated with decomposition due to fluoride elimination to furnish the corresponding unstable difluoroenone.^59−61^ This was foreseen as the major obstacle toward an efficient DKR-ATH-based catalytic asymmetric synthesis of β-CF_3_ alcohols.

## Results and Discussion

A model racemic ketone 2-CF_3_-1-indanone **1a** was prepared in one step by triflic acid mediated annulation of benzene with 2-CF_3_-acrylic acid.^62^ It was subjected to DKR-ATH using a commonly used formic acid/triethylamine 3:2 mixture as a source of hydrogen and chlorobenzene as a cosolvent, and five representative Noyori–Ikariya type Ru(II) catalysts were tested (Table 1, runs 1–5). **C1** is the archetypical Noyori catalyst,^63,64^ and the rest are the so-called tethered catalysts, which proved to be superior for the reduction of structurally complex ketones.^65^ Chronologically, **C2** was developed by Wills et al.,^66^ followed by oxy-tethered catalyst **C3** by Ikariya et al.,^67^ sulfamoyl-DPEN-cored **C4**,^68^ and benzosultam-cored **C5** by Mohar and co-workers.^69−71^ The reactions using 1 mol % of catalysts **C1**–**C5** all reached >95% conversion within 3 h (Table 1, entries 1–5). Delightfully, all the catalysts yielded the product **2a** with excellent stereoselectivity^72^ (*cis*/*trans* > 99:1 and > 99% ee) as determined by ^19^F NMR and chiral HPLC, respectively. The absolute configuration of **2a** as (*S,S*) was determined by single-crystal X-ray diffraction (SCXRD) analysis of a product from the run with (*S*,*S*)-**C2** (Table 1, entry 2).

**Table 1:**
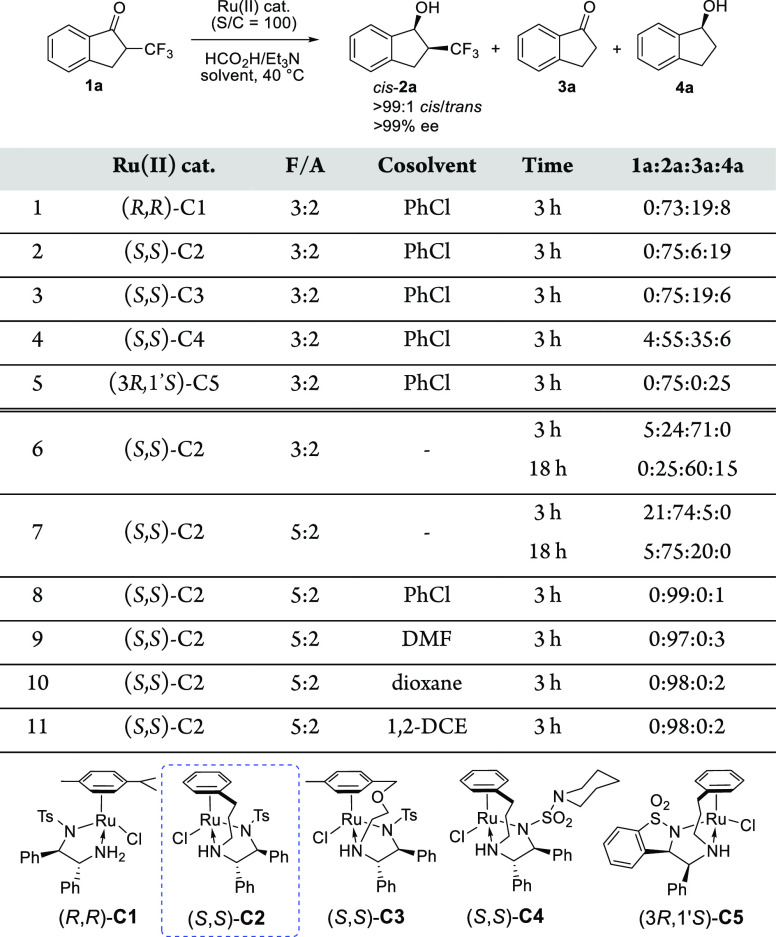
Catalyst and Solvent Screening for Ru(II)-Catalyzed DKR-ATH of **1a**^a^

a DKR-ATH of **1a** (50 mg, 0.25 mmol) was carried out using a Ru(II) catalyst (1 mol %), HCO_2_H/Et_3_N (F/A) (0.25 mL) and cosolvent (0.5 mL) at 40 °C. The product ratio was determined by NMR analysis of reaction mixture aliquots, and the ratio of **2a** stereoisomers (*cis*/*trans* > 99:1; > 99% ee in all cases) was determined after isolation by ^19^F NMR and HPLC analysis using the chiral stationary phase. PhCl = chlorobenzene; DMF = *N*,*N*-dimethylformamide; dioxane = 1,4-dioxane; 1,2-DCE = 1,2-dichloroethane.

Disappointedly, significant amounts of side products, indanone **3a** and/or indanol **4a** (up to 41% total), were also detected in the reaction mixtures, indicating that detrifluoromethylation indeed took place during DKR-ATH. The catalysts performed differently regarding side product formation, and **C2** was chosen for further studies because of its wide availability and favorable reaction kinetics (Table S1). Control experiments indicated that the trifluoromethyl moiety is eliminated from the ketone **1a** rather than the product *cis*-**2a** via a non-ruthenium-catalyzed process involving the formation of the Et_3_N/HF adduct (see the Supporting Information (SI)). To mitigate fluoride elimination, the use of HCO_2_H/Et_3_N in a 5:2 molar ratio with the most efficient (*S,S*)-**C2** was attempted. Performing the DKR-ATH in neat HCO_2_H/Et_3_N 3:2 or 5:2 (Table 1, entries 6 and 7) revealed that, by increasing the relative amount of formic acid, the extent of detrifluoromethylation dramatically decreases while excellent stereoselectivities are still obtained. Further solvent screening revealed that the use of any cosolvent together with HCO_2_H/Et_3_N 5:2 was beneficial for the reaction yield as less than 3% of the side products were observed in chlorobenzene, DMF, 1,4-dioxane, or 1,2-dichloroethane (Table 1, entries 8–11). The first one was deemed optimal with only 1 mol % 1-indanol accompanying the target product **2a**.

**Figure 2:**
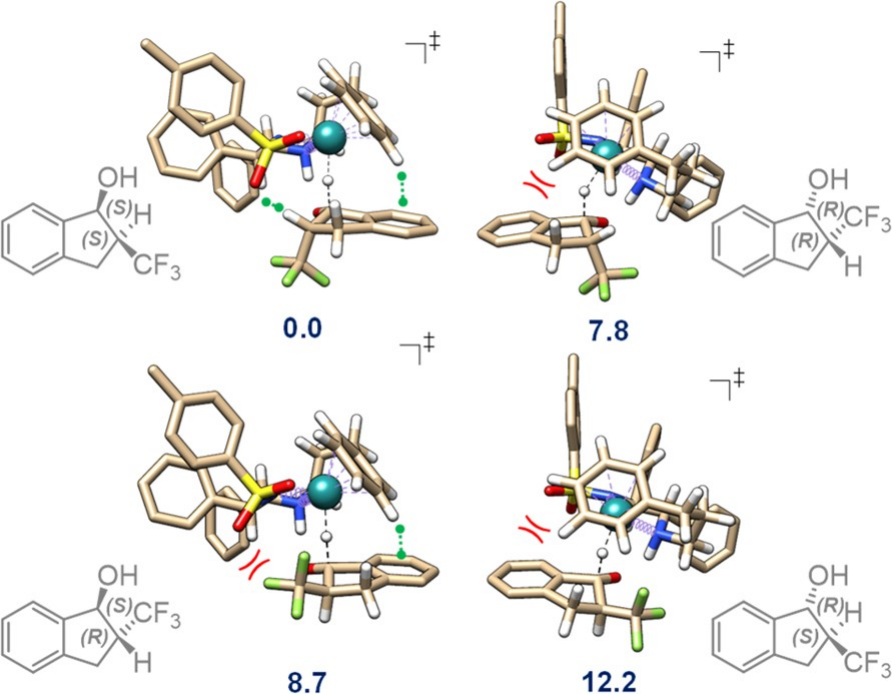
Optimized transition state geometries en route to the four stereomeric products **2a** taking place with *R*_Ru_,λ structural arrangement of the (*S,S*)-**C2** catalyst active form (see text). The relative free energies are given in kcal·mol^–1^. Some attractive and repulsive interactions are highlighted by green and red symbols, respectively. Noncritical H atoms are omitted for clarity.

Computational modeling was further performed to corroborate the high level of stereoselectivities and realize the possible mechanism of **1a** racemization being the core process of DKR. The reaction between **1a** and the active form of precatalyst (*S*,*S*)-**C2** was studied using the M06-2X-D3/SMD(chlorobenzene)/def2-qzvp//def2-svp method. Four diastereomeric transition states are possible (Figure 2).

For the *R*_Ru_,λ-catalyst structural arrangement,^73,74^ observed in the solid-state of (*S*,*S*)-**C2**,^66^ computations predict the ratio of the reaction rates leading to each stereoisomer as ∼10^9^ (*S*,*S*):1800 (*R*,*R*):400 (*S*,*R*):1 (*R*,*S*).^75^ This transforms into a *cis*/*trans* ratio of 2.5 × 10^6^ and enantioselectivity of 99.9996% for the *cis* product.^76^ The discrepancy between experimentally and theoretically predicted % ee is likely due to the additional mechanisms of the generation of chirality.^77^ However, the calculation reproduces and points to a high level of stereodiscrimination. Two spatial regions of the catalyst simultaneously control the final stereoselectivity: the region of the tethered η^6^-arene ligand and the region of the SO_2_ moiety.^71,77^ Dynamic equilibrium and interplay of attraction and repulsion in each region through various noncovalent interactions lead to stabilization/destabilization of the corresponding stereoselectivity-determining transition states. The presence of the α-CF_3_ functionality is crucial for exceptionally high stereoselectivity. As a comparison, DKR-ATH of 2-methyl-1-indanone using **C3** yielded the corresponding alcohol with a lower *cis*-selectivity (*cis*/*trans* = 98:2, 98% ee),^45^ whereas DKR-ATH of 2-acetamido-1-indanone (hydrogen bond donor α-substituent) using **C5** was *trans*-selective (*cis*/*trans* = 9:91).^71^

A 3:2 mixture of HCO_2_H/Et_3_N is a typical choice for DKR with Noyori–Ikariya catalysts,^78^ whereas a 5:2 mixture is usually used for ATH of simple ketones and imines.^79−82^ Although generally not explained, an Et_3_N or Et_3_N/HCO_2_H mixture might serve as a catalyst for the DKR-enabling rapid in situ racemization of the α-substituted ketones, consistent with the 3:2 choice.^83^ Indeed, computations point that direct noncatalyzed epimerization of **1a** is energetically prohibitive (Figure S1, top). On the contrary, **1a** racemization catalyzed by Et_3_N (“enolate-anion” pathway) and the concerted Et_3_N/HCO_2_H process (“enol” pathway) are energetically plausible with the preference to the former by 4.3 kcal·mol^–1^ (Figure S1, middle and bottom). Increasing the relative concentration of formic acid, associated with decreased fluoride elimination, pushes the major racemization pathway toward the concerted Et_3_N/HCO_2_H process.

With optimal conditions in hand, we turned our attention to DKR-ATH of various α-trifluoromethyl substituted benzo-fused cyclic ketones **1b**–**1m** (Table 2). These were prepared as described for **1a**,^62^ via radical desulfur-fragmentation followed by reconstruction of enol triflates^84^ and radical trifluoromethylation of the corresponding olefins^85^ and enol acetates,^86^ respectively (see the SI). The ketones **1a**–**1m** were all converted to the corresponding stereopure alcohols **2a**–**2m** using the optimized reaction conditions (1 mol % of **C2** in HCO_2_H/Et_3_N 5:2 and chlorobenzene at 40 °C) with reaction times to reach full conversion being between 1 and 6 h. Their (*S*,*S*)-absolute configuration was assigned based on SCXRD analysis of indan-cored **2a**, **2d**, and **2f** and tetralin-cored **2k**. The values of *cis*/*trans* ratio and ee in Table 2 are given as “>99”, but the other three possible stereoisomers were in fact present below the limit of detection for most cases,^87^ and the ee of the benzosuberol **2m** was determined to be 99.2%. The tetramethyl substituted indanone **1c** required a higher catalyst loading (5 mol %) to reach full conversion. The reaction yield was affected by competing detrifluoromethylaton which was generally more expressed during DKR-ATH of indan-cored ketones compared to their six-membered analogs. Nevertheless, the decomposition products **3a**–**3h** and **4a**–**4h** were readily removable by flash chromatography. 7-Acetamido analog **2h** was formed in only 37% NMR yield with fast decomposition coupled to fast reduction in HCO_2_H/Et_3_N 3:2, which still outperformed the 5:2 ratio with 25% NMR yield and full conversion achieved only after 18 h. The tetralin derivatives **1i**–**1k** were devoid of detrifluoromethylation, and the corresponding stereopure products **2i**−**2k** were isolated directly after the extraction.

**Table 2:**
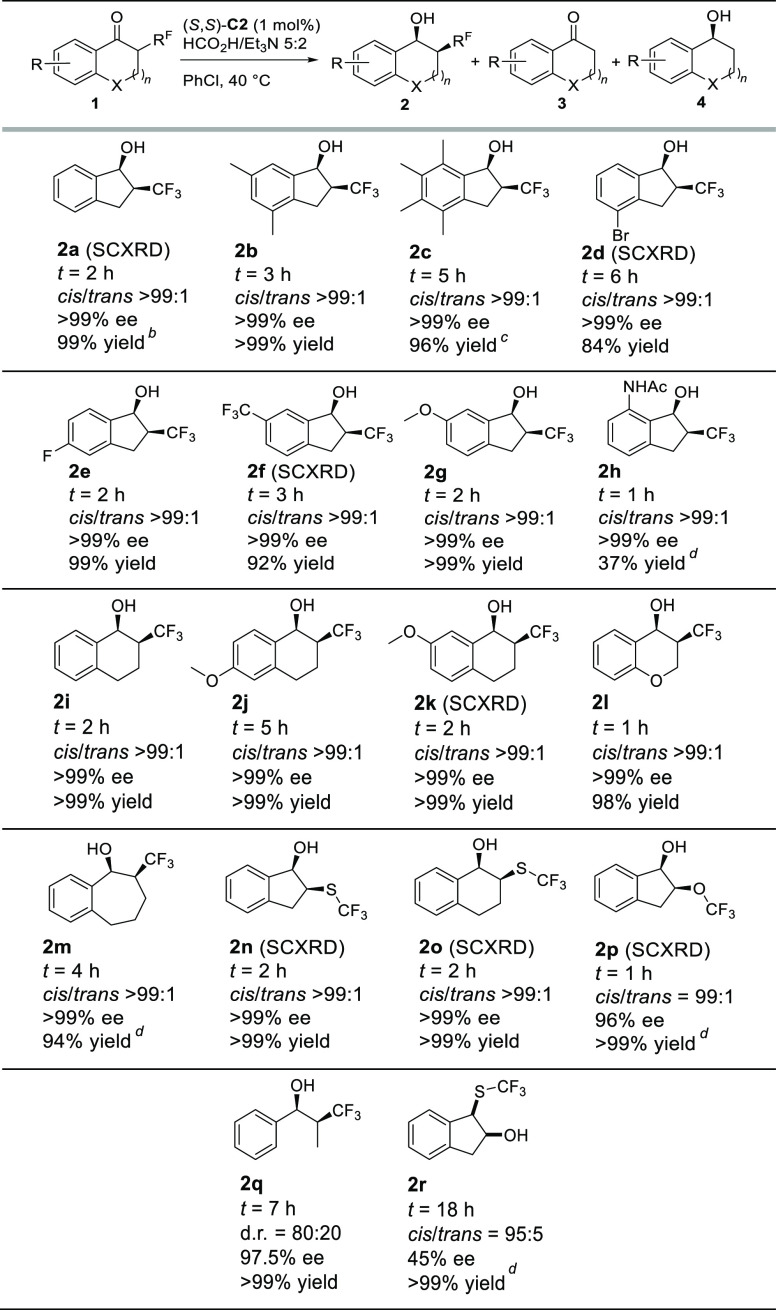
Scope of the DKR-ATH^a^

a Unless otherwise specified, the reactions were carried out using (*S*,*S*)-**C2** (1 mol %) in HCO_2_H/Et_3_N 5:2 and chlorobenzene at 40 °C.

b NMR yield based on integration of **2** relative to **1**,**3** and **4**. Isolated yields after extraction and optional column chromatography were 1–15% lower.

c 5 mol % of (S,S)-**C2** used.

d HCO_2_H/Et_3_N 3:2 used.

The method was then extended to the synthesis of stereopure 2-SCF_3_ and 2-OCF_3_ carbinols **2n**–**2p**, where no side reactions were observed in either HCO_2_H/Et_3_N ratio tested. Stereoselectivities for both trifluoromethylthioethers **2n** and **2o** were determined to be *cis*/*tran*s = 99.9:0.1 and 99.8% ee by ^19^F NMR and chiral GC, respectively, which gives an estimate of the detection limit. The starting 2-SCF_3_ ketones **1n** and **1o** were prepared by means of Billard’s reagent under acidic conditions from the corresponding bare ketones.^88^ 2-Trifluoromethoxy-1-indanol **2p** was obtained with somewhat lower stereopurity (*cis*/*trans* = 99:1, 96% ee) with the same sense of enantioselectivity (SCXRD analysis); its ketone precursor **1p** was accessed via silver mediated oxidative trifluoromethylation of 2-hydroxy-1-indanone.^89^ Pushing it further, the linear analogue **1q** was successfully reduced within 7 h using the same standard conditions, delivering the product **2q** as a 3:1 mixture of *anti* and *syn* diastereomers with 97.4% and 90.4% ee, respectively. The reduction of 1-SCF_3_-2-indanone **1r** to the corresponding alcohol **2r** was unfortunately not highly enantioselective (45% ee), although a 95:5 *cis*/*trans* ratio was achieved.

**Figure 3:**
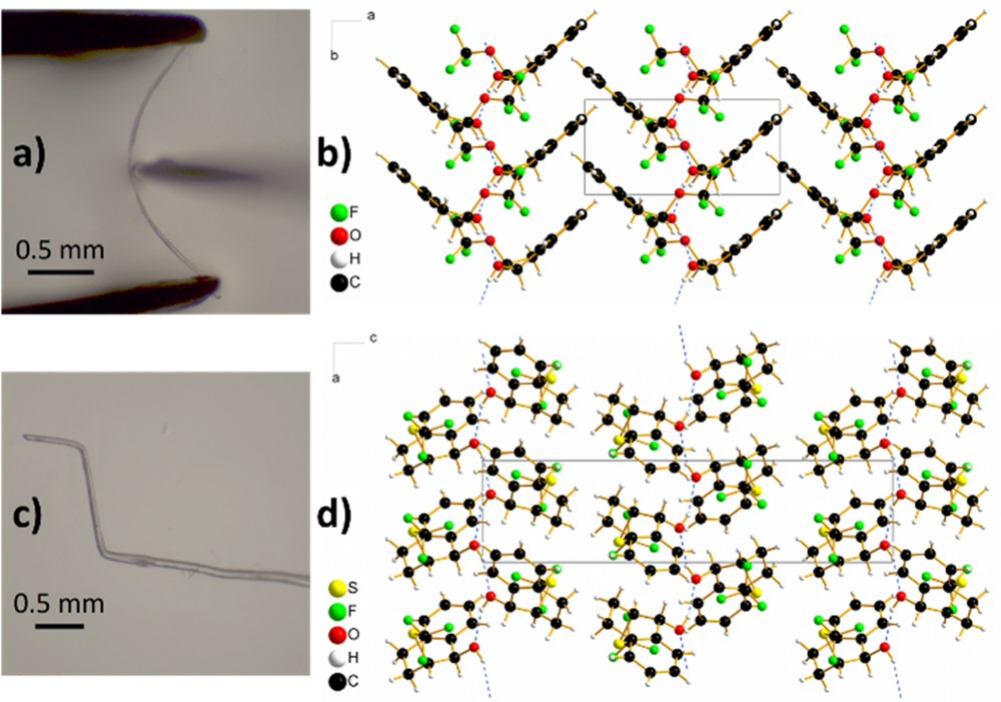
(a) Three-point bending experiment with elastically flexible needle-shaped crystal of **2p**. (b) Crystal packing of **2p**, view along *c*-axis. (c) Bent plastically flexible crystal of **2o**. (d) Crystal packing of **2o**, view along *b*-axis.

We were pleased to find out that some of the novel enantiopure compounds prepared by our method crystallize as needle-shaped crystals which are elastically (**2a**, **2d**, **2p**, and **4d**) or plastically (**2o**) flexible (Figure 3 and SI). Mechanically responsive molecular crystals are being recognized as an unexplored platform for applications ranging from adaptive systems and actuators to biocompatible devices and all-organic soft robots.^90−94^ The crystal structures of **2a**, **2d**, **2o**, **2p**, and **4d** exhibit some of the same features that were identified in other crystals with elastic^95−98^ or plastic deformation behavior:^99−101^ in particular, a short crystal axis (∼5 Å), anisotropic packing, corrugated crystal packing, and a prominent intermolecular interaction that is highly directional (i.e., hydrogen-bonded chains parallel to the short *a*-crystallographic axis in structures with *P*2_1_2_1_2_1_ symmetry and parallel to the short *b*-crystallographic axis in compounds crystallizing in *P*2_1_ space group) with much weaker interactions in the perpendicular directions. The slippage of molecular layers lined with trifluoromethyl groups has previously been established to be the mechanism of the observed plastic deformation.^101^ In our case, chiral OH and the semisaturated benzo-fused scaffold clearly also contribute to mechanic responsiveness as detrifluoromethylated bromoindanol **4d** was also to some degree elastically flexible.^102^ Moreover, for plastically flexible **2o**, two polymorphs (RT *P*2_1_, and 100 K *P*2_1_2_1_2_1_) were identified. On the other hand, the single crystals of **2f** (*P*2_1_2_1_2_1_), **2k** (*P*1), and **2n** (*P*2_1_2_1_2_1_) exhibit a typical brittle behavior, suggesting that subtle differences in molecular structure and crystal packing determine the sweet spot of homochiral single-component flexible crystals.

**Scheme 1:**
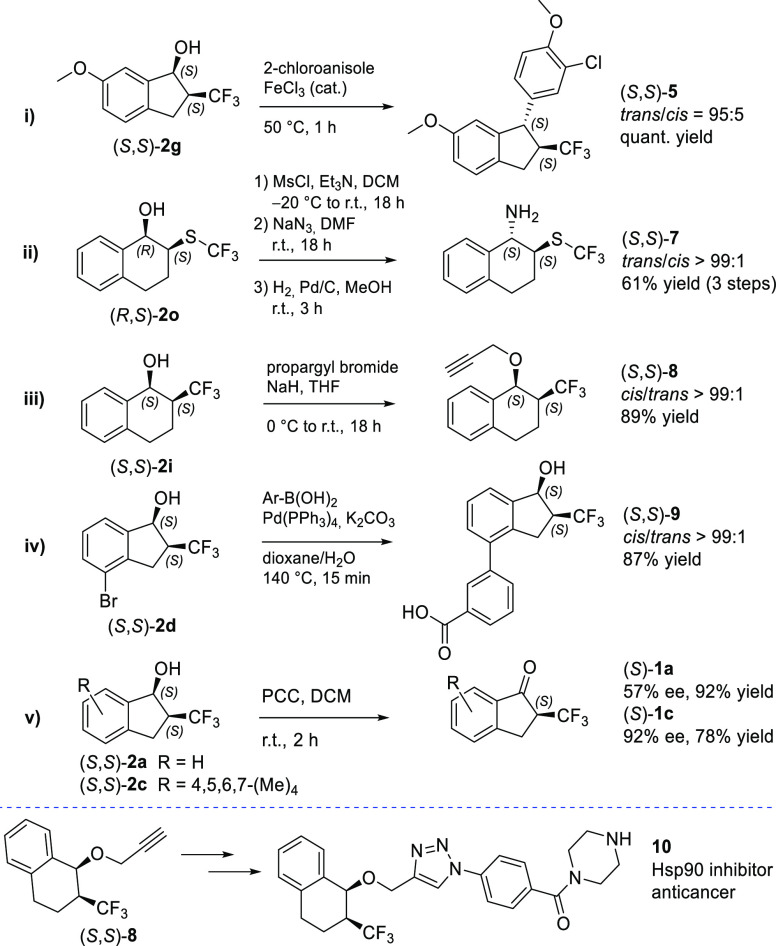
Further Synthetic Transformations of Stereopure DKR-ATH Products **2**

From the medicinal chemistry point of view, the stereopure products **2** represent hitherto synthetically inaccessible building blocks featuring intrinsic nonplanarity, potential for specific interactions with the protein binding sites, and several growth vectors.^103−105^ Selected stereopure products **2** were thus prepared on the 1 mmol scale, and relevant further synthetic transformations were demonstrated (Scheme 1). **2g** was transformed to *trans*-configured **5** via iron-catalyzed diastereoselective Friedel–Crafts benzylation of 2-chloroanisole.^106,107^ This hydroxy-substituted 1-arylindan motif is characteristic of resveratrol dimer natural products.^108,109^**2o** was converted to azide **6** (*trans*/*cis* = 92:8) via nucleophilic substitution (S_N_2) of the corresponding mesylate ester. It was further reduced to the corresponding amine **7** which was isolated as a single stereomer after chromatography. **2i** was *O*-alkylated to obtain stereopure clickable building block **8**. **2d** was converted to biaryl **9** via Suzuki coupling reaction, illustrating that unprotected 2-CF_3_-carbinols are compatible with palladium catalysis. And finally, stereopure **2a** and **2c** were reoxidized using pyridinium chlorochromate to obtain enantio-enriched **1a** and **1c** with 57% and 92% ee, respectively. To showcase the direct applicability of the developed synthetic methods in a medicinal chemistry setting, alkyne **8** was incorporated into **10** which represents a novel structural class of heat shock protein 90 (Hsp90) inhibitors. Compound **10** was designed using a molecular-dynamics-derived pharmacophore model (Figure S2).^110,111^ It was shown to inhibit Hsp90 in the luciferase refolding assay and display antiproliferative activity in the SkBr3 breast cancer cell line (IC_50_ = 51 ± 2 μM).

## Conclusion

In conclusion, we have successfully developed a highly efficient dynamic kinetic resolution strategy for the Noyori–Ikariya asymmetric transfer hydrogenation of racemic α-CF_3_, α-SCF_3,_ and α-OCF_3_ aryl ketones with excellent stereoselectivities (up to 99.9% ee, up to 99.9:0.1 dr) and suppressed detrifluoromethylation. The origin of DKR (in situ epimerization of the ketone substrate and stereoselectivity) were investigated by DFT calculations. Applicability in the field of medicinal chemistry was demonstrated by several further transformations of the stereopure products including incorporation into a promising in vitro anticancer compound. Moreover, an unprecedented class of homochiral small organic molecules, which crystallize as mechanically responsive single-component crystals, was identified. Overall, the presented synthetic methodology opens the door to new chiral fluorinated bioactive lead compounds and to materials science applications based on adaptive chiral molecular crystals.

## Supporting Information Available

Movie 2d: movie showing the elastic flexibility of **2d** (AVI)
Movie 4d: movie showing the elastic flexibility of **4d** (AVI)
Experimental procedures, chiral HPLC and GC chromatograms, NMR spectra of the prepared compounds, cell-based assays, computational and SCXRD details, photos of mechanically responsive behavior (PDF)

## Supplementary Material

gg2c00019_si_001.avi

gg2c00019_si_002.avi

## References

[ref1] Inoue M.; Sumii Y.; Shibata N. Contribution of Organofluorine Compounds to Pharmaceuticals.. ACS Omega 2020, 5, 10633–10640. 10.1021/acsomega.0c00830. 32455181 PMC7240833

[ref2] Wang J.; Sánchez-Roselló M.; Aceña J. L.; del Pozo C.; Sorochinsky A. E.; Fustero S.; Soloshonok V. A.; Liu H. Fluorine in Pharmaceutical Industry: Fluorine-Containing Drugs Introduced to the Market in the Last Decade (2001–2011).. Chem. Rev. 2014, 114, 2432–2506. 10.1021/cr4002879. 24299176

[ref3] Gillis E. P.; Eastman K. J.; Hill M. D.; Donnelly D. J.; Meanwell N. A. Applications of Fluorine in Medicinal Chemistry.. J. Med. Chem. 2015, 58, 8315–8359. 10.1021/acs.jmedchem.5b00258. 26200936

[ref4] Han J.; Remete A. M.; Dobson L. S.; Kiss L.; Izawa K.; Moriwaki H.; Soloshonok V. A.; O’Hagan D. Next Generation Organofluorine Containing Blockbuster Drugs.. J. Fluorine Chem. 2020, 239, 109639 10.1016/j.jfluchem.2020.109639.

[ref5] Mei H.; Remete A. M.; Zou Y.; Moriwaki H.; Fustero S.; Kiss L.; Soloshonok V. A.; Han J. Fluorine-Containing Drugs Approved by the FDA in 2019.. Chin. Chem. Lett. 2020, 31, 2401–2413. 10.1016/j.cclet.2020.03.050.

[ref6] Mei H.; Han J.; Fustero S.; Medio-Simon M.; Sedgwick D. M.; Santi C.; Ruzziconi R.; Soloshonok V. A. Fluorine-Containing Drugs Approved by the FDA in 2018.. Chem.—Eur. J. 2019, 25, 11797–11819. 10.1002/chem.201901840. 31099931

[ref7] Ragni R.; Punzi A.; Babudri F.; Farinola G. M. Organic and Organometallic Fluorinated Materials for Electronics and Optoelectronics: A Survey on Recent Research.. Eur. J. Org. Chem. 2018, 2018, 3500–3519. 10.1002/ejoc.201800657.

[ref8] Fluorinated Materials for Energy Conversion; Nakajima T., Groult H., Eds.; Elsevier: Amsterdam, San Diego, Oxford, 2005.

[ref9] Ogawa Y.; Tokunaga E.; Kobayashi O.; Hirai K.; Shibata N. Current Contributions of Organofluorine Compounds to the Agrochemical Industry.. iScience 2020, 23, 101467 10.1016/j.isci.2020.101467. 32891056 PMC7479632

[ref10] Walker M. C.; Chang M. C. Y. Natural and Engineered Biosynthesis of Fluorinated Natural Products.. Chem. Soc. Rev. 2014, 43, 6527–6536. 10.1039/C4CS00027G. 24776946

[ref11] Emerging Fluorinated Motifs: Synthesis, Properties, and Applications; Cahard D., Ma J.-A., Eds.; Wiley-VCH Verlag GmbH & Co., 2020.

[ref12] Meyer S.; Häfliger J.; Gilmour R. Expanding Organofluorine Chemical Space: The Design of Chiral Fluorinated Isosteres Enabled by I(I)/I(III) Catalysis.. Chem. Sci. 2021, 12, 10686–10695. 10.1039/D1SC02880D. 34476053 PMC8372324

[ref13] Zhu Y.; Han J.; Wang J.; Shibata N.; Sodeoka M.; Soloshonok V. A.; Coelho J. A. S.; Toste F. D. Modern Approaches for Asymmetric Construction of Carbon–Fluorine Quaternary Stereogenic Centers: Synthetic Challenges and Pharmaceutical Needs.. Chem. Rev. 2018, 118, 3887–3964. 10.1021/acs.chemrev.7b00778. 29608052 PMC6497456

[ref14] Cahard D.; Bizet V. The influence of fluorine in asymmetric catalysis.. Chem. Soc. Rev. 2014, 43, 135–147. 10.1039/C3CS60193E. 24162874

[ref15] Nie J.; Guo H.-C.; Cahard D.; Ma J.-A. Asymmetric Construction of Stereogenic Carbon Centers Featuring a Trifluoromethyl Group from Prochiral Trifluoromethylated Substrates.. Chem. Rev. 2011, 111, 455–529. 10.1021/cr100166a. 21117644

[ref16] Xie R.; Wu L. Preparation Method of Trifluoromethyl Tetralone Compound.CN111333613A, June 26, 2020.

[ref17] Fang L.; Gao Z.; Jiang X.; Liu K. K. C.; Mak S. Y. F.; Oyang C.; Wang C.; Wang T.; Wu J.; Yingming W.; Xiao Q. Heterocyclic Wdr5 Inhibitors as Anti-Cancer Compounds.WO2021028806A1, February 18, 2021.

[ref18] Zhang C.; Ye F.; Wang J.; He P.; Lei M.; Huang L.; Huang A.; Tang P.; Lin H.; Liao Y.; Liang Y.; Ni J.; Yan P. Design, Synthesis, and Evaluation of a Series of Novel Super Long-Acting DPP-4 Inhibitors for the Treatment of Type 2 Diabetes.. J. Med. Chem. 2020, 63, 7108–7126. 10.1021/acs.jmedchem.0c00374. 32452679

[ref19] Levitre G.; Dagousset G.; Anselmi E.; Tuccio B.; Magnier E.; Masson G. Four-Component Photoredox-Mediated Azidoalkoxy-Trifluoromethylation of Alkenes.. Org. Lett. 2019, 21, 6005–6010. 10.1021/acs.orglett.9b02152. 31339319

[ref20] Zhang L.; Zhang G.; Wang P.; Li Y.; Lei A. Electrochemical Oxidation with Lewis-Acid Catalysis Leads to Trifluoromethylative Difunctionalization of Alkenes Using CF_3_SO_2_Na.. Org. Lett. 2018, 20, 7396–7399. 10.1021/acs.orglett.8b03081. 30461286

[ref21] Jud W.; Kappe C. O.; Cantillo D. Catalyst-Free Oxytrifluoromethylation of Alkenes through Paired Electrolysis in Organic-Aqueous Media.. Chem.—Eur. J. 2018, 24, 17234–17238. 10.1002/chem.201804708. 30285302

[ref22] Valverde E.; Kawamura S.; Sekine D.; Sodeoka M. Metal-Free Alkene Oxy- and Amino-Perfluoroalkylations via Carbocation Formation by Using Perfluoro Acid Anhydrides: Unique Reactivity between Styrenes and Perfluoro Diacyl Peroxides.. Chem. Sci. 2018, 9, 7115–7121. 10.1039/C8SC02547A. 30310632 PMC6137437

[ref23] Yang Y.; Liu Y.; Jiang Y.; Zhang Y.; Vicic D. A. Manganese-Catalyzed Aerobic Oxytrifluoromethylation of Styrene Derivatives Using CF_3_SO_2_Na as the Trifluoromethyl Source.. J. Org. Chem. 2015, 80, 6639–6648. 10.1021/acs.joc.5b00781. 26057534

[ref24] Smirnov V. O.; Maslov A. S.; Kokorekin V. A.; Korlyukov A. A.; Dilman A. D. Photoredox Generation of the Trifluoromethyl Radical from Borate Complexes via Single Electron Reduction.. Chem. Commun. 2018, 54, 2236–2239. 10.1039/C8CC00245B. 29431841

[ref25] Li Y.; Studer A. Tranzition-Metal-Free Trifluoromethylaminoxylation of Alkenes.. Angew. Chem. Int. Ed. 2012, 51, 8221–8224. 10.1002/anie.201202623. 22833448

[ref26] Yasu Y.; Koike T.; Akita M. Three-component Oxytrifluoromethylation of Alkenes: Highly Efficient and Regioselective Difunctionalization of C=C Bonds Mediated by Photoredox Catalysts.. Angew. Chem. Int. Ed. 2012, 51, 9567–9571. 10.1002/anie.201205071. 22936394

[ref27] Wang P.; Zhu S.; Lu D.; Gong Y. Intermolecular Trifluoromethyl-Hydrazination of Alkenes Enabled by Organic Photoredox Catalysis.. Org. Lett. 2020, 22, 1924–1928. 10.1021/acs.orglett.0c00287. 32056439

[ref28] Zhu C.-L.; Wang C.; Qin Q.-X.; Yruegas S.; Martin C. D.; Xu H. Iron(II)-Catalyzed Azidotrifluoromethylation of Olefins and N-Heterocycles for Expedient Vicinal Trifluoromethyl Amine Synthesis.. ACS Catal. 2018, 8, 5032–5037. 10.1021/acscatal.8b01253. 29938121 PMC6010075

[ref29] Zhang Y.; Han X.; Zhao J.; Qian Z.; Li T.; Tang Y.; Zhang H.-Y. Synthesis of β-Trifluoromethylated Alkyl Azides via a Manganese-Catalyzed Trifluoromethylazidation of Alkenes with CF_3_SO_2_Na and TMSN_3_.. Adv. Synth. Catal. 2018, 360, 2659–2667. 10.1002/adsc.201800488.

[ref30] Wang F.; Qi X.; Liang Z.; Chen P.; Liu G. Copper-Catalyzed Intermolecular Trifluoromethylazidation of Alkenes: Convenient Access to CF_3_-Containing Alkyl Azides.. Angew. Chem., Int. Ed. 2014, 53, 1881–1886. 10.1002/anie.201309991. 24505007

[ref31] Dagousset G.; Carboni A.; Magnier E.; Masson G. Photoredox-Induced Three-Component Azido- and Aminotrifluoromethylation of Alkenes.. Org. Lett. 2014, 16, 4340–4343. 10.1021/ol5021477. 25102254

[ref32] Yasu Y.; Koike T.; Akita M. Intermolecular Aminotrifluoromethylation of Alkenes by Visible-Light-Driven Photoredox Catalysis.. Org. Lett. 2013, 15, 2136–2139. 10.1021/ol4006272. 23600821

[ref33] Chen Q.; Qing F.-L. Stereoselective Construction of the 1,1,1-Trifluoroisopropyl Moiety by Asymmetric Hydrogenation of 2-(Trifluoromethyl)Allylic Alcohols and Its Application to the Synthesis of a Trifluoromethylated Amino Diol.. Tetrahedron 2007, 63, 11965–11972. 10.1016/j.tet.2007.09.013.

[ref34] Trost B. M.; Wang Y.; Hung C.-I. J. Use of α-Trifluoromethyl Carbanions for Palladium-Catalysed Asymmetric Cycloadditions.. Nat. Chem. 2020, 12, 294–301. 10.1038/s41557-019-0412-9. 32015479

[ref35] Franck X.; Seon-Meniel B.; Figadère B. Highly Diastereoselective Aldol Reaction with α-CF_3_-Substituted Enolates.. Angew. Chem., Int. Ed. 2006, 45, 5174–5176. 10.1002/anie.200600927. 16823794

[ref36] Shimada T.; Yoshioka M.; Konno T.; Ishihara T. Highly Stereoselective TiCl_4_-Catalyzed Evans–Aldol and Et_3_Al-Mediated Reformatsky Reactions. Efficient Accesses to Optically Active syn- or anti-α-Trifluoromethyl-β-Hydroxy Carboxylic Acid Derivatives.. Org. Lett. 2006, 8, 1129–1131. 10.1021/ol0531435. 16524285

[ref37] Cotman A. E. Escaping from Flatland: Stereoconvergent Synthesis of Three-Dimensional Scaffolds via Ruthenium(II)-Catalyzed Noyori–Ikariya Transfer Hydrogenation.. Chem.—Eur. J. 2021, 27, 39–53. 10.1002/chem.202002779. 32691439

[ref38] Molina Betancourt R.; Echeverria P.-G.; Ayad T.; Phansavath P.; Ratovelomanana-Vidal V. Recent Progress and Applications of Transition-Metal-Catalyzed Asymmetric Hydrogenation and Transfer Hydrogenation of Ketones and Imines through Dynamic Kinetic Resolution.. Synthesis 2021, 53, 30–50. 10.1055/s-0040-1705918.

[ref39] Echeverria P.-G.; Ayad T.; Phansavath P.; Ratovelomanana-Vidal V. Recent Developments in Asymmetric Hydrogenation and Transfer Hydrogenation of Ketones and Imines through Dynamic Kinetic Resolution.. Synthesis 2016, 48, 2523–2539. 10.1055/s-0035-1561648.

[ref40] Matsunami A.; Kayaki Y. Upgrading and Expanding the Scope of Homogeneous Transfer Hydrogenation.. Tetrahedron Lett. 2018, 59, 504–513. 10.1016/j.tetlet.2017.12.078.

[ref41] Echeverria P.-G.; Ayad T.; Phansavath P.; Ratovelomanana-Vidal V. Asymmetric (Transfer) Hydrogenation of Substituted Ketones Through Dynamic Kinetic Resolution. In Asymmetric Hydrogenation and Transfer Hydrogenation; John Wiley & Sons, Ltd, 2021; pp 129–174.

[ref42] Vyas V. K.; Clarkson G. J.; Wills M. Sulfone Group as a Versatile and Removable Directing Group for Asymmetric Transfer Hydrogenation of Ketones.. Angew. Chem. Int. Ed. 2020, 59, 14265–14269. 10.1002/anie.202004658. PMC7496949 32463162

[ref43] Wang F.; Yang T.; Wu T.; Zheng L.-S.; Yin C.; Shi Y.; Ye X.-Y.; Chen G.-Q.; Zhang X. Asymmetric Transfer Hydrogenation of α-Substituted-β-Keto Carbonitriles via Dynamic Kinetic Resolution.. J. Am. Chem. Soc. 2021, 143, 2477–2483. 10.1021/jacs.0c13273. 33529522

[ref44] Touge T.; Nara H.; Kida M.; Matsumura K.; Kayaki Y. Convincing Catalytic Performance of Oxo-Tethered Ruthenium Complexes for Asymmetric Transfer Hydrogenation of Cyclic α-Halogenated Ketones through Dynamic Kinetic Resolution.. Org. Lett. 2021, 23, 3070–3075. 10.1021/acs.orglett.1c00739. 33780258

[ref45] Touge T.; Sakaguchi K.; Tamaki N.; Nara H.; Yokozawa T.; Matsumura K.; Kayaki Y. Multiple Absolute Stereocontrol in Cascade Lactone Formation via Dynamic Kinetic Resolution Driven by the Asymmetric Transfer Hydrogenation of Keto Acids with Oxo-Tethered Ruthenium Catalysts.. J. Am. Chem. Soc. 2019, 141, 16354–16361. 10.1021/jacs.9b07297. 31502833

[ref46] Gediya S. K.; Clarkson G. J.; Wills M. Asymmetric Transfer Hydrogenation: Dynamic Kinetic Resolution of α-Amino Ketones.. J. Org. Chem. 2020, 85, 11309–11330. 10.1021/acs.joc.0c01438. 32786626

[ref47] Carmona J. A.; Rodríguez-Franco C.; López-Serrano J.; Ros A.; Iglesias-Sigüenza J.; Fernández R.; Lassaletta J. M.; Hornillos V. Atroposelective Transfer Hydrogenation of Biaryl Aminals via Dynamic Kinetic Resolution. Synthesis of Axially Chiral Diamines.. ACS Catal. 2021, 11, 4117–4124. 10.1021/acscatal.1c00571.

[ref48] Zhang Y.-M.; Zhang Q.-Y.; Wang D.-C.; Xie M.-S.; Qu G.-R.; Guo H.-M. Asymmetric Transfer Hydrogenation of Rac-α-(Purin-9-yl)Cyclopentones via Dynamic Kinetic Resolution for the Construction of Carbocyclic Nucleosides.. Org. Lett. 2019, 21, 2998–3002. 10.1021/acs.orglett.9b00451. 30939024

[ref49] Luo Z.; Sun G.; Wu S.; Chen Y.; Lin Y.; Zhang L.; Wang Z. η^6^-Arene CH–O Interaction Directed Dynamic Kinetic Resolution – Asymmetric Transfer Hydrogenation (DKR-ATH) of α-Keto/Enol-Lactams.. Adv. Synth. Catal. 2021, 363, 3030–3034. 10.1002/adsc.202100288.

[ref50] More G. V.; Malekar P. V.; Kalshetti R. G.; Shinde M. H.; Ramana C. V. Ru-Catalyzed Asymmetric Transfer Hydrogenation of α-Acyl Butyrolactone via Dynamic Kinetic Resolution: Asymmetric Synthesis of bis-THF Alcohol Intermediate of Darunavir.. Tetrahedron Lett. 2021, 66, 152831 10.1016/j.tetlet.2021.152831.

[ref51] Šterk D.; Stephan M.; Mohar B. Highly Enantioselective Transfer Hydrogenation of Fluoroalkyl Ketones.. Org. Lett. 2006, 8, 5935–5938. 10.1021/ol062358r. 17165898

[ref52] Cotman A. E.; Cahard D.; Mohar B. Stereoarrayed CF_3_-Substituted 1,3-Diols by Dynamic Kinetic Resolution: Ruthenium(II)-Catalyzed Asymmetric Transfer Hydrogenation.. Angew. Chem., Int. Ed. 2016, 55, 5294–5298. 10.1002/anie.201600812. 27001134

[ref53] Ros A.; Magriz A.; Dietrich H.; Fernández R.; Alvarez E.; Lassaletta J. M. Enantioselective Synthesis of Vicinal Halohydrins via Dynamic Kinetic Resolution.. Org. Lett. 2006, 8, 127–130. 10.1021/ol052821k. 16381584

[ref54] Betancourt R. M.; Phansavath P.; Ratovelomanana-Vidal V. Ru(II)-Catalyzed Asymmetric Transfer Hydrogenation of 3-Fluorochromanone Derivatives to Access Enantioenriched *cis*-3-Fluorochroman-4-ols through Dynamic Kinetic Resolution.. J. Org. Chem. 2021, 86, 12054–12063. 10.1021/acs.joc.1c01415. 34375115

[ref55] Wang T.; Phillips E. M.; Dalby S. M.; Sirota E.; Axnanda S.; Shultz C. S.; Patel P.; Waldman J. H.; Alwedi E.; Wang X.; Zawatzky K.; Chow M.; Padivitage N.; Weisel M.; Whittington M.; Duan J.; Lu T. Manufacturing Process Development for Belzutifan, Part 5: A Streamlined Fluorination–Dynamic Kinetic Resolution Process.. Org. Process Res. Dev. 2022, 26 (3), 543–550. 10.1021/acs.oprd.1c00242.

[ref56] Wehn P. M.; Rizzi J. P.; Dixon D. D.; Grina J. A.; Schlachter S. T.; Wang B.; Xu R.; Yang H.; Du X.; Han G.; Wang K.; Cao Z.; Cheng T.; Czerwinski R. M.; Goggin B. S.; Huang H.; Halfmann M. M.; Maddie M. A.; Morton E. L.; Olive S. R.; Tan H.; Xie S.; Wong T.; Josey J. A.; Wallace E. M. Design and Activity of Specific Hypoxia-Inducible Factor-2α (HIF-2α) Inhibitors for the Treatment of Clear Cell Renal Cell Carcinoma: Discovery of Clinical Candidate (*S*)-3-((2,2-Difluoro-1-hydroxy-7-(methylsulfonyl)-2,3-dihydro-1*H*-inden-4-yl)oxy)-5-fluorobenzonitrile (PT2385).. J. Med. Chem. 2018, 61, 9691–9721. 10.1021/acs.jmedchem.8b01196. 30289716

[ref57] Mohar B.; Stephan M.; Urleb U. Stereoselective Synthesis of Fluorine-Containing Analogues of Anti-Bacterial Sanfetrinem and LK-157.. Tetrahedron 2010, 66, 4144–4149. 10.1016/j.tet.2010.03.104.

[ref58] Tan X.; Zeng W.; Wen J.; Zhang X. Iridium-Catalyzed Asymmetric Hydrogenation of α-Fluoro Ketones via a Dynamic Kinetic Resolution Strategy.. Org. Lett. 2020, 22, 7230–7233. 10.1021/acs.orglett.0c02565. 32866391

[ref59] Yokozawa T.; Nakai T.; Ishikawa N. (Trifluoromethyl)ketene silyl acetal as an equivalent to the trifluoropropionic ester enolate: preparation and aldol-type reactions with acetals.. Tetrahedron Lett. 1984, 25, 3987–3990. 10.1016/0040-4039(84)80047-7.

[ref60] Itoh Y.; Yamanaka M.; Mikami K. Direct Generation of Ti-Enolate of α-CF_3_ Ketone: Theoretical Study and High-Yielding and Diastereoselective Aldol Reaction.. J. Am. Chem. Soc. 2004, 126, 13174–13175. 10.1021/ja046518a. 15479042

[ref61] Kizirian J.-C.; Aiguabella N.; Pesquer A.; Fustero S.; Bello P.; Verdaguer X.; Riera A. Regioselectivity in Intermolecular Pauson-Khand Reactions of Dissymmetric Fluorinated Alkynes.. Org. Lett. 2010, 12, 5620–5623. 10.1021/ol102283c. 21082812

[ref62] Prakash G. K. S.; Paknia F.; Vaghoo H.; Rasul G.; Mathew T.; Olah G. A. Preparation of Trifluoromethylated Dihydrocoumarins, Indanones, and Arylpropanoic Acids by Tandem Superacidic Activation of 2-(Trifluoromethyl)Acrylic Acid with Arenes.. J. Org. Chem. 2010, 75, 2219–2226. 10.1021/jo9026275. 20218629

[ref63] Hashiguchi S.; Fujii A.; Takehara J.; Ikariya T.; Noyori R. Asymmetric Transfer Hydrogenation of Aromatic Ketones Catalyzed by Chiral Ruthenium(II) Complexes.. J. Am. Chem. Soc. 1995, 117, 7562–7563. 10.1021/ja00133a037.

[ref64] Haack K.-J.; Hashiguchi S.; Fujii A.; Ikariya T.; Noyori R. The Catalyst Precursor, Catalyst, and Intermediate in the Ru^II^-Promoted Asymmetric Hydrogen Transfer between Alcohols and Ketones.. Angew. Chem. Int. Ed. 1997, 36, 285–288. 10.1002/anie.199702851.

[ref65] G. Nedden H.; Zanotti-Gerosa A.; Wills M. The Development of Phosphine-Free “Tethered” Ruthenium(II) Catalysts for the Asymmetric Reduction of Ketones and Imines.. Chem. Rec. 2016, 16, 2623–2643. 10.1002/tcr.201600084. 27524696

[ref66] Hayes A. M.; Morris D. J.; Clarkson G. J.; Wills M. A Class of Ruthenium(II) Catalyst for Asymmetric Transfer Hydrogenations of Ketones.. J. Am. Chem. Soc. 2005, 127, 7318–7319. 10.1021/ja051486s. 15898773

[ref67] Touge T.; Hakamata T.; Nara H.; Kobayashi T.; Sayo N.; Saito T.; Kayaki Y.; Ikariya T. Oxo-Tethered Ruthenium(II) Complex as a Bifunctional Catalyst for Asymmetric Transfer Hydrogenation and H_2_ Hydrogenation.. J. Am. Chem. Soc. 2011, 133, 14960–14963. 10.1021/ja207283t. 21870824

[ref68] Kišić A.; Stephan M.; Mohar B. *ansa*-Ruthenium(II) Complexes of R_2_NSO_2_DPEN-(CH_2_)_n_(η^6^-Aryl) Conjugate Ligands for Asymmetric Transfer Hydrogenation of Aryl Ketones.. Adv. Synth. Catal. 2015, 357, 2540–2546. 10.1002/adsc.201500288.

[ref69] Rast S.; Modec B.; Stephan M.; Mohar B. γ-Sultam-Cored *N*,*N*-Ligands in the Ruthenium(II)-Catalyzed Asymmetric Transfer Hydrogenation of Aryl Ketones.. Org. Biomol. Chem. 2016, 14, 2112–2120. 10.1039/C5OB02352A. 26781998

[ref70] Jeran M.; Cotman A. E.; Stephan M.; Mohar B. Stereopure Functionalized Benzosultams via Ruthenium(II)-Catalyzed Dynamic Kinetic Resolution–Asymmetric Transfer Hydrogenation.. Org. Lett. 2017, 19, 2042–2045. 10.1021/acs.orglett.7b00670. 28406647

[ref71] Cotman A. E.; Lozinšek M.; Wang B.; Stephan M.; Mohar B. *trans*-Diastereoselective Ru(II)-Catalyzed Asymmetric Transfer Hydrogenation of α-Acetamido Benzocyclic Ketones via Dynamic Kinetic Resolution.. Org. Lett. 2019, 21, 3644–3648. 10.1021/acs.orglett.9b01069. 31058516 PMC6750876

[ref72] (*S*,*S*)-**2a** was obtained with (*S*,*S*)-DPEN based catalysts **C2**, **C3**, and **C4**, and (*R*,*R*)-**2a** was obtained with (*R*,*R*)-**C1** and (3*R*,1’*S*)-**C5**.

[ref73] Dub P. A.; Gordon J. C. The Mechanism of Enantioselective Ketone Reduction with Noyori and Noyori–Ikariya Bifunctional Catalysts.. Dalton Trans. 2016, 45, 6756–6781. 10.1039/C6DT00476H. 26998962

[ref74] Hall A. M. R.; Berry D. B. G.; Crossley J. N.; Codina A.; Clegg I.; Lowe J. P.; Buchard A.; Hintermair U. Does the Configuration at the Metal Matter in Noyori–Ikariya Type Asymmetric Transfer Hydrogenation Catalysts?. ACS Catal. 2021, 11, 13649–13659. 10.1021/acscatal.1c03636. 34777911 PMC8576814

[ref75] Using absolute rate theory, the ratio of the reaction rates for the two pathways is: ln(va/vb) = exp(−Δ*G*_298K_/*RT*)); *RT* = 0.59 kcal·mol^–1^.

[ref76] ee (%) = 100 × [exp(−Δ*G*_298K_/*RT*) – 1]/[exp(−Δ*G*_298K_/*RT*) + 1], where Δ*G*_298K_ is the free-energy difference in kcal·mol^–1^ between the transition states leading to *S*- and *R*-products; *RT* = 0.59 kcal·mol^–1^.

[ref77] Dub P. A.; Tkachenko N. V.; Vyas V. K.; Wills M.; Smith J. S.; Tretiak S. Enantioselectivity in the Noyori–Ikariya Asymmetric Transfer Hydrogenation of Ketones.. Organometallics 2021, 40, 1402–1410. 10.1021/acs.organomet.1c00201.

[ref78] For molar ratios of triethylamine larger than 0.4, the mixture is biphasic. For an example, see: Narita K.; Sekiya M. Vapor-Liquid Equilibrium for Formic Acid-Triethylamine System Examined by the Use of a Modified Still. Formic Acid-Trialkylamine Azeotropes.. Chem. Pharm. Bull. 1977, 25, 135–140. 10.1248/cpb.25.135.

[ref79] Dub P. A.; Matsunami A.; Kuwata S.; Kayaki Y. Cleavage of N–H Bond of Ammonia via Metal–Ligand Cooperation Enables Rational Design of a Conceptually New Noyori–Ikariya Catalyst.. J. Am. Chem. Soc. 2019, 141, 2661–2677. 10.1021/jacs.8b12961. 30715874

[ref80] Barrios-Rivera J.; Xu Y.; Wills M. Asymmetric Transfer Hydrogenation of Unhindered and Non-Electron-Rich 1-Aryl Dihydroisoquinolines with High Enantioselectivity.. Org. Lett. 2020, 22, 6283–6287. 10.1021/acs.orglett.0c02034. 32806188

[ref81] Zheng Y.; Clarkson G. J.; Wills M. Asymmetric Transfer Hydrogenation of O-Hydroxyphenyl Ketones: Utilizing Directing Effects That Optimize the Asymmetric Synthesis of Challenging Alcohols.. Org. Lett. 2020, 22, 3717–3721. 10.1021/acs.orglett.0c01213. 32298124

[ref82] Westermeyer A.; Guillamot G.; Phansavath P.; Ratovelomanana-Vidal V. Synthesis of Enantioenriched β-Hydroxy-γ-Acetal Enamides by Rhodium-Catalyzed Asymmetric Transfer Hydrogenation.. Org. Lett. 2020, 22, 3911–3914. 10.1021/acs.orglett.0c01193. 32330052

[ref83] Epimerization is significantly faster using a higher molar ratio of triethylamine. For an epimerization kinetics study of α-substituted ketone in HCO_2_H/Et_3_N 3:2 or 5:2, see ref (52).

[ref84] Su X.; Huang H.; Yuan Y.; Li Y. Radical Desulfur-Fragmentation and Reconstruction of Enol Triflates: Facile Access to α-Trifluoromethyl Ketones.. Angew. Chem., Int. Ed. 2017, 56, 1338–1341. 10.1002/anie.201608507. 28000360

[ref85] Deb A.; Manna S.; Modak A.; Patra T.; Maity S.; Maiti D. Oxidative Trifluoromethylation of Unactivated Olefins: An Efficient and Practical Synthesis of α-Trifluoromethyl-Substituted Ketones.. Angew. Chem., Int. Ed. 2013, 52, 9747–9750. 10.1002/anie.201303576. 23934929

[ref86] Lu Y.; Li Y.; Zhang R.; Jin K.; Duan C. Highly Efficient Cu(I)-Catalyzed Trifluoromethylation of Aryl(Heteroaryl) Enol Acetates with CF_3_ Radicals Derived from CF_3_SO_2_Na and TBHP at Room Temperature.. J. Fluorine Chem. 2014, 161, 128–133. 10.1016/j.jfluchem.2014.01.020.

[ref87] There was nothing to integrate in ^19^F NMR spectra at signal/noise > 3000 and in chiral HPLC or GC chromatograms of the samples with concentration of 0.5 mg/mL.

[ref88] Alazet S.; Ismalaj E.; Glenadel Q.; Le Bars D.; Billard T. Acid-Catalyzed Synthesis of α-Trifluoromethylthiolated Carbonyl Compounds.. Eur. J. Org. Chem. 2015, 2015, 4607–4610. 10.1002/ejoc.201500710.

[ref89] Liu J.-B.; Xu X.-H.; Qing F.-L. Silver-Mediated Oxidative Trifluoromethylation of Alcohols to Alkyl Trifluoromethyl Ethers.. Org. Lett. 2015, 17, 5048–5051. 10.1021/acs.orglett.5b02522. 26418394

[ref90] Thompson A. J.; Chamorro Orué A. I.; Nair A. J.; Price J. R.; McMurtrie J.; Clegg J. K. Elastically Flexible Molecular Crystals.. Chem. Soc. Rev. 2021, 50, 11725–11740. 10.1039/D1CS00469G. 34528036

[ref91] Naumov P.; Karothu D. P.; Ahmed E.; Catalano L.; Commins P.; Mahmoud Halabi J.; Al-Handawi M. B.; Li L. The Rise of the Dynamic Crystals.. J. Am. Chem. Soc. 2020, 142, 13256–13272. 10.1021/jacs.0c05440. 32559073

[ref92] Commins P.; Karothu D. P.; Naumov P. Is a Bent Crystal Still a Single Crystal?. Angew. Chem., Int. Ed. 2019, 58, 10052–10060. 10.1002/anie.201814387. 30762922

[ref93] Saha S.; Mishra M. K.; Reddy C. M.; Desiraju G. R. From Molecules to Interactions to Crystal Engineering: Mechanical Properties of Organic Solids.. Acc. Chem. Res. 2018, 51, 2957–2967. 10.1021/acs.accounts.8b00425. 30351918

[ref94] Reddy C. M.; Rama Krishna G.; Ghosh S. Mechanical properties of molecular crystals—applications to crystal engineering.. CrystEngComm 2010, 12, 2296–2314. 10.1039/c003466e.

[ref95] Pisačić M.; Biljan I.; Kodrin I.; Popov N.; Soldin Ž.; Đaković M. Elucidating the Origins of a Range of Diverse Flexible Responses in Crystalline Coordination Polymers.. Chem. Mater. 2021, 33, 3660–3668. 10.1021/acs.chemmater.1c00539.

[ref96] Worthy A.; Grosjean A.; Pfrunder M. C.; Xu Y.; Yan C.; Edwards G.; Clegg J. K.; McMurtrie J. C. Atomic Resolution of Structural Changes in Elastic Crystals of Copper(II) Acetylacetonate.. Nat. Chem. 2018, 10, 65–69. 10.1038/nchem.2848. 29256512

[ref97] Thompson A. J.; Price J. R.; McMurtrie J. C.; Clegg J. K. The Mechanism of Bending in Co-Crystals of Caffeine and 4-Chloro-3-Nitrobenzoic Acid.. Nat. Commun. 2021, 12, 5983 10.1038/s41467-021-26204-z. 34671030 PMC8528856

[ref98] Feiler T.; Michalchuk A. A. L.; Schröder V.; List-Kratochvil E.; Emmerling F.; Bhattacharya B. Elastic Flexibility in an Optically Active Naphthalidenimine-Based Single Crystal.. Crystals 2021, 11, 1397 10.3390/cryst11111397.

[ref99] Reddy C. M.; Gundakaram R. C.; Basavoju S.; Kirchner M. T.; Padmanabhan K. A.; Desiraju G. R. Structural basis for bending of organic crystals.. Chem. Commun. 2005, 3945–3947. 10.1039/b505103g. 16075080

[ref100] Reddy C. M.; Padmanabhan K. A.; Desiraju G. R. Structure–Property Correlations in Bending and Brittle Organic Crystals.. Cryst. Growth Des. 2006, 6, 2720–2731. 10.1021/cg060398w.

[ref101] Bhandary S.; Thompson A. J.; McMurtrie J. C.; Clegg J. K.; Ghosh P.; Mangalampalli S. R. N. K.; Takamizawa S.; Chopra D. The Mechanism of Bending in a Plastically Flexible Crystal.. Chem. Commun. 2020, 56, 12841–12844. 10.1039/D0CC05904H. 32968742

[ref102] For a qualitative comparison of elastic flexibility of **2d** and **4d**, see SI Movie 2d and Movie 4d.

[ref103] Goldberg F. W.; Kettle J. G.; Kogej T.; Perry M. W. D.; Tomkinson N. P. Designing Novel Building Blocks Is an Overlooked Strategy to Improve Compound Quality.. Drug Discovery Today 2015, 20, 11–17. 10.1016/j.drudis.2014.09.023. 25281855

[ref104] Grygorenko O. O.; Volochnyuk D. M.; Vashchenko B. V. Emerging Building Blocks for Medicinal Chemistry: Recent Synthetic Advances.. Eur. J. Org. Chem. 2021, 2021, 6478–6510. 10.1002/ejoc.202100857.

[ref105] Boström J.; Brown D. G.; Young R. J.; Keserü G. M. Expanding the Medicinal Chemistry Synthetic Toolbox.. Nat. Rev. Drug Discovery 2018, 17, 709–727. 10.1038/nrd.2018.116. 30140018

[ref106] Tsoung J.; Krämer K.; Zajdlik A.; Liébert C.; Lautens M. Diastereoselective Friedel–Crafts Alkylation of Hydronaphthalenes.. J. Org. Chem. 2011, 76, 9031–9045. 10.1021/jo201781x. 21951196

[ref107] Cotman A. E.; Modec B.; Mohar B. Stereoarrayed 2,3-Disubstituted 1-Indanols via Ruthenium(II)-Catalyzed Dynamic Kinetic Resolution–Asymmetric Transfer Hydrogenation.. Org. Lett. 2018, 20, 2921–2924. 10.1021/acs.orglett.8b00980. 29746141

[ref108] Keylor M. H.; Matsuura B. S.; Stephenson C. R. J. Chemistry and Biology of Resveratrol-Derived Natural Products.. Chem. Rev. 2015, 115, 8976–9027. 10.1021/cr500689b. 25835567 PMC4566929

[ref109] Karageorgis G.; Foley D. J.; Laraia L.; Waldmann H. Principle and Design of Pseudo-Natural Products.. Nat. Chem. 2020, 12, 227–235. 10.1038/s41557-019-0411-x. 32015480

[ref110] Tomašič T.; Durcik M.; Keegan B. M.; Skledar D. G.; Zajec Ž.; Blagg B. S. J.; Bryant S. D. Discovery of Novel Hsp90 C-Terminal Inhibitors Using 3D-Pharmacophores Derived from Molecular Dynamics Simulations.. Int. J. Mol. Sci. 2020, 21, 6898 10.3390/ijms21186898. 32962253 PMC7555175

[ref111] Dernovšek J.; Zajec Ž.; Durcik M.; Mašič L. P.; Gobec M.; Zidar N.; Tomašič T. Structure-Activity Relationships of Benzothiazole-Based Hsp90 C-Terminal-Domain Inhibitors.. Pharmaceutics 2021, 13, 1283 10.3390/pharmaceutics13081283. 34452244 PMC8400049

