# Supplementary material for: Catalytic Stereoconvergent Synthesis of Homochiral β-CF3, β-SCF3, and β-OCF3 Benzylic Alcohols
Source: ACS Org Inorg Au. 2022 Jun 8;2(5):396–404. doi: 10.1021/acsorginorgau.2c00019 (PMC9542724; doi:10.1021/acsorginorgau.2c00019)
Supplement: Supplementary file 3 — gg2c00019_si_003.pdf [file gg2c00019_si_003.pdf]

Catalytic stereoconvergent synthesis of homochiral β-CF_3, -SCF_3 and -OCF_3 benzylic alcohols

Andrej Emanuel Cotman,*,† Pavel A. Dub,‡ Maša Sterle,† Matic Lozinšek,§ Jaka Dernovšek,† Živa Zajec,† Anamarija Zega,† Tihomir Tomašič,† Dominique Cahard◇

† Faculty of Pharmacy, University of Ljubljana, Aškerčeva cesta 7, SI-1000 Ljubljana, Slovenia
‡ Chemistry Division, Los Alamos National Laboratory, Los Alamos, New Mexico 87545, United States
§ Jožef Stefan Institute, Jamova cesta 39, SI-1000, Ljubljana, Slovenia.
◇ CNRS UMR 6014 COBRA, Normandie Université, 76821 Mont Saint Aignan, France

Supporting Information

Contents

Materials and instrumentation 2
Additional results 3
Synthesis of α-substituted ketones 1a–1r 4
Asymmetric reductions 5
Further synthetic transformations of stereopure products 2 10
Control experiments regarding detrifluoromethylation 14
Determination of stereomeric ratios 15
NMR spectra 34
Computational analysis 63
Design and molecular modeling of compound 10 74
Cell-based assays 75
Single crystal X-ray analysis 76
Photos of mechanically responsive behavior 99

Materials and instrumentation

General. Reactions were conducted under an inert atmosphere using anhydrous solvents when required. For reactions that require heating, an oil bath was used as the heat source. Analytical thin layer chromatography (TLC) was performed on Silica Gel $60F_{254}$ plates. Flash column chromatography was performed using Silica Gel 60 (40–63 μm). The enantiomeric excess (ee) of reduced products was determined by normal phase HPLC analysis on Agilent Technologies 1100 instrument with G1365B UV−vis detector, G1316A thermostat, and G1313A autosampler using Chiralpak AS column (25 cm), Chiralpak IB-3 column, or Kromasil 3-Cellucoat column (15 cm) as specified; or GC analysis on Shimadzu GC-2010 gas chromatograph, equipped with AOC-20i auto injector, helium as a carrier gas, and flame ionisation detector, using CP-ChiraSil-DEX CB column (25 m x 0.25 cm). ^{1}H NMR (400 MHz; internal Me_4Si), ^{13}C NMR (100 MHz; internal Me_4Si), and ^{19}F NMR (380 MHz, external CCl_3F) spectra were recorded on a Bruker AVANCE III 400 spectrometer (Bruker Corporation, Billerica, MA, USA). HRMS were obtained using Exactive Plus Orbitrap mass spectrometer (Thermo Fisher Scientific, Waltham, MA, USA). The catalysts were weighted on Metler Toledo XPR2 balance (± 0.5 μg) and the other reagents on Metler Toledo XSR205 balance (± 10 μg).

Catalysts. (*R*,*R*)-**C1** and (*S*,*S*)-**C3** were purchased from TCI (Tokyo, Japan); (*S*,*S*)-**C2** and (*R*,R)-**C2** were purchased from Sigma-Aldrich (St. Louis, MO, USA) as ruthenium chloride monomers. (*S*,*S*)-**C4** and (3*R*, 1'*S*)-**C5** were prepared according to the literature procedures as μ-(ruthenium dichloride) dimers.[1] All catalysts were activated by stirring in HCO_2H/Et_3N at r.t. for 30 min under a light stream of argon.

Reagents. HCO_2H/Et_3N 5:2 was prepared by adding Et_3N (280 mL, 2 mol) to HCO_2H (189 mL, 5 mol) at 0°C under nitrogen atmosphere and used as such. It was stored at room temperature without any precautions regarding air and moisture. Analogously, HCO_2H/Et_3N 3:2 was prepared by adding Et_3N (280 mL, 2 mol) to HCO_2H (113 mL, 3 mol). The molar ratios were confirmed by ^{1}H NMR analysis (relaxation delay = 25 s).

[1] a) For preparation of **C4**, see: Kišić, A.; Stephan, M.; Mohar, B. *Adv. Synt. Catal.* **2015**, *357*, 2540–2546. b) For preparation of **C5**, see: Cotman, A. E.; Lozinšek, M.; Wang, B.; Stephan, M.; Mohar, B. *Org. Lett.* **2019**, *21*, 3644–3648.

Additional results

Table S1. Additional results on catalyst and solvent screening for Ru(II)-catalyzed DKR-ATH of **1a**.

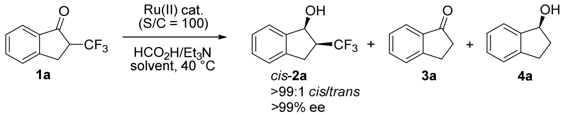

	Ru(II) cat.	F/A	cosolvent	Time	1a:2a:3a:4a
1	(*R*,*R*)-**C1**	3:2	PhCl	1 h	18:62:20:0
				3 h	0:73:19:8
2	(*S*,*S*)-**C2**	3:2	PhCl	1 h	8:67:21:3
				3 h	0:75:6:19
3	(*S*,*S*)-**C3**	3:2	PhCl	1 h	19:59:20:2
				3 h	0:75:19:6
4	(*S*,*S*)-**C4**	3:2	PhCl	1 h	40:33:27:0
				3 h	4:55:35:6
5	(3*R*,1'*S*)-**C5**	3:2	PhCl	1 h	6:70:14:10
				3 h	0:75:0:25
6	(*S*,*S*)-**C2**	3:2	-	1 h	13:24:63:0
				3 h	5:24:71:0
				18 h	0:25:60:16
7	(*S*,*S*)-**C2**	5:2	-	1 h	37:61:2:0
				3 h	21:74:5:0
				18 h	5:75:20:0
8	(*S*,*S*)-**C2**	5:2	PhCl	1 h	15:85:0:0
				3 h	0:99:0:1
9	(*S*,*S*)-**C2**	5:2	DMF	1 h	0:98:1:2
				3 h	0:97:0:3
10	(*S*,*S*)-**C2**	5:2	dioxane	1 h	10:89:1:0
				3 h	0:98:0:2
11	(*S*,*S*)-**C2**	5:2	1,2-DCE	1 h	18:81:0:0
				3 h	0:98:0:2

DKR-ATH of **1a** (50 mg, 0.25 mmol) was carried out using Ru(II) cat. (1 mol%), HCO_2H/Et_3N (0.25 mL) and cosolvent (0.5 mL) at 40 °C. The product ratio was determined by NMR analysis of reaction mixture aliquots, and the ratio of **2a** stereomers (cis/trans >99:1; >99% in all cases) was determined after isolation by HPLC analysis using chiral stationary phase. 1a : 2a : 3a : 4a ratio was determined by ^{1}H NMR (400 MHz, Chloroform-*d*) δ 7.82 (d, *J* = 7.4 Hz, 1H, **1a**), 7.77 (d, *J* = 7.7 Hz, 1H, **3a**), 5.36 (d, *J* = 5.7 Hz, 1H, **2a**), 5.28 – 5.23 (m, 1H, **4a**).

Synthesis of α-substituted ketones 1a–1r

2-(Trifluoromethyl)indanone derivatives **1a–1c** were prepared by construction of the alicyclic ring by acidic activation of 2-(trifluoromethyl)acrylic acid with arenes according to Prakash and Olah's procedure.[2] 2-(Trifluoromethyl)indanone derivatives **1d–1g** and **1j**–**1l** were prepared by radical desulfur-fragmentation and reconstruction of enol triflates according to Yuan and Li's procedure.[3] 2-(Trifluoromethyl)tetralone **1i** was obtained by oxidative trifluoromethylation of the corresponding unactivated olefin as described by Maiti and coworkers.[4] 2-(Trifluoromethyl)benzosuberone **1m** was obtained by radical trifluoromethylation of the corresponding enol acetate by means of sodium triflinate.[5] All these α-CF_3 ketones are known compounds, for which analytical data are identical to those reported in the literature: **1a** CAS [157364-39-1],[2] **1b** CAS [1219921-37-5],[2] **1c** CAS [1219921-42-2],[2] **1d** CAS [2412527-44-5],[6] **1e** CAS [1822781-36-1],[7] **1f** CAS [2615310-31-9],[7] **1g** CAS [1823807-20-0],[7] **1i** CAS [620606-49-7],[4] **1j** CAS [889946-30-9],[8] **1k** CAS [889946-33-2],[7] **1l** CAS [2041791-75-5],[3] **1m** CAS [889946-43-4].[9]

Synthesis of α-SCF_3 ketones. 2-(Trifluoromethylthio)indanone **1n**, 2-(trifluoromethylthio)tetralone **1o**, and 1-(trifluoromethylthio)indan-2-one **1r** were prepared by means of Billard's reagent under acidic conditions from the corresponding carbonyl substrates.[10] Compounds **1n** and **1o** are known compounds, for which analytical data are identical to those reported in the literature: compounds **1n** CAS [1644085-03-9],[10] **1o** CAS [1644085-04-0].[10] 1-(Trifluoromethylthio)indan-2-one **1r** is a new compound.

Synthesis of α-OCF_3 ketone 1p. 2-(Trifluoromethoxy)-2,3-dihydro-1H-inden-1-one **1p** was prepared by a silver-mediated trifluoromethylation of the 2-(hydroxy)indanone under adapted Qing's procedure.[11] Compound **AD21** is a known compound, for which analytical data are identical to those reported in the literature: compounds **AD21** CAS [2226427-07-0],[12]

Synthesis of 7-acetamido-2-trifluoromethyl-1-indanone (1h).

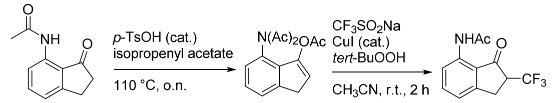

STEP1. 4-(*N*-acetylacetamido)-1*H*-inden-3-yl acetate. A mixture of 7-acetamido-1-indanone (2.0 g, 10.6 mmol), isopropenyl acetate (23 mL, 20 eq) and *p*-TsOH (183 mg, 1.06 mmol, 0.1 eq) was refluxed overnight. After cooling to r.t. the reaction mixture was partitioned between saturated aqueous $NaHCO_3$ and EtOAc, the organic layer washed with brine, dried over Na_2SO_4, filtered through a short pad of silica, and concentrated. The crude product was distilled using Kugel-Rohr apparatus (140 °C, 0.1 mbar) to get the title compound as a white solid (2.82 g, 97% yield). **^{1}H NMR** (303 MHz, Chloroform-*d*) δ 7.49 (dd, *J* = 7.5, 0.9 Hz, 1H), 7.34 (t, *J* = 7.7 Hz, 1H), 7.05 (dd, *J* = 7.8, 1.0 Hz, 1H), 6.48 (t, *J* = 2.3 Hz, 1H), 3.47 (d, *J* = 2.3 Hz, 2H), 2.30 (s, 6H), 2.23 (s, 3H). **^{13}C{^{1}H} NMR** (76 MHz, Chloroform-*d*) δ 173.1, 168.1, 147.6, 144.1, 136.1, 131.1, 127.5, 127.0, 125.0, 118.4, 35.2, 27.0, 21.3.

STEP2. 7-acetamido-2-trifluoromethyl-1-indanone (1h). A mixture of the above enol acetate (1.00 g, 3.66 mmol), sodium trifluoromethynesulfinate (2.28 g, 14.6 mmol), CuI (105 mg, 0.549 mmol) and acetonitrile (18 mL) was cooled to 0 °C and treated dropwise with *tert*-butyl hydroperoxide, 70 % in water (2.65 mL, 18.3 mmol). After stirring at 20 °C for 2 h (full conv. by TLC), the reaction mixture was concentrated and purified by flash chromatography, eluent hexane/EtOAc gradiently from 7:1 to 2:1 (Rf (4:1) = 0.28). Pale yellow solid (226 mg, 24% yield). **^{1}H NMR** (400 MHz, Chloroform-*d*) δ 10.19 (s, 1H), 8.47 (d, *J* = 8.3 Hz, 1H), 7.63 (t, *J* = 7.9 Hz, 1H), 7.15 (dq, *J* = 7.6, 0.9 Hz, 1H), 3.56–3.36 (m, 2H), 3.28 (dd, *J* = 17.0, 3.8 Hz, 1H), 2.25 (s, 3H). **^{19}F NMR** (376 MHz, Chloroform-*d*) δ −67.81 (d, *J* = 9.8 Hz). **^{13}C{^{1}H} NMR** (101 MHz, Chloroform-*d*) δ 199.0 (q, *J* = 1.9 Hz), 169.5, 152.5, 139.4, 138.3, 124.7 (q, *J* = 278.7 Hz), 121.6 (q, *J* = 1.8 Hz), 120.2, 117.5, 50.0 (q, *J* = 27.8 Hz), 27.2 (q, *J* = 2.4 Hz), 25.0. **HRMS** calcd. for $C_{12}H_{11}O_2NF_3$ $[M+H]^+$ 258.0736, found 258.0734 (−1.01 ppm).

[2] Prakash, G. K. S.; Paknia, F.; Vaghoo, H.; Rasul, G.; Mathew, T.; Olah, G. A. *J. Org. Chem.* **2010**, *75*, 2219–2226.
[3] Su, X.; Huang, H.; Yuan, Y.; Li, Y. *Angew. Chem., Int. Ed.* **2017**, *56*, 1338–1341.
[4] Deb, A.; Manna, S.; Modak, A.; Patra, T.; Maity, S.; Maiti, D. *Angew. Chem. Int. Ed.* **2013**, *52*, 9747–9750.
[5] Lu, Y.; Li, Y.; Zhang, R.; Jin, K.; Duan, C. *J. Fluorine Chem.* **2014**, *161*, 128–133.
[6] Trost, B. M.; Hung, C.-I. J.; Mata, G.; Liu, Y.; Lu, Y.; Gnanamani, E. *Org. Lett.* **2020**, *22*, 2437–2441.
[7] Zhu, Y.; Ni, Y.; Lu, C.; Wang, X.; Wang, Y.; Xue, X.-S.; Pan, Y. *Org. Lett.* **2021**, *23*, 2443–2448.
[8] Zhang, R.; Ni, C.; He, Z.; Hu, J. *Top. Catal.* **2018**, *61*, 664–673.
[9] Liu, S.; Jie, J.; Yu, J.; Yang, X. *Adv. Synth. Catal.* **2018**, *360*, 267–271.
[10] Alazet, S.; Ismalaj, E.; Glenadel, Q.; Le Bars, D.; Billard, T. *Eur. J. Org. Chem.* **2015**, *2015*, 4607–4610.
[11] Liu, J.-B.; Xu, X.-H.; Qing, F.-L. *Org. Lett.* **2015**, *17*, 5048–5051.
[12] Duhail, T.; Bortolato, T.; Mateos, J.; Anselmi, E.; Jelier, B.; Togni, A.; Magnier, E.; Dagousset, G.; Dell'Amico, L. *Org. Lett.* **2021**, *23*, 7088–7093.

Asymmetric reductions

General procedure for DKR-ATH. The ruthenium catalyst (*S*,*S*)-**C2** (1.55 mg, 2.5 µmol, 1 mol%) was treated with HCO_2H/Et_3N 5:2 (0.25 mL) and stirred at r.t. for 30 min under a light stream of argon. A solution of the ketone **1** (0.25 mmol) in chlorobenzene (0.5 mL) was then added and the resulting solution was stirred at 40 °C with continued argon sweeping. The reaction was monitored by NMR analysis and after full conversion, the reaction mixture was partitioned between ethyl acetate (10 mL) and water (10 mL). The organic layer was washed with brine, dried over Na_2SO_4, filtered through a short pad of silica, and concentrated to get the crude product **2**. This was optionally purified by flash chromatography (hexane/ethyl acetate) to remove the de-trifluoromethylation side products.

Practical note: This class of compounds is prone to sublimation. It is advisable not to dry the products **2** at reduced pressure under 20 mbar.

General procedure for $NaBH_4$ reduction. To a solution of the ketone **1** (0.25 mmol) in absolute ethanol (1 mL) at 0 °C was added portionwise $NaBH_4$ (24 mg, 2.5 eq). The resulting mixture was stirred at r.t. for 2 h, then it was partitioned between EtOAc and water, and the organic layer concentrated to get the alcohol **2** as a mixture of stereomers. This was used as a standard for determination of diastereomeric ratio by non-decoupled ^{19}F NMR or ^{1}H NMR analysis, and enantiomeric ratio by chiral GC or HPLC analysis.

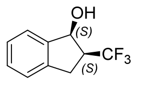

Chemical Formula: $C_{10}H_9F_3O$
Molecular Weight: 202,18

2a. (1*S*,2*S*)-2-(Trifluoromethyl)-1-indanol. Prepared from **1a** (200 mg, 1 mmol) according to the *General procedure for DKR-ATH*. Full conversion after 2 h; 99% NMR yield; 182 mg (90% isol. yield) after column chromatography (hexane/EtOAc 4:1); *cis*/*trans* > 99 :1, >99% ee. White crystals. **^{1}H NMR** (400 MHz, Chloroform-*d*) δ 7.49–7.42 (m, 1H), 7.39–7.25 (m, 3H), 5.40–5.31 (m, 1H), 3.42–3.29 (m, 1H), 3.20–3.04 (m, 2H), 1.77 (d, *J* = 7.5 Hz, 1H). **^{19}F NMR** (376 MHz, Chloroform-*d*) δ −65.42 (d, *J* = 8.9 Hz). **^{13}C NMR{^{1}H}** (101 MHz, Chloroform-*d*) δ 142.2, 140.7, 129.6, 127.7, 126.6 (q, *J* = 277.8 Hz), 125.1, 125.1, 74.5 (q, *J* = 1.9 Hz), 47.4 (q, *J* = 26.1 Hz), 30.4 (q, *J* = 2.6 Hz). **HRMS** calcd. for $C_{10}H_8F_3$ $[M{-}OH]^+$ 185.0573, found 185.0579 (+3.45 ppm); calcd. for $C_{10}H_8OF_3$ $[M{-}H]^-$ 201.05327, found 201.05277 (−2.50 ppm). **SCXRD**. Crystals suitable for X-ray analysis were obtained by recrystallization from EtOAc. See also single-crystal X-ray analysis section.

$NaBH_4$ reduction afforded racemic **2a** in a 97:3 *cis*/*trans* ratio. **^{19}F NMR** (376 MHz, $CDCl_3$) δ –65.43 (d, *J* = 9.2 Hz, *cis*-**2a**), –69.94 (d, *J* = 8.8 Hz, *trans*-**2a**); Chiral **HPLC** analysis on Chiralpak AS column (25 cm), eluent hexane/2-propanol 97:3, flow rate 1 mL/min, λ = 220 nm, t_R = 8.6 min (*S*,*S*) and 13.8 min (*R*,*R*).

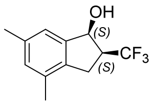

Chemical Formula: $C_{12}H_{13}F_3O$
Molecular Weight: 230,23

2b. (1*S*,2*S*)-4,6-dimethyl-2-(trifluoromethyl)-1-indanol Prepared from **1b** (57 mg, 0.25 mmol) according to the *General procedure for DKR-ATH*. Full conversion after 3 h; >99% NMR yield; 50 mg (87% isol. yield) after extraction; *cis*/*trans* > 99 :1, >99% ee. White crystals. **^{1}H NMR** (400 MHz, Chloroform-*d*) δ 7.08 (s, 1H), 6.98 (s, 1H), 5.28 (t, *J* = 6.5 Hz, 1H), 3.26–2.93 (m, 3H), 2.33 (s, 3H), 2.25 (s, 3H), 1.78 (d, *J* = 7.4 Hz, 1H). **^{19}F NMR** (376 MHz, Chloroform-*d*) δ −65.39 (d, *J* = 8.8 Hz). **^{13}C NMR{^{1}H}** (101 MHz, Chloroform-*d*) δ 142.2, 137.8, 136.7, 134.2, 131.3, 126.8 (q, *J* = 277.8 Hz), 122.8, 74.7 (q, *J* = 2.1 Hz), 47.3 (q, *J* = 26.0 Hz), 28.9 (q, *J* = 2.7 Hz), 21.2, 18.8. **HRMS** calcd. for $C_{12}H_{12}F_3$ $[M{-}OH]^+$ 213.0886, found 213.0887 (+0.56 ppm); calcd. for $C_{12}H_{12}OF_3$ $[M{-}H]^-$ 229.08457, found 229.08459 (+0.07 ppm).

$NaBH_4$ reduction afforded racemic **2b** in a 93:7 *cis*/*trans* ratio. **^{19}F NMR** (376 MHz, $CDCl_3$) δ –65.39 (d, *J* = 8.9 Hz, *cis*-**2b**), –70.04 (d, *J* = 9.5 Hz, *trans*-**2n**); chiral **HPLC** analysis on Kromasil 3-Cellucoat column (15 cm), eluent hexane/2-propanol 98:2, flow rate 1 mL/min, λ = 220 nm, t_R = 5.7 min (*S*,*S*) and 6.3 min (*R*,*R*).

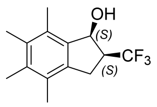

Chemical Formula: $C_{14}H_{17}F_3O$
Molecular Weight: 258,28

2c. (1*S*,2*S*)-4,5,6,7-Tetramethyl-2-(trifluoromethyl)-1-indanol. Prepared from **1c** (256 mg, 1 mmol) according to the *General procedure for DKR-ATH* using (*S*,*S*)-**C2** (31 mg, 5 mol%). Full conversion after 5 h; 96% NMR yield; 227 mg (88% isol. yield) after column chromatography (hexane/EtOAc 9:1); *cis*/*trans* > 99 : 1, >99% ee. White crystals.**^{1}H NMR** (400 MHz, Chloroform-*d*) δ 5.42 (dd, *J* = 8.0, 5.7 Hz, 1H), 3.23 (dd, *J* = 15.2, 9.4 Hz, 1H), 3.13 – 2.94 (m, 2H), 2.33 (s, 3H), 2.22 (s, 3H), 2.21 (s, 3H), 2.20 (s, 3H), 1.51 (d, *J* = 8.0 Hz, 1H). **^{19}F NMR** (376 MHz, Chloroform-*d*) δ −65.42 (d, *J* = 8.9 Hz). **^{13}C NMR{^{1}H}** (101 MHz, Chloroform-*d*) δ 138.1, 136.9, 136.6, 134.8, 130.8, 129.8, 126.8 (q, *J* = 277.6 Hz), 73.9 (q, *J* = 2.2 Hz), 46.9 (q, *J* = 26.0 Hz), 29.9 (q, *J* = 2.6 Hz), 16.4, 16.2, 15.9, 15.8. **HRMS** calcd. for $C_{14}H_{16}F_3$ $[M{-}OH]^+$ 241.1199, found 241.1193 (–2.54 ppm); calcd. for $C_{14}H_{16}OF_3$ $[M{-}H]^-$ 257.1159, found 257.1158 (–0.48 ppm).

$NaBH_4$ reduction afforded racemic **2c** in a 95:5 *cis*/*trans* ratio. **^{19}F NMR** (376 MHz, $CDCl_3$) δ –65.42 (d, *J* = 9.3 Hz, *cis*-**2c**), –71.05 (d, *J* = 10.2 Hz, *trans*-**2c**); Chiral **GC** analysis on CP-ChiraSil-DEX CB column (25 m x 0.25 cm), isothermal elution with helium (150 °C). t_R = 29.5 min (*R*,*R*) and 33.0 min (*S*,*S*).

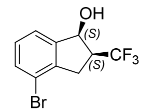

Chemical Formula: $C_{10}H_8BrF_3O$
Molecular Weight: 281,07

(*S*,*S*)-2d. **(1*S*,2*S*)-4-bromo-2-(trifluoromethyl)-1-indanol**. Prepared from **1d** (279 mg, 0.25 mmol) according to the *General procedure for DKR-ATH*. Full conversion after 6 h; 84% NMR yield; 169 mg (60% isol. yield) after column chromatography (hexane/EtOAc 4:1); *cis*/*trans* > 99 : 1, >99% ee. White solid. **^{1}H NMR** (400 MHz, Chloroform-*d*) δ 7.50 (dd, *J* = 7.9, 0.6 Hz, 1H), 7.39 (d, *J* = 7.5 Hz, 1H), 7.19 (t, *J* = 7.7 Hz, 1H), 5.44 (t, *J* = 6.6 Hz, 1H), 3.38 – 3.23 (m, 1H), 3.24 – 3.06 (m, 2H), 1.87 (d, *J* = 7.5 Hz, 1H). **^{19}F NMR** (376 MHz, Chloroform-*d*) δ −65.46 (d, *J* = 8.9 Hz). **^{13}C NMR{^{1}H}** (101 MHz, Chloroform-*d*) δ 143.9, 141.3, 132.6, 129.6, 126.4 (q, *J* = 277.9 Hz), 124.0, 120.1, 75.2 (q, *J* = 2.1 Hz), 46.5 (q, *J* = 26.3 Hz), 32.0 (q, *J* = 2.9 Hz). **HRMS** calcd. for $C_{10}H_7OBrF_3$ $[M-H]^-$ 278.9638, found 278.9641 (+ 1.24 ppm). **SCXRD**. Crystals suitable for X-ray analysis grew after complete evaporation of a dichloromethane solution of pure (*S*,*S*)-**2d.** See also single-crystal X-ray analysis section.

$NaBH_4$ reduction afforded racemic **2d** in a 90:10 *cis*/*trans* ratio accompanied by an unidentified impurity. **^{19}F NMR** (376 MHz, Chloroform-*d*) δ −65.45 (d, *J* = 8.9 Hz, *cis*-**2d**), −70.05 (d, *J* = 8.8 Hz, *trans*-**2d**). Chiral **GC** analysis on CP-ChiraSil-DEX CB column (25 m x 0.25 cm), isothermal elution with helium (130 °C). t_R = 45.5 min (*R*,*R*) and 46.1 min (*S*,*S*).

(*R*,*R*)-2d (1*R*,2*R*)-4-bromo-2-(trifluoromethyl)-1-indanol. Prepared from **1d** (35 mg, 0.13 mmol) according to the *General procedure for DKR-ATH* using HCO_2H/Et_3N 3:2 (0.5 mL), Chlorobenzene (0.5 mL) and (*R*,*R*)-**C2** (1.55 mg, S/C = 50). Full conversion after 1h. After extraction, ^{1}H NMR analysis revealed the crude product (light brown oil) contained **2d**:**4d** = 50:50.[13] This crude product was used as a GC standard for unambiguous determination of enantiomeric purity of the above pure (*S*,*S*)-**2d**, see Determination of stereomeric ratios section. Needle-shaped crystals grew from the crude product after prolonged standing which were determined by **SCXRD** analysis to be (*R*)-**4d**, see also single-crystal X-ray analysis section.

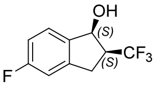

Chemical Formula: $C_{10}H_8F_4O$
Molecular Weight: 220,17

2e. **(1*S*,2*S*)-5-Fluoro-2-(trifluoromethyl)-1-indanol**. Prepared from **1e** (55 mg, 0.25 mmol) according to the *General procedure for DKR-ATH*. Full conversion after 2 h; 99% NMR yield; 47 mg (85% isol. yield) after column chromatography (hexane/EtOAc 4:1); *cis*/*trans* > 99 : 1, >99% ee. White crystals. **^{1}H NMR** (400 MHz, Chloroform-*d*) δ 7.43–7.38 (m, 1H), 7.03–6.94 (m, 2H), 5.37–5.29 (m, 1H), 3.43–3.28 (m, 1H), 3.24–3.05 (m, 2H), 1.79 (d, *J* = 7.3 Hz, 1H). **^{19}F NMR** (376 MHz, Chloroform-*d*) δ –65.56 (d, *J* = 9.4 Hz), –112.08 (app td, *J* = 8.9, 5.3 Hz). **^{13}C NMR{^{1}H}** (101 MHz, Chloroform-*d*) δ 163.8 (d, *J* = 247.4 Hz), 143.2 (d, *J* = 8.8 Hz), 138.0 (d, *J* = 2.5 Hz), 126.6 (d, *J* = 9.3 Hz), 126.4 (q, *J* = 277.8 Hz), 115.0 (d, *J* = 23.0 Hz), 112.0 (d, *J* = 22.6 Hz), 73.6 (q, *J* = 2.0 Hz), 47.8 (q, *J* = 26.2 Hz), 30.5 (app p, *J* = 2.7 Hz). **HRMS** calcd. for $C_{10}H_7F_4$ $[M-OH]^+$ 203.0478, found 203.0480 (+ 0.79 ppm); calcd. for $C_{10}H_7OF_4$ $[M-H]^-$ 219.04385, found 219.04374 (−0.51 ppm).

$NaBH_4$ reduction afforded racemic **2e** in a 94:6 *cis*/*trans* ratio. **^{19}F NMR** (376 MHz, $CDCl_3$) δ –65.55 (d, *J* = 8.9 Hz, *cis*-**2e**), – 70.13 (d, *J* = 8.9 Hz, *trans*-**2e**); chiral **HPLC** analysis on Chiralpak AS column (25 cm), eluent hexane/2-propanol from 98:2 to 90:10 in 20 min, flow rate 1 mL/min, λ = 220 nm, t_R = 10.2 min (*S*,*S*) and 13.2 min (*R*,*R*).

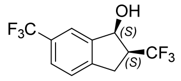

Chemical Formula: $C_{11}H_8F_6O$
Molecular Weight: 270,17

2f. **(1*S*,2*S*)-2,6-bis(trifluoromethyl)-1-indanol**. Prepared from **1f** (70 mg, 0.25 mmol) according to the *General procedure for DKR-ATH*. Full conversion after 3 h; 92% NMR yield; 50 mg (74% isol. yield) after column chromatography (hexane/EtOAc 4:1); *cis*/*trans* > 99 : 1, >99% ee. **^{1}H NMR** (400 MHz, Chloroform-*d*) δ 7.72 (s, 1H), 7.61 (d, *J* = 7.9 Hz, 1H), 7.42 (d, *J* = 8.0 Hz, 1H), 5.43 (t, *J* = 6.4 Hz, 1H), 3.48–3.32 (m, 1H), 3.27–3.05 (m, 2H), 1.96 (d, *J* = 7.1 Hz, 1H). **^{19}F NMR** (376 MHz, Chloroform-*d*) δ −62.24, −65.48 (d, *J* = 8.9 Hz). **^{13}C{^{1}H} NMR** (101 MHz, Chloroform-*d*) δ 130.32 (q, *J* = 1.1 Hz), 126.61 (q, *J* = 3.7 Hz), 126.34 (q, *J* = 277 Hz), 125.45, 124.0 (q, *J* = 272 Hz) 122.3 (q, *J* = 3.8 Hz), 74.0 (q, *J* = 1.9 Hz), 47.4 (q, *J* = 26.3 Hz), 30.5 (q, *J* = 2.9 Hz). **HRMS** calcd. for $C_{11}H_7OF_6$ $[M-H]^-$ 269.0407, found 269.0411 (+1.46 ppm). **SCXRD.** Crystals suitable for X-ray analysis grew after complete evaporation of a dichloromethane solution of pure (*S*,*S*)-**2f**; see also single-crystal X-ray analysis section.

$NaBH_4$ reduction afforded racemic **2f** in a 90:10 *cis*/*trans* ratio. **^{19}F NMR** (376 MHz, Chloroform-*d*) δ −65.46 (d, *J* = 8.8 Hz, *cis*-**2f**), −69.97 (d, *J* = 8.3 Hz, *trans*-**2f**); chiral **GC** analysis on CP-ChiraSil-DEX CB column (25 m x 0.25 cm), isothermal elution with helium (150 °C). t_R = 5.3 min (*R*,*R*) and 5.5 min (*S*,*S*).

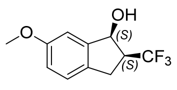

Chemical Formula: $C_{11}H_{11}F_3O_2$
Molecular Weight: 232,20

2g. **(1*S*,2*S*)-6-Methoxy-2-(trifluoromethyl)-1-indanol**. Prepared from **1g** (230 mg, 1.0 mmol) according to the *General procedure for DKR-ATH*. Full conversion after 2 h; >99% NMR yield; 197 mg (85% isol. yield) after extraction; *cis*/*trans* > 99 : 1, >99% ee. White crystals. **^{1}H NMR** (400 MHz, Chloroform-*d*) δ 7.19 (d, *J* = 8.3 Hz, 1H), 6.97 (d, *J* = 2.4 Hz, 1H), 6.89 (dd, *J* = 8.4, 2.5 Hz, 1H), 5.31 (dd, *J* = 7.8, 6.0 Hz, 1H), 3.81 (s, 3H), 3.27 (dd, *J* = 14.7, 8.1 Hz, 1H), 3.22–2.99 (m, 2H), 1.79 (d, *J* = 7.8 Hz, 1H). **^{19}F NMR** (376 MHz, Chloroform-*d*) δ −65.45 (d, *J* = 9.0 Hz). **^{13}C NMR{^{1}H}** (101 MHz, Chloroform-d) δ 159.5, 143.5, 132.4, 126.6 (q, J = 277.9 Hz), 125.7, 116.4, 109.5, 74.6 (q, J = 2.2 Hz), 55.5, 47.8 (q, J = 26.0 Hz), 29.6 (q, J = 2.8 Hz). **HRMS** calcd. for $C_{11}H_{10}OF_3$ $[M-OH]^+$ 215.0678, found 215.0679 (+ 0.44 ppm); calcd. for $C_{11}H_{10}O_2F_3$ $[M-H]^-$ 231.06384, found 231.06387 (+ 0.14 ppm).

[13] ^{1}H NMR analysis of **4d** was referenced to a) Zhang, L.; Tang, Y; Han, Z.; Ding, K. *Angew. Chem. Int. Ed.* **2019**, *58*, 4973–4977.

NaBH$_4$ reduction afforded racemic **2g** in a 92:8 *cis/trans* ratio. **^{19}F NMR** (376 MHz, CDCl$_3$) δ –65.45 (d, *J* = 9.4 Hz, *cis*-**2g**), –69.95 (d, *J* = 8.9 Hz, *trans*-**2g**); chiral HPLC analysis on Chiralpak AS column (25 cm), eluent hexane/2-propanol from 98:2 to 90:10 in 20 min, flow rate 1 mL/min, λ = 220 nm, t$_R$ = 12.7 min (*S*,*S*) and 16.8 min (*R*,*R*).

2h. **(1*S*,2*S*)-7-Acetamido-2-(trifluoromethyl)-1-indanol**. Prepared from **1g** (64 mg, 0.25 mmol) according to the *General procedure for DKR-ATH* using HCO$_2$H/Et$_3$N 3:2. Full conversion after 1 h; 37% NMR yield; 17 mg (26% isol. yield) after flash chromatography (hexane/EtOAc 1:1); *cis/trans* > 99 : 1, >99% ee. White crystals. **^{1}H NMR** (400 MHz, Acetone-*d$_6$*) δ 8.88 (s, 1H), 7.68 (d, *J* = 8.0 Hz, 1H), 7.26 (t, *J* = 7.8 Hz, 1H), 7.08 (d, *J* = 7.5 Hz, 1H), 5.44 (t, *J* = 5.8 Hz, 1H), 4.96 (d, *J* = 5.7 Hz, 1H), 3.31 (dd, *J* = 14.6, 9.2 Hz, 1H), 3.28–3.10 (m, 1H), 3.08 (dd, *J* = 14.8, 7.7 Hz, 1H), 2.15 (s, 3H). **^{19}F NMR** (376 MHz, Acetone-*d$_6$*) δ –65.94 (d, *J* = 9.5 Hz). **^{13}C NMR{^{1}H}** (101 MHz, Acetone-*d$_6$*) δ 169.9, 143.0, 136.0, 135.1, 130.4, 127.9 (q, *J* = 277.2 Hz), 121.7, 121.1, 72.3 (q, *J* = 2.2 Hz), 48.0 (q, *J* = 26.1 Hz), 31.4 (q, *J* = 2.8 Hz), 24.0. **HRMS** calcd. for $C_{12}H_{13}O_2NF_3$ [M+H]$^+$ 260.0893, found 260.0888 (−2.08 ppm).

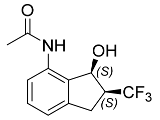

Chemical Formula: $C_{12}H_{12}F_3NO_2$
Molecular Weight: 259,23

NaBH$_4$ reduction afforded racemic **2h** in a 93:7 *cis/trans* ratio. **^{19}F NMR** (376 MHz, Chloroform-*d*) δ −65.53 (d, *J* = 8.9 Hz), −69.89 (d, *J* = 8.7 Hz); chiral **GC** analysis on CP-ChiraSil-DEX CB column (25 m x 0.25 cm), isothermal elution with helium (160 °C). t$_R$ = 54.8 min (*R*,*R*) and 55.6 min (*S*,*S*).

2i. **(1*S*,2*S*)-2-(Trifluoromethyl)-1-tetralol**. Prepared from **1i** (214 mg, 1.00 mmol) according to the *General procedure for DKR-ATH*. Full conversion after 2 h; >99% NMR yield; 207 mg (96% isol. yield) after extraction; *cis/trans* > 99 : 1, >99% ee. Colorless crystals. **^{1}H NMR** (400 MHz, Chloroform-*d*) δ 7.35 (dd, *J* = 7.2, 1.8 Hz, 1H), 7.31–7.22 (m, 2H), 7.19–7.15 (m, 1H), 5.03 (app t, *J* = 3.4 Hz, 1H), 3.02 (dd, *J* = 17.2, 5.6 Hz, 1H), 2.85 (ddd, *J* = 17.6, 12.3, 6.3 Hz, 1H), 2.46 (dqt, *J* = 13.1, 9.1, 2.8 Hz, 1H), 2.18 (app qd, *J* = 12.9, 5.8 Hz, 1H), 2.04 (ddq, *J* = 12.7, 6.0, 2.6, 2.2 Hz, 1H), 1.79 (d, *J* = 4.8 Hz, 1H). **^{19}F NMR** (376 MHz, Chloroform-*d*) δ −68.66 (d, *J* = 8.9 Hz). **^{13}C{^{1}H} NMR** (101 MHz, Chloroform-*d*) δ 136.2, 135.6, 130.0, 129.2, 128.8, 127.1 (q, *J* = 279.5 Hz), 126.7, 65.7 (q, *J* = 2.9 Hz), 44.5 (q, *J* = 25.7 Hz), 28.2, 16.3 (q, *J* = 2.5 Hz). **HRMS** calcd. for $C_{11}H_{10}F_3$ [M−OH]$^+$ 199.0729, found 199.0731 (+ 0.90 ppm); calcd for $C_{11}H_{10}OF_3$ [M−H]$^-$ 215.06892, found 215.06873 (−0.90 ppm).

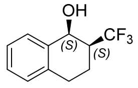

Chemical Formula: $C_{11}H_{11}F_3O$
Molecular Weight: 216,20

NaBH$_4$ reduction afforded racemic **2i** in a 90:10 *cis/trans* ratio. **^{19}F NMR** (376 MHz, CDCl$_3$) δ –68.67 (d, *J* = 9.0 Hz, *cis*-**2i**), –69.76 (d, *J* = 8.5 Hz, *trans*-**2i**); chiral **HPLC** analysis on Chiralpak AS column (25 cm), eluent hexane/2-propanol from 98:2 to 90:10 in 20 min, flow rate 1 mL/min, λ = 220 nm, t$_R$ = 8.4 min (*S*,*S*) and 10.1 min (*R*,*R*).

2j. **(1*S*,2*S*)-6-Methoxy-2-(trifluoromethyl)-1-tetralol**. Prepared from **1k** (61 mg, 0.25 mmol) according to the *General procedure for DKR-ATH*. Full conversion after 5 h; >99% NMR yield; 51 mg (83% isol. yield) after extraction; *cis/trans* > 99 : 1, >99% ee. Colorless crystals. **^{1}H NMR** (400 MHz, Chloroform-*d*) δ 7.26 (d, *J* = 8.5 Hz, 1H), 6.79 (dd, *J* = 8.5, 2.6 Hz, 1H), 6.66 (d, *J* = 2.5 Hz, 1H), 4.99 (t, *J* = 3.7 Hz, 1H), 3.79 (s, 3H), 2.98 (dd, *J* = 17.2, 5.5 Hz, 1H), 2.82 (ddd, *J* = 17.5, 12.4, 6.2 Hz, 1H), 2.42 (dddd, *J* = 13.0, 11.9, 9.1, 6.3 Hz, 1H), 2.16 (qd, *J* = 12.9, 5.8 Hz, 1H), 2.06–1.95 (m, 1H), 1.71 (d, *J* = 5.2 Hz, 1H). **^{19}F NMR** (376 MHz, Chloroform-*d*) δ −68.72 (d, *J* = 9.3 Hz). **^{13}C{^{1}H} NMR** (101 MHz, Chloroform-*d*) δ 159.7, 137.1, 131.3, 128.9, 127.1 (q, *J* = 279.5 Hz), 113.4, 113.1, 65.2 (q, *J* = 2.9 Hz), 55.3, 44.7 (q, *J* = 25.6 Hz), 28.5, 16.3 (q, *J* = 2.5 Hz). **HRMS** calcd. for $C_{12}H_{13}O_2F_3Na$ [M+Na]$^+$ 269.0760, found 269.0755 (−1.73 ppm).

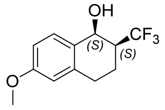

Chemical Formula: $C_{12}H_{13}F_3O_2$
Molecular Weight: 246,23

NaBH$_4$ reduction afforded racemic **2j** in an 81:19 *cis/trans* ratio. **^{19}F NMR** (376 MHz, Chloroform-*d*) δ −68.72 (d, *J* = 9.1 Hz, *cis*-**2j**), −69.73 (d, *J* = 8.8 Hz, *trans*-**2j**); chiral **GC** analysis on CP-ChiraSil-DEX CB column (25 m x 0.25 cm), isothermal elution with helium (170 °C). t$_R$ = 8.2 min (*R*,*R*) and 8.3 min (*S*,*S*).

2k. **(1*S*,2*S*)-7-Methoxy-2-(trifluoromethyl)-1-tetralol**. Prepared from **1k** (61 mg, 0.25 mmol) according to the *General procedure for DKR-ATH*. Full conversion after 2 h; >99% NMR yield; 51 mg (83% isol. yield) after extraction; *cis/trans* > 99 : 1, >99% ee. Colorless crystals. **^{1}H NMR** (400 MHz, Chloroform-*d*) δ 7.08 (d, *J* = 8.8 Hz, 1H), 6.84 (d, *J* = 2.7 Hz, 2H), 4.98 (dd, *J* = 4.7, 2.8 Hz, 1H), 3.80 (s, 3H), 2.94 (dd, *J* = 16.8, 5.5 Hz, 1H), 2.77 (ddd, *J* = 17.3, 12.4, 6.2 Hz, 1H), 2.52–2.35 (m, 1H), 2.14 (qd, *J* = 12.8, 5.7 Hz, 1H), 2.07–1.96 (m, 1H), 1.80 (d, *J* = 5.5 Hz, 1H). **^{13}C{^{1}H} NMR** (101 MHz, Chloroform-*d*) δ 158.2, 137.1, 130.2, 127.5, 127.1 (q, *J* = 279.5 Hz), 115.6, 114.1, 65.9 (q, *J* = 2.9 Hz), 55.3, 44.5 (q, *J* = 25.6 Hz), 27.4, 16.6 (q, *J* = 2.4 Hz). **HRMS** calcd. for $C_{12}H_{12}OF_3$ [M−OH]$^+$ 229.0835, found 229.0840 (+2.20 ppm); calcd. for $C_{12}H_{12}O_2F_3$ [M−H]$^-$ 245.0795, found 245.0791 (− 1.70 ppm). **SCXRD** Crystals suitable for X-ray analysis grew after complete evaporation of a dichloromethane solution of pure (*S*,*S*)-2k; see single-crystal X-ray analysis section.

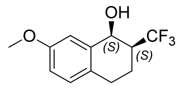

Chemical Formula: $C_{12}H_{13}F_3O_2$
Molecular Weight: 246,23

NaBH$_4$ reduction afforded racemic **2k** in a 91:9 *cis/trans* ratio. **^{19}F NMR** (376 MHz, CDCl$_3$) δ –68.60 (d, *J* = 9.3 Hz, *cis*-**2k**), –69.70 (d, *J* = 8.5 Hz, *trans*-**2k**); chiral **HPLC** analysis on Chiralpak AS column (25 cm), eluent hexane/2-propanol from 98:2 to 90:10 in 20 min, flow rate 1 mL/min, λ = 220 nm, t$_R$ = 11.3 min (*S*,*S*) and 15.7 min (*R*,*R*).

2l. **(3*S*,4*S*)-3-(Trifluoromethyl)-4-chromanol**. Prepared from **1l** (54 mg, 0.25 mmol) according to the *General procedure for DKR-ATH*. Full conversion after 1 h; 98% NMR yield; 46 mg (84% isol. yield) after flash chromatography (hexane/EtOAc 6:1); *cis*/*trans* > 99 : 1, >99% ee. White solid. **^{1}H NMR** (400 MHz, Chloroform-*d*) δ 7.34 – 7.23 (m, 2H), 6.98 (td, *J* = 7.5, 1.1 Hz, 1H), 6.90 (d, *J* = 8.3 Hz, 1H), 5.05–4.98 (m, 1H), 4.41 (ddd, *J* = 10.8, 3.8, 1.5 Hz, 1H), 4.39–4.28 (m, 1H), 2.85–2.72 (m, 1H), 2.06 (d, *J* = 4.6 Hz, 1H). **^{19}F NMR** (376 MHz, Chloroform-*d*) δ −66.02 (d, *J* = 8.9 Hz). **^{13}C{^{1}H} NMR** (101 MHz, Chloroform-*d*) δ 153.6, 130.6, 130.2, 125.5 (q, *J* = 279.4 Hz), 122.2, 121.4, 117.3, 62.2 (q, *J* = 2.8 Hz), 59.0 (q, *J* = 3.3 Hz), 43.5 (q, *J* = 26.1 Hz). **HRMS** calcd. for $C_{10}H_8O_2F_3$ [M−H]$^-$ 217.0482, found 217.0477 (–2.43 ppm).

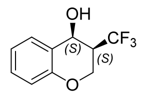

Chemical Formula: $C_{10}H_9F_3O_2$
Molecular Weight: 218,18

NaBH$_4$ reduction afforded racemic **2l** in a 96:4 *cis*/*trans* ratio. **^{19}F NMR** (376 MHz, $CDCl_3$) δ −66.02 (d, *J* = 8.9 Hz, *cis*-**2l**), −68.55 (d, *J* = 9.0 Hz, *trans*-**2l**); chiral **GC** analysis on CP-ChiraSil-DEX CB column (25 m x 0.25 cm), isothermal elution with helium (150 °C). t_R = 6.9 min (*R*,*R*) and 7.2 min (*S*,*S*).

2m. **(1*S*,2*S*)-2-(Trifluoromethyl)-1-benzosuberol**. Prepared from **1m** (57 mg, 0.25 mmol) according to the *General procedure for DKR-ATH* using HCO_2H/Et_3N 3:2. Full conversion after 4 h; 94% NMR yield; 46 mg (80% isol. yield) after flash chromatography (hexane/EtOAc 4:1); *cis*/*trans* = 99.95 : 0.05 (chiral GC), 99.2% ee. Colorless oil. **^{1}H NMR** (400 MHz, Chloroform-*d*) δ 7.24–7.10 (m, 4H), 5.13 (d, *J* = 2.9 Hz, 1H), 3.43–3.31 (m, 1H), 2.66 (ddt, *J* = 14.3, 6.8, 1.3 Hz, 1H), 2.47–2.30 (m, 2H), 2.20–2.06 (m, 1H), 2.11–1.96 (m, 1H), 1.93 (d, *J* = 3.0 Hz, 1H), 1.57–1.42 (m, 1H). **^{19}F NMR** (376 MHz, Chloroform-*d*) δ −70.47 (br s). **^{13}C{^{1}H} NMR** (101 MHz, Chloroform-*d*) δ 142.5, 140.1, 130.7, 128.9, 128.7, 127.5 (q, *J* = 281.2 Hz), 126.3, 73.7 (q, *J* = 3.0 Hz), 47.8 (q, *J* = 23.6 Hz), 34.7, 27.2, 23.4 (q, *J* = 2.5 Hz). **HRMS** calcd. for $C_{12}H_{12}F_3$ [M−OH]$^+$ 213.0886, found 213.0884 (–0.99 ppm).

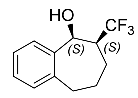

Chemical Formula: $C_{12}H_{13}F_3O$
Molecular Weight: 230,23

NaBH$_4$ reduction afforded racemic **2m** in a 90:10 *cis*/*trans* ratio. Chiral **GC** analysis on CP-ChiraSil-DEX CB column (25 m x 0.25 cm), isothermal elution with helium (150 °C). t_R = 9.3 min (*trans*), 9.8 min (*S*,*S*), 10.3 min (*ent-trans*), 11.1 min (*R*,*R*). Diastereomeric ratio was determined by chiral GC analysis (see below) as the crude ^{19}F NMR spectrum was difficult to interpret.

2n. **(1*R*,2*S*)-2-(Trifluoromethylthio)-1-indanol**. Prepared from **1n** (58 mg, 0.25 mmol) according to the *General procedure for DKR-ATH*. Full conversion after 2 h; >99% NMR yield; 60 mg (100% isol. yield) after extraction; *cis*/*trans* = 99.9 : 0.1, 99.8% ee. Colorless crystals. **^{1}H NMR** (400 MHz, Chloroform-*d*) δ 7.45 (d, *J* = 7.3 Hz, 1H), 7.36–7.26 (m, 3H), 5.14 (d, *J* = 5.1 Hz, 1H), 4.02 (ddd, *J* = 8.9, 7.7, 5.2 Hz, 1H), 3.35 (dd, *J* = 16.0, 7.7 Hz, 1H), 3.20 (dd, *J* = 15.9, 8.9 Hz, 1H), 2.22 (s, 1H). **^{19}F NMR** (376 MHz, Chloroform-*d*) δ −40.24. **^{13}C{^{1}H} NMR** (101 MHz, Chloroform-*d*) δ 141.9, 141.2, 131.0 (q, *J* = 306.4 Hz), 129.6, 127.6, 125.2, 124.8, 75.6, 49.0 (q, *J* = 1.6 Hz), 37.8. **HRMS** calcd. for $C_{10}H_8OF_3S$ [M−H]$^-$ 233.0253, found 233.0254 (+0.11 ppm). **SCXRD.** Crystals suitable for X-ray analysis grew after complete evaporation of a dichloromethane solution of pure (*S*,*S*)-**2n**; see also single-crystal X-ray analysis section.

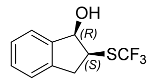

Chemical Formula: $C_{10}H_9F_3OS$
Molecular Weight: 234,24

NaBH$_4$ reduction afforded racemic **2n** in a 42:58 *cis*/*trans* ratio. **^{19}F NMR** (376 MHz, Chloroform-*d*) δ −39.60 (*trans*-**2n**), −40.24 (*cis*-**2n**); chiral **GC** analysis on CP-ChiraSil-DEX CB column (25 m x 0.25 cm), isothermal elution with helium (150 °C). t_R = 8.2 min (1*S*,2*R*) and 8.5 min.

2o. **(1*R*,2*S*)-2-(Trifluoromethylthio)-1-tetralol**. Prepared from **1o** (246 mg, 1.00 mmol) according to the *General procedure for DKR-ATH*. Full conversion after 2 h; >99% NMR yield; 244 mg (98% isol. yield) after extraction; *cis*/*trans* = 99.9 : 0.1, >99% ee. Colorless crystals.d.r. = 99.9:0.1, 99.8% ee. **^{1}H NMR** (400 MHz, Chloroform-*d*) δ 7.41–7.33 (m, 1H), 7.31–7.19 (m, 2H), 7.18–7.10 (m, 1H), 4.89 (dd, *J* = 6.0, 3.4 Hz, 1H), 3.67 (dt, *J* = 11.6, 3.3 Hz, 1H), 3.02 (ddd, *J* = 17.5, 6.0, 3.9 Hz, 1H), 2.91 (ddd, *J* = 17.2, 10.3, 6.1 Hz, 1H), 2.30 (dddd, *J* = 13.3, 11.6, 10.3, 5.9 Hz, 1H), 2.23 (d, *J* = 6.0 Hz, 1H), 2.21 – 2.13 (m, 1H). **^{19}F NMR** (376 MHz, Chloroform-*d*) δ −39.63. **^{13}C{^{1}H} NMR** (101 MHz, Chloroform-*d*) δ 136.1, 135.1, 131.2 (q, *J* = 306.3 Hz), 129.6, 129.0, 128.7, 126.7, 69.4, 47.9 (q, *J* = 1.6 Hz), 28.5, 25.2. **HRMS** calcd. for $C_{11}H_{10}F_3S$ [M−OH]$^+$ 231.0450, found 231.0450 (+0.08 ppm). **SCXRD.** Crystals suitable for X-ray analysis grew after complete evaporation of a dichloromethane solution of pure (*S*,*S*)-**2o**; see also single-crystal X-ray analysis section.

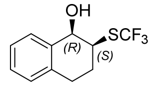

Chemical Formula: $C_{11}H_{11}F_3OS$
Molecular Weight: 248,26

NaBH$_4$ reduction afforded racemic **2o** in a 70:30 *cis*/*trans* ratio. **^{19}F NMR** (376 MHz, Chloroform-*d*) δ −38.85 (s, *trans*-**2o**),−39.63 (s, *cis*-**2o**); chiral **GC** analysis on CP-ChiraSil-DEX CB column (25 m x 0.25 cm), isothermal elution with helium (150 °C). t_R = 12.4 min (1*S*,2*R*) and 12.9 min (1*R*,2*S*).

2p. **(1*R*,2*S*)-2-(Trifluoromethoxy)-1-indanol**. Prepared from **1p** (20 mg, 0.092 mmol) according to the *General procedure for DKR-ATH* using HCO_2H/Et_3N 3:2. Full conversion after 1 h; >99% NMR yield; 16 mg (80% isol. yield) after extraction; *cis*/*trans* = 98:2, 95.7% ee. Colorless crystals. **^{1}H NMR** (400 MHz, Chloroform-*d*) δ 7.50 – 7.43 (m, 1H), 7.38 – 7.27 (m, 2H), 7.31 – 7.23 (m, 1H), 5.16 (dd, *J* = 7.6, 4.9 Hz, 1H), 4.93 (dt, *J* = 5.8, 4.6 Hz, 1H), 3.28 (dd, *J* = 16.4, 4.4 Hz, 1H), 3.18 (dd, *J* = 16.4, 5.8 Hz, 1H), 2.28 (d, *J* = 7.9 Hz, 1H). **^{19}F NMR** (376 MHz, Chloroform-*d*) δ −58.49. **^{13}C{^{1}H} NMR** (101 MHz, Chloroform-*d*) δ 140.7,

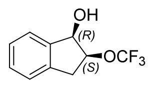

Chemical Formula: $C_{10}H_9F_3O_2$
Molecular Weight: 218,18

138.4, 129.4, 127.7, 125.1, 124.8, 121.8 (q, J = 255.8 Hz), 79.8 (q, J = 2.2 Hz), 74.9, 36.0. **HRMS** calcd. for $C_{10}H_9O_2F_3Na$ $[M+Na]^+$ 241.0447, found 241.0444 (−1.18 ppm). **SCXRD.** Crystals suitable for X-ray analysis grew after complete evaporation of a dichloromethane solution of pure (*S*,*S*)-**2p**; see also single-crystal X-ray analysis section.

$NaBH_4$ reduction afforded racemic **2p** in a 67:33 *cis*/*trans* ratio. **^{19}F NMR** (376 MHz, Chloroform-*d*) δ −58.50, −58.75; chiral **GC** analysis on CP-ChiraSil-DEX CB column (25 m x 0.25 cm), isothermal elution with helium (130 °C). t_R = 6.0 min (1*S*,2*R*) and 6.4 min (1*R*,2*S*).

2q. 3,3,3-trifluoro-2-methyl-1-phenylpropan-1-ol. Prepared from **1r** (51 mg, 0.25 mmol) according to the *General procedure for DKR-ATH*. Full conversion after 7 h; >99% NMR yield; 47 mg (92% isol. yield) after extraction; *d.r.* = 80:20, 97.5% ee. Colorless oil. Major stereomer, (*S*,*S*)-**2q**: **^{1}H NMR** (400 MHz, Chloroform-*d*) δ 7.43–7.21 (m, 5H), 5.18 (d, J = 2.8 Hz, 1H), 3.17 (br s, 1H), 2.52–2.37 (m, 1H), 1.08 (d, J = 7.1 Hz, 3H). **^{19}F NMR** (376 MHz, Chloroform-*d*) δ −69.98 (d, J = 9.5 Hz).[14] The absolute configuration was assigned by analogy to **2a** and **2k**.

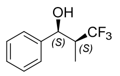

Chemical Formula: $C_{10}H_{11}F_3O$
Molecular Weight: 204,19

$NaBH_4$ reduction afforded racemic **2q** in a 86 : 14 (*R*,S**)/(*R*,R**). ^{19}F NMR (376 MHz, Chloroform-*d*) δ −68.59 (d, J = 8.8 Hz), −70.13 (d, J = 9.5 Hz); chiral **GC** analysis on CP-ChiraSil-DEX CB column (25 m x 0.25 cm), isothermal elution with helium (130 °C). t_R = 6.9 min (*S*,*S*), 7.1 min (*R*,S**), 7.4 min (*R*,*R*), 8.1 min (*R*,S**)

2r. ***cis*-1-(Trifluoromethylthio)-2-indanol**. Prepared from **1r** (58 mg, 0.25 mmol) according to the *General procedure for DKR-ATH*, using HCO_2H/Et_3N 3:2. Full conversion after 18 h; >99% NMR yield; 52 mg (90 % isol. yield) after extraction; *cis*/*trans* = 95:5 (by ^{1}H NMR and GC-FID), 45% ee. Off-white solid. **^{1}H NMR** (400 MHz, Chloroform-*d*) δ 7.47–7.36 (m, 1H), 7.28 (d, J = 2.7 Hz, 3H), 4.83 (d, J = 4.6 Hz, 1H), 4.70 (qd, J = 4.8, 2.5 Hz, 1H), 3.20 (dd, J = 16.4, 5.1 Hz, 1H), 3.07 (dd, J = 16.4, 2.5 Hz, 1H), 2.30 (d, J = 4.6 Hz, 1H). **^{19}F NMR** (376 MHz, Chloroform-*d*) δ −39.66. **^{13}C{^{1}H} NMR** (101 MHz, Chloroform-*d*) δ 140.3, 137.9, 130.8 (q, J = 307.2 Hz), 129.0, 127.6, 125.5, 125.4, 73.7, 55.1 (q, J = 1.8 Hz), 40.0. **HRMS** calcd. for $C_{10}H_8OF_3S$ $[M-H]^-$ 233.0253, found 233.0252 (−0.44 ppm).

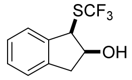

Chemical Formula: $C_{10}H_9F_3OS$
Molecular Weight: 234,24

$NaBH_4$ reduction afforded racemic **2r** in a 2:3 *cis*/*trans* ratio. *trans*-**2r**: **^{1}H NMR** (400 MHz, Chloroform-*d*) δ 7.46–7.36 (m, 1H), 7.34–7.24 (m, 3H), 4.74–4.66 (m, 1H), 4.62 (d, J = 3.7 Hz, 1H), 3.43 (dd, J = 16.5, 6.3 Hz, 1H), 2.95 (dd, J = 16.5, 4.2 Hz, 1H), 2.17 (d, J = 5.1 Hz, 1H). chiral **GC** analysis on CP-ChiraSil-DEX CB column (25 m x 0.25 cm), isothermal elution with helium (130 °C). t_R = 14.6 min (minor *cis*-**2r**), 15.0 min (major *cis*-**2r**), 15.3 min (major *trans*-**2r**), 16.5 min (minor *trans*-**2r**).

[14] NMR data is in accordance with the literature data for racemic (*R**,*R**)-**2q**. Yasu, Y.; Koike, T.; Akita, M. *Angew. Chem. Int. Ed.* **2012**, *51*, 9567–9571.

Further synthetic transformations of stereopure products 2

(1*S*,2*S*)-1-(3-Chloro-4-methoxyphenyl)-6-methoxy-2-(trifluoromethyl)-indan (5).

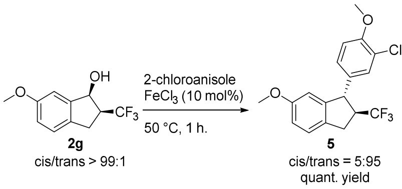

To a solution of (*S*,*S*)-**2g** (116 mg, 0.5 mmol) in 2-chloroanisole (2.5 mL) was added anhydrous $FeCl_3$ (8 mg, 0.05 mmol) and the reaction mixture was stirred at 50 °C for 1 h. The resulting amber solution was filtered through a short pad of silica, eluent EtOAc, and concentrated under reduced pressure. The residual 2-chloroanisole was separated from the product by flash chromatography, eluent hexane/EtOAc 6:1 to get the title compound as a colorless oil (178 mg, quant. yield), d.r. ≥ 95:5. The 1,2-trans disposition was confirmed by the ^1H-^1H NOESY NMR experiment, see below. Major stereomer: **^{1}H NMR** (400 MHz, Chloroform-*d*) δ 7.19–7.14 (m, 1H), 7.05 (dd, *J* = 8.4, 2.2 Hz, 1H), 6.88 (d, *J* = 8.4 Hz, 1H), 6.80 (ddd, *J* = 8.3, 2.5, 0.9 Hz, 1H), 6.38 (d, *J* = 2.4 Hz, 1H), 4.43 (d, *J* = 7.5 Hz, 1H), 3.90 (s, 2H), 3.70 (s, 2H), 3.32 – 3.19 (m, 1H), 3.17 – 3.03 (m, 2H). **^{19}F NMR** (376 MHz, Chloroform-*d*) δ −70.12 (d, *J* = 8.2 Hz). **^{13}C{^{1}H} NMR** (101 MHz, Chloroform-*d*) δ 159.7, 154.2, 145.6, 136.4, 132.3, 130.0, 127.9, 127.9 (q, *J* = 278.1 Hz), 125.1, 122.8, 114.2, 112.3, 110.2, 56.3, 55.6, 52.9 (q, *J* = 26.3 Hz), 51.3 (q, *J* = 2.5 Hz), 31.6 (q, *J* = 2.7 Hz). **HRMS** calcd. for $C_{18}H_{17}O_2ClF_3$ $[M+H]^+$ 357.0864, found 357.0856 (−2.24 ppm).

(1*S*,2*S*)-1-azido-2-trifluoromethylthiotetralin (6).

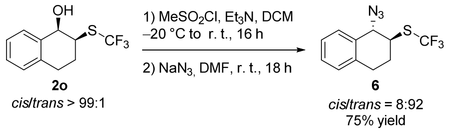

STEP 1. (1*R*,2*S*)-2-(trifluoromethylthio)-1-tetralyl mesylate. To a stirred solution of **2o** (124 mg, 0.5 mmol) in dry dichloromethane (5.0 mL, 0.1 M) at −20 °C under argon atmosphere were added Et_3N (0.11 mL, 1.5 eq) and mesyl chloride (50 µL, 1.3 eq) successively. The reaction was stirred at −20 °C for 2 h, then it was allowed to warm to r.t. overnight. The volatiles were removed under reduced to get the crude mesylate which was used as such in the next reaction step.

STEP 2. Compound 6. The above crude mesylate was taken up in dry dimethylformamide (5.0 mL, 0.1M), NaN_3 was added at room temperature and the reaction mixture was stirred overnight. The resulting solution was partitioned between water (50 mL) and Et_2O (50 mL). The organic layer was then washed with brine, dried over Na_2SO_4, filtered, and concentrated. The crude was purified by flash chromatography, eluent hexane (R_f = 0.16) to yield the title compound as colorless oil (102 mg, 75% yield), d.r. = 92:8. Major stereomer: **^{1}H NMR** (400 MHz, Chloroform-*d*) δ 7.36–7.30 (m, 1H), 7.30–7.27 (m, 1H), 7.24–7.16 (m, 2H), 4.77 (d, *J* = 3.3 Hz, 1H), 3.65–3.57 (m, 1H), 3.07–2.89 (m, 2H), 2.35–2.17 (m, 2H). **^{19}F NMR** (376 MHz, Chloroform-*d*) δ −40.07 (s). **^{13}C{^{1}H} NMR** (101 MHz, Chloroform-*d*) δ 135.1, 132.1, 131.1 (q, *J* = 307.4 Hz), 129.6, 129.4, 129.4, 126.6, 63.2, 45.2 (q, *J* = 2.4 Hz), 28.8, 26.0. HRMS was not obtained due to compound's low ionization.

(1*S*,2*S*)-1-amino-2-trifluoromethylthiotetralin (7).

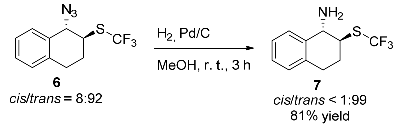

The above azide **6** (50 mg, 0.18 mmol) was dissolved in methanol (2 mL) in a 10 mL round bottom flask, then 10% Pd/C (7,5 mg) was added. The reaction mixture was evacuated and back filled with hydrogen (1 atm). This process was repeated 4 times every 45 minutes until the starting material was consumed (TLC). The reaction mixture was filtered through Celite® and concentrated. The *trans*-isomer was isolated by flash chromatography, eluent EtOAc/toluene 4:1, to yield the title compound as colorless oil, d.r. > 99:1 (36 mg, 81% yield). **^{1}H NMR** (400 MHz, Chloroform-*d*) δ δ 7.34–7.28 (m, 1H), 7.24–7.17 (m, 2H), 7.11 (m, 1H), 4.18 (d, *J* = 4.1 Hz, 1H), 3.70–3.59 (m, 1H), 3.07–2.84 (m, 2H), 2.30–2.13 (m, 2H), 1.60 (s, 3H). **^{19}F NMR** (376 MHz, Chloroform-*d*) δ −39.67 (s). **^{13}C{^{1}H} NMR** (101 MHz, Chloroform-*d*) δ 139.0, 134.4, 131.5 (q, *J* = 306.4 Hz), 129.1, 129.0, 127.6, 126.5, 52.2, 48.6–48.5 (m), 28.6, 25.4. **HRMS** calcd. for $C_{11}H_{13}NF_3S$ $[M+H]^+$ 248.0715, found 248.0711 (−1.90 ppm).

(1*S*,2*S*)-1-(Propargyloxy)-2-(trifluoromethyl)-tetralin (8)

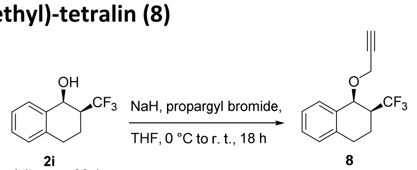

2i (108 mg, 0.5 mmol) and sodium hydride, 60% dispersion in mineral oil (60 mg, 3 eq), were taken up in dry THF at 0 °C. After stirring at 0 °C for 30 min, propargyl bromide, 80% solution in toluene (0.37 mL, 5 eq.), was added and the reaction was stirred overnight at room temperature. The reaction mixture was concentrated and purified by flash chromatography, eluent hexane/EtOAc 8:1 [R_f (hexane/EtOAC 6:1) = 0.45], to yield the title compound as colorless oil (113 mg, 89% yield), d.r. > 99:1. **^{1}H NMR** (400 MHz, Chloroform-*d*) δ 7.36–7.27 (m, 2H), 7.23–7.17 (m, 2H), 4.92 (d, *J* = 2.1 Hz, 2H), 4.22 (dd, J_1 = 16.1 Hz, J_2 = 2.4 Hz, 1H), 4.06 (dd, J_1 = 16.1 Hz, J_2 = 2.4 Hz, 1H), 3.14–3.01 (m, 1H), 2.93–2.79 (m, 1H), 2.57–2.41 (m, 2H), 2.39–2.24 (m, 1H), 2.08–1.95 (m, 1H). **^{19}F NMR** (376 MHz, Chloroform-*d*) δ −68.66 (d, *J* = 8.8 Hz). **^{13}C{^{1}H} NMR** (101 MHz, Chloroform-*d*) δ 136.5, 132.8, 130.2, 129.6, 129.0, 126.9 (q, *J* = 279.5 Hz), 125.6, 79.6, 74.9, 70.7 (q, *J* = 3.0 Hz), 55.2, 43.9 (q, *J* = 26.6 Hz), 27.5, 16.6 (q, *J* = 2.9 Hz). **HRMS** calcd. for $C_{14}H_{14}OF_3$ $[M+H]^+$ 255.0991, found 255.0989 (−0.97 ppm).

3-((1*S*,2*S*)-1-Hydroxy-2-(trifluoromethyl)-indan-4-yl)benzoic acid (9)

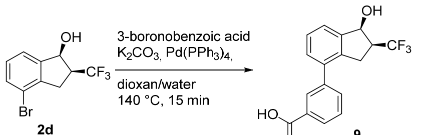

2d (50 mg, 0.18 mmol), 3-boronobenzoic acid (30 mg, 0.18 mmol), potassium carbonate (62 mg, 0.45 mmol) and tetrakis(triphenylphosphine)palladium (2.1 mg, 0.0018 mmol) were dissolved in a degassed mixture of water/dioxane 1:3 (3 mL), and the reaction mixture was heated to 140 °C for 15 min in a microwave reactor. The volatiles were removed under reduced pressure, and the crude was purified by flash chromatography, eluent DCM/MeOH/CH_3COOH 20:1:0.1 (R_f = 0.27). The solvent was then co-evaporated with toluene to yield the title compound as colorless oil (50 mg, 87% yield), d.r. > 99:1. **^{1}H NMR** (400 MHz, Chloroform-*d*) δ 8.20–8.17 (m, 1H), 8.16–8.11 (m, 1H), 7.70–7.65 (m, 1H), 7.61–7.54 (m, 1H), 7.52–7.47 (m, 1H), 7.44–7.39 (m, 2H), 5.41 (d, *J* = 5.5 Hz, 1H), 3.54–3.42 (m, 1H), 3.14–3.00 (m, 2H). **^{19}F NMR** (376 MHz, Chloroform-*d*) δ −65.34 (d, *J* = 8.7 Hz). **^{13}C{^{1}H} NMR** (101 MHz, Chloroform-*d*) δ 171.6, 143.0, 140.5, 138.6, 133.6, 130.1, 130.0, 129.7, 129.2, 128.9, 128.50, 126.5 (q, *J* = 277.9 Hz), 124.7, 74.4 (q, *J* = 3.1 Hz), 47.6 (q, *J* = 26.2 Hz), 30.1 (q, *J* = 3.3 Hz), traces of toluene at 137.9, 129.0, 128.3, 125.3 and 21.5 ppm. **HRMS** calcd. for $C_{17}H_{14}O_3F_3$ $[M+H]^+$ 323.0890, found 323.0888 (−0.39 ppm).

(*S*)-2-Trifluoromethyl-1-indanone, (*S*)-1a.

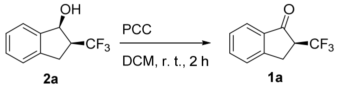

2a (50 mg, 0.25 mmol) was dissolved in dry dichloromethane (5 mL) and treated with a homogenous mixture of pyridinum chlorochromate (PCC) (270 mg, 1.25 mmol) and Celite® (330 mg). The resulting slurry was stirred for 2 h at room temperature, then it was filtered through silica, eluent DCM, to get the title compound as a white solid (46 mg, 92% yield) (3 mol% of residual **2a**), 57% ee. **^{1}H NMR** (400 MHz, Chloroform-*d*) δ 7.84–7.81 (m, 1H), 7.67 (td, J_1 = 7.6 Hz, J_2 = 1.2 Hz, 1H), 7.54–7.51 (m, 1H), 7.47–7.41 (m, 1H), 3.52–3.27 (m, 3H). **^{19}F NMR** (376 MHz, Chloroform-*d*) δ −67.74 (d, *J* = 9.8 Hz). NMR data is identical to the reported data for racemic compound **1a**, see page S4.

Enantiomeric excess was determined by chiral **HPLC** analysis on Chiralpak IB-3 column (25 cm), eluent hexane/2-propanol 98:2, flow rate 1 mL/min, λ = 254 nm, t_R = 7.8 min (*R*)-**1a** and 8.3 min (*S*)-**1a**.

(*S*)-4,5,6,7-tetramethyl-2-Trifluoromethyl-1-indanone, (*S*)-1c.

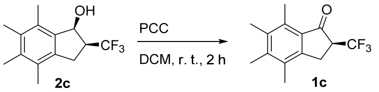

2c (50 mg, 0.19 mmol) was dissolved in dry dichloromethane (5 mL) and treated with a homogenous mixture of pyridinum chlorochromate (PCC) (205 mg, 0.95 mmol) and Celite® (250 mg). The resulting slurry was stirred for 2 h at room temperature, then it was filtered through silica, eluent DCM, and concentrated. The crude was purified by flash chromatography, gradient elution with hexane/EtOAc from 9:1 to 4:1, to get the title compound as a white solid (38 mg, 78% yield), 92% ee. **^{1}H NMR** (400 MHz, Chloroform-*d*) δ 3.43–3.30 (m, 1H), 3.29–3.20 (m, 1H), 3.12–3.04 (m, 1H), 2.62 (s,

1H), 2.31 (s, 1H), 2.27 (s, 1H), 2.24 (s, 1H). **^{19}F NMR** (376 MHz, Chloroform-*d*) δ −67.94 (d, *J* = 10.2 Hz). NMR data is identical to the reported data for racemic compound **1c**, see page S4.

Enantiomeric excess was determined by chiral **GC** analysis on CP-ChiraSil-DEX CB column (25 m x 0.25 cm), isothermal elution with helium (140 °C). t_R = 36.6 min (*R*)-**1c** and 38.3 min (*S*)-**1c**.

Synthesis of compound 10

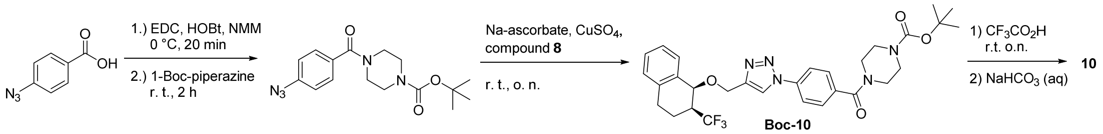

***tert*-Butyl 4-(4-azidobenzoyl)piperazine-1-carboxylate.**[15] *p*-Azidobenzoic acid (163 mg, 1 mmol) was dissolved in DMF. 1-Ethyl-3-carbodiimide (EDC, 202 mg, 1.3 eq), 1-hydroxybenzotriazole (HOBt, 176 mg, 1.3 eq) and N-methylmorpholine (NMM, 0.220 mL, 2 eq) were added at 0 °C. The reaction mixture was stirred on ice bath for 20 minutes, then 1-Boc-piperazine (224 mg, 1.2 eq) was added and the reaction was stirred at room temperature for 2 h. The solvent was then evaporated, and the residue was taken up in ethyl acetate (15 mL) and washed with 1 % citric acid (2 × 15 mL), 1M NaOH (2 × 15 mL) and brine (15 mL). The organic layer was then dried over Na_2SO_4 and the volatiles were evaporated under reduced pressure to yield the title compound as an off-white powder (307 mg, 93% yield). **^{1}H NMR** (400 MHz, Chloroform-*d*) δ 7.45–7.40 (m, 2H), 7.09–7.05 (m, 2H), 3.81–3.33 (m, 8H), 1.47 (s, 9H). **MS** (ESI+) calcd. for $C_{16}H_{21}N_5O_3$ $[M+H]^+$ 332.2, found 332.0.

Boc-10. tert-butyl 4-(4-(4-((((1S,2S)-2-(trifluoromethyl)-1,2,3,4-tetrahydronaphthalen-1-yl)oxy)methyl)-1H-1,2,3-triazol-1-yl)benzoyl)piperazine-1-carboxylate. The above azide (78.2 mg, 0.234 mmol), sodium ascorbate (18.7 mg, 0.4 eq.) and copper(II) sulfate (7.53 mg, 0.2 eq) were dissolved in the mixture of MeOH and water (3 mL/3 mL). Compound **8** (60 mg, 1 eq.) was separately dissolved in 3 mL of MeOH and was added to the reaction mixture. The reaction was stirred overnight at room temperature. Then the methanol was evaporated, and the residue was diluted with water (15 mL) and extracted with ethyl acetate (20 mL). The organic layer was washed with brine (20 mL), dried over Na_2SO_4 and the volatiles were evaporated under reduced pressure. The product was further purified by flash chromatography, eluent EtOAc/Hexane 1:1 (R_f = 0.17), to yield the title compound as colorless oil (91 mg, 66% yield). **^{1}H NMR** (400 MHz, Chloroform-*d*) δ 7.93 (s, 1H), 7.85–7.76 (m, 2H), 7.62–7.54 (m, 2H), 7.38–7.27 (m, 2H), 7.28–7.17 (m, 2H), 4.86–4.77 (m, 2H), 4.74 (d, *J* = 2.1 Hz, 1H), 3.79–3.39 (m, 8H), 3.14–3.04 (m, 1H), 2.95–2.82 (m, 1H), 2.58–2.46 (m, 1H), 2.45–2.30 (m, 1H), 2.08–2.00 (m, 1H), 1.48 (s, 9H). **^{19}F NMR** (376 MHz, Chloroform-*d*) δ −68.46 (d, *J* = 8.8 Hz). **^{13}C{^{1}H} NMR** (101 MHz, Chloroform-*d*) δ 169.2, 154.5, 146.8, 138.0, 136.2, 135.7, 133.1, 130.2, 129.6, 129.1, 128.8, 127.1 (q, *J* = 279.7 Hz), 125.8, 120.6, 120.5, 80.5, 72.7 (q, *J* = 2.9 Hz), 62.4, 48.0–47.3 (m), 44.0 (q, *J* = 26.3 Hz), 44.6–42.9 (m), 42.5–42.0 (m), 28.4, 27.5, 16.7 (q, *J* = 2.3 Hz). **MS** (ESI+) calcd. for $C_{30}H_{34}F_3N_5O_4$ $[M+H]^+$ 586.3, found 586.2.

10. Piperazin-1-yl(4-(4-((((1S,2S)-2-(trifluoromethyl)-1,2,3,4-tetrahydronaphthalen-1-yl)oxy)methyl)-1H-1,2,3-triazol-1-yl)phenyl)methanone. **Boc-10** (58.6 mg, 0.1 mmol) was taken up in dichloromethane (5 mL) and trifluoroacetic acid (0.191 mL, 25 eq.) was added. The reaction mixture was stirred overnight at room temperature. The volatiles were evaporated, and the residue was taken up in ethyl acetate (10 mL) and washed with $NaHCO_3$ (2 × 10 mL), brine (10 mL) and dried over Na_2SO_4. The product was purified by column chromatography, eluent DCM/MeOH/NH_4OH 9:1:0.1 (R_f = 0.29), to yield the title compound as a crispy foam (26.2 mg, 54% yield). **^{1}H NMR** (400 MHz, Chloroform-*d*) δ 7.93 (s, 1H), 7.83–7.75 (m, 2H), 7.62–7.54 (m, 2H), 7.38–7.27 (m, 2H), 7.28–7.17 (m, 2H), 4.86–4.77 (m, 2H), 4.74 (d, *J* = 2.1 Hz, 1H), 3.85–3.72 (m, 2H), 3.49–3.36 (m, 2H), 3.14–3.03 (m, 1H), 3.02–2.78 (m, 5H), 2.58–2.45 (m, 1H), 2.44–2.31 (m, 1H), 2.08–2.00 (m, 1H) . **^{19}F NMR** (376 MHz, Chloroform-*d*) δ −68.47 (d, *J* = 8.6 Hz). **^{13}C{^{1}H} NMR** (101 MHz, Chloroform-*d*) δ 169.0, 146.7, 137.8, 136.2, 136.2, 133.1, 130.2, 129.6, 129.1, 128.8, 127.1 (q, *J* = 279.6 Hz), 125.8, 120.6, 120.5, 72.6 (q, *J* = 2.9 Hz), 62.47, 49.4–48.7 (m), 46.8–45.6 (m), 44.1 (q, *J* = 26.3 Hz), 43.5–43.1 (m), 27.5, 16.7 (q, *J* = 2.3 Hz). **HRMS** calcd. for $C_{25}H_{26}F_3N_5O_2$ $[M+H]^+$ 486.2111, found 486.2099 (−2.65 ppm).

The purity of compound **10** (>95%) was assessed by liquid chromatography–mass spectrometry that was performed using method A on a 1260 Infinity II LC system (Agilent Technologies, Santa Clara, CA, USA), equipped with a quaternary pump, a wavelength detector and coupled to the mass spectrometer (Expression CMS; Advion Inc., Ithaca, NY, USA).

Method A: A C18 column was used (Waters xBridge BEH; 4.6 mm × 150 mm, 3.5 µm) at 40 °C, while the flow rate of the mobile phase was 1.5 mL/min, the injection volume was 10 µL. The following elution gradient was used: 0→1 min, 25% B; 1→6 min, 25%→98% B; 6→6.5 min, 98% B; 6.5→7 min, 98%→25% B; 7→10 min, 25% B and the products were detected at 254 nm. Solvent A composition: 1% CH_3CN and 0.1% HCOOH in double-distilled H_2O Solvent B composition: CH_3CN. **HPLC**: t_r = 4.92 (99.4% at 254 nm).

[15] Tanaka, M.; Hirata, Y.; Sawaguchi, T.; Kurosawa, S. *Arkivoc* **2018**, 330–343.

HPLC trace of compound 10.
UV detector (top), MS detector (bottom)

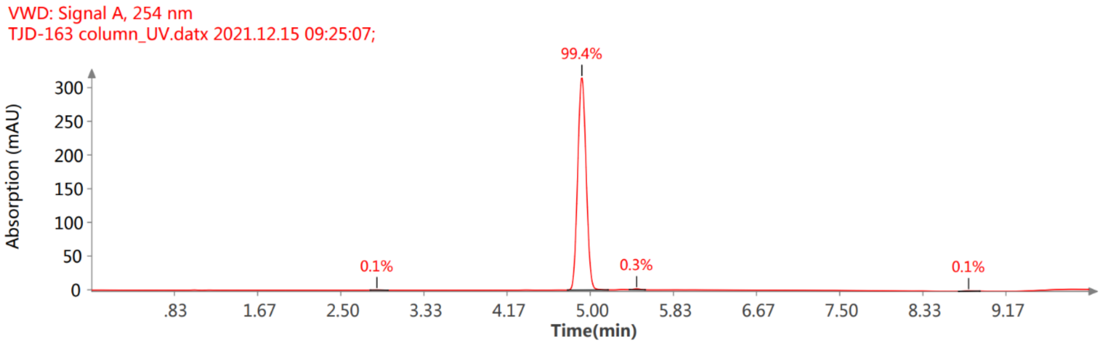

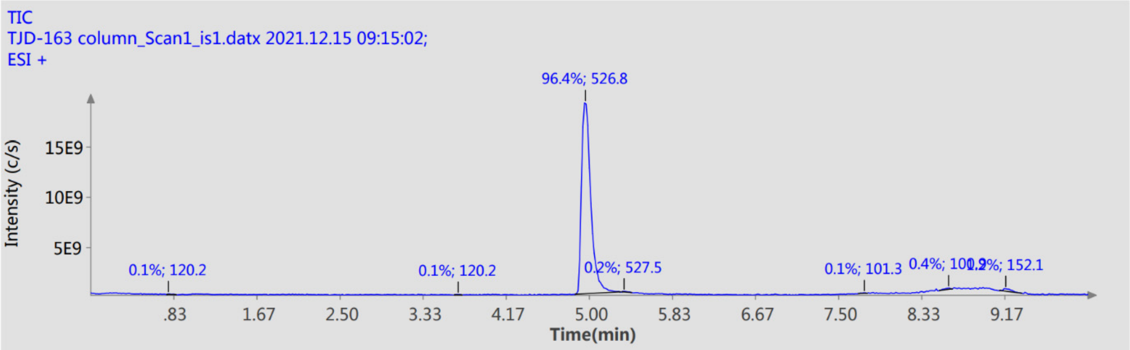

Control experiments regarding detrifluoromethylation

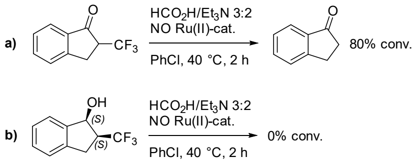

a) Heating **1a** (10 mg) in a mixture of HCO_2H/Et_3N 3:2 (0.1 mL) and PhCl (0.2 mL) (without any catalyst) at 40 °C for 2 h resulted in 80% conversion to 1-indanone **3a**. Diagnostic NMR signals: ^{1}H NMR (400 MHz, Chloroform-*d*) δ 7.82 (d, *J* = 7.4 Hz, 1H, **1a**), 7.77 (d, *J* = 7.7 Hz, 1H, **3a**).

b) Heating **2a** (10 mg) in a mixture of HCO_2H/Et_3N 3:2 (0.1 mL) and PhCl (0.2 mL) (without any catalyst) at 40 °C for 2 h resulted in no conversion to 1-indanone **3a** or 1-indanol **4a**. Diagnostic NMR signals : ^{1}H NMR (400 MHz, Chloroform-*d*) δ 7.82 (d, *J* = 7.4 Hz, 1H, **1a**), 7.77 (d, *J* = 7.7 Hz, 1H, **3a**), 5.36 (d, *J* = 5.7 Hz, 1H, **2a**), 5.28–5.23 (m, 1H, **4a**).

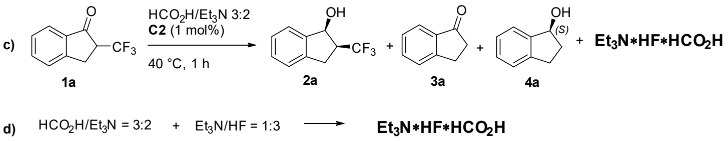

c) Two signals around −130 ppm were observed in ^{19}F NMR spectra of the crude reaction mixtures when significant de-trifluoromethylation occurred (for example Table 1, entry 6): One reproducibly at −130ppm and another broader one at variable shifts. These correspond to Et_3N– HF–HCO_2H complex.

d) The same signals were observed in the ^{19}F NMR spectrum of a mixture of HCO_2H/Et_3N 3:2 and commercially available triethylammonium trihydrofluoride (Sigma-Aldrich).

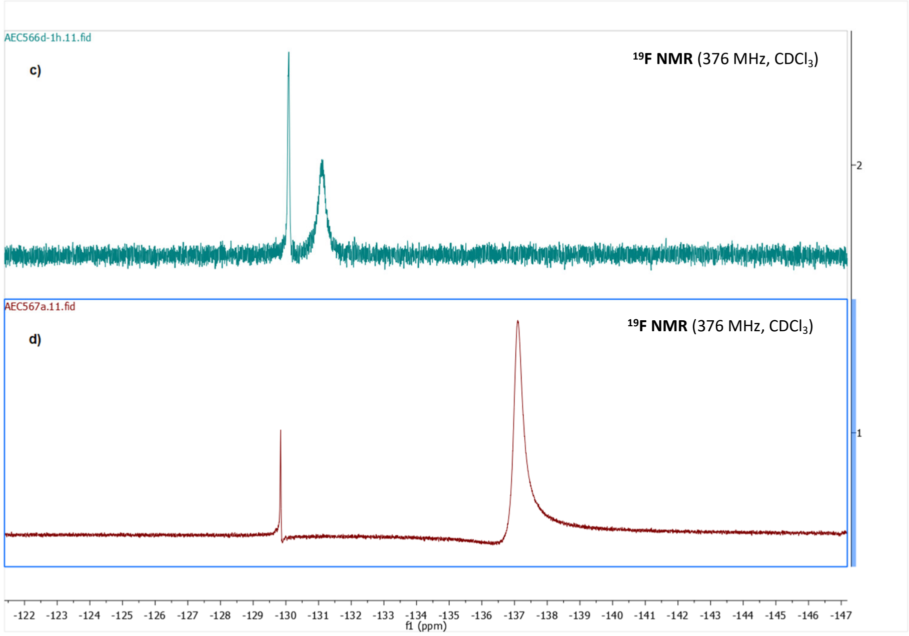

Based on the observed HF elimination, a possible mechanism for the loss of CF_3 group can be speculated:

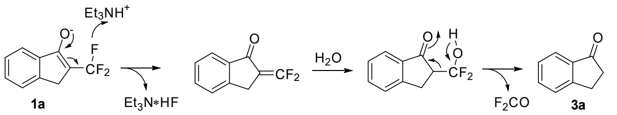

A similar mode of detrifluoromethylation via fluoride elimination followed by hydrolysis/solvolysis of difluoroenone has been documented for α-trifluoromethyl ketones orchestrated by inorganic or organic bases.[16]

[16] a) Itoh, Y.; Mikami, K. *Org. Lett.* **2005**, *7*, 4883–4885. b) Kizirian, J.-C.; Aiguabella, N.; Pesquer, A.; Fustero, S.; Bello, P.; Verdaguer, X.; Riera, A. *Org. Lett.* **2010**, *12*, 5620–5623.

Determination of stereomeric ratios

A $NaBH_4$ reduction product was used as a standard, and for the DKR-ATH products, the stereomeric ratios were determined for crude, non-recrystallized samples.

Diastereomeric ratio was determined by non-decoupled ^{19}F NMR (376 MHz, $CDCl_3$), or by ^{1}H NMR (400 MHz, $CDCl_3$).

Enantiomeric ratio was determined by HPLC or GC analysis using columns with chiral stationary phase as specified above.

2a. 2-Trifluoromethyl-1-indanol.

- **^{19}F NMR. Top:** $NaBH_4$ reduction, d.r. = 97:3. **Bottom:** DKR-ATH, d.r. > 99.9:0.1 signal/noise = 3000

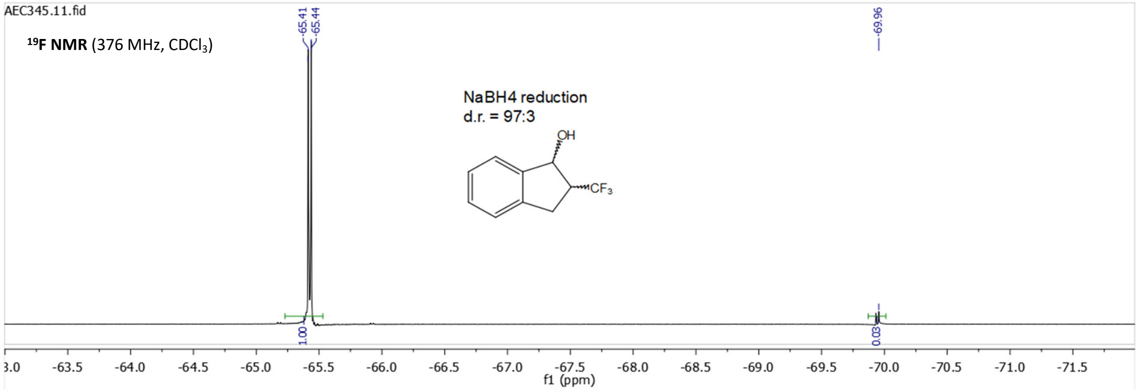

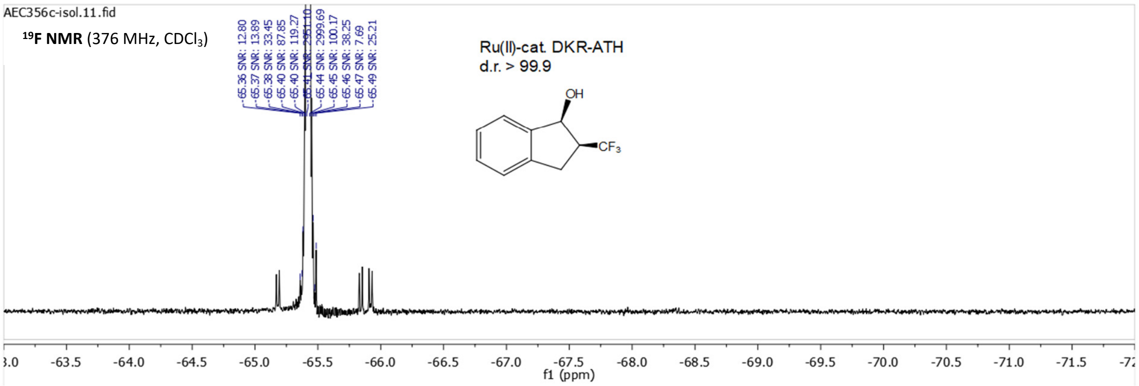

- **Chiral HPLC. Top:** $NaBH_4$ reduction, **Bottom:** DKR-ATH using (*S*,*S*)-**C2**, >99% ee

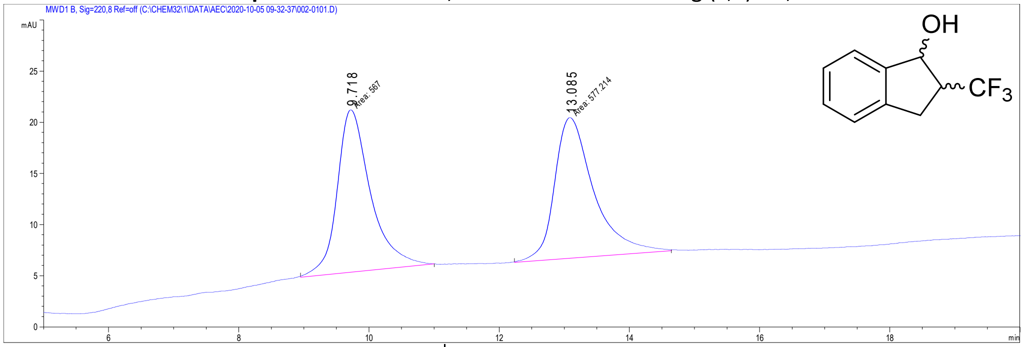

#	Time	Area	Height	Width	Area%	Symmetry
(*S*,*S*)-**2a**	9.718	567	15.9	0.595	49.554	0.663
(*R*,*R*)-**2a**	13.085	577.2	13.8	0.6989	50.446	0.641

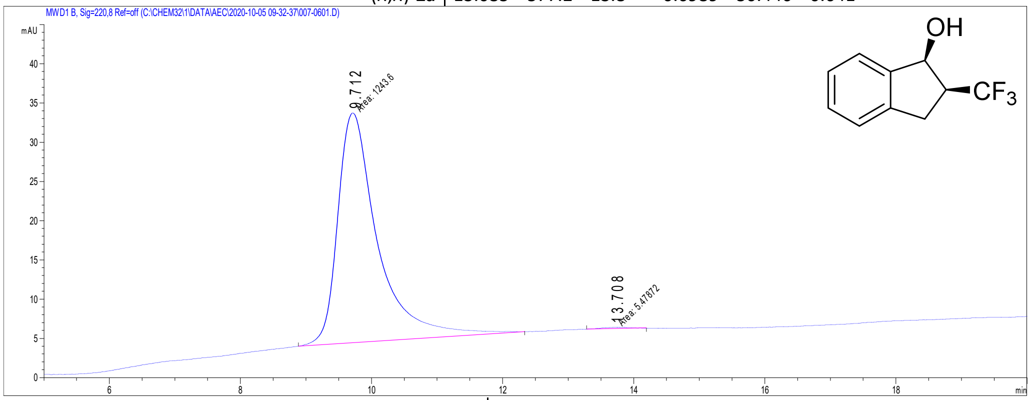

#	Time	Area	Height	Width	Area%	Symmetry
(*S*,*S*)-**2a**	9.712	1243.6	29.3	0.707	99.561	0.583
impurity or (*R*,*R*)-**2a**	13.708	5.5	0.18	0.5012	0.439	1.022

2b. 2-Trifluoromethyl-4,6-dimethyl-1-indanol.

- **^{19}F NMR. Top:** $NaBH_4$ reduction, d.r. = 92:8. **Bottom:** DKR-ATH, d.r. > 99.9:0.1 signal/noise = 3000

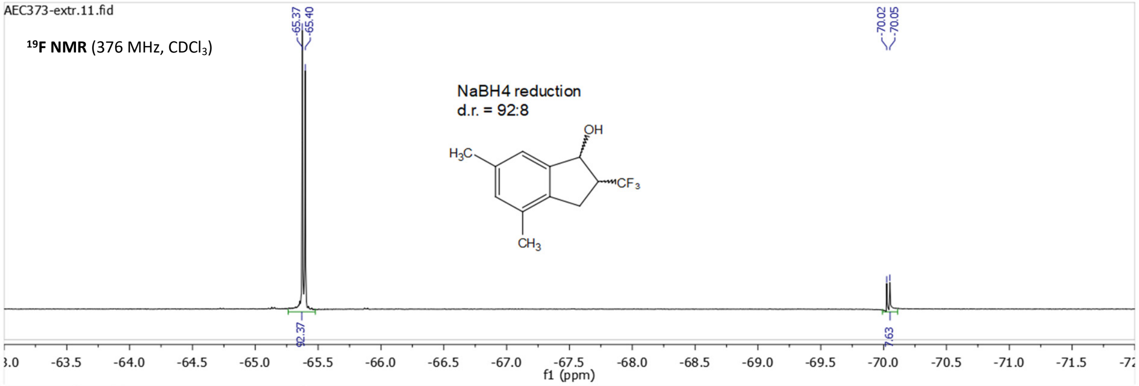

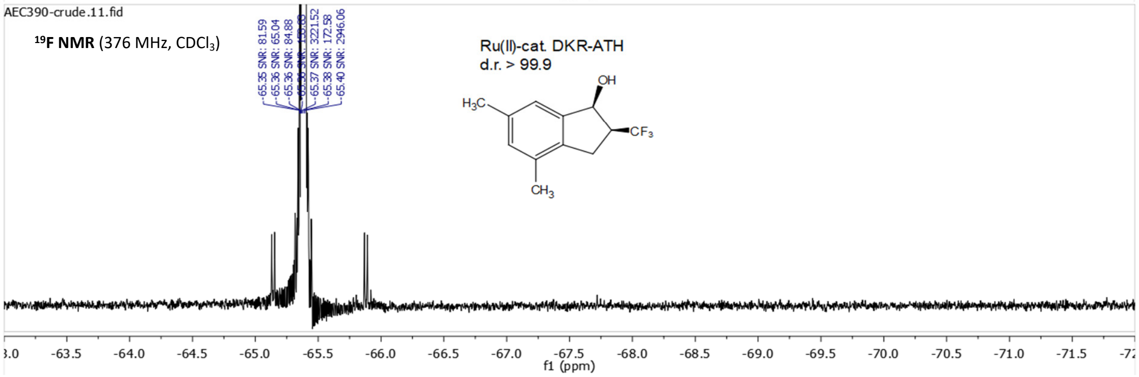

- **Chiral HPLC. Top:** $NaBH_4$ reduction, **Bottom:** DKR-ATH using (*S*,*S*)-**C2**, >99% ee

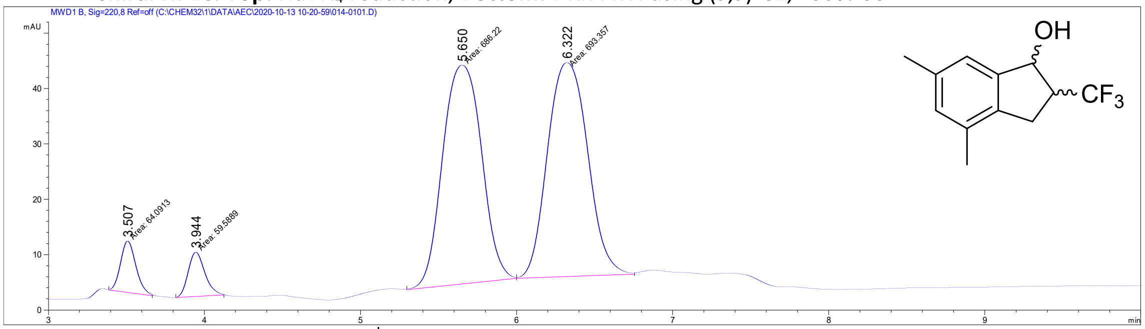

#	Time	Area	Height	Width	Area%	Symmetry
trans-**2b**	3.507	64.1	9.3	0.1145	4.263	0.858
ent-trans-**2b**	3.944	59.6	8.1	0.1229	3.964	0.805
(*S*,*S*)-**2b**	5.65	686.2	39.6	0.2889	45.649	0.971
(*R*,*R*)-**2b**	6.322	693.4	38.7	0.2986	46.124	0.912

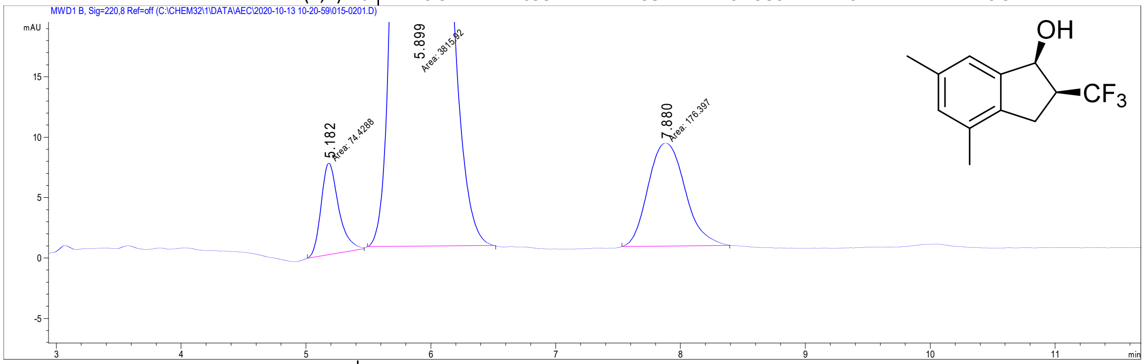

#	Time	Area	Height	Width	Area%	Symmetry
impurity	5.182	74.4	7.6	0.1627	1.83	0.709
(*S*,*S*)-**2b**	5.899	3815.9	203.5	0.3126	93.832	0.865
impurity	7.88	176.4	8.6	0.3437	4.338	0.887

2c. 2-Trifluoromethyl-4,5,6,7-tetramethyl-1-indanol.

- **^{19}F NMR: Top:** $NaBH_4$ reduction, d.r. = 95:5. **Bottom:** DKR-ATH, d.r. > 99.9:0.1 signal/noise = 11600

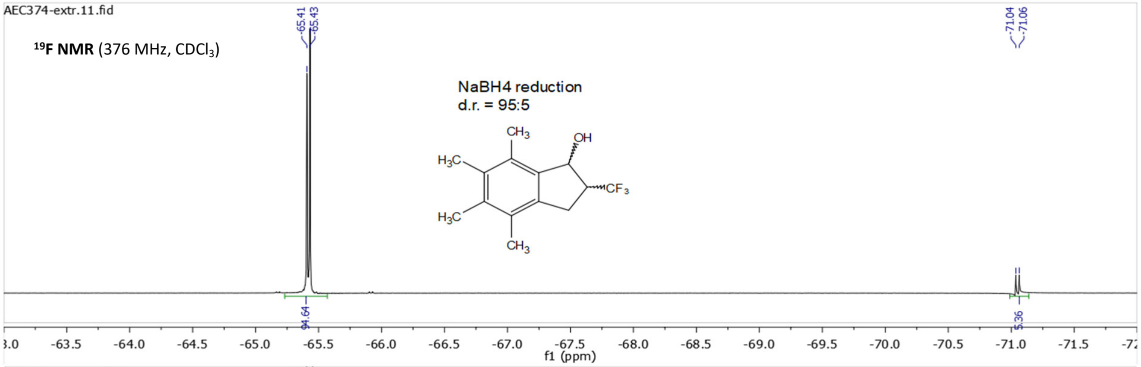

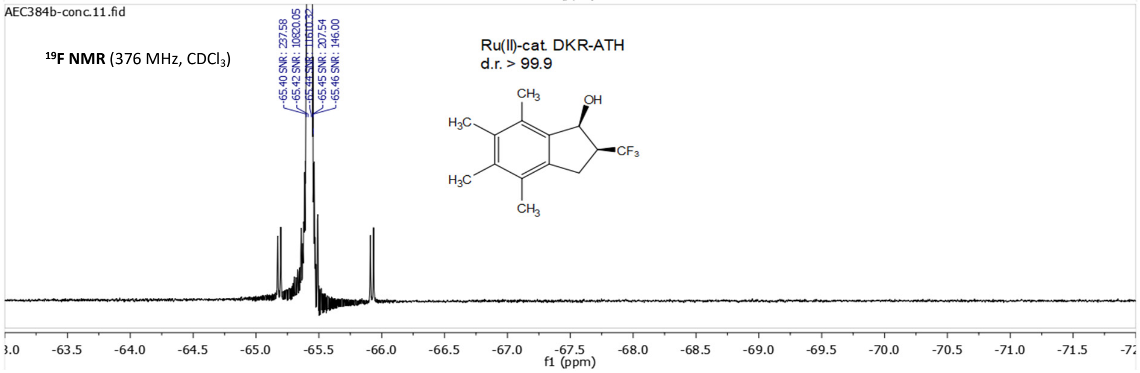

- **chiral GC, Top:** $NaBH_4$ reduction **Bottom:** DKR-ATH using (*S*,*S*)-**C2**, >99% ee

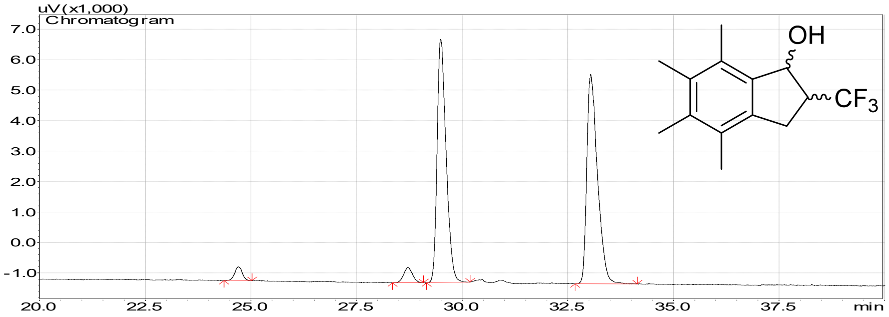

Peak	tR (min)	Area	Height	Area %
trans-**2c**	24.700	5742.1	451.9	2.2731
ent-trans-**2c**	28.711	7302.6	502.6	2.8909
(*R*,*R*)-**2c**	29.487	118354.0	7975.6	46.8529
(*S*,*S*)-**2c**	33.039	121209.2	6861.7	47.9831

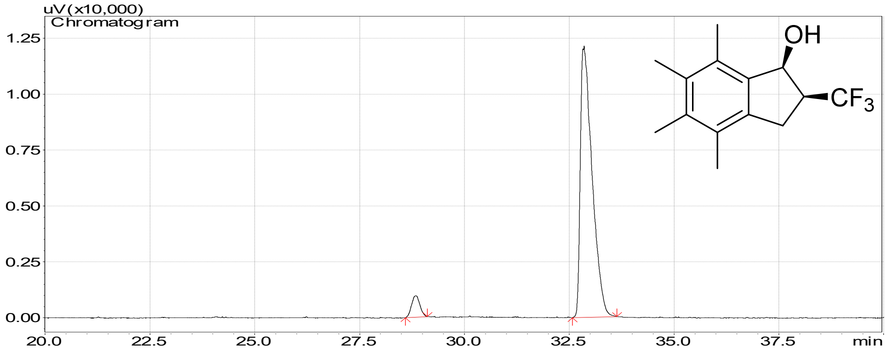

Peak	tR (min)	Area	Height	Area %
impurity	28.839	13361.0	961.2	5.3448
(*S*,*S*)-**2c**	32.855	236622.3	12125.9	94.6552

2d. 4-Bromo-2-trifluoromethyl-1-indanol.

- **^{19}F NMR. Top:** $NaBH_4$ reduction, d.r. = 90:10. **Bottom:** DKR-ATH, d.r. > 99.9:0.1 signal/noise = 5000

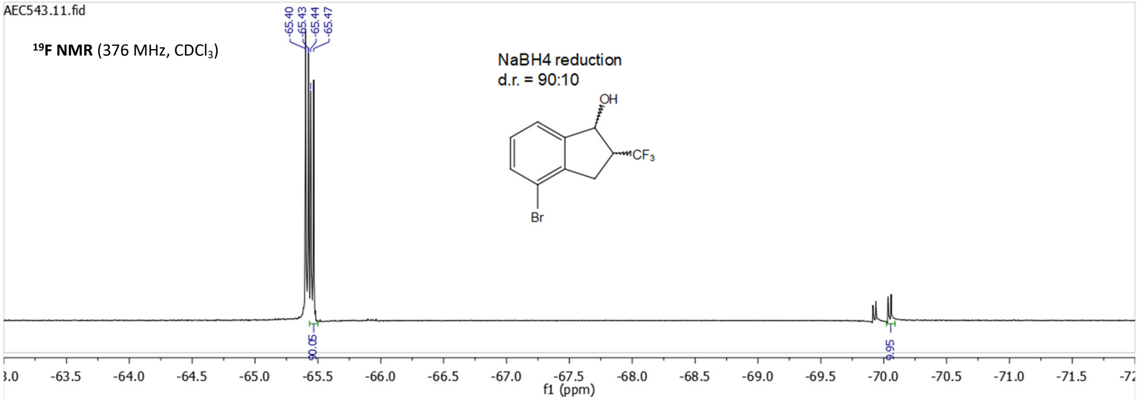

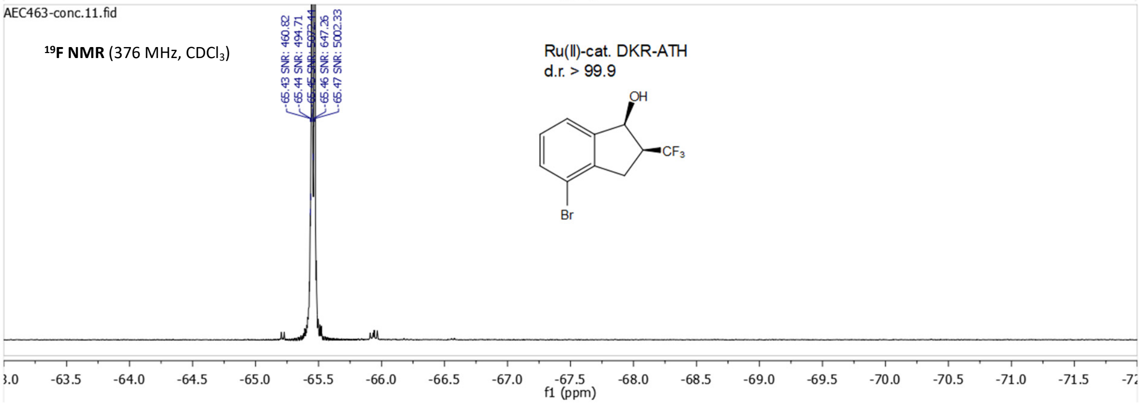

- **chiral GC, Top:** $NaBH_4$ reduction **Bottom:** DKR-ATH using (*S*,*S*)-**C2** (left) or (*R*,*R*)-**C2** (right), >99% ee

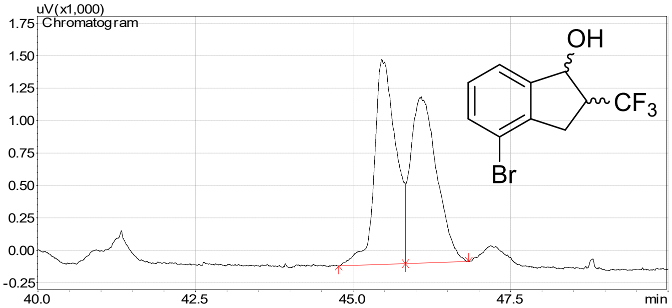

Peak	tR (min)	Area	Height	Area %
(*R*,*R*)-**2d**	45.453	37742.8	1579.4	50.4032
(*S*,*S*)-**2d**	46.082	37138.9	1281.2	49.5968

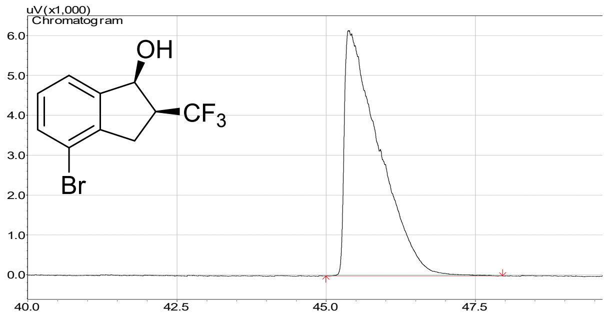

Peak	tR (min)	Area	Height	Area %
(*S*,*S*)-**2d**	45.377	248725.2	6154.4	100.0000

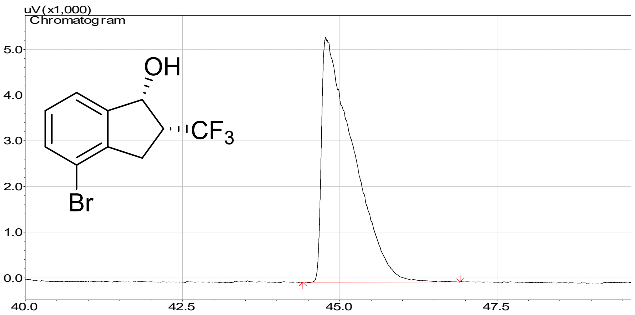

Peak	tR (min)	Area	Height	Area %
(*R*,*R*)-**2d**	44.776	187600.6	5354.3	100.0000

2e. 2-Trifluoromethyl-5-fluoro-1-indanol.

Top: $NaBH_4$ reduction, d.r. = 94:6. **Bottom:** DKR-ATH, d.r. > 99.9:0.1 signal/noise = 3200

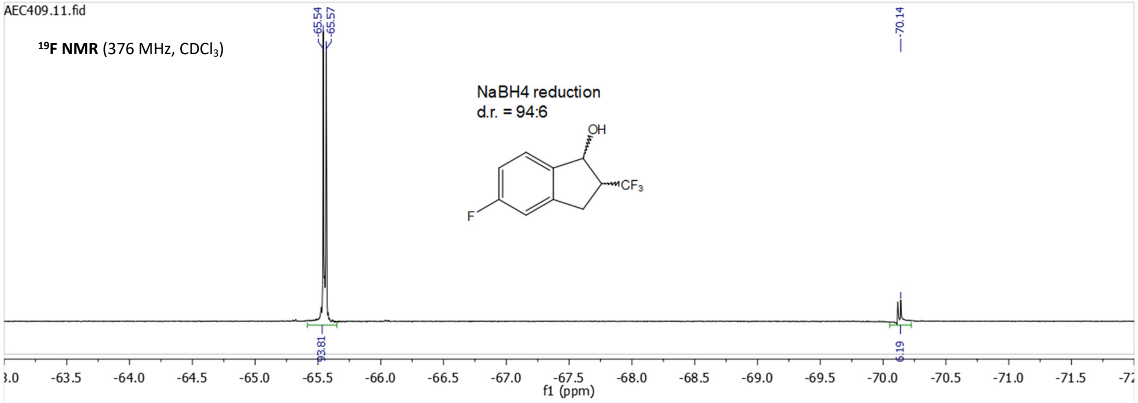

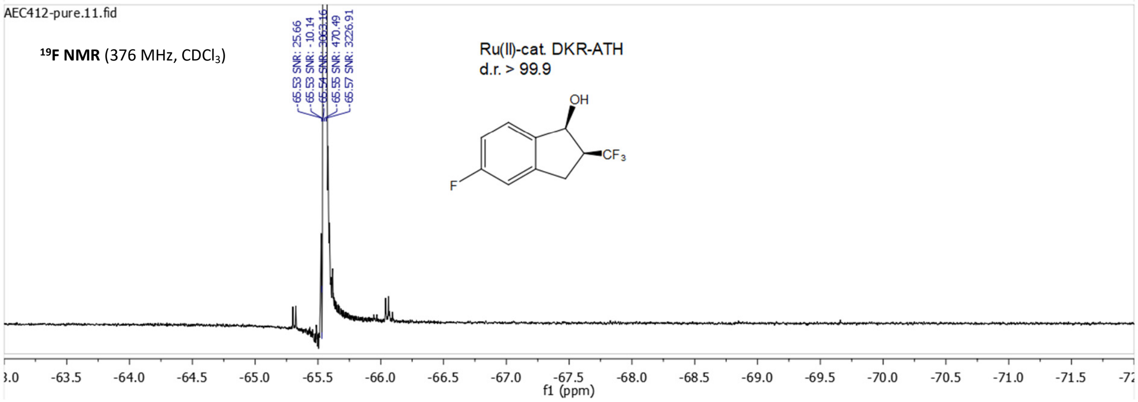

- **chiral GC, Top:** $NaBH_4$ reduction; **Bottom:** DKR-ATH using (*S*,*S*)-**C2**, 99.8% ee

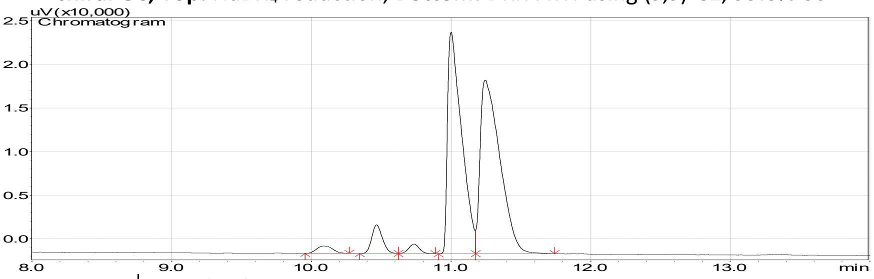

Peak	tR (min)	Area	Height	Area %
not assigned	10.087	6529.9	828.1	1.6175
not assigned	10.467	17557.5	3299.3	4.3491
(*R*,*R*)-**2e**	10.734	5747.9	1090.0	1.4238
(*S*,*S*)-**2e**	11.001	178223.7	25377.1	44.1471
not assigned	11.246	195645.5	19914.6	48.4626

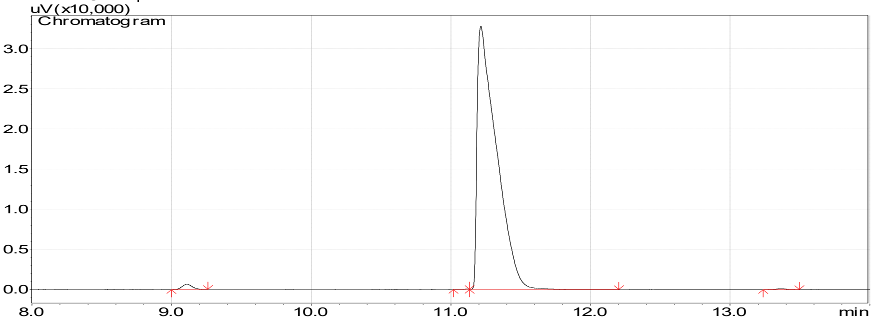

Peak	tR (min)	Area	Height	Area %
impurity	9.110	3440.3	656.8	1.0723
(*R*,*R*)-**2e**	11.117	206.2	73.2	0.0643
(*S*,*S*)-**2e**	11.214	316359.0	32771.3	98.6020
impurity	13.364	838.9	120.4	0.2615

2f. 2,6-Bis-trifluoromethyl-1-indanol.

- **^{19}F NMR. Top:** $NaBH_4$ reduction, d.r. = 92:8. **Bottom:** DKR-ATH, d.r. > 99.9:0.1 signal/noise = 4500

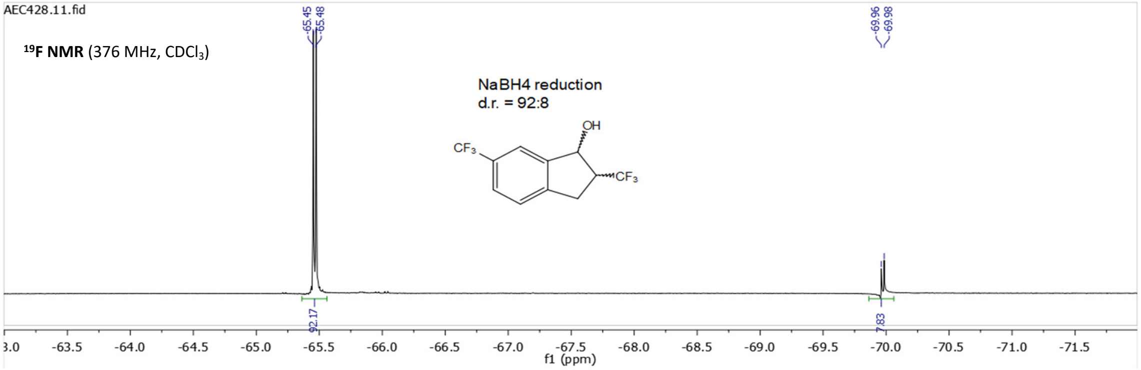

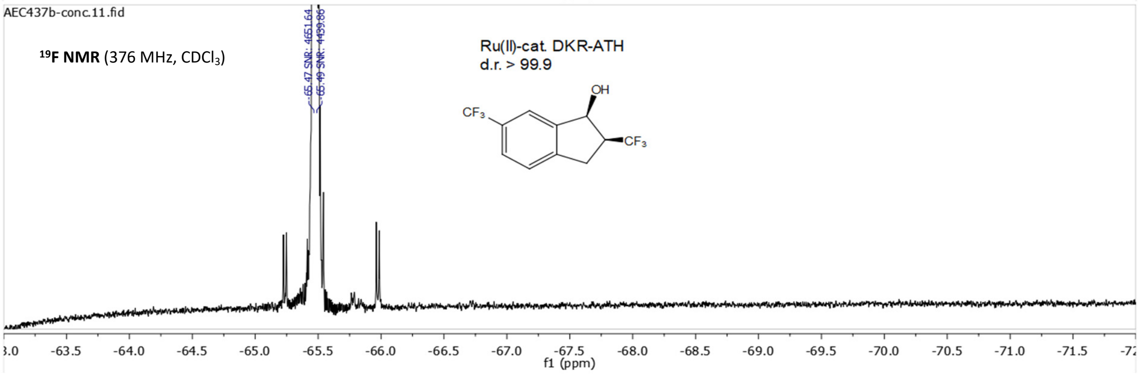

- **Chiral GC. Top:** $NaBH_4$ reduction, **Bottom:** DKR-ATH using (*S*,*S*)-**C2**, >99% ee

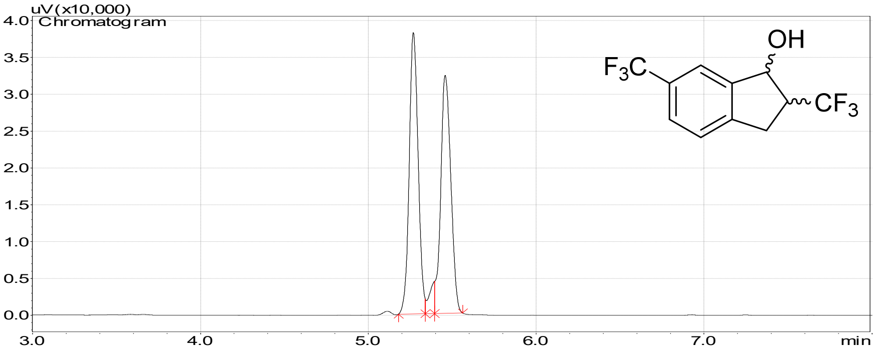

Peak	tR (min)	Area	Height	Area %
(*R*,*R*)-**2f** + *trans*-**2f**	5.268	141376.7	38122.3	49.2906
ent-trans-**2f**	5.391	9896.8	4208.7	3.4505
(*S*,*S*)-**2f**	5.458	135549.3	32262.0	47.2589

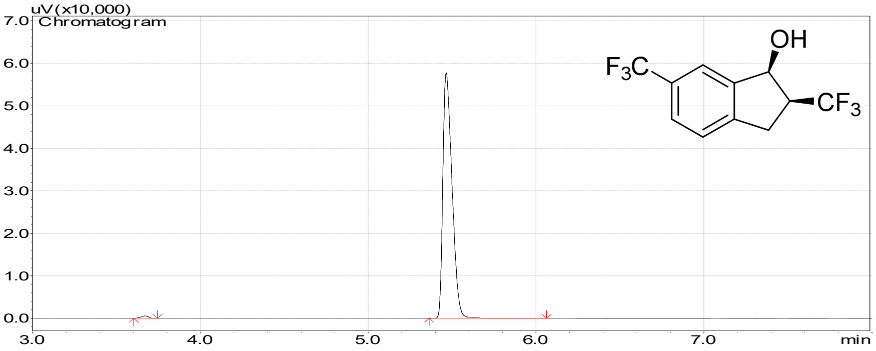

Peak	tR (min)	Area	Height	Area %
impurity	3.671	1784.6	565.3	0.8021
(*S*,*S*)-**2f**	5.464	220708.7	57622.5	99.1979

2g. 2-Trifluoromethyl-6-methoxy-1-indanol

- **19F NMR. Top:** $NaBH_4$ reduction, d.r. = 92:8. **Bottom:** DKR-ATH, d.r. > 99.9:0.1 signal/noise = 9000

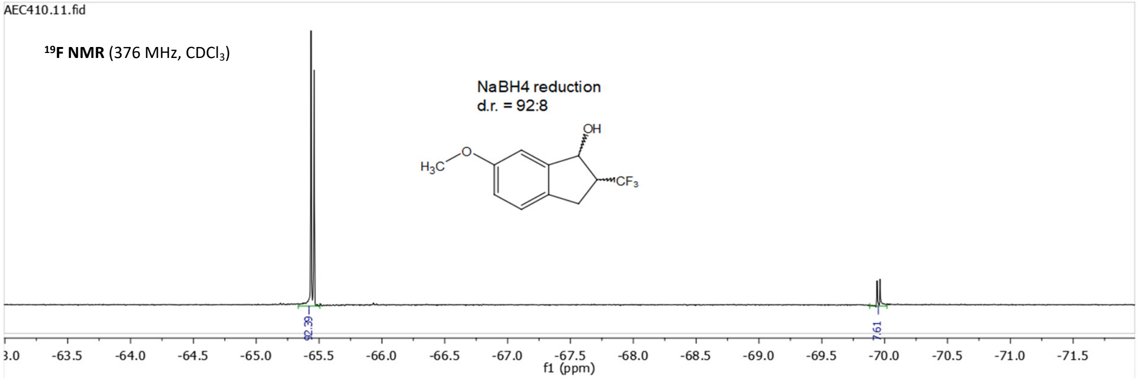

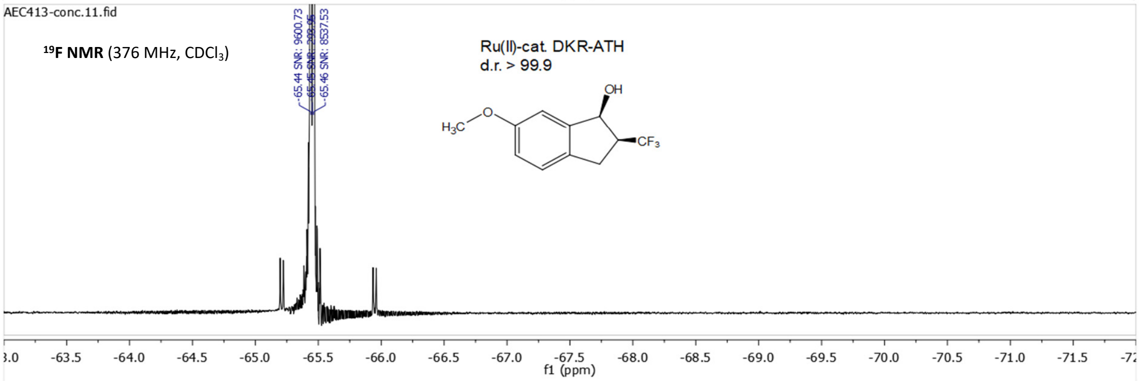

- **Chiral HPLC. Top:** $NaBH_4$ reduction, **Bottom:** DKR-ATH using (*S*,*S*)-**C2**, >99% ee

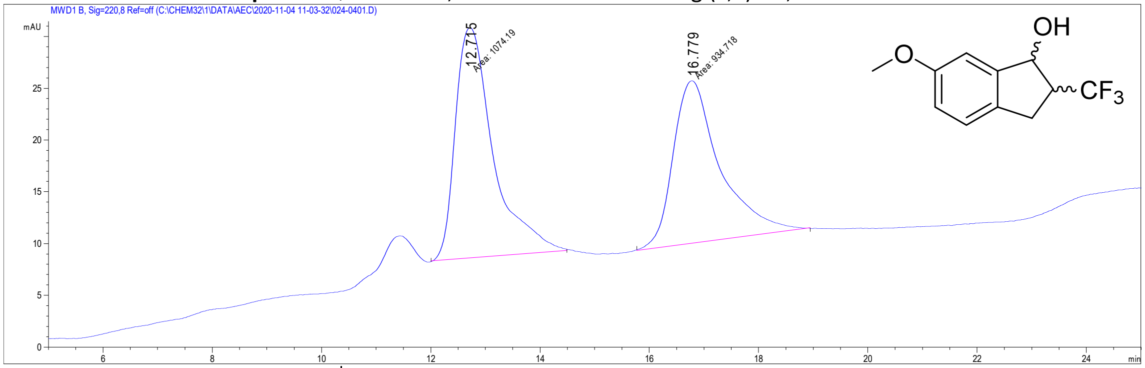

#	Time	Area	Height	Width	Area%	Symmetry
(*S*,*S*)-**2g**	12.715	1074.2	22.3	0.8045	53.471	0.606
(*R*,*R*)-**2g**	16.779	934.7	15.7	0.9911	46.529	0.662

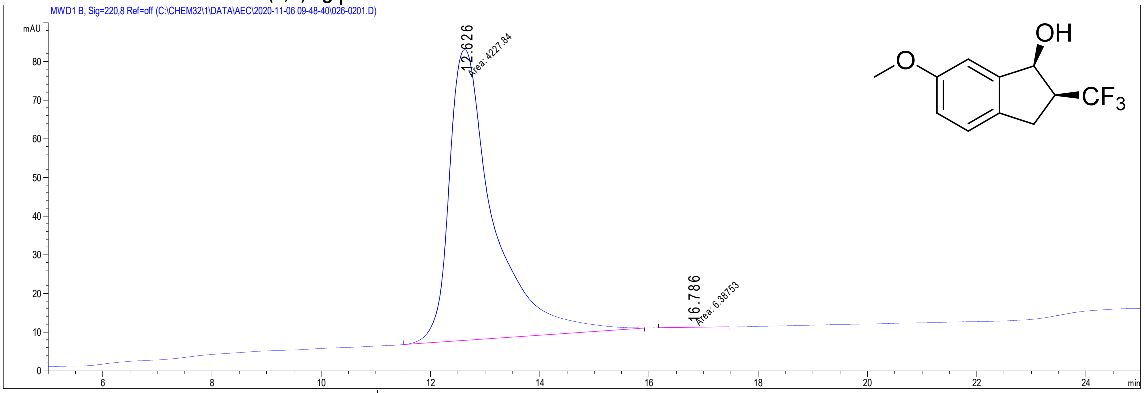

#	Time	Area	Height	Width	Area%	Symmetry
(*S*,*S*)-**2g**	12.626	4227.8	75.2	0.9365	99.849	0.528
possible (*R*,*R*)-**2g**	16.786	6.4	1.60E-01	0.669	0.151	1.024

2h. 7-Acetamido-6-methoxy-1-indanol.

- **19F NMR. Top:** $NaBH_4$ reduction, d.r. = 93:7. **Bottom:** DKR-ATH, d.r. > 99.9:0.1 signal/noise = 580

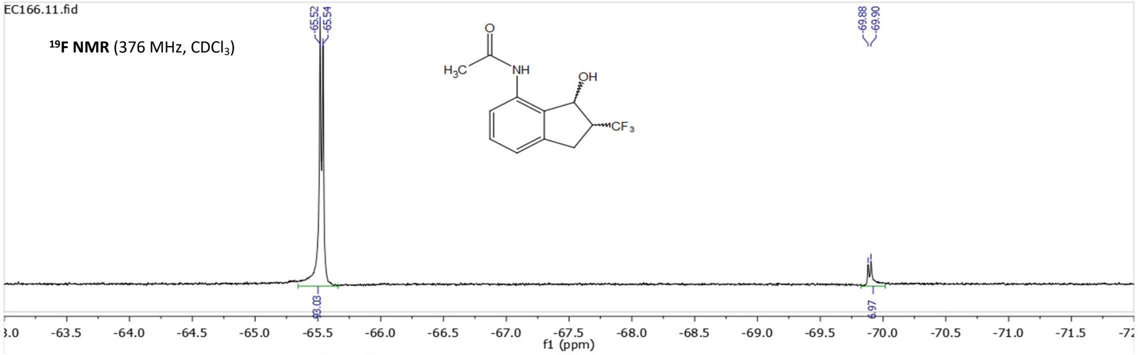

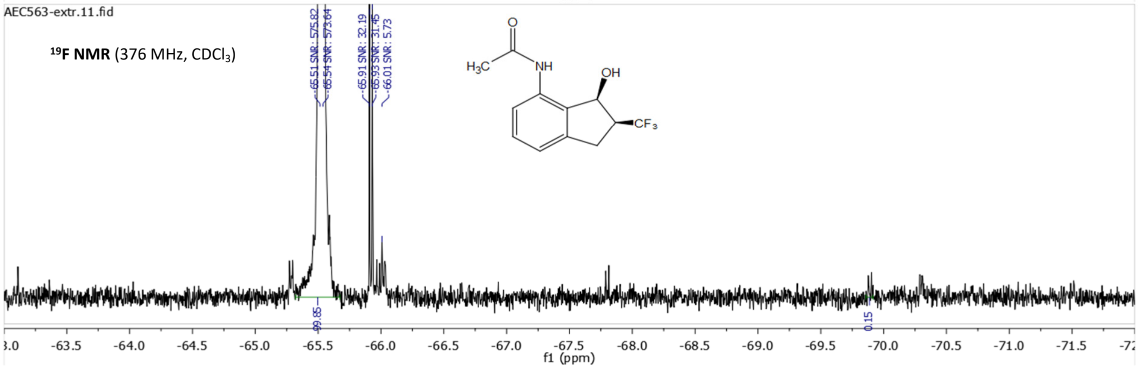

- **Chiral GC. Top:** $NaBH_4$ reduction, **Bottom:** DKR-ATH using (*S*,*S*)-**C2**, >99% ee

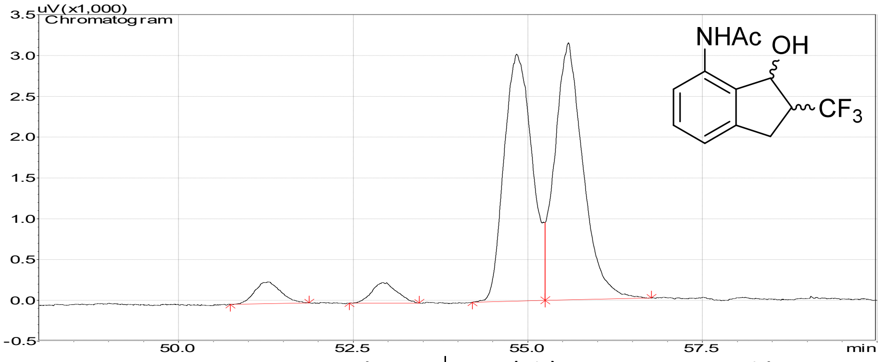

Peak	tR (min)	Area	Height	Area %
trans-**2h**	51.295	7023.4	268.0	3.7502
ent-trans-**2h**	52.921	6373.4	253.0	3.4031
(*R*,*R*)-**2h**	54.841	85125.2	3027.1	45.4531
(*S*,*S*)-**2h**	55.581	88759.1	3151.3	47.3935

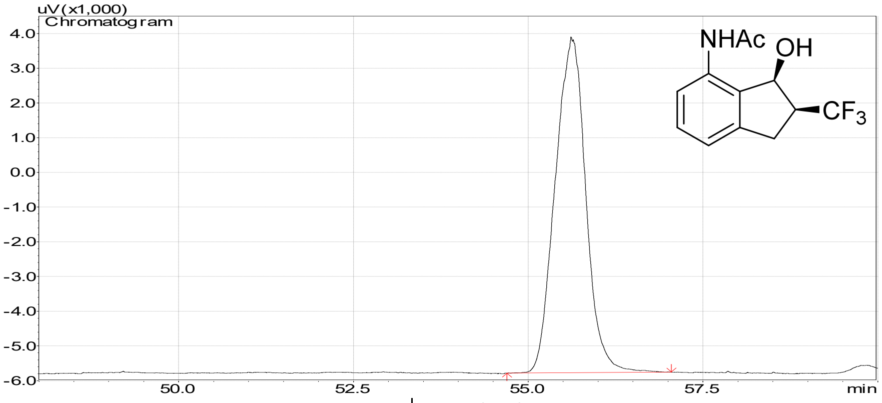

Peak	tR (min)	Area	Height	Area %
(*S*,*S*)-**2h**	55.613	294498.4	9668.5	100.0000

2i. 2-trifluoromethyl-1-tetralol.

Top: $NaBH_4$ reduction, d.r. = 91:9. **Bottom:** DKR-ATH, d.r. > 99.9:0.1 signal/noise = 2900

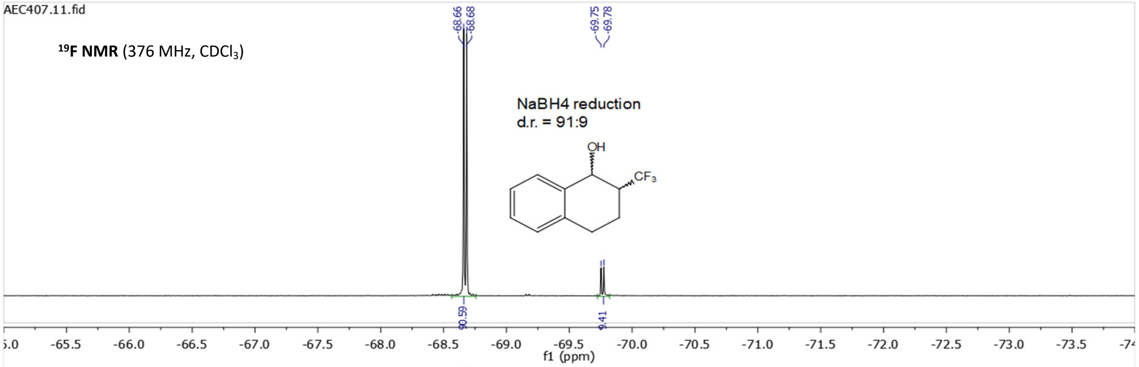

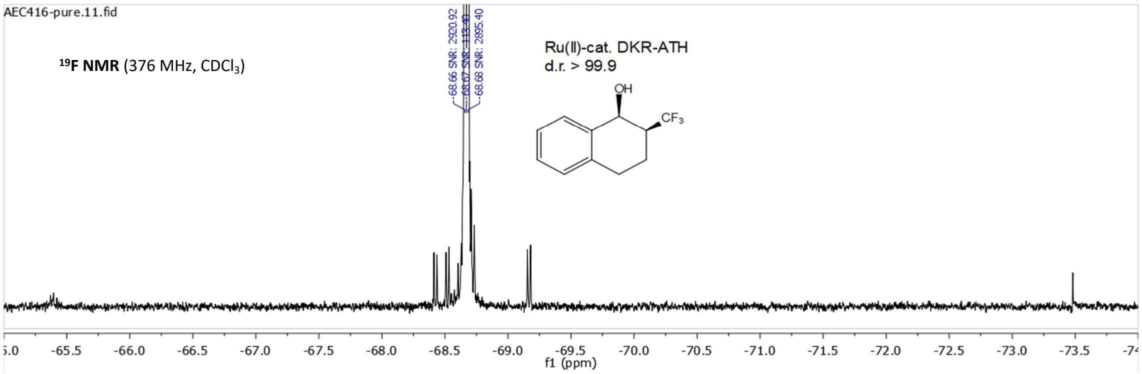

- **Chiral HPLC. Top:** $NaBH_4$ reduction, **Bottom:** DKR-ATH using (*S*,*S*)-**C2**, >99% ee

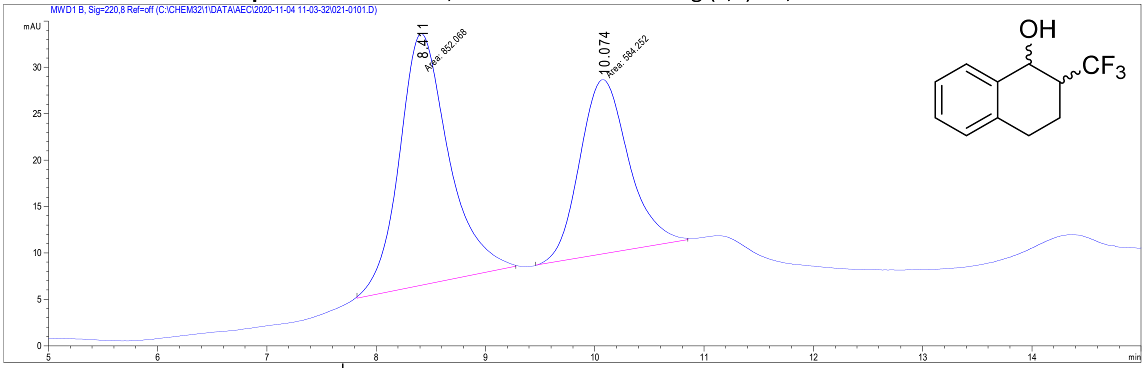

#	Time	Area	Height	Width	Area%	Symmetry
(*S*,*S*)-**2i**	8.411	852.1	27.2	0.5227	59.323	0.798
(*R*,*R*)-**2i**	10.074	584.3	1.88E+01	0.5174	40.677	0.884

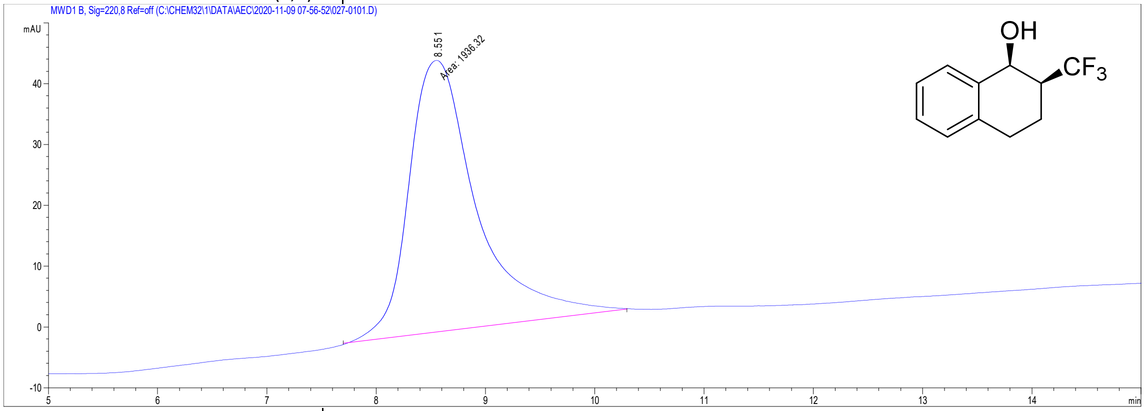

#	Time	Area	Height	Width	Area%	Symmetry
1	8.551	1936.3	44.7	0.7219	100	0.663

2j. 6-methoxy-2-trifluoromethyl-1-tetralol.

- **^{19}F NMR. Top:** $NaBH_4$ reduction, d.r. = 81:19. **Bottom:** DKR-ATH, d.r. > 99.9:0.1 signal/noise = 2600

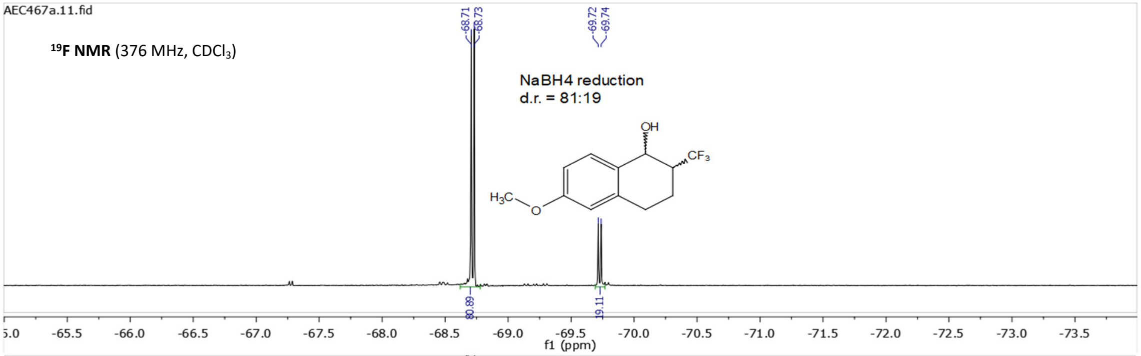

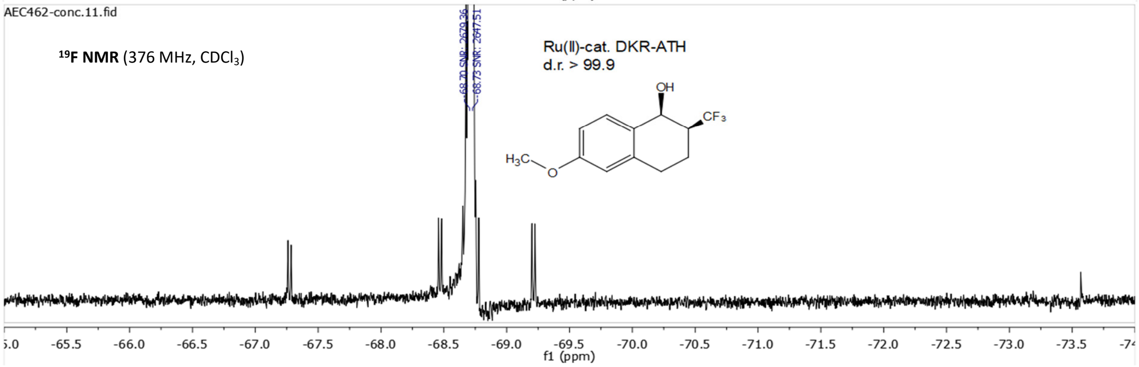

- **Chiral GC. Top:** $NaBH_4$ reduction, **Bottom:** DKR-ATH using (*S*,*S*)-**C2**, >99% ee

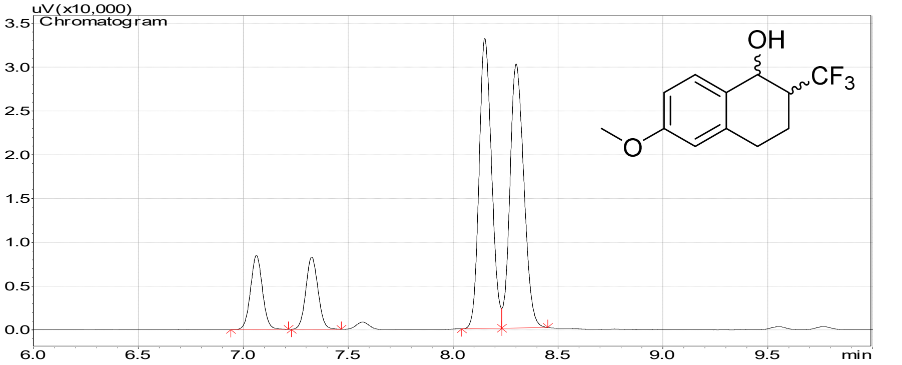

Peak	tR (min)	Area	Height	Area %
trans-**2j**	7.062	33296.3	8436.4	9.7288
ent-trans-**2j**	7.326	33232.5	8224.8	9.7102
(*R*,*R*)-**2j**	8.151	137045.8	33059.7	40.0434
(*S*,*S*)-**2j**	8.301	138668.7	30099.2	40.5176

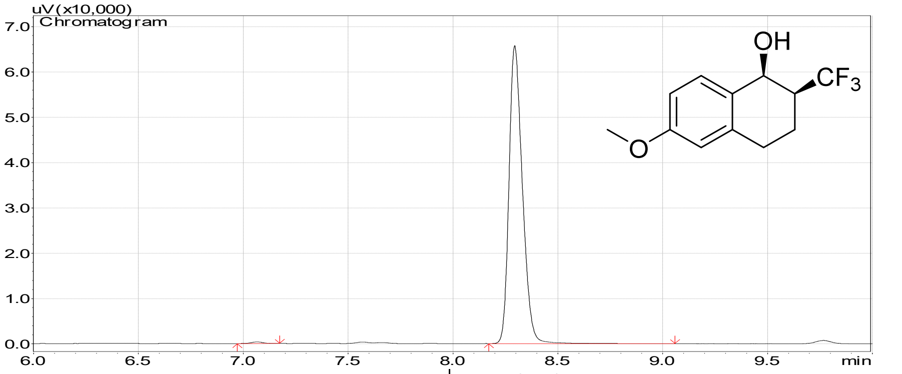

Peak	tR (min)	Area	Height	Area %
possible *trans*-**2j**	7.066	1378.0	350.9	0.4695
(*S*,*S*)-**2j**	8.295	292103.2	65596.9	99.5305

2k. 7-methoxy-2-trifluoromethyl-1-tetralol.

- **^{19}F NMR. Top:** NaBH$_4$ reduction, d.r. = 91:9. **Bottom:** DKR-ATH, d.r. > 99.9:0.1 signal/noise = 11000

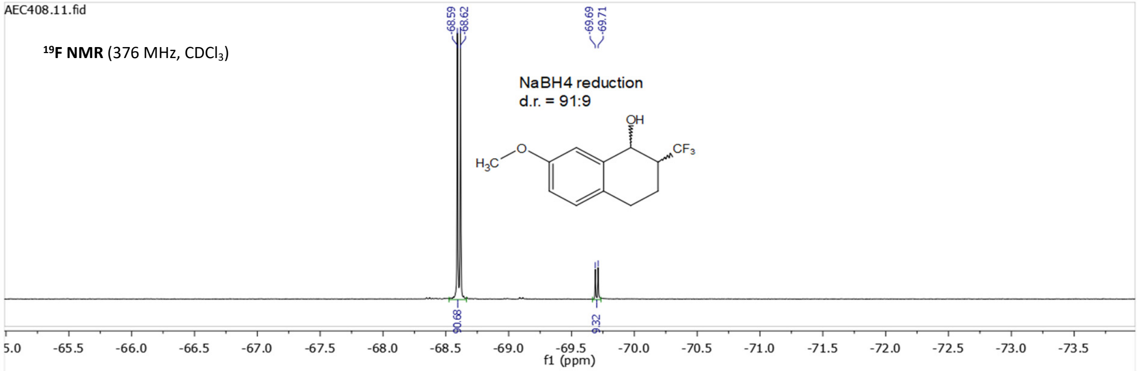

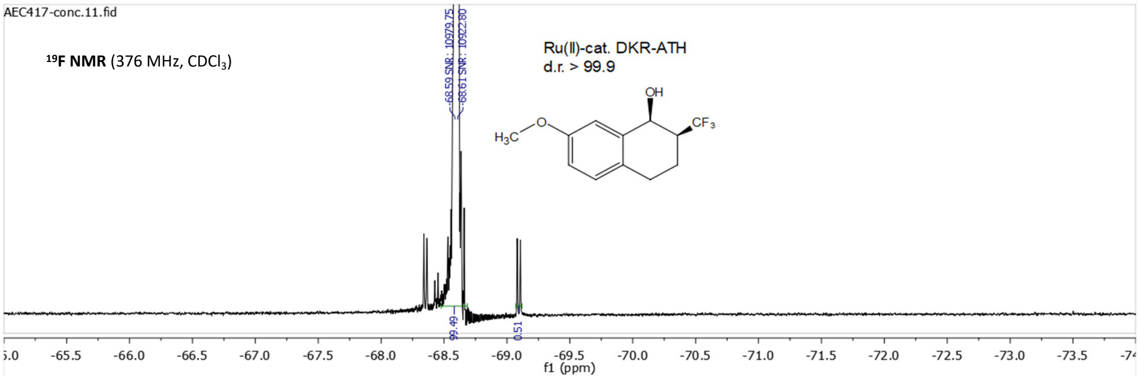

- **Chiral HPLC. Top:** NaBH$_4$ reduction, **Bottom:** DKR-ATH using (*S*,*S*)-**C2**, >99% ee

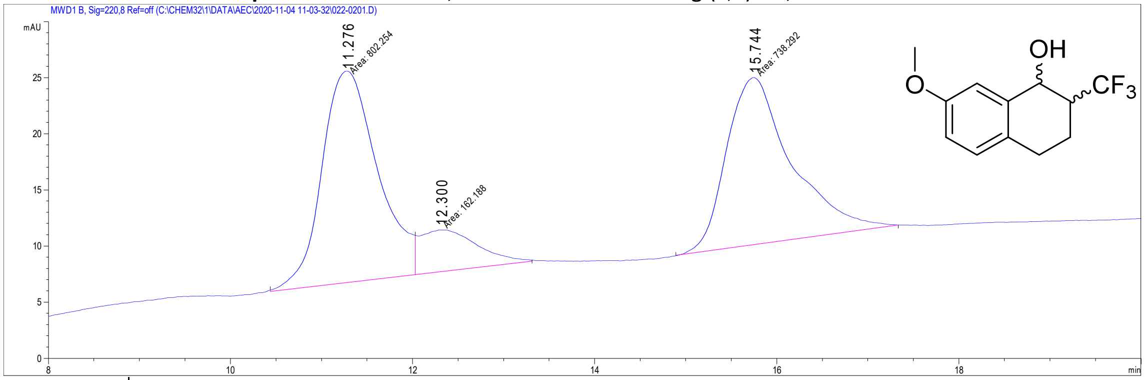

#	Time	Area	Height	Width	Area%	Symmetry
(*S*,*S*)-**2k**	11.276	802.3	18.9	0.7091	47.116	0.798
	12.3	162.2	3.70	0.7213	9.525	0.534
(*R*,*R*)-**2k**	15.744	738.3	14.9	0.8252	43.359	0.69

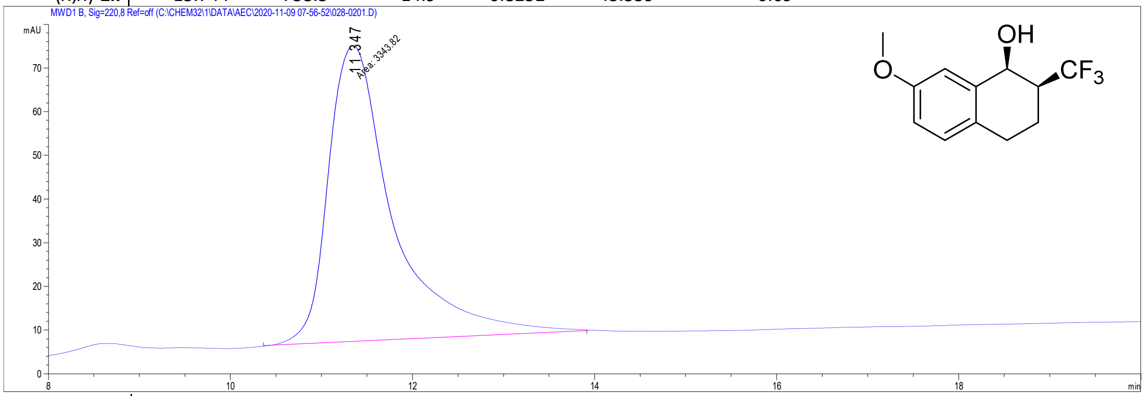

#	Time	Area	Height	Width	Area%	Symmetry
1	11.347	3343.8	67.8	0.822	100	0.589

2l. 3-trifluoromethyl-4-chromanol.

- **^{19}F NMR. Top:** NaBH$_4$ reduction, d.r. = 96:4. **Bottom:** DKR-ATH, d.r. > 99.9:0.1 signal/noise = 14000

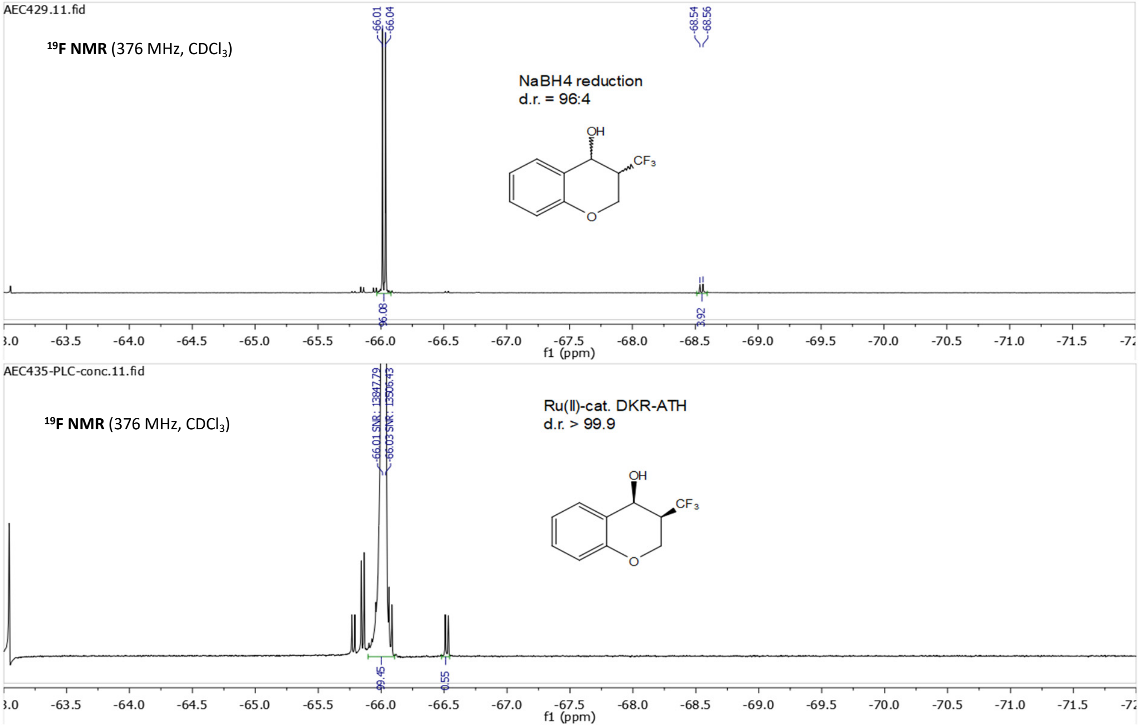

- **Chiral GC. Top:** NaBH$_4$ reduction, **Bottom:** DKR-ATH using (*S*,*S*)-**C2**, >99% ee

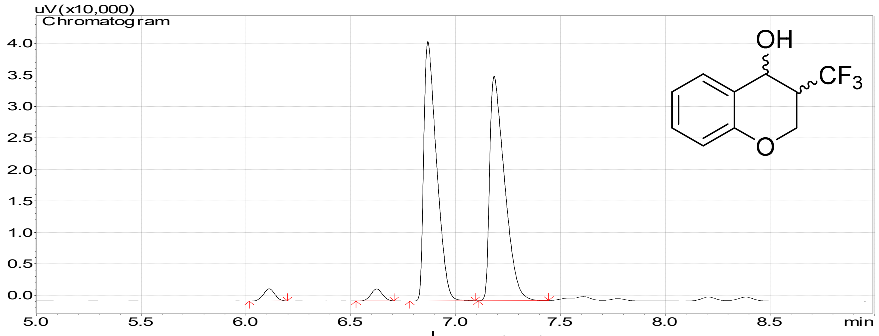

Peak	tR (min)	Area	Height	Area %
trans-**2l**	6.111	7411.5	1951.1	2.0266
ent-trans-**2l**	6.624	7252.3	1914.0	1.9831
(*R*,*R*)-**2l**	6.868	174413.0	41151.5	47.6914
(*S*,*S*)-**2l**	7.184	176635.2	35568.2	48.2990

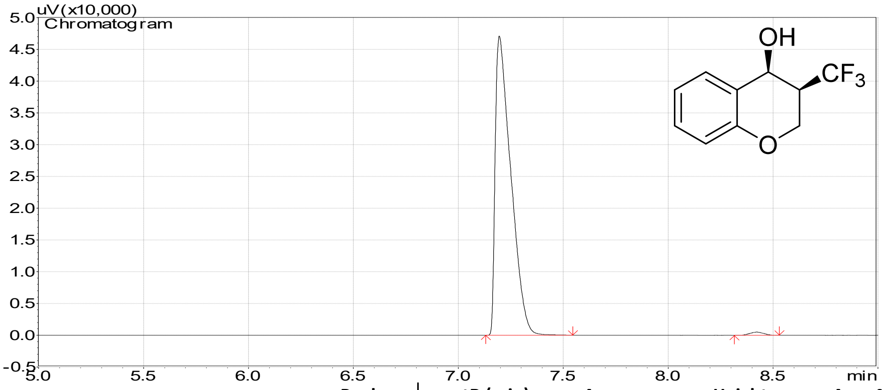

Peak	tR (min)	Area	Height	Area %
(*S*,*S*)-**2l**	7.196	244190.3	46993.5	98.9436
impurity	8.422	2607.2	534.4	1.0564

2m. 2-trifluoromethyl-1-benzosuberol.

- **Chiral GC. Top:** $NaBH_4$ reduction, d.r. = 90:10, **Bottom:** DKR-ATH using (*S*,*S*)-**C2**, d.r.> 99:1, 99.2 % ee

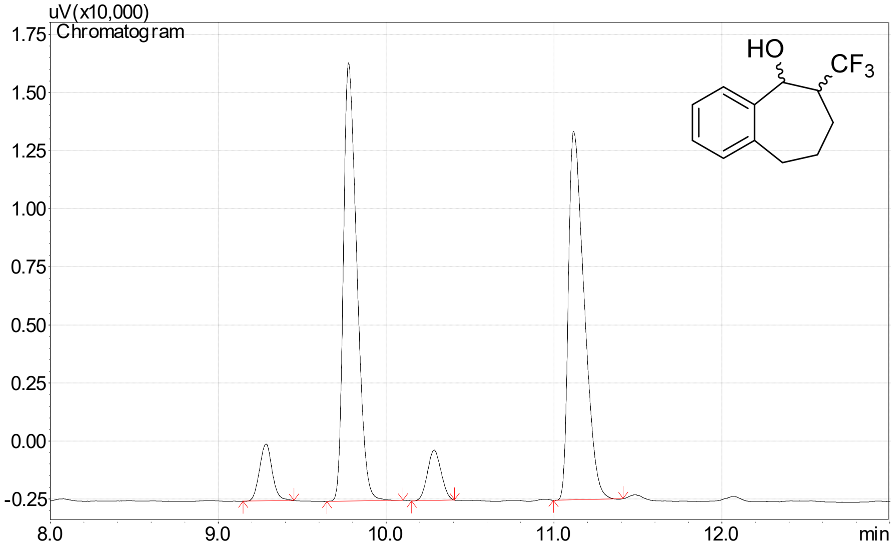

Peak	tR (min)	Area	Height	Area %
1	9.284	12864.7	2446.9	5.5069
2	9.776	104710.9	18846.8	44.8225
3	10.285	12270.0	2163.2	5.2523
4	11.116	103766.5	15839.0	44.4183

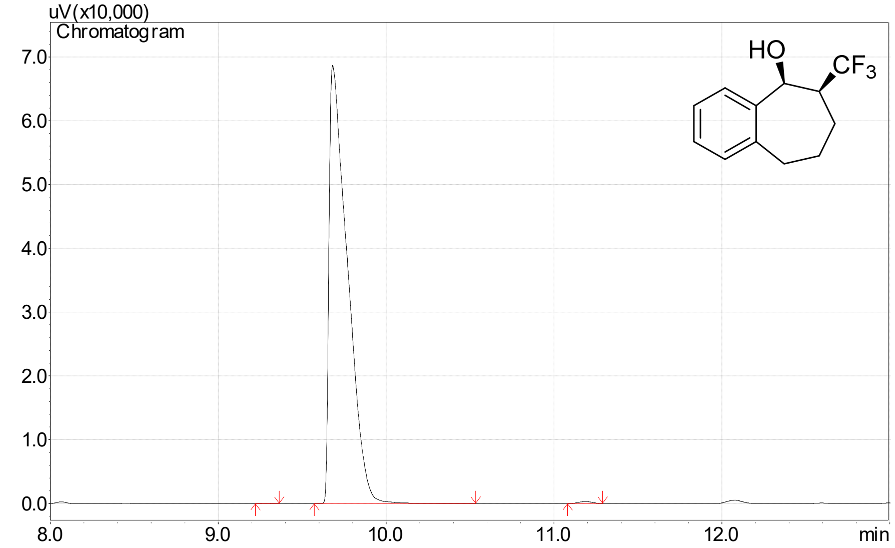

Peak	tR (min)	Area	Height	Area %
1	9.284	254.6	62.9	0.0484
2	9.682	523725.7	68612.7	99.5748
3	11.188	1981.6	323.1	0.3768

2n. 2-trifluoromethylthio-1-indanol.

- **^{19}F NMR. Top:** $NaBH_4$ reduction, d.r. = 58 : 42. **Bottom:** DKR-ATH, d.r. = 99.9 : 0.1 signal/noise = 18000

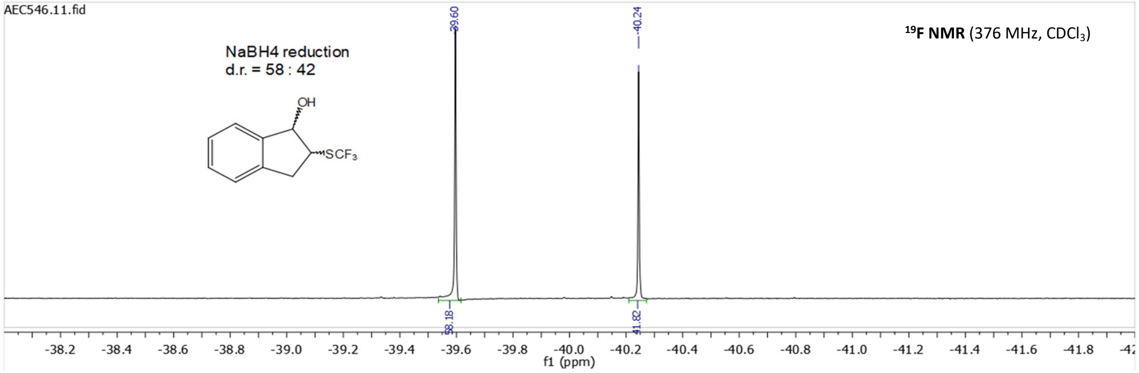

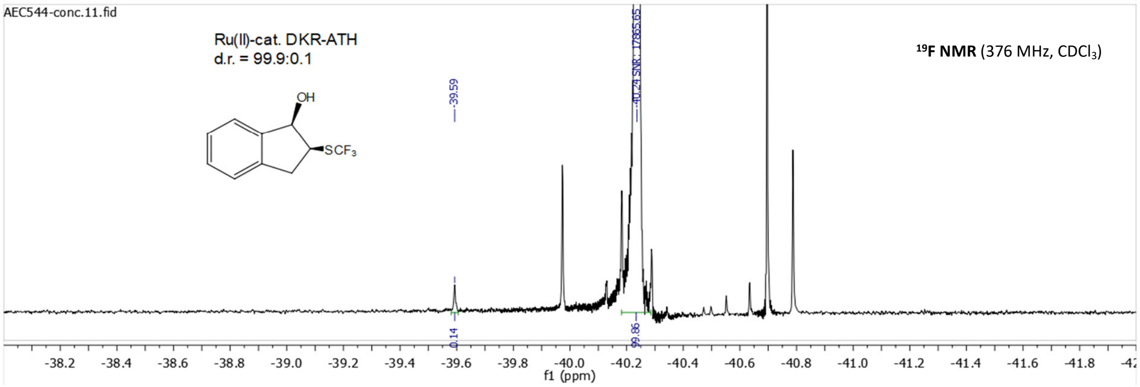

- **Chiral GC. Top:** $NaBH_4$ reduction, **Bottom:** DKR-ATH using (*S*,*S*)-**C2**, 99.8 % ee

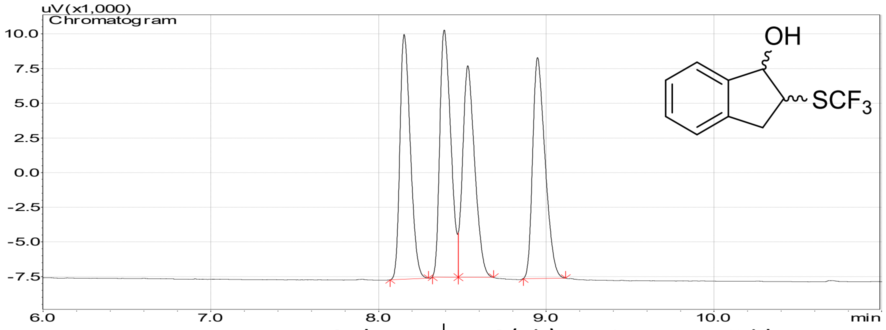

Peak	tR (min)	Area	Height	Area %
(1*S*,2*R*)-**2n**	8.152	76190.0	17621.4	24.1278
trans-**2n**	8.391	81369.1	17753.5	25.7679
(1*R*,2*S*)-**2n**	8.532	76317.4	15183.0	24.1681
ent-trans-**2n**	8.948	81900.8	15900.6	25.9362

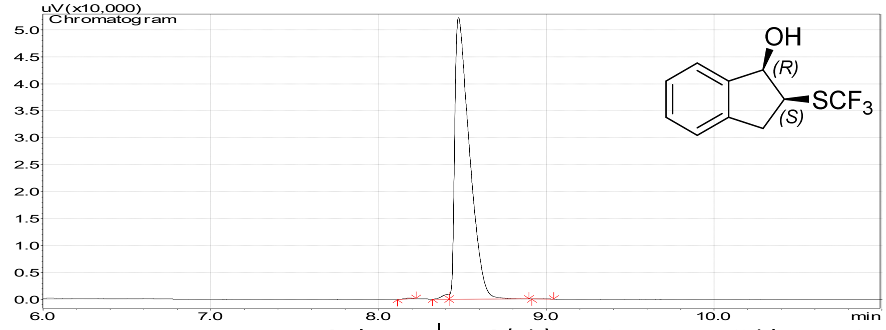

Peak	tR (min)	Area	Height	Area %
(1*S*,2*R*)-**2n**	8.182	447.3	147.4	0.1396
trans-**2n**	8.408	2480.8	850.4	0.7742
(1*R*,2*S*)-**2n**	8.476	317340.8	52128.1	99.0391
ent-trans-**2n**	8.984	150.9	36.5	0.0471

2o. 2-trifluoromethylthio-1-tetralol

- **19F NMR. Top:** $NaBH_4$ reduction, d.r. = 69 : 31. **Bottom:** DKR-ATH, d.r. = 99.9 : 0.1 signal/noise = 8000

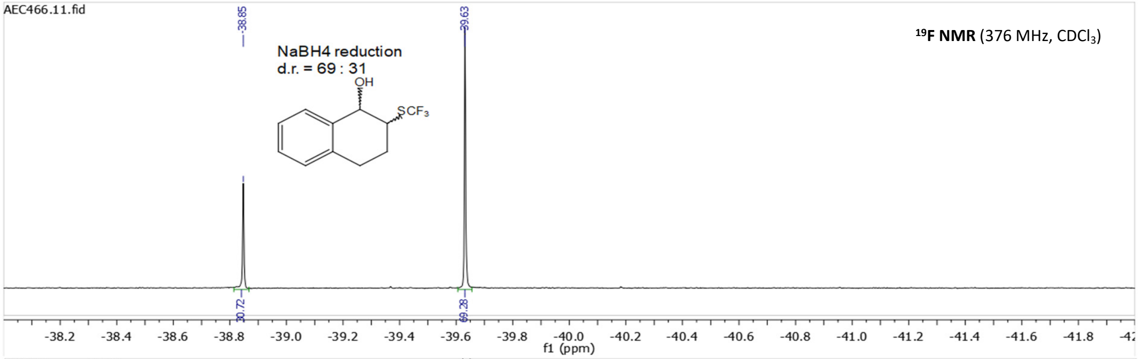

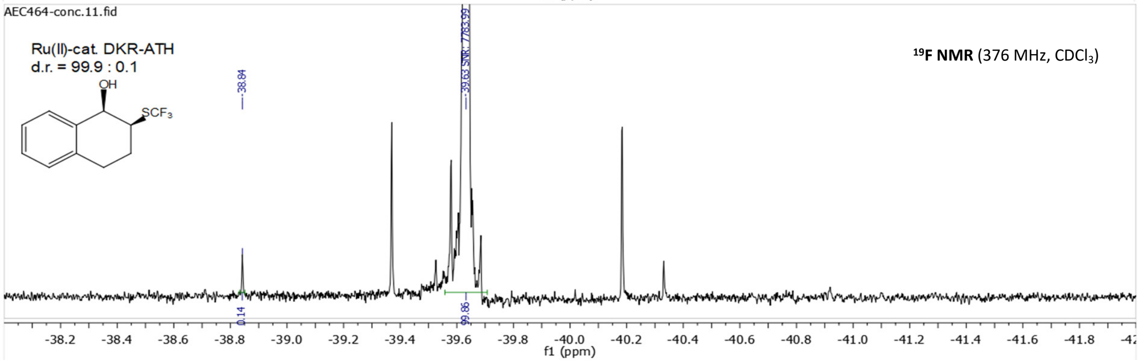

- **Chiral GC. Top:** $NaBH_4$ reduction, **Bottom:** DKR-ATH using (*S*,*S*)-**C2**, 99.8 % ee

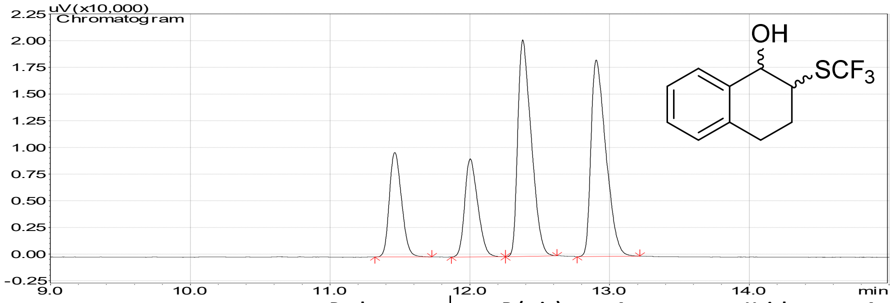

Peak	tR (min)	Area	Height	Area %
trans-**2o**	11.463	59687.9	9800.9	15.1910
ent-trans-**2o**	12.003	59694.9	9166.3	15.1928
(1*S*,2*R*)-**2o**	12.379	136587.2	20284.0	34.7625
(1*R*,2*S*)-**2o**	12.904	136945.3	18399.8	34.8537

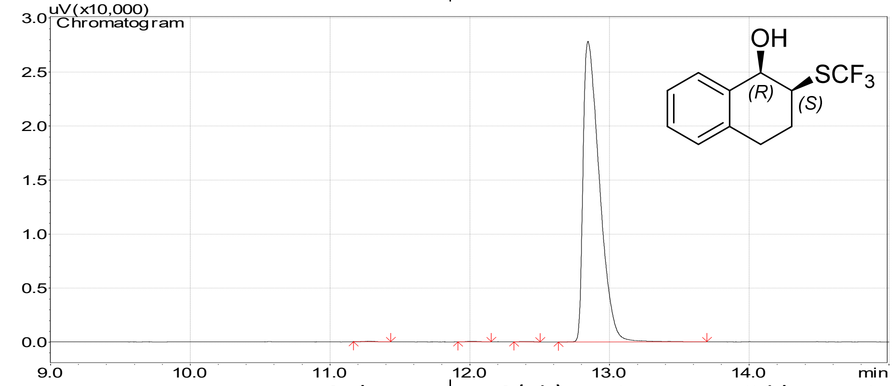

Peak	tR (min)	Area	Height	Area %
trans-**2o**	11.278	221.8	50.1	0.0978
ent-trans-**2o**	12.009	327.5	51.0	0.1444
(1*S*,2*R*)-**2o**	12.444	144.7	27.2	0.0638
(1*R*,2*S*)-**2o**	12.846	226050.3	27793.7	99.6939

2p. 2-trifluoromethyloxy-1-indanol.

- **19F NMR. Top:** $NaBH_4$ reduction, d.r. = 67 : 33. **Bottom:** DKR-ATH, d.r. = 99 : 1 signal/noise = 9100

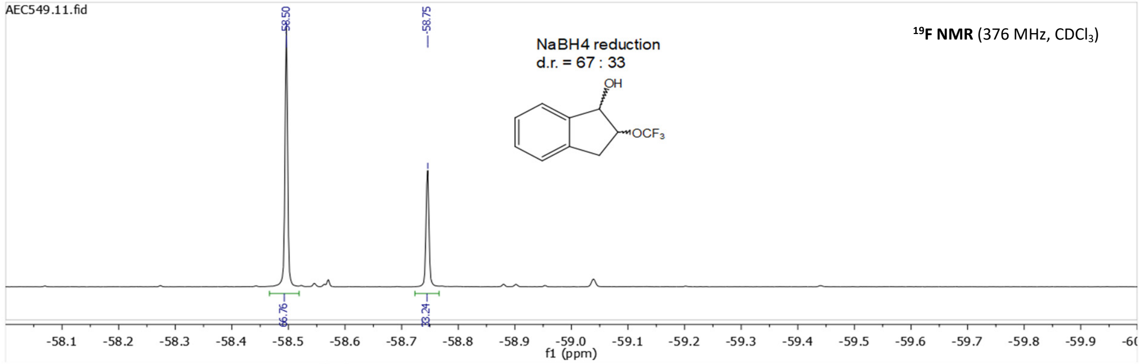

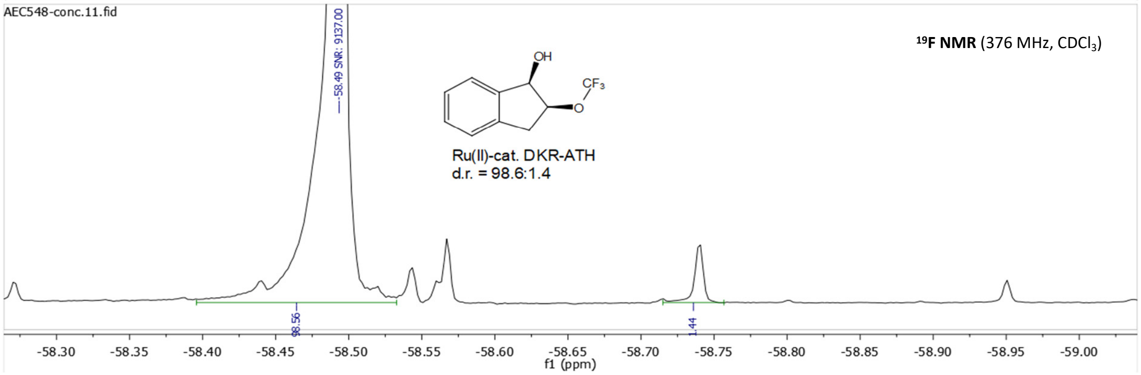

- **Chiral GC. Top:** $NaBH_4$ reduction, **Bottom:** DKR-ATH using (*S*,*S*)-**C2**, 95.7 % ee

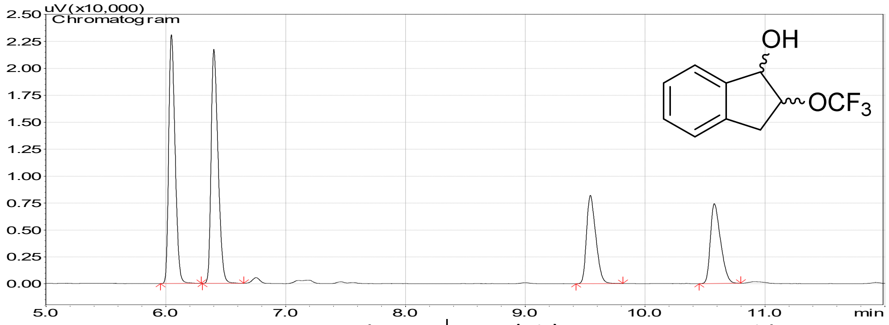

Peak	tR (min)	Area	Height	Area %
(1*S*,2*R*)-**2p**	6.046	85438.8	23042.8	32.7861
(1*R*,2*S*)-**2p**	6.401	87511.8	21684.3	33.5816
trans-**2p**	9.543	43995.6	8170.9	16.8828
ent-trans-**2p**	10.577	43648.0	7400.9	16.7494

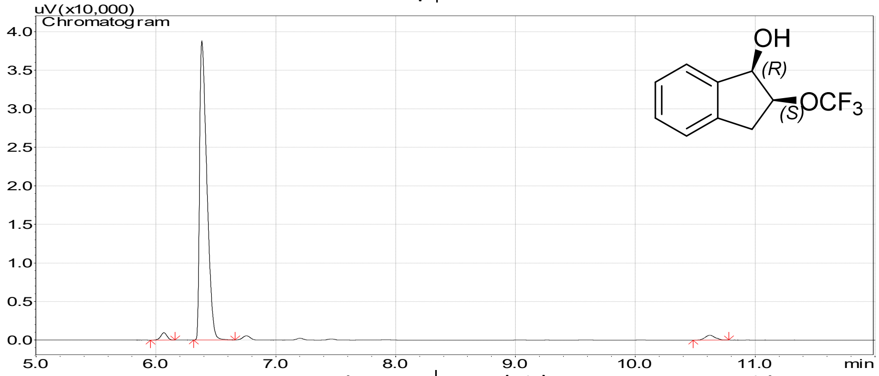

Peak	tR (min)	Area	Height	Area %
(1*S*,2*R*)-**2p**	6.067	3573.9	969.2	2.0963
(1*R*,2*S*)-**2p**	6.384	162994.8	38737.9	95.6066
ent-trans-**2p**	10.622	3916.2	649.9	2.2971

2q. 3,3,3-trifluoro-2-methyl-1-phenylpropan-1-ol.

- **^{19}F NMR. Top:** NaBH$_4$ reduction, d.r. = 86:14. **Bottom:** DKR-ATH, d.r. = 20:80.

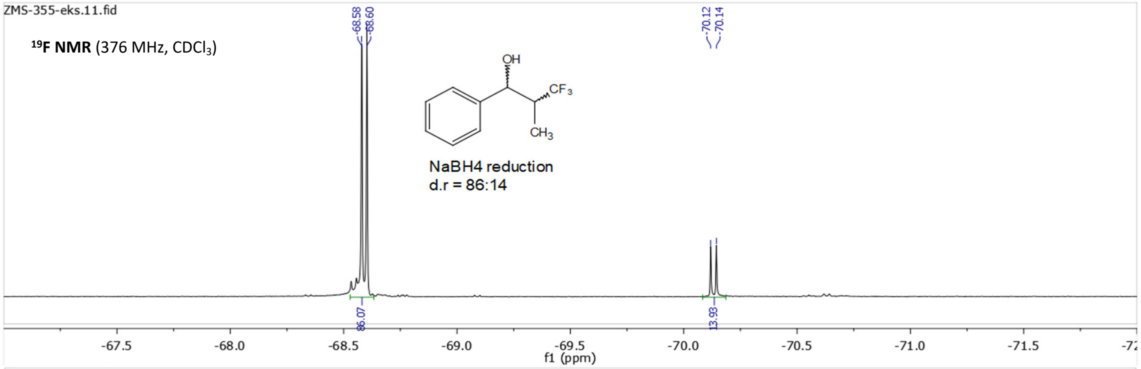

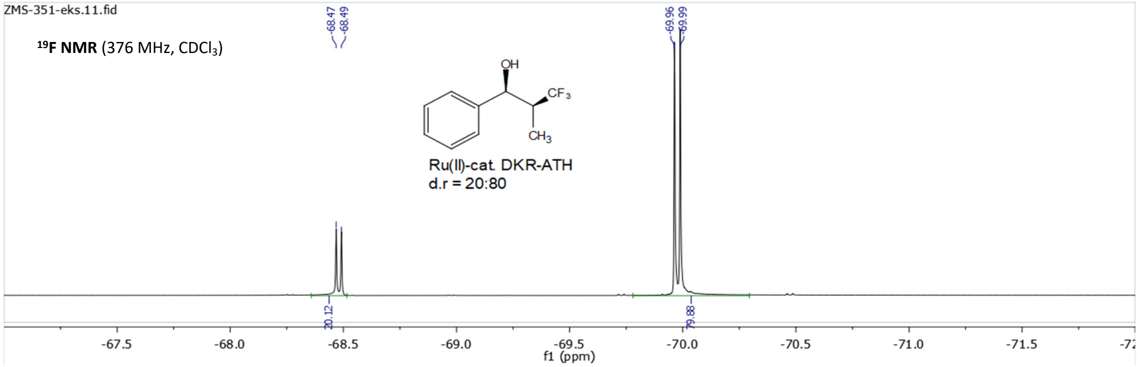

- **Chiral GC. Top:** NaBH$_4$ reduction, **Bottom:** DKR-ATH using (*S*,*S*)-**C2**, 97.5 % ee

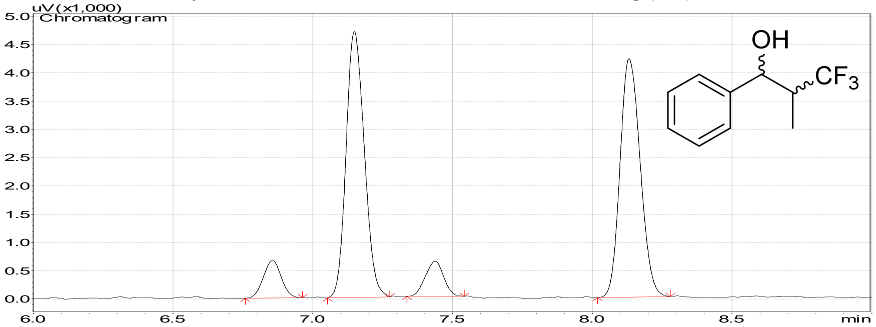

Peak	tR (min)	Area	Height	Area %
(*S*,*S*)-**2q**	6.857	2950.5	662.0	6.1091
(*R**,*S**)-**2q**	7.149	21349.7	4695.7	44.2050
(*R*,*R*)-**2q**	7.438	2833.9	619.9	5.8677
ent-(*R**,*S**)-**2q**	8.132	21162.9	4207.0	43.8182

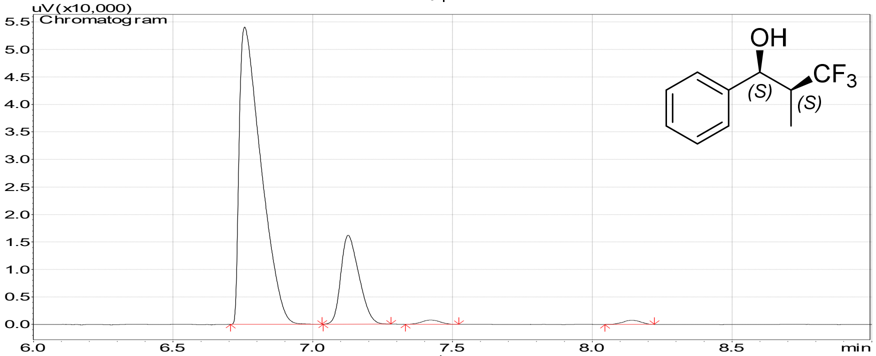

Peak	tR (min)	Area	Height	Area %
(*S*,*S*)-**2q**	6.756	292820.5	54038.8	78.4552
(*R**,*S**)-**2q**	7.127	72935.0	16093.5	19.5414
(*R*,*R*)-**2q**	7.422	3787.2	806.9	1.0147
ent-(*R**,*S**)-**2q**	8.143	3690.0	794.2	0.9887

2r. 1-trifluoromethylthio-2-indanol.

- **^{1}H NMR. Top:** $NaBH_4$ reduction, d.r. = 3:2. **Bottom:** DKR-ATH, d.r. = 95:5.

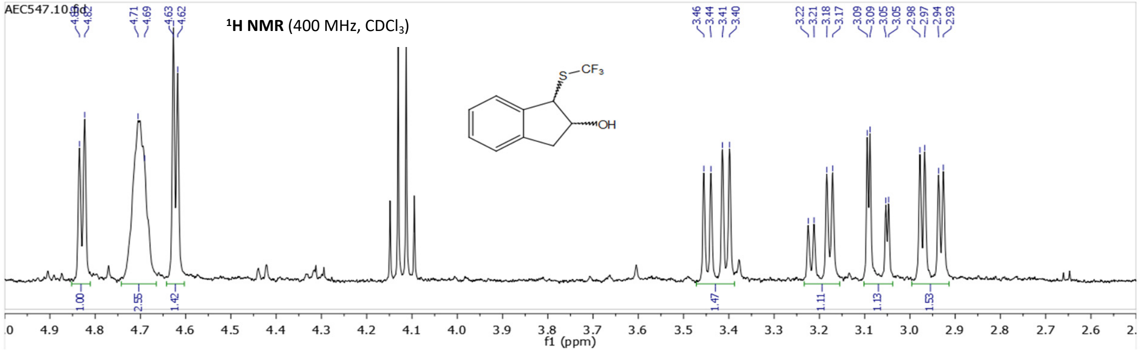

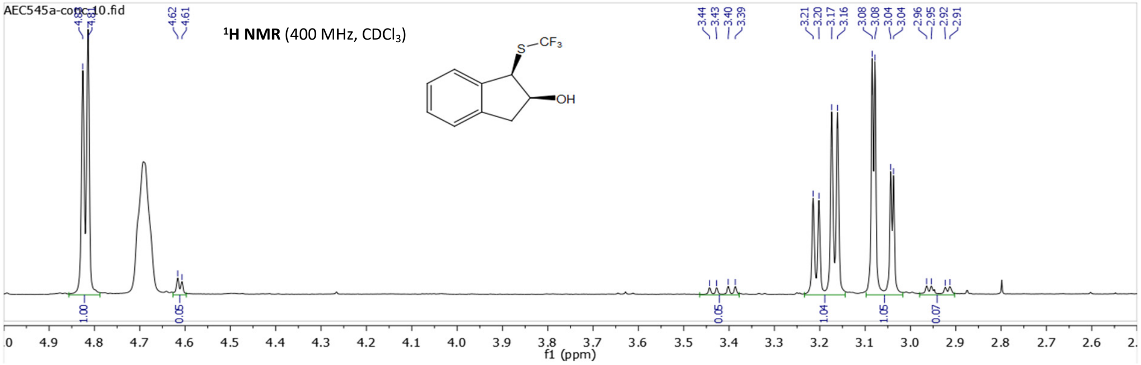

- **Chiral GC. Top:** $NaBH_4$ reduction, **Bottom:** DKR-ATH using (*S*,*S*)-**C2**, 45 % ee

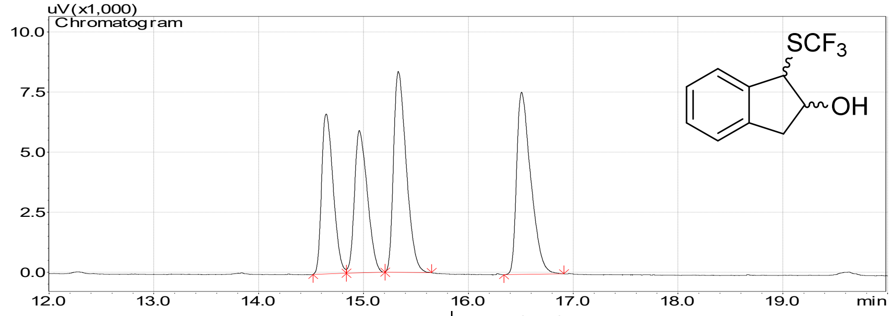

Peak	tR (min)	Area	Height	Area %
cis-**2r**	14.644	51133.9	6666.5	20.9892
ent-cis-**2r**	14.960	50481.9	5928.9	20.7215
trans-**2r**	15.332	70387.2	8354.4	28.8922
ent-trans-**2r**	16.509	71617.3	7588.0	29.3971

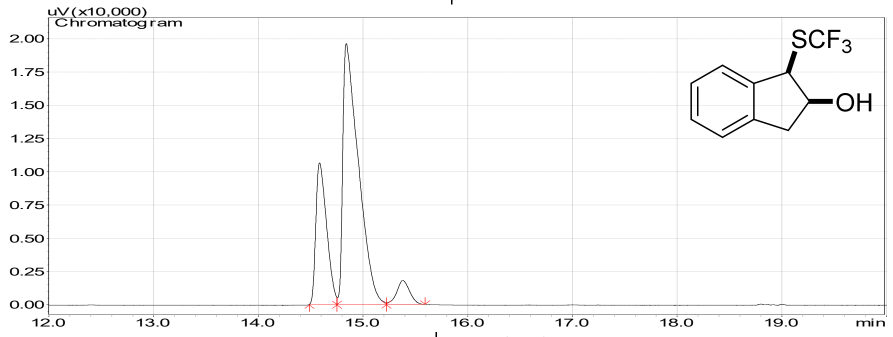

Peak	tR (min)	Area	Height	Area %
cis-**2r**	14.585	76667.7	10660.2	25.8321
ent-cis-**2r**	14.840	204469.9	19621.0	68.8931
trans-**2r**	15.381	15655.3	1821.7	5.2748

NMR spectra

Compound **1h**, ^{1}H NMR:

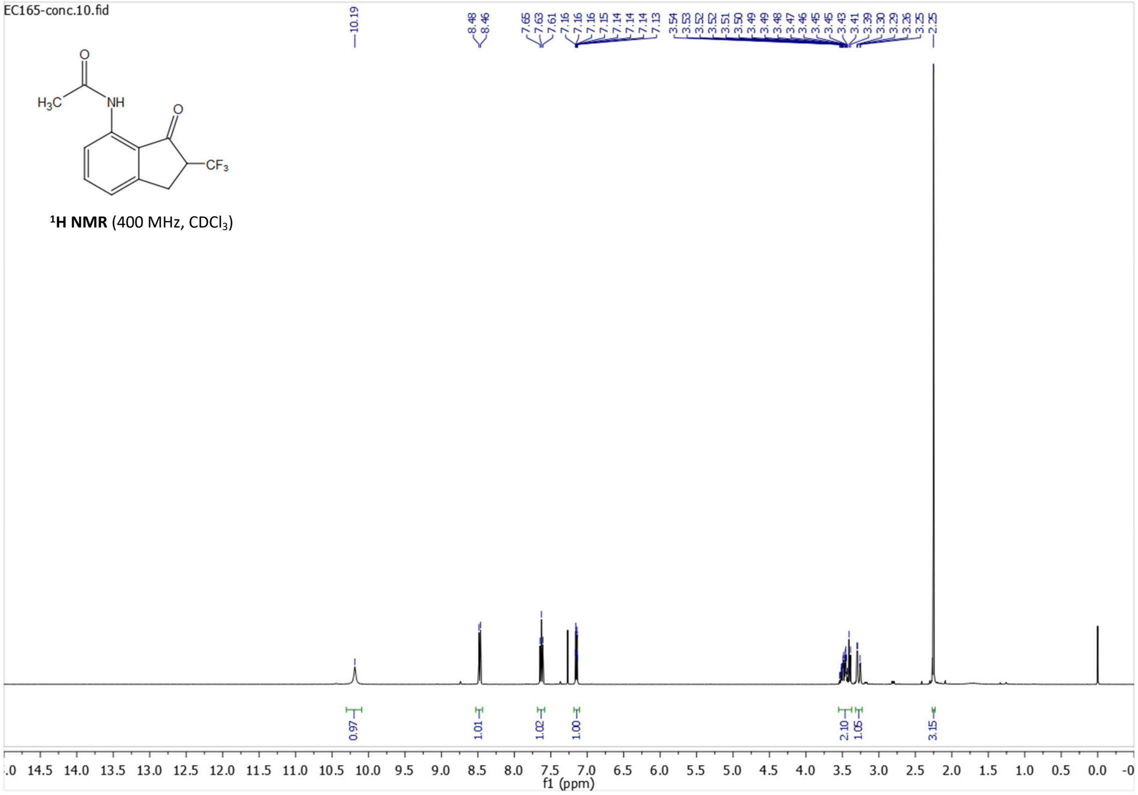

Compound **1h**, ^{13}C{^{1}H} NMR:

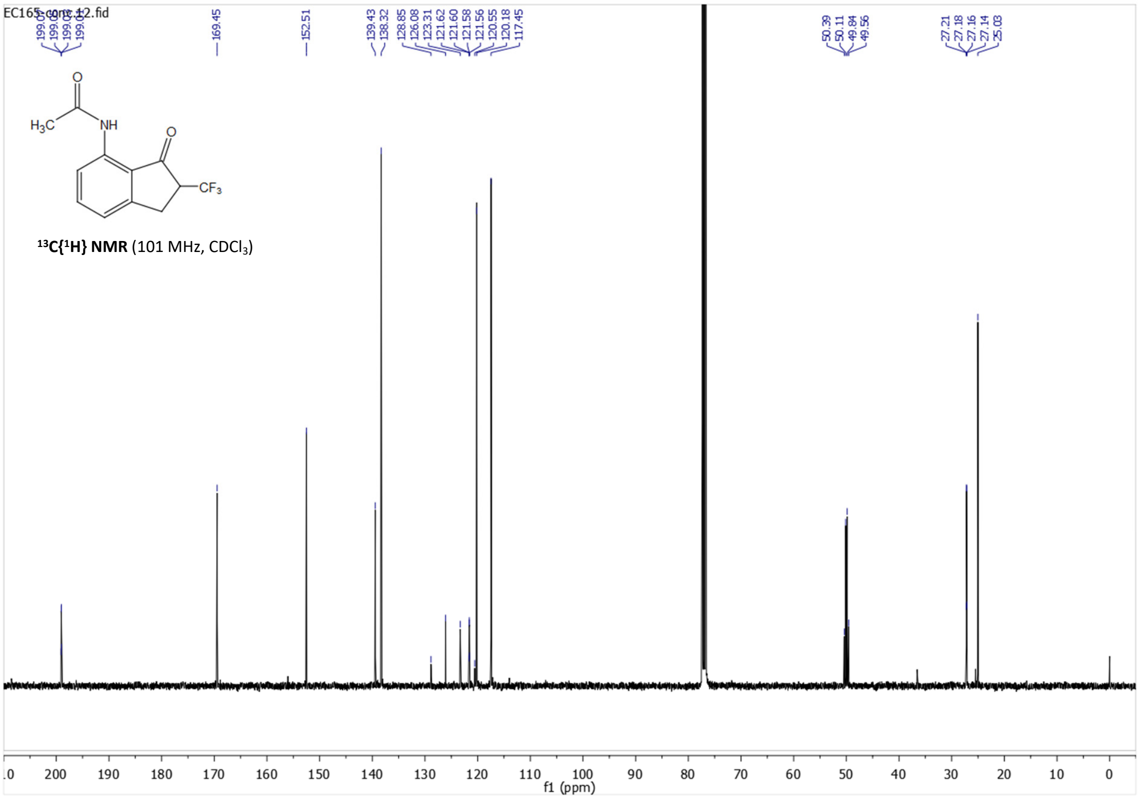

Compound **1h**, HSQC NMR:

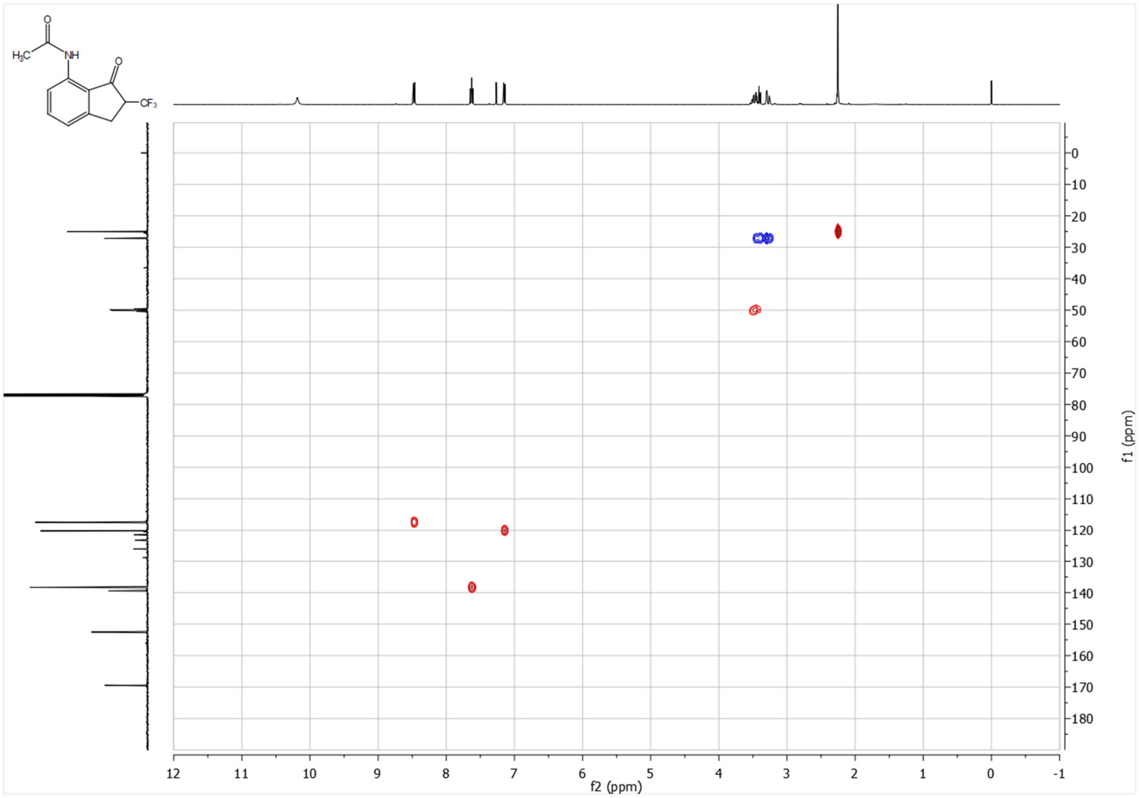

Compound **1h**, HMBC NMR:

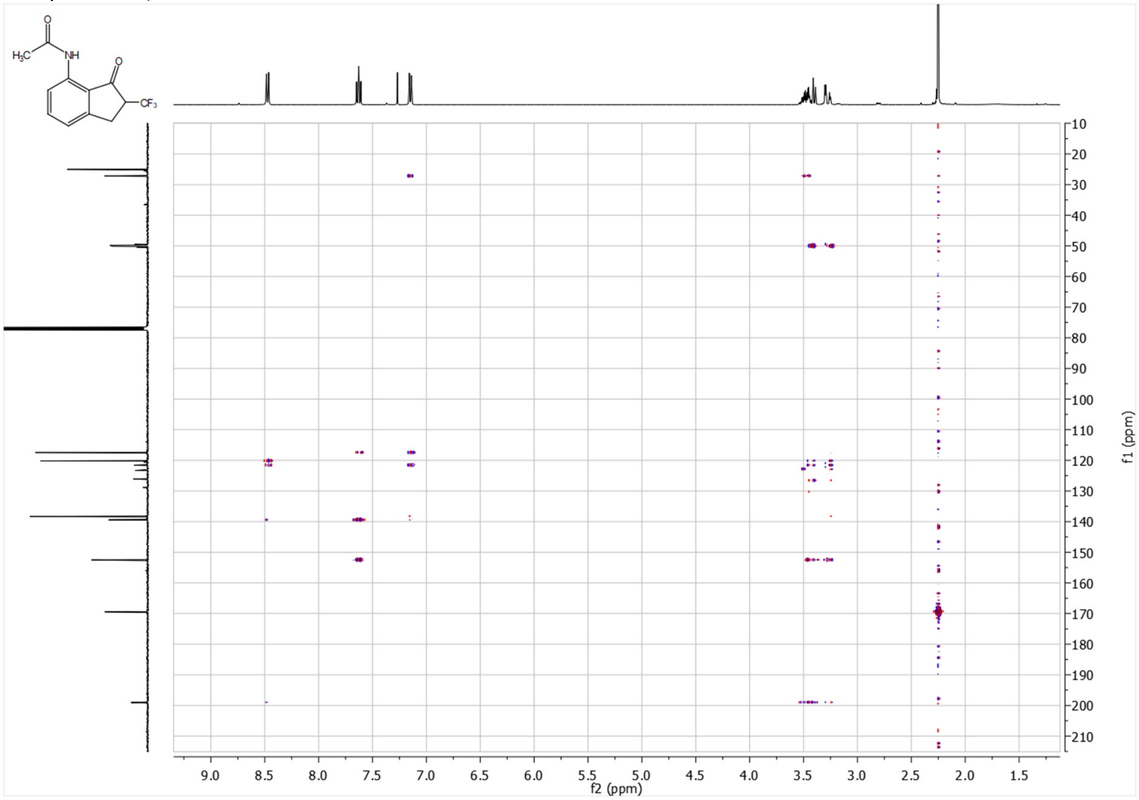

Compound **2a**, ^{1}H NMR:

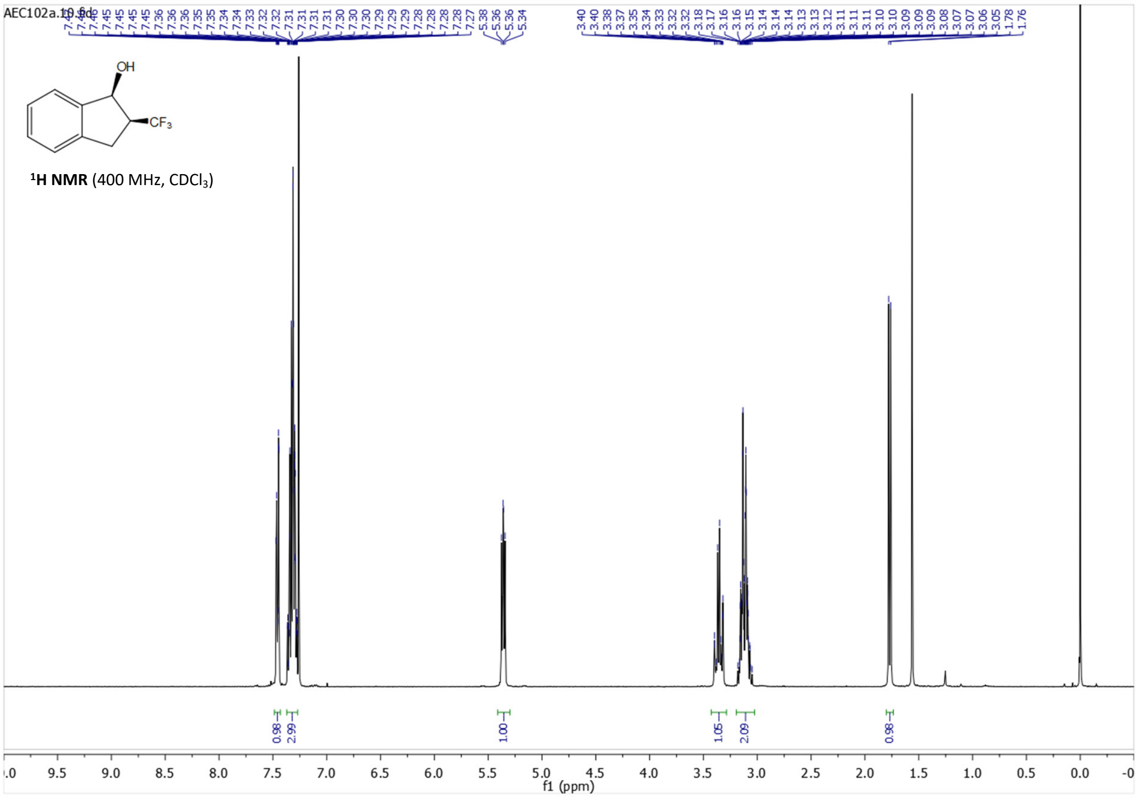

Compound **2a**, ^{13}C{^{1}H} NMR:

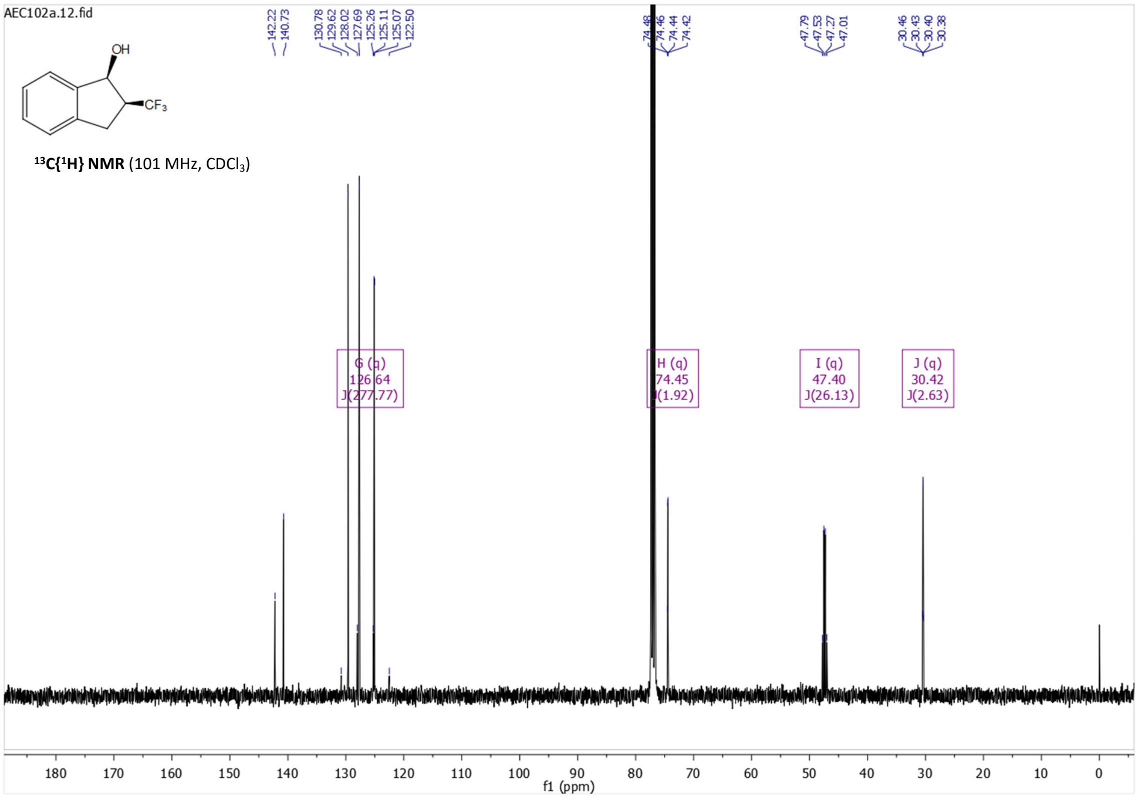

Compound **2b**, ^{1}H NMR:

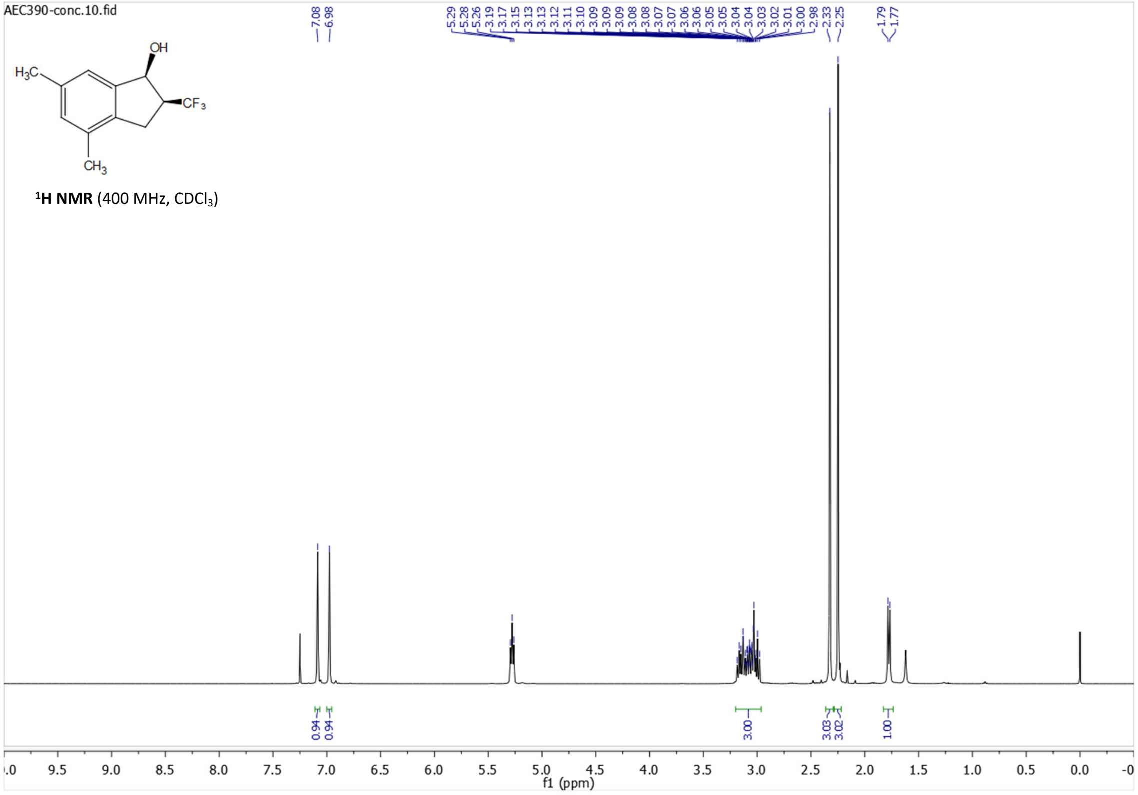

Compound **2b**, ^{13}C{^{1}H} NMR:

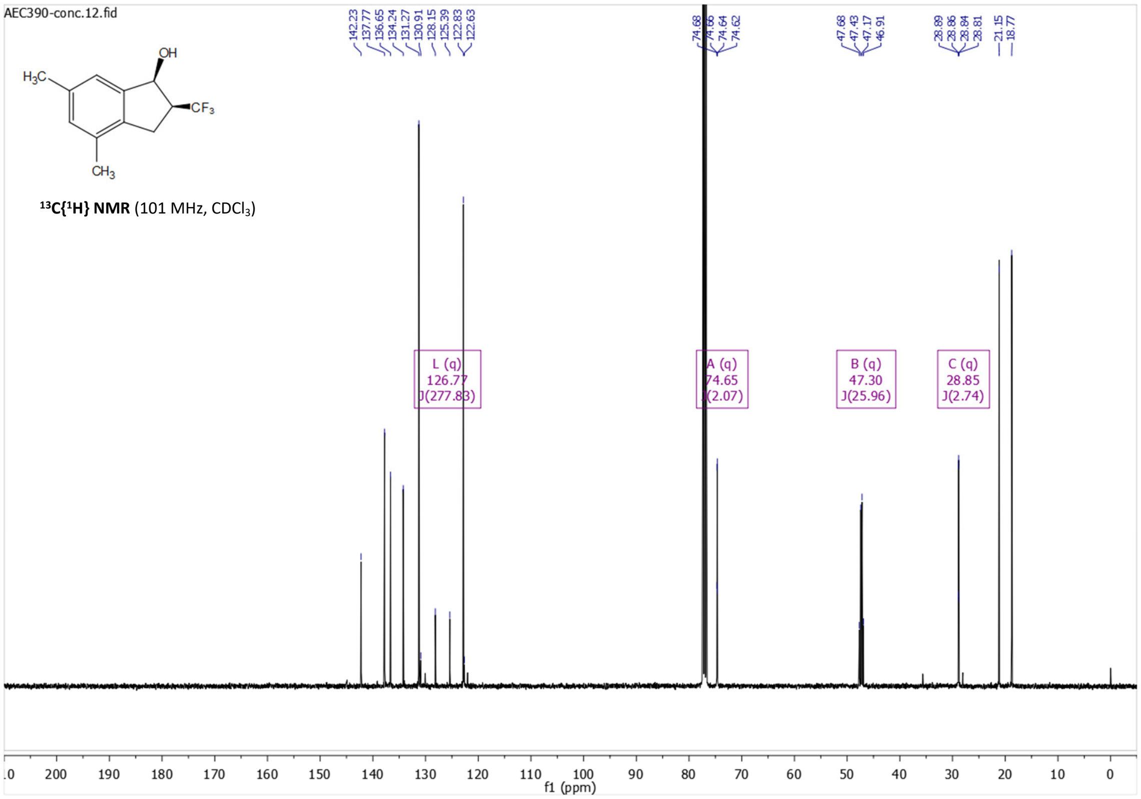

Compound **2c**, ^{1}H NMR:

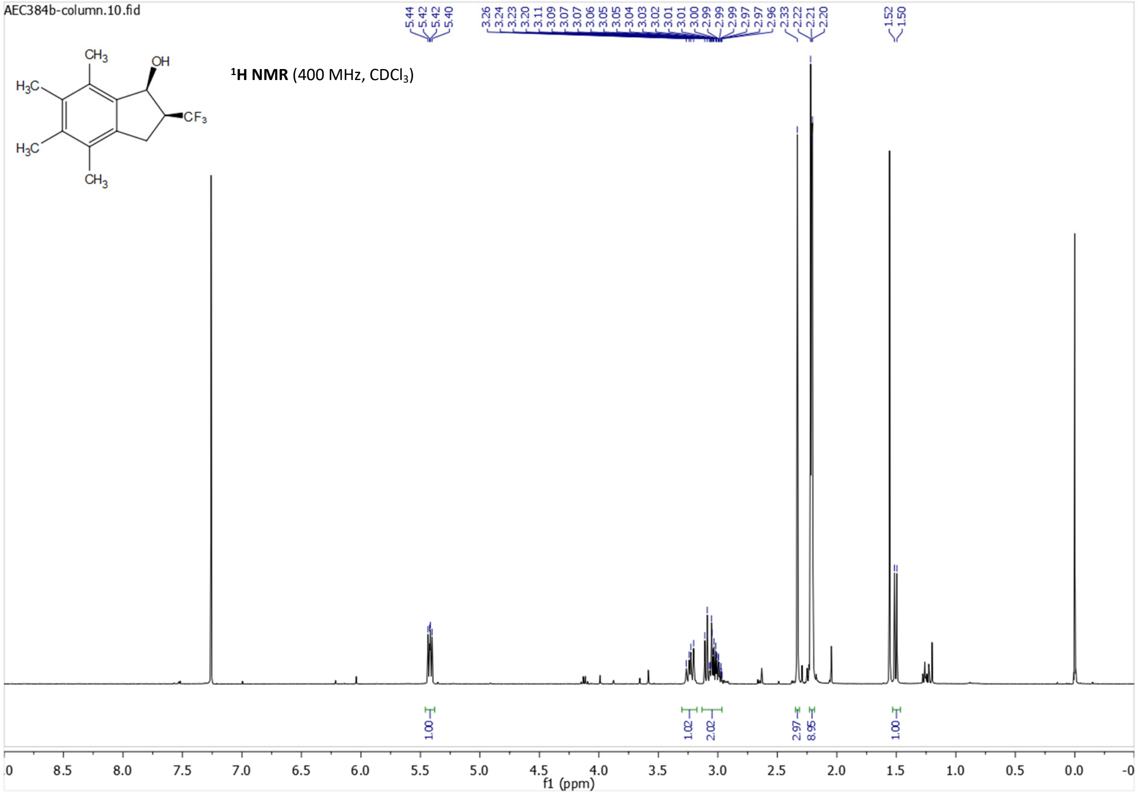

Compound **2c**, ^{13}C{^{1}H} NMR:

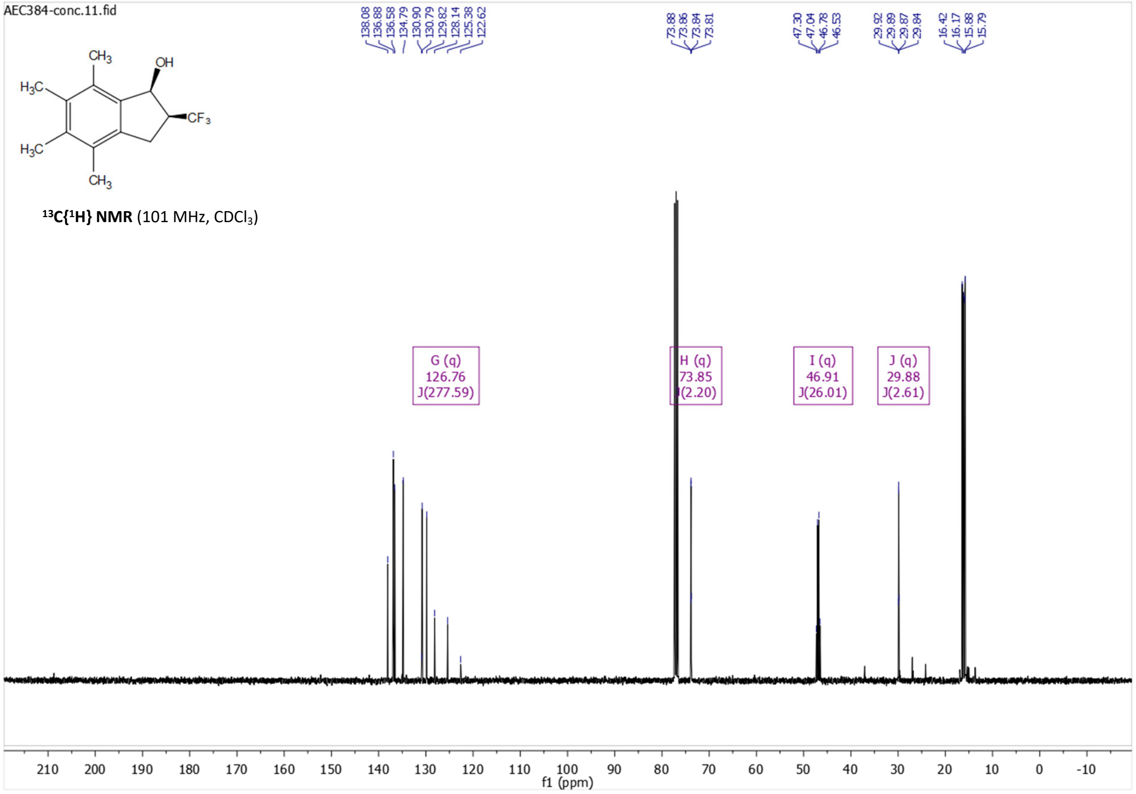

Compound **2d**, ^{1}H NMR:

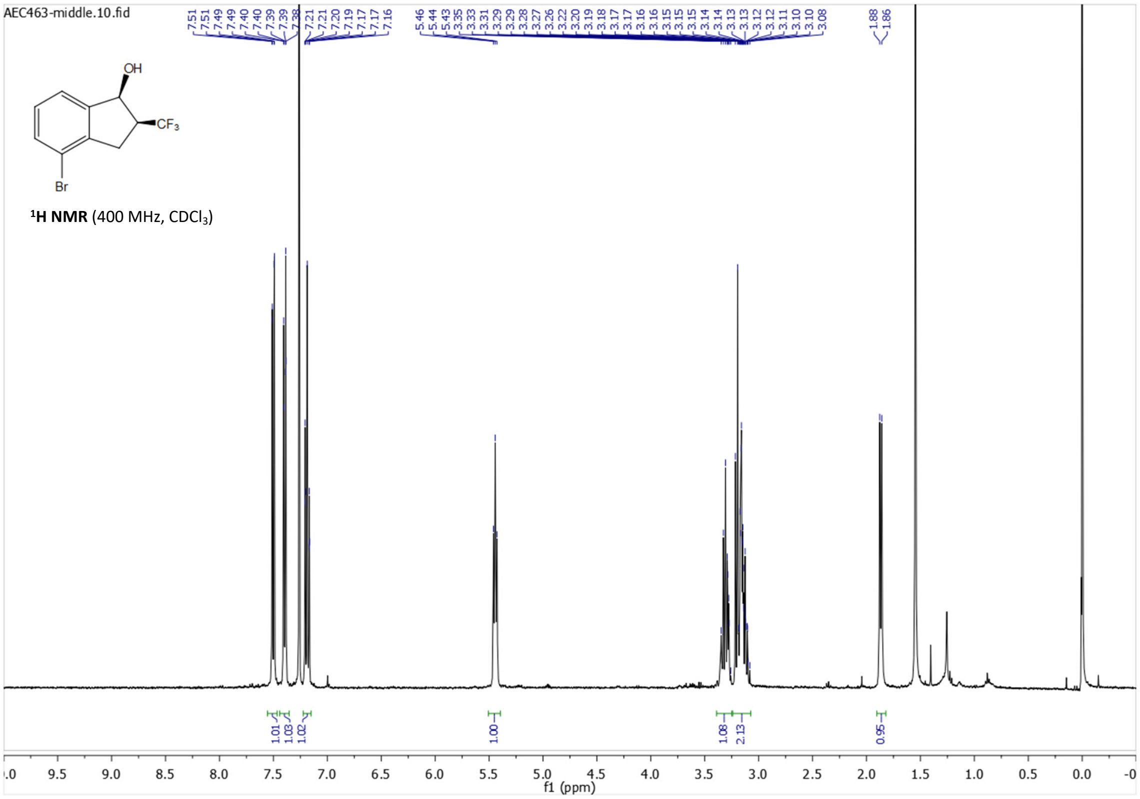

Compound **2d**, ^{13}C{^{1}H} NMR:

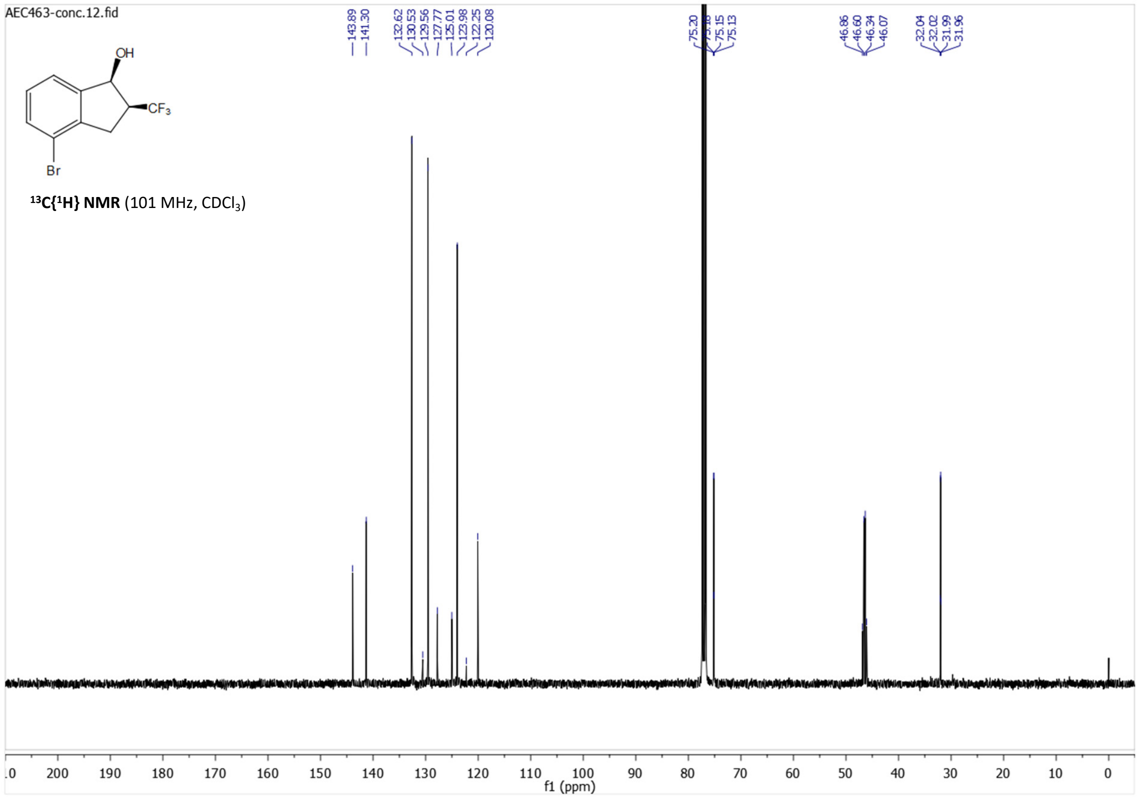

Compound **2e**, ^{1}H NMR:

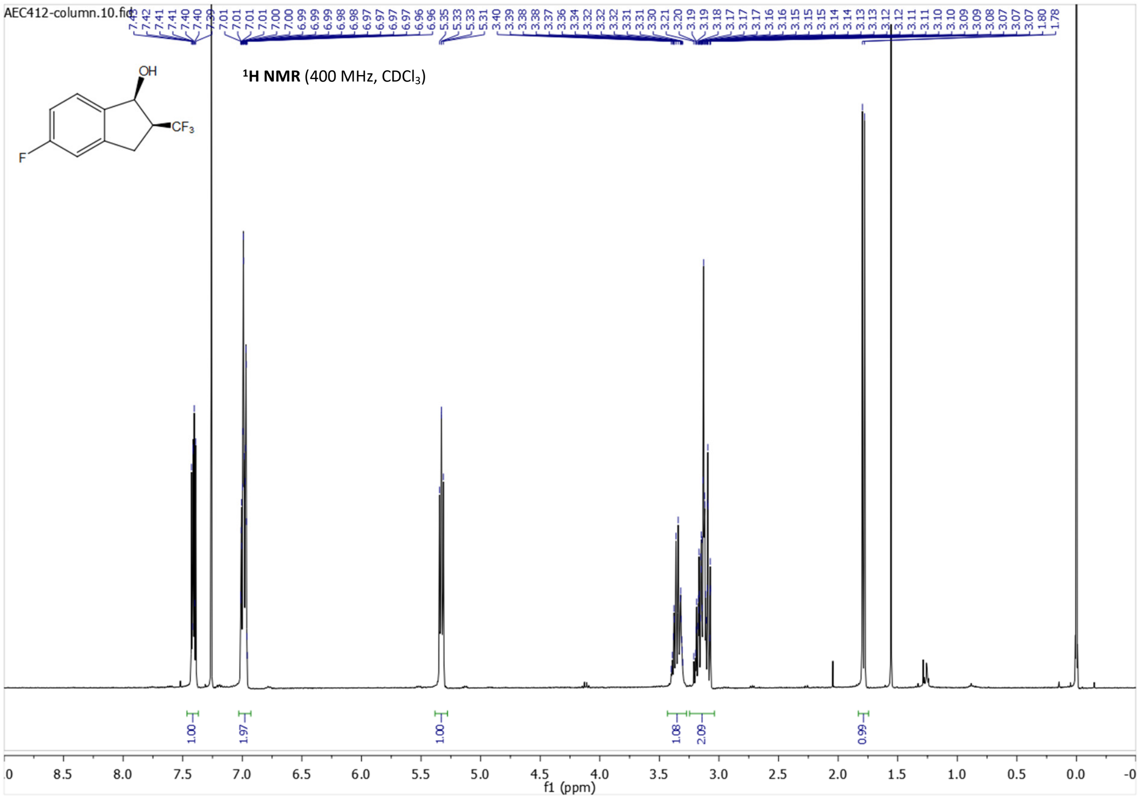

Compound **2e**, ^{13}C{^{1}H} NMR:

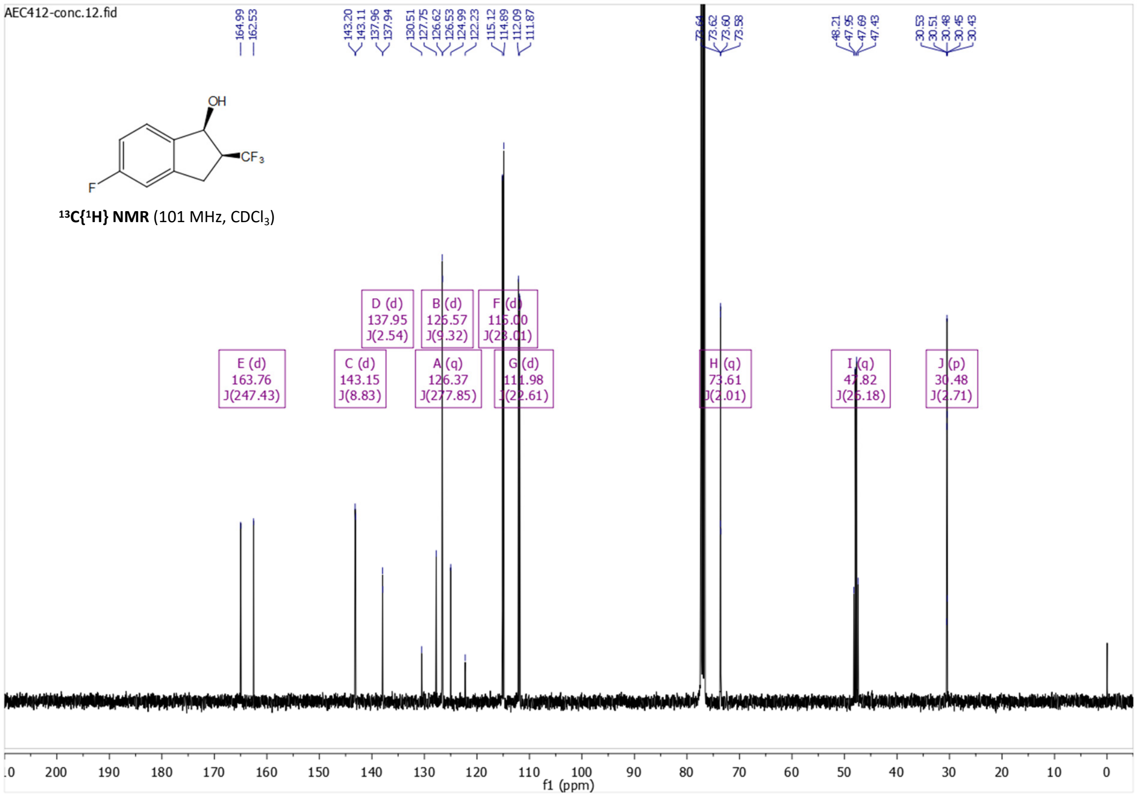

Compound **2f**, ^{1}H NMR:

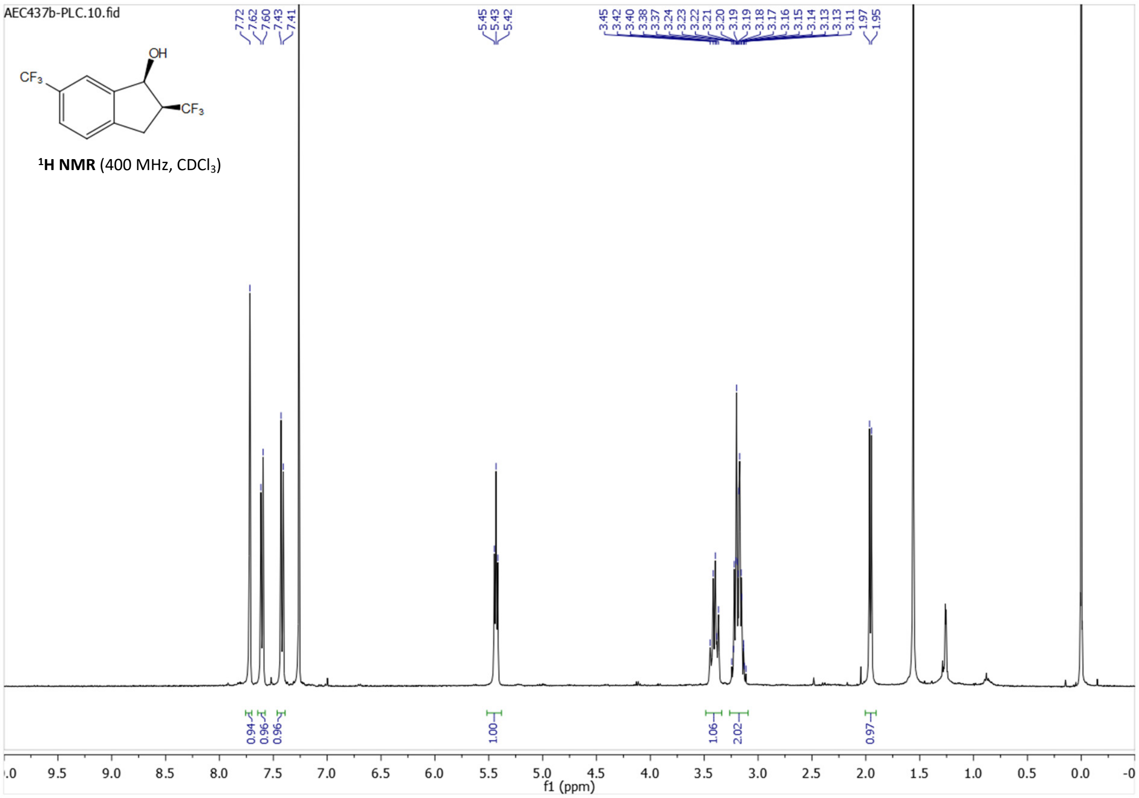

Compound **2f**, ^{13}C{^{1}H} NMR:

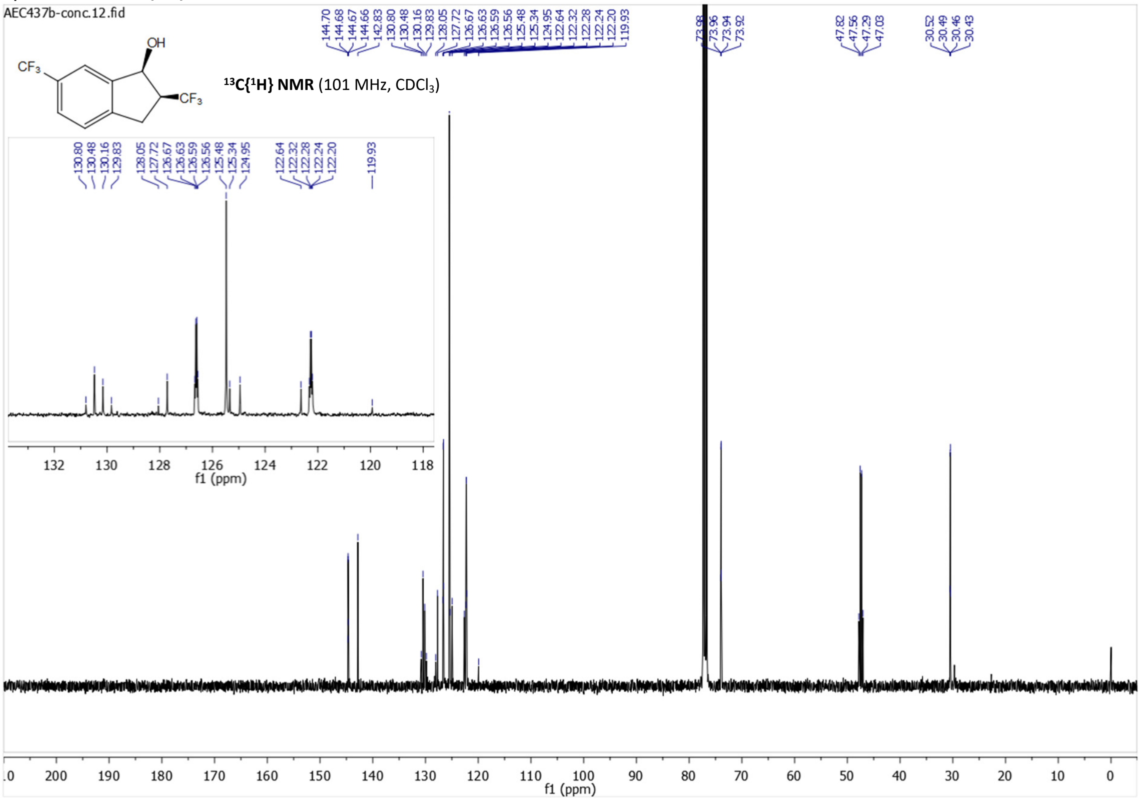

Compound **2g**, ^{1}H NMR:

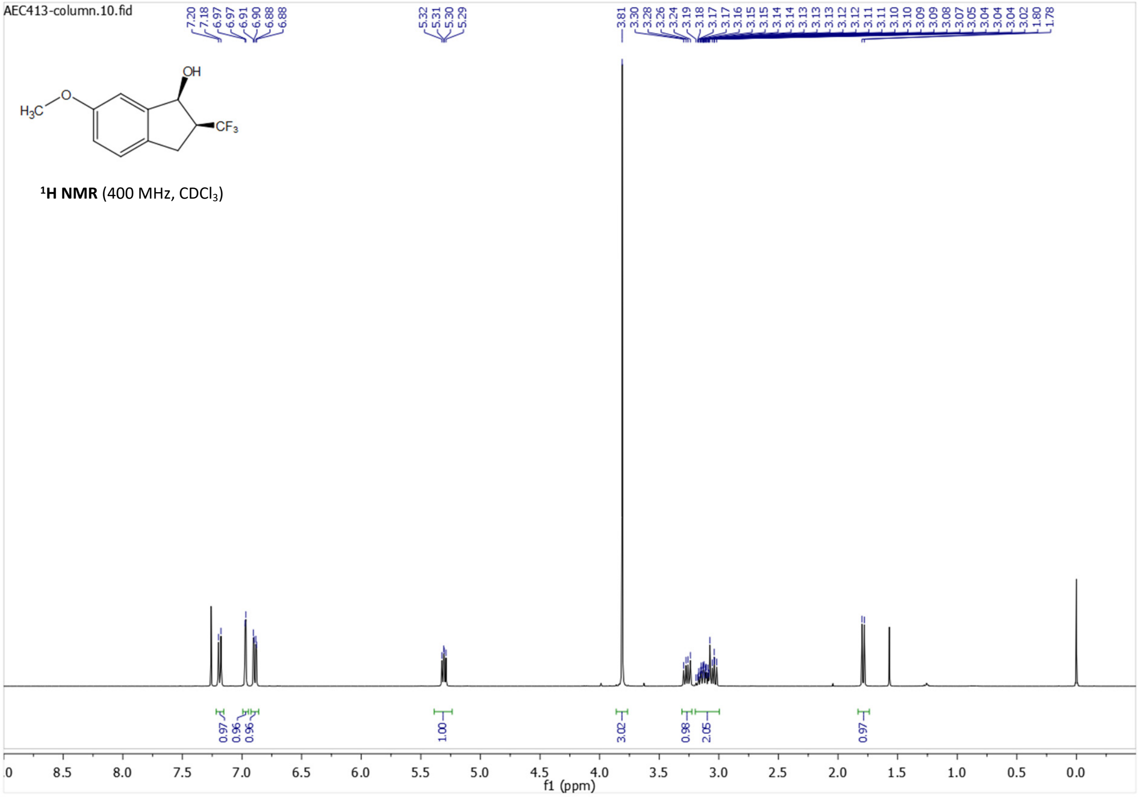

Compound **2g**, ^{13}C{^{1}H} NMR:

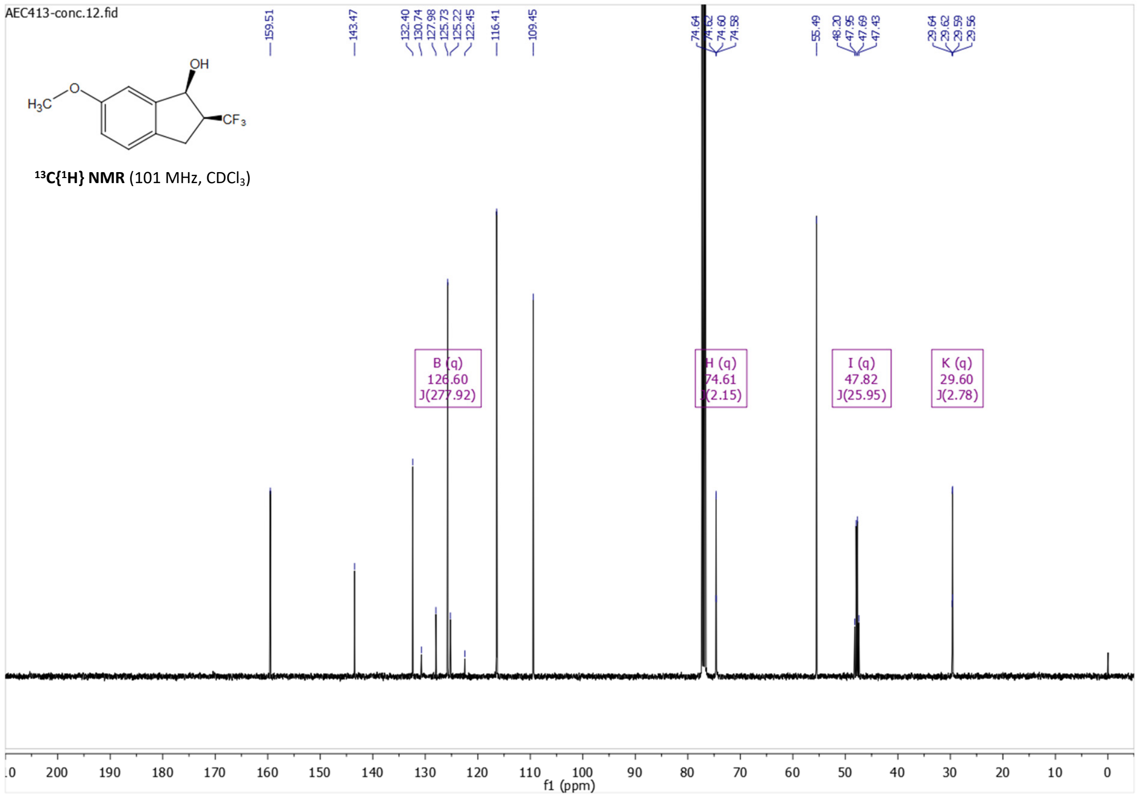

Compound **2h**, ^{1}H NMR:

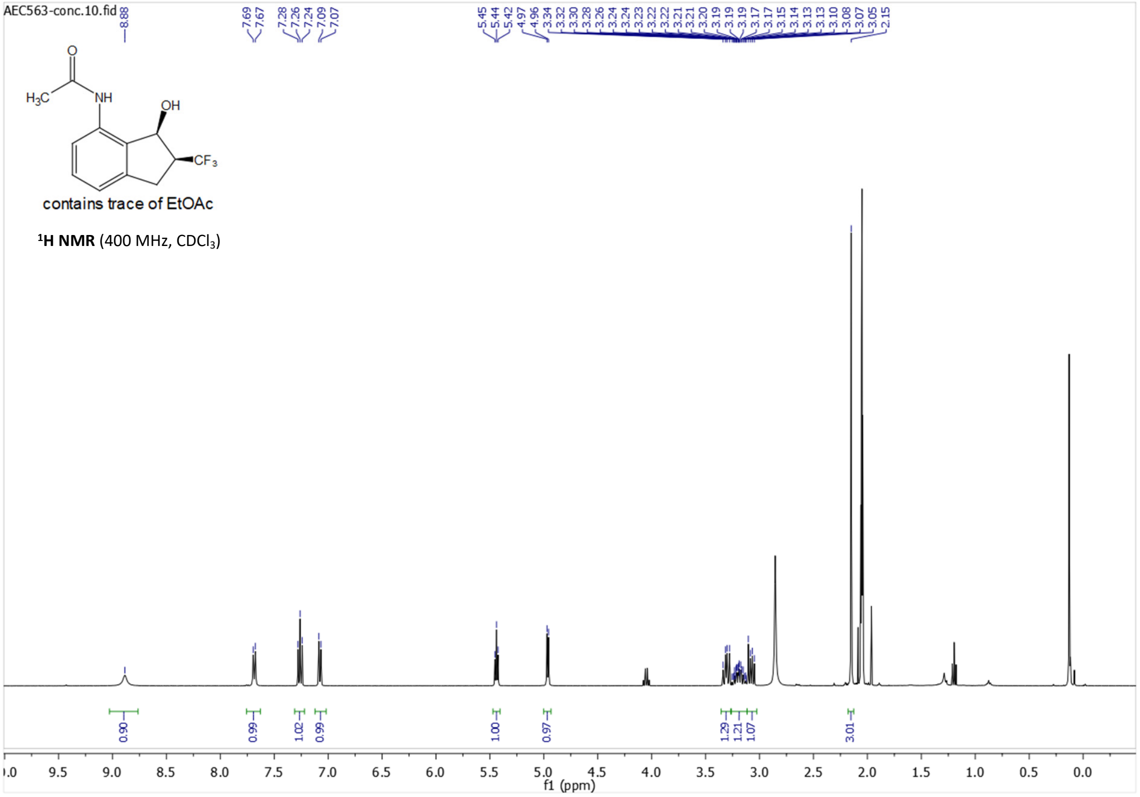

Compound **2h**, ^{13}C{^{1}H} NMR:

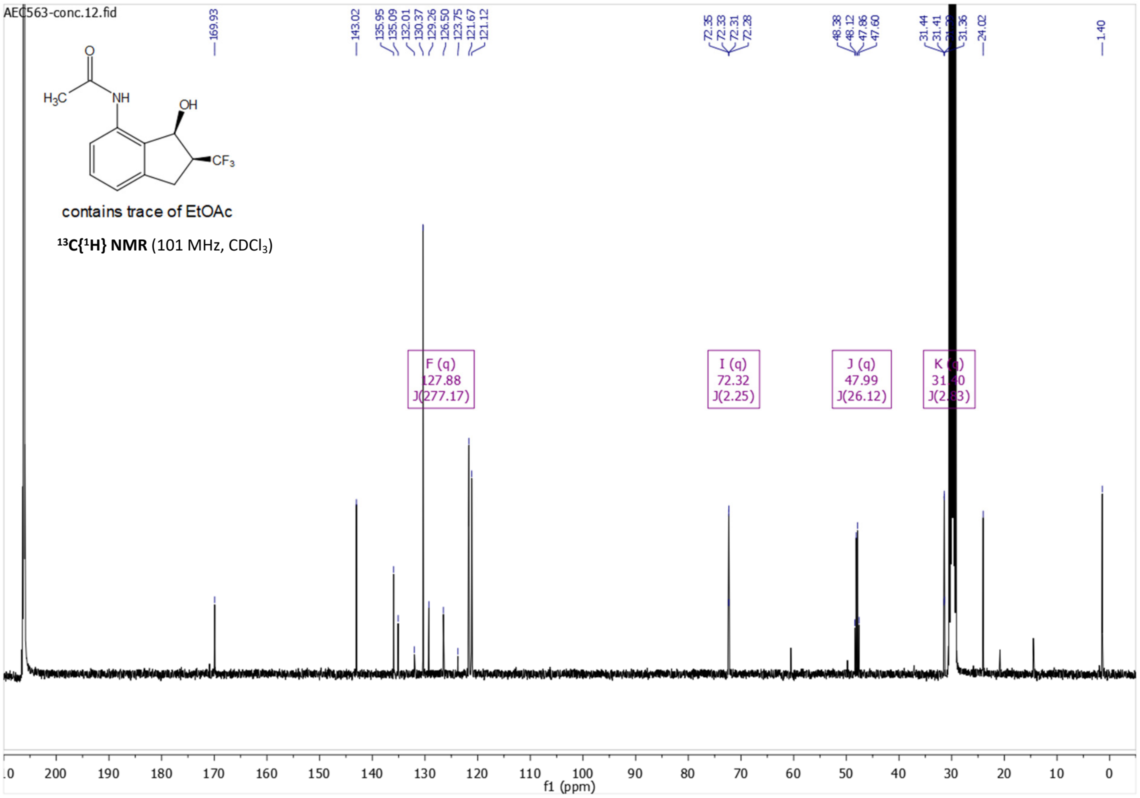

Compound **2i**, ^{1}H NMR:

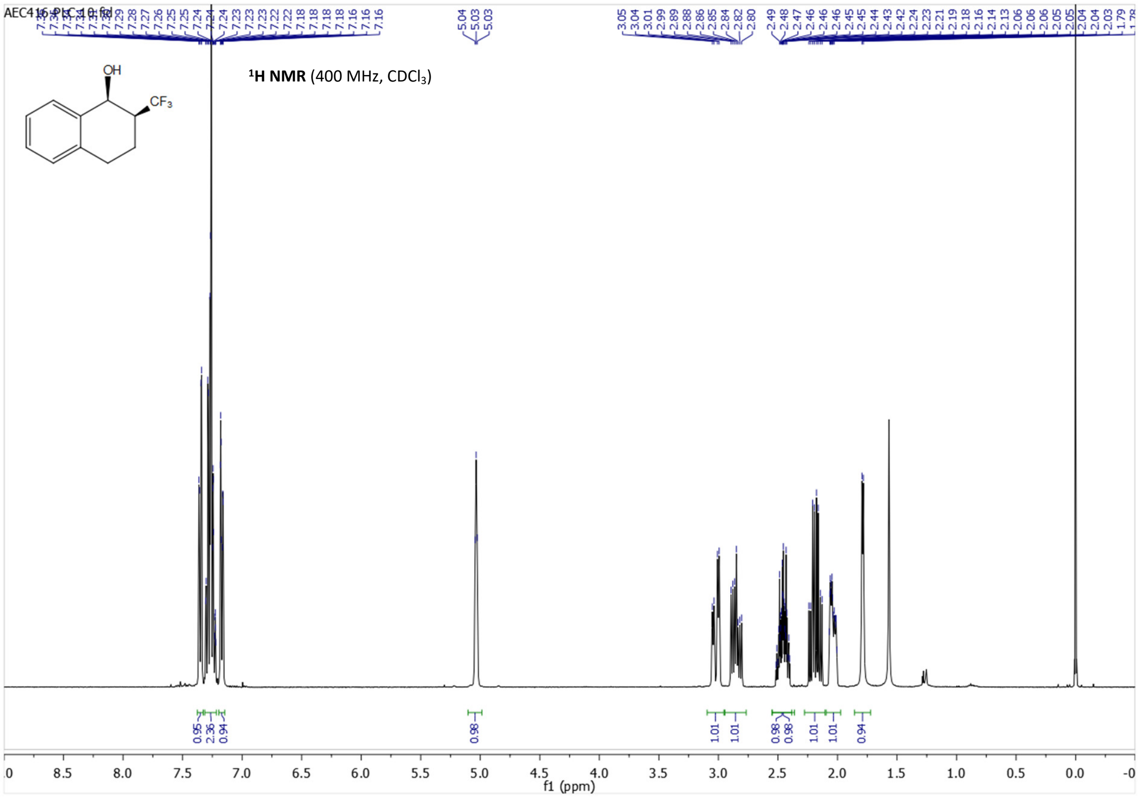

Compound **2i**, ^{13}C{^{1}H} NMR:

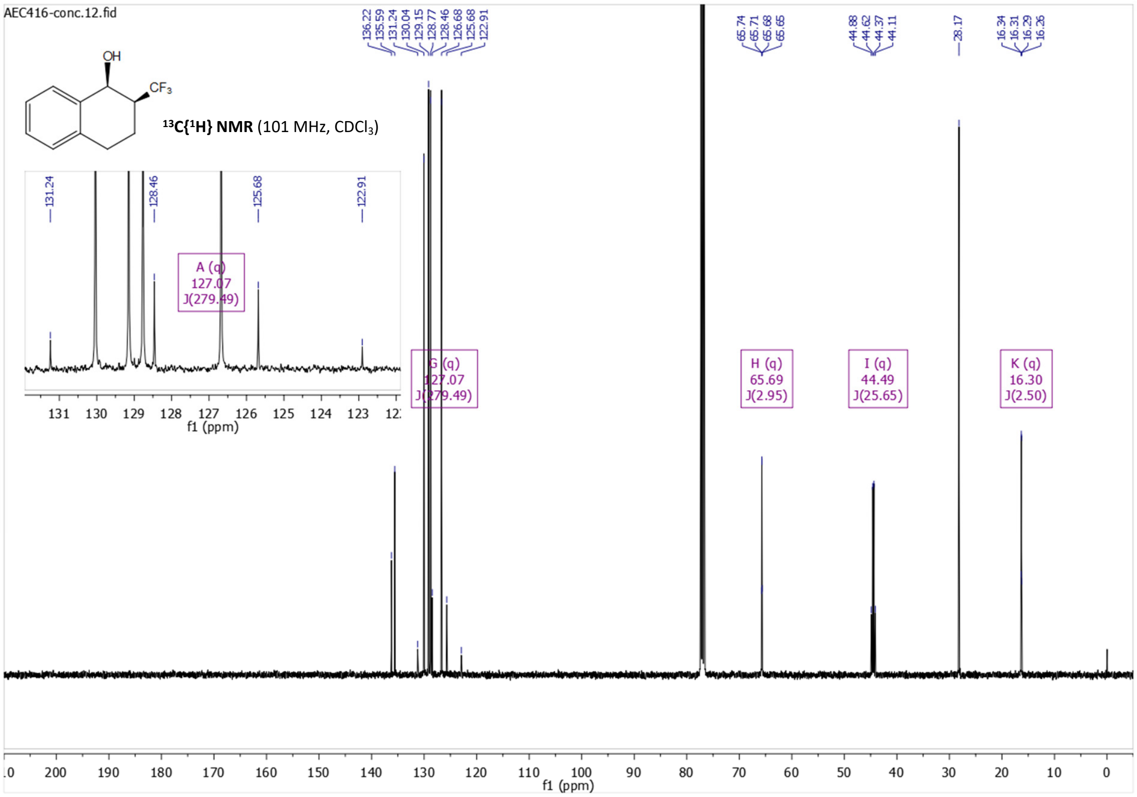

Compound **2j**, ^{1}H NMR:

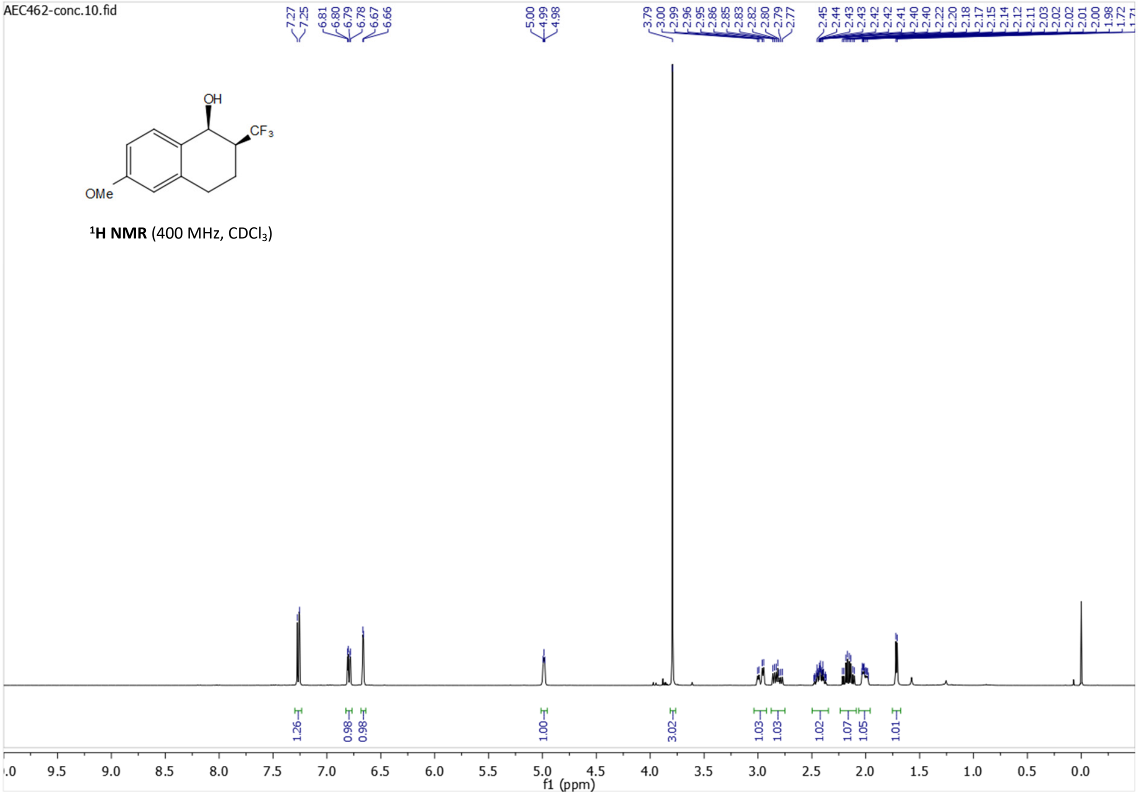

Compound **2j**, ^{13}C{^{1}H} NMR:

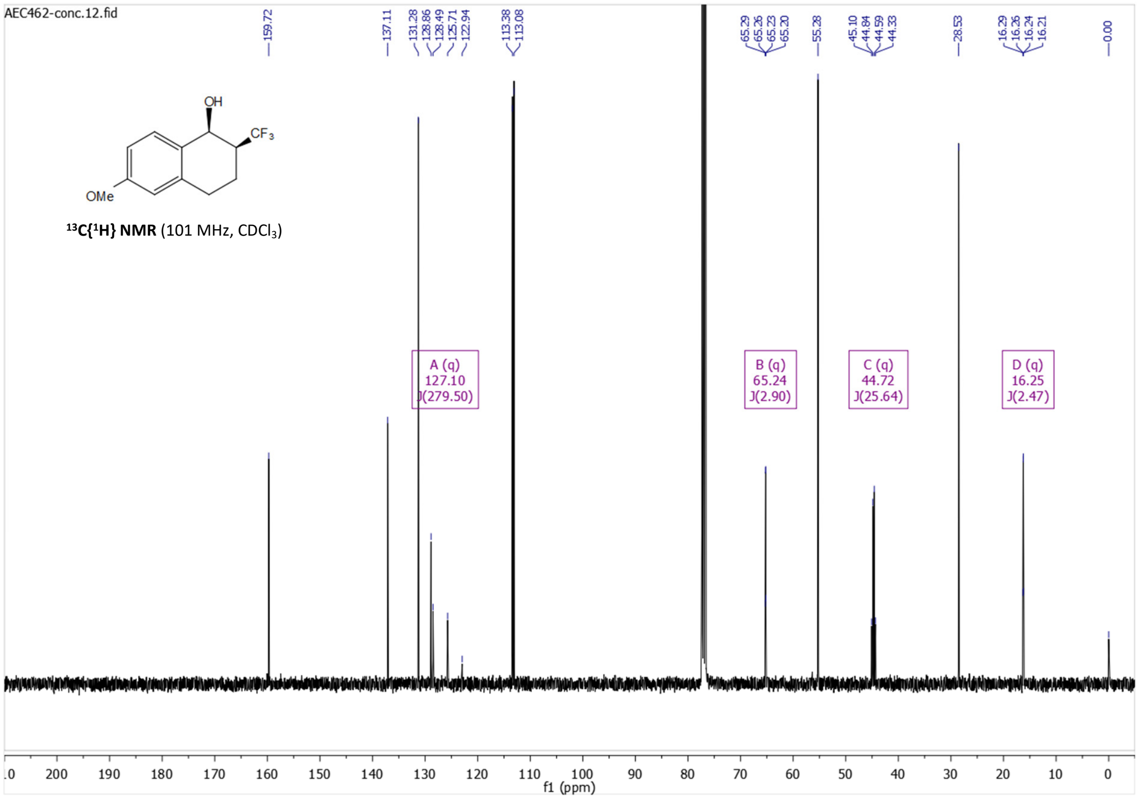

Compound **2k**, ^{1}H NMR:

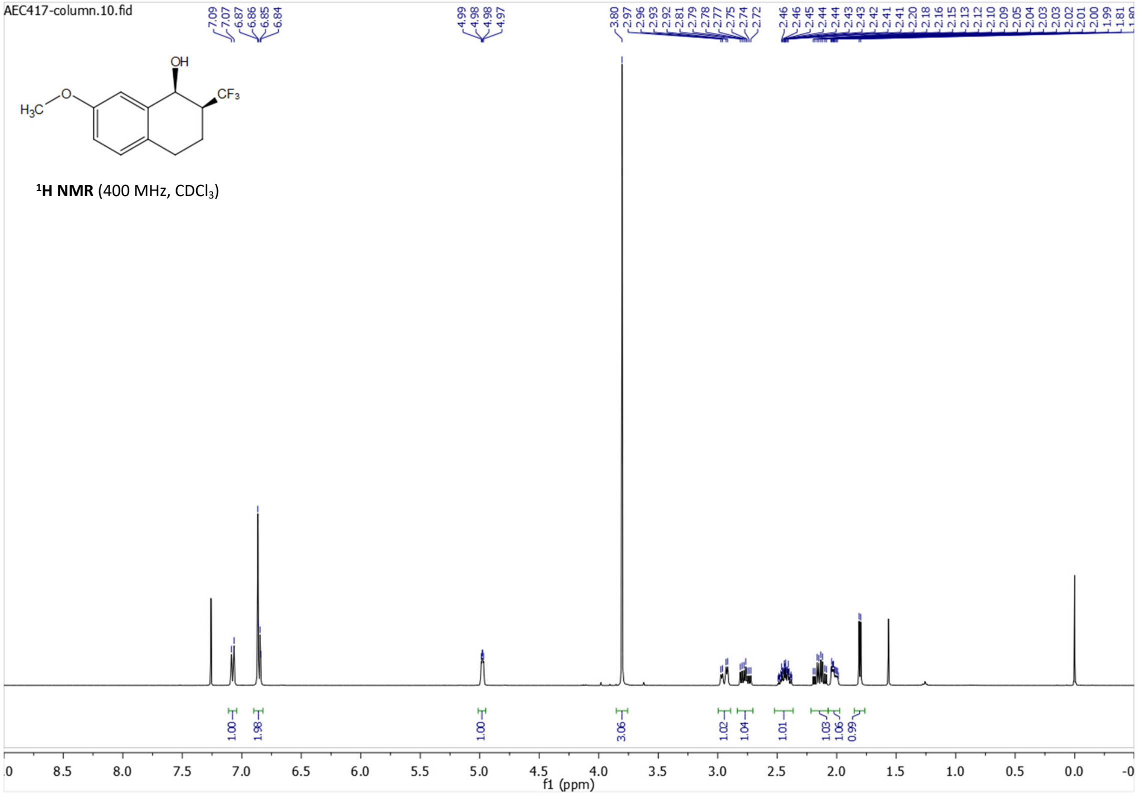

Compound **2k**, $^{13}C\{^{1}H\}$ NMR:

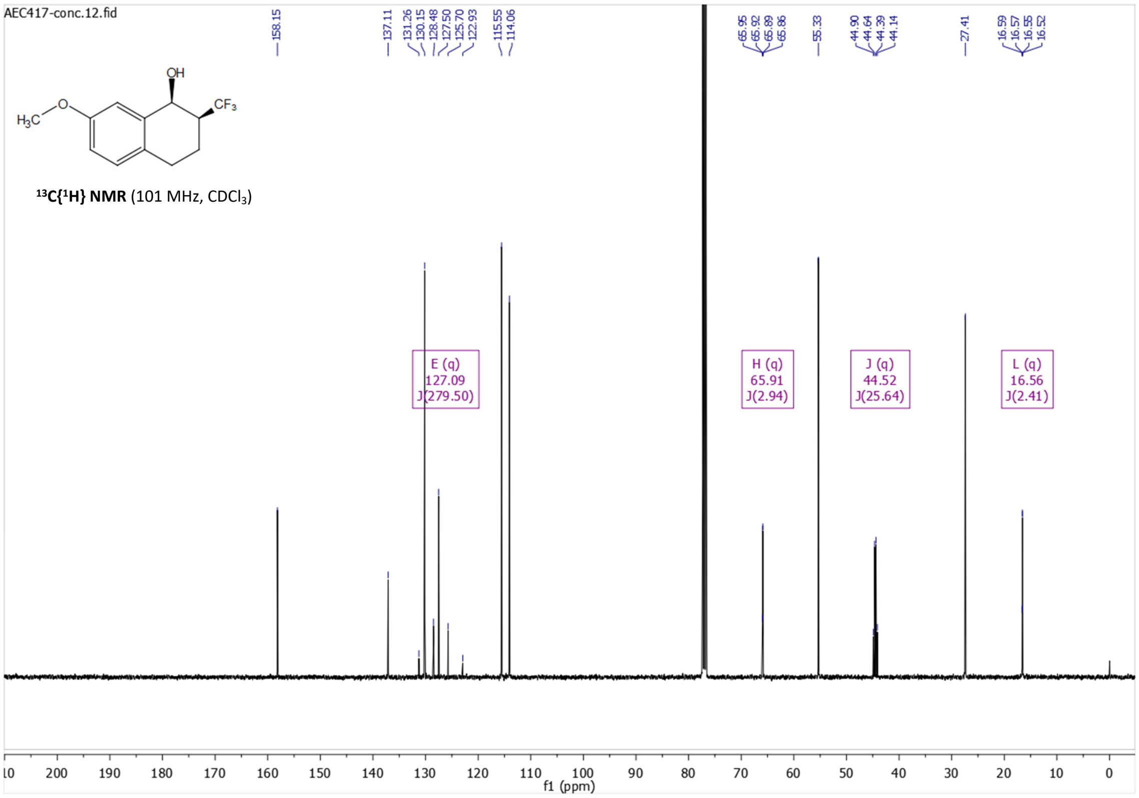

Compound **2l**, ^{1}H NMR:

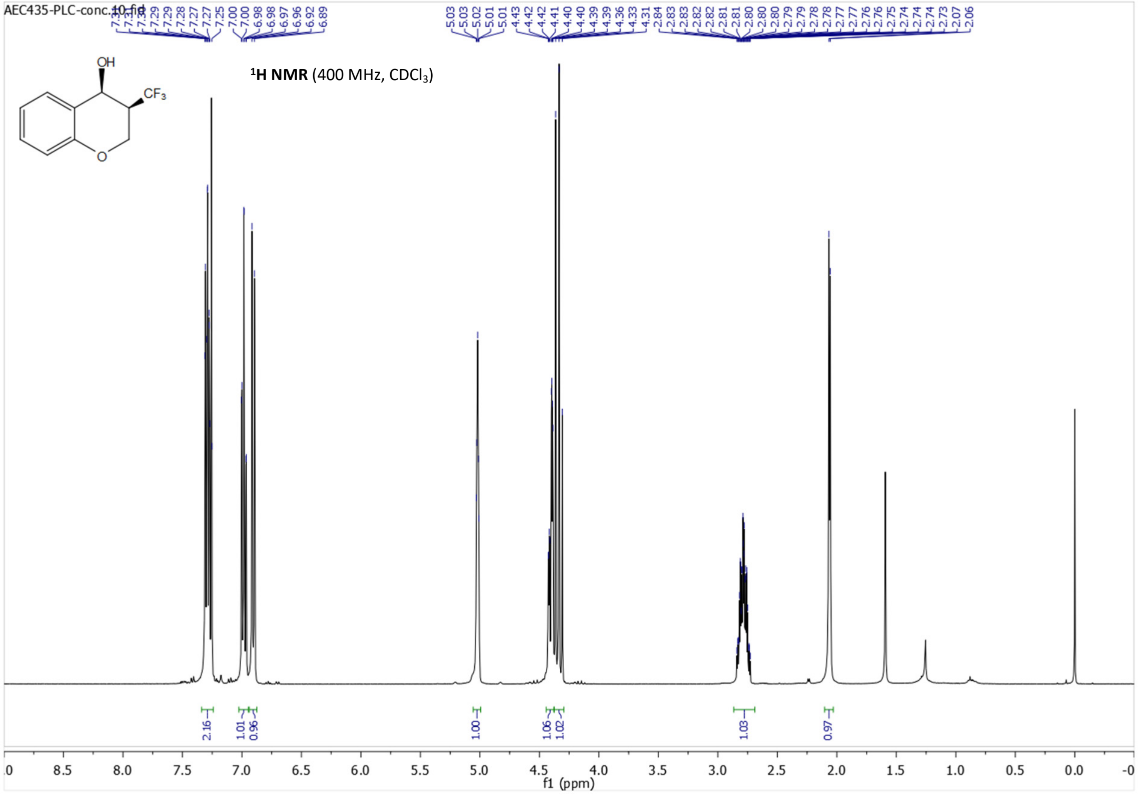

Compound **2l**, ^{13}C{^{1}H} NMR:

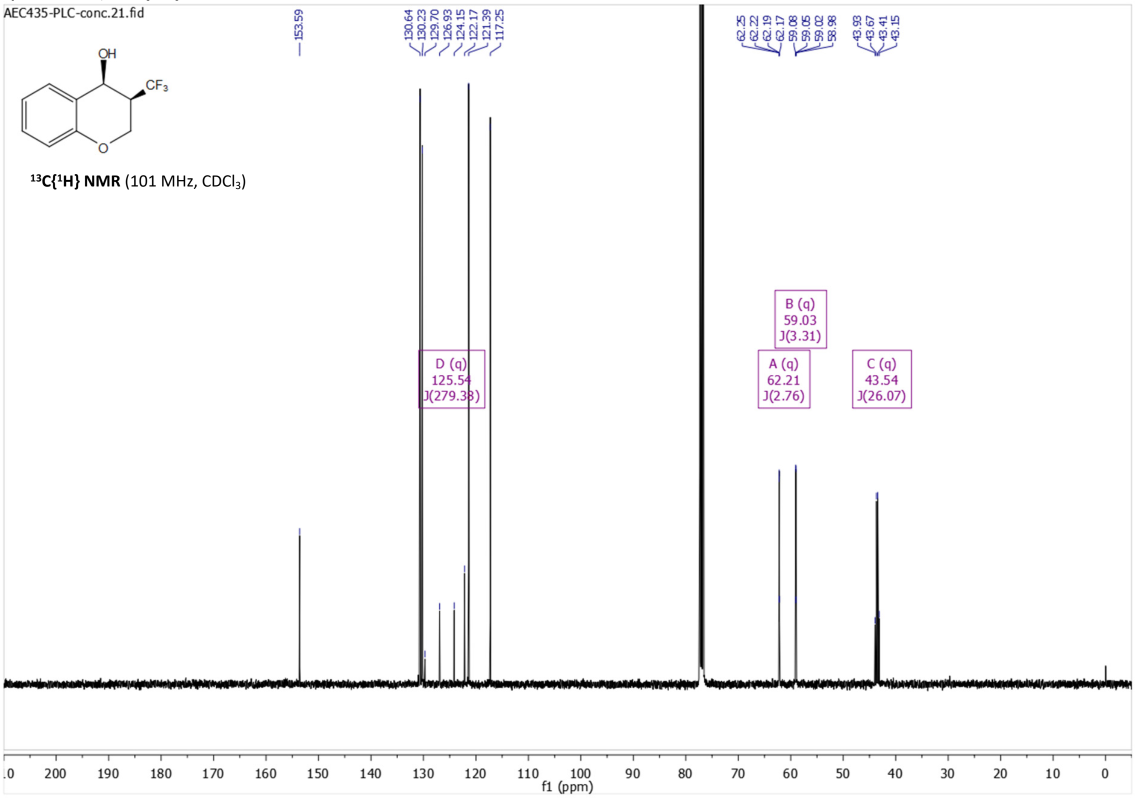

Compound **2m**, ^{1}H NMR:

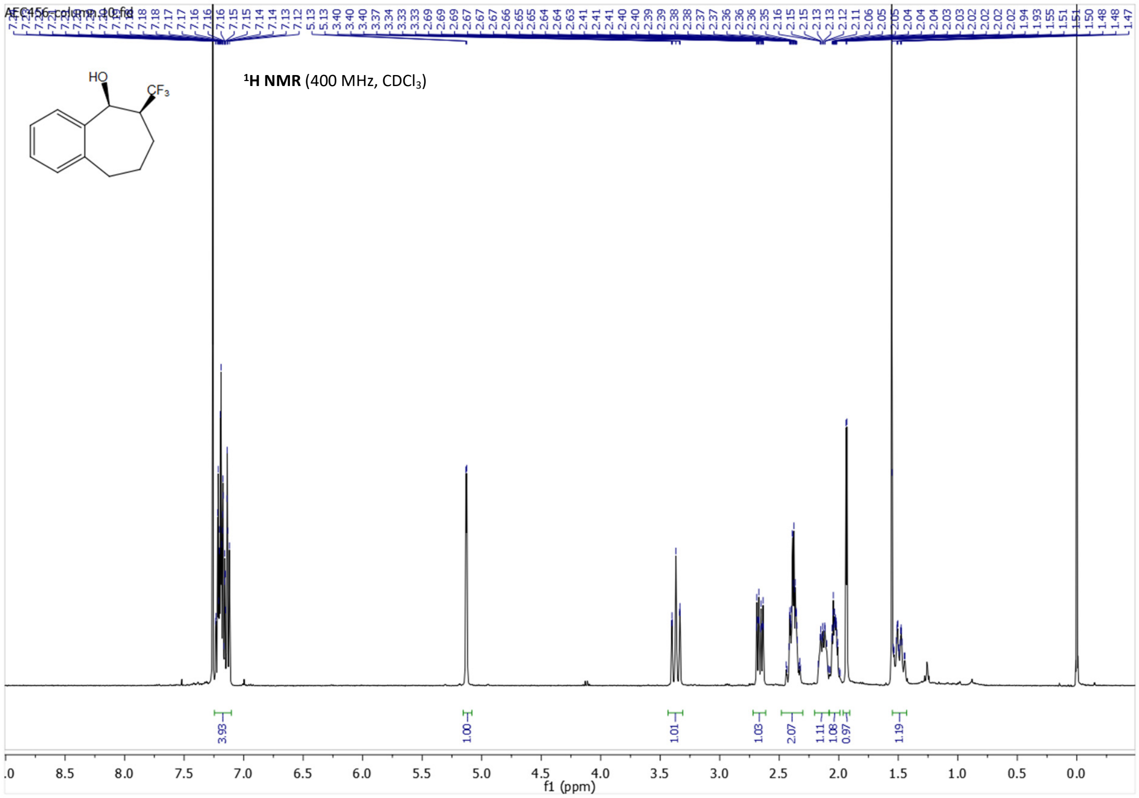

Compound **2m**, ^{13}C{^{1}H} NMR:

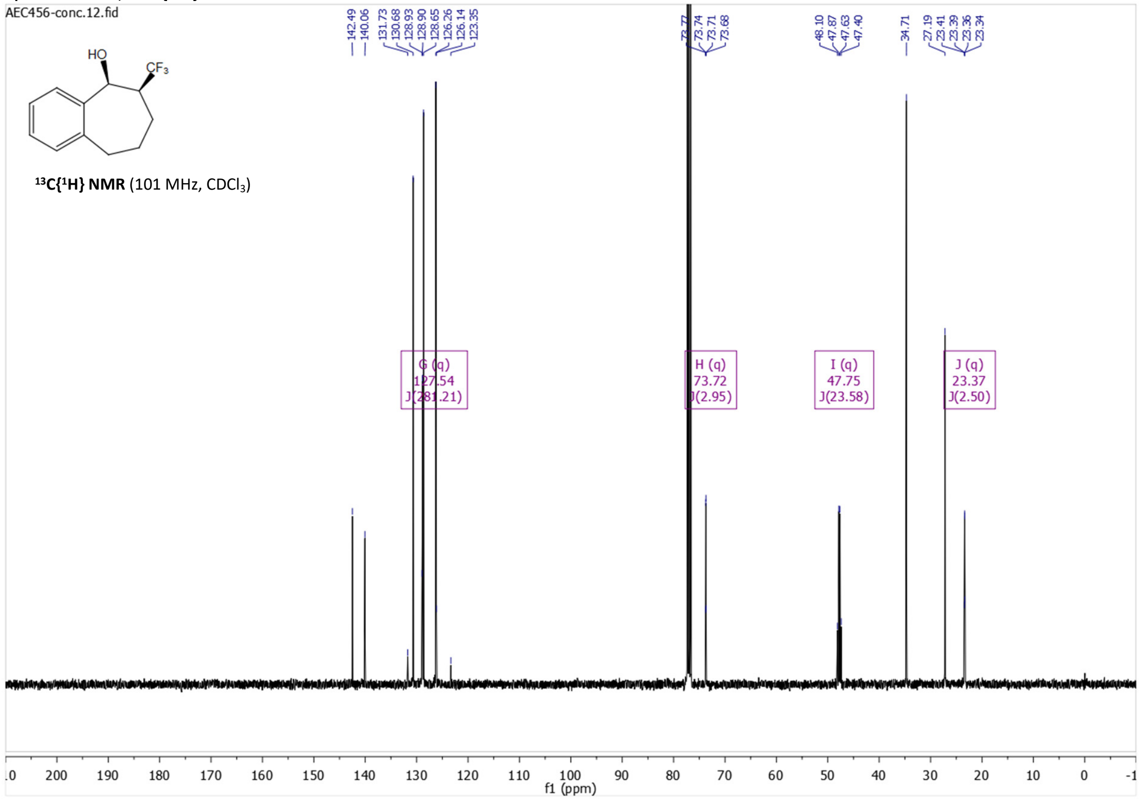

Compound **2n**, ^{1}H NMR:

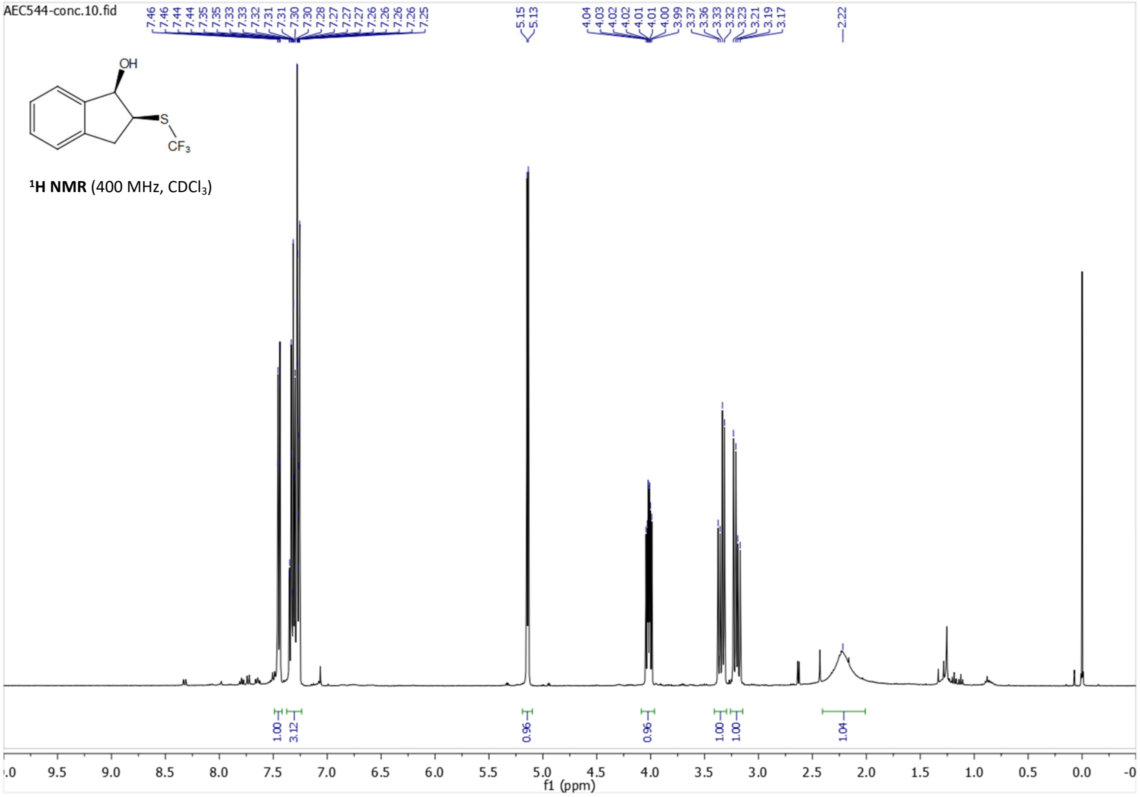

Compound **2n**, ^{13}C{^{1}H} NMR:

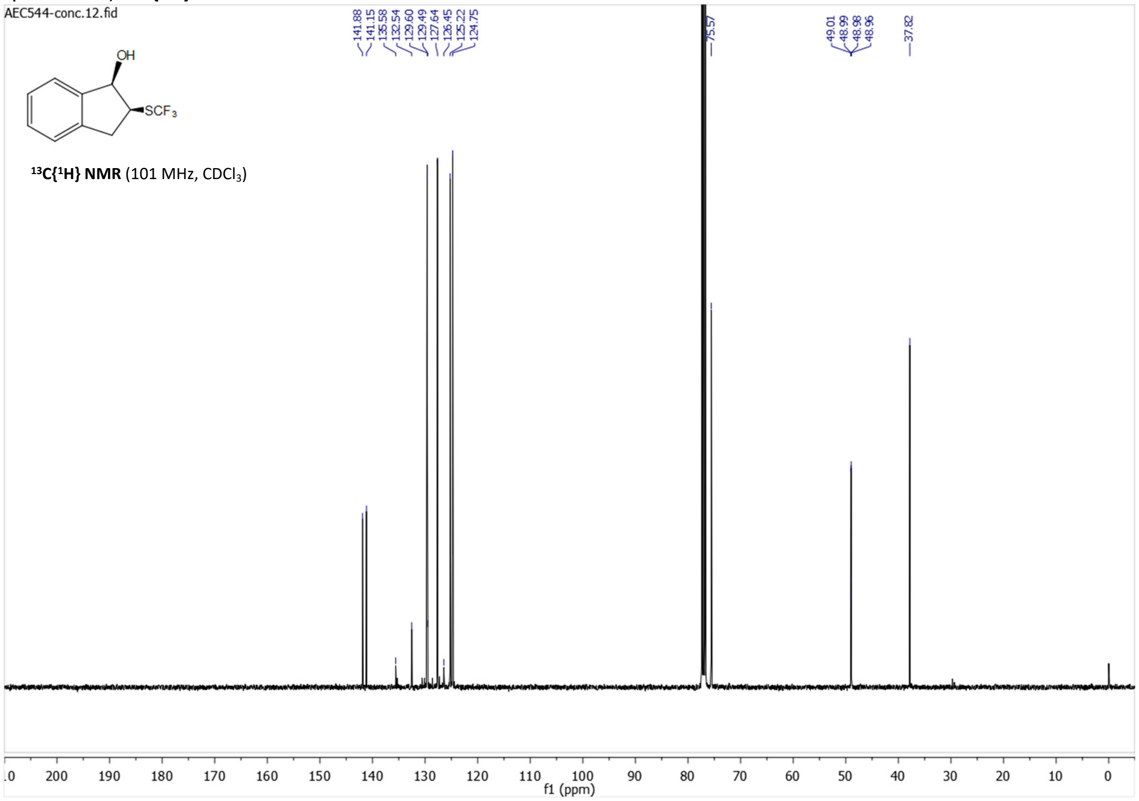

Compound **2o**, ^{1}H NMR:

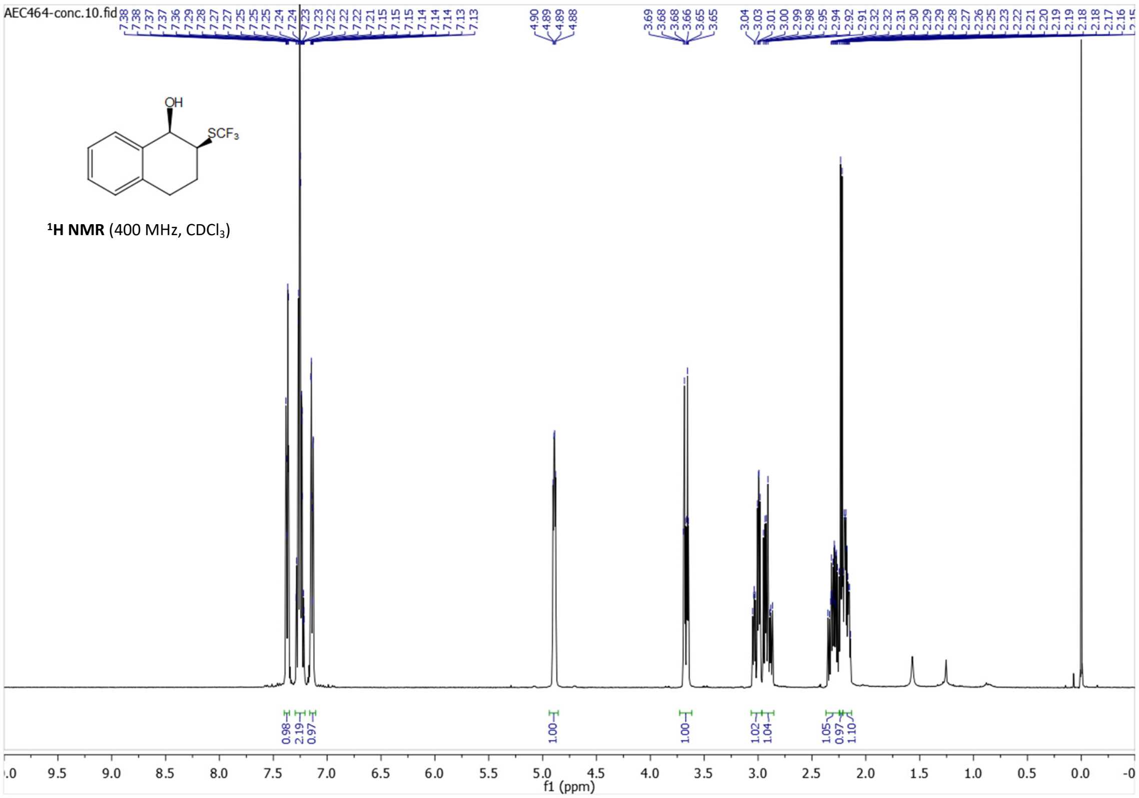

Compound **2o**, $^{13}C\{^{1}H\}$ NMR:

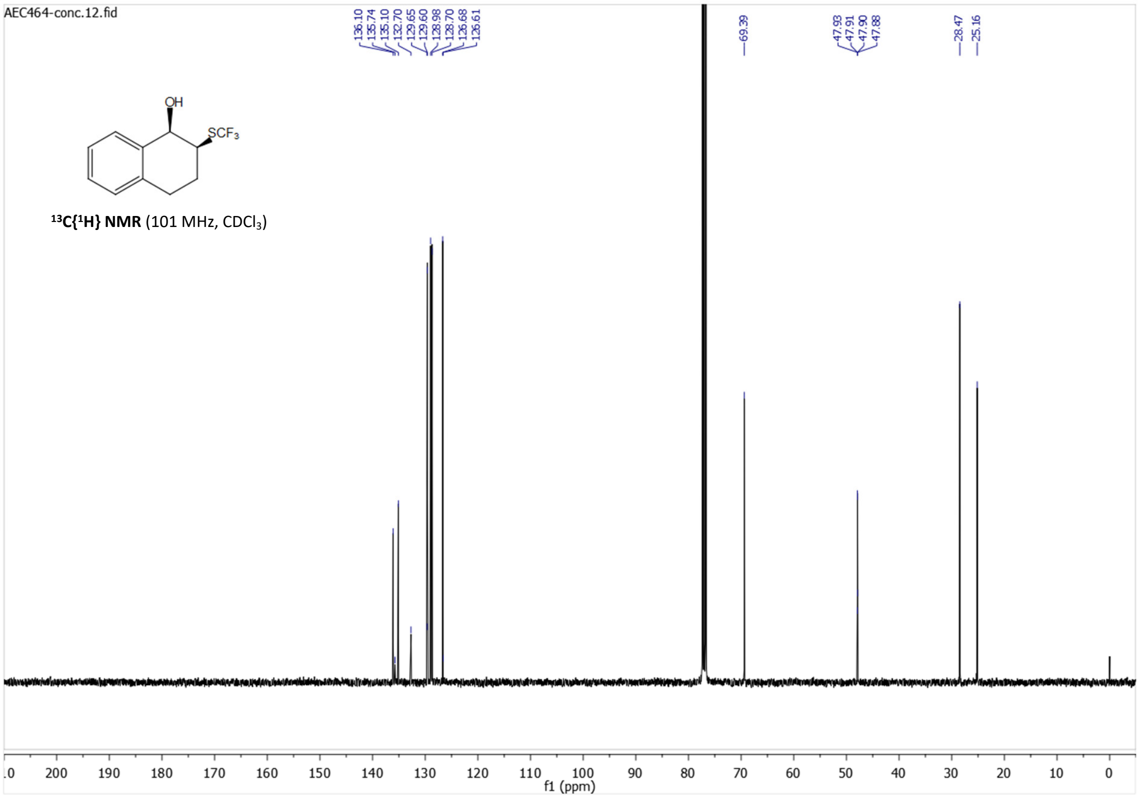

Compound **2p**, ^{1}H NMR:

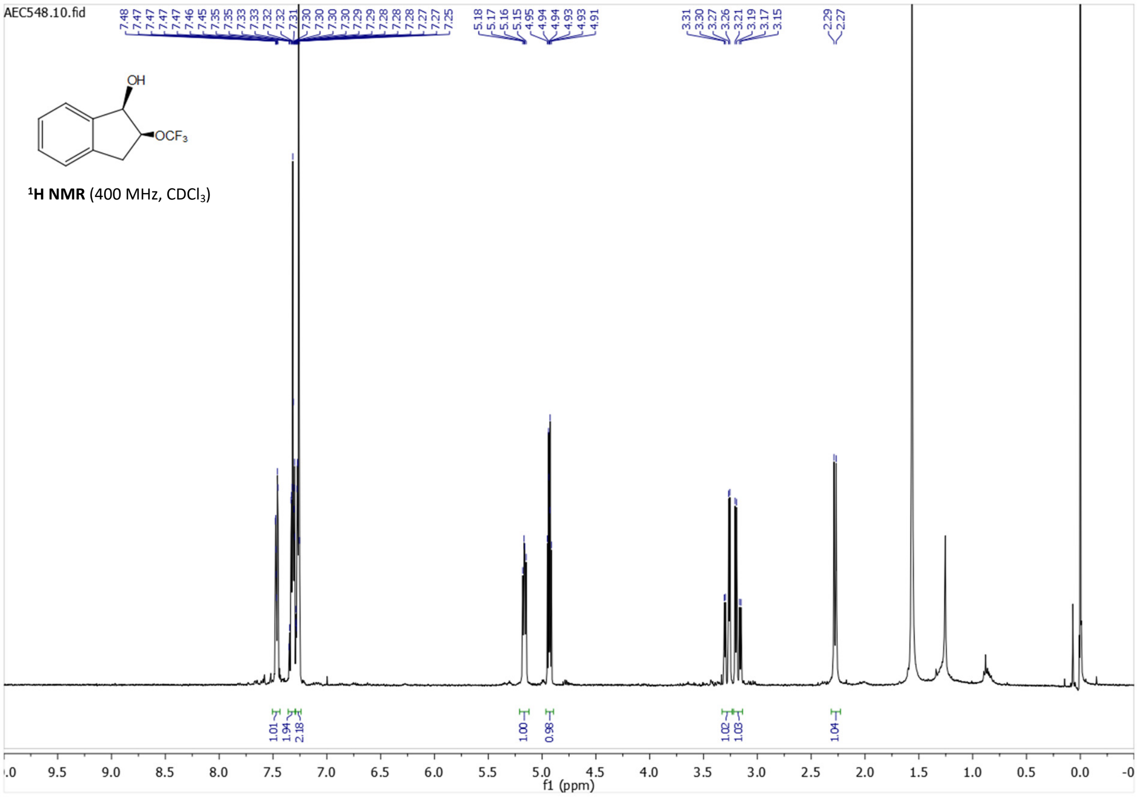

Compound **2p**, $^{13}C\{^{1}H\}$ NMR:

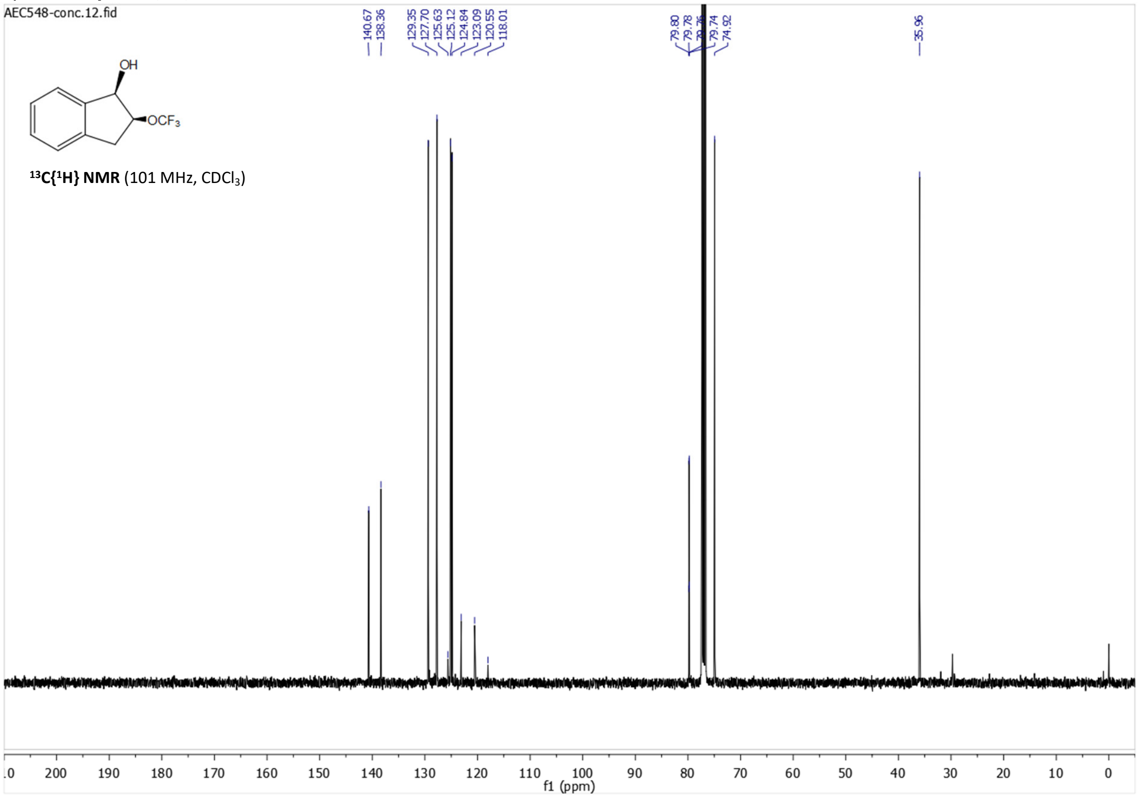

Compound **2q**, ^{1}H NMR:

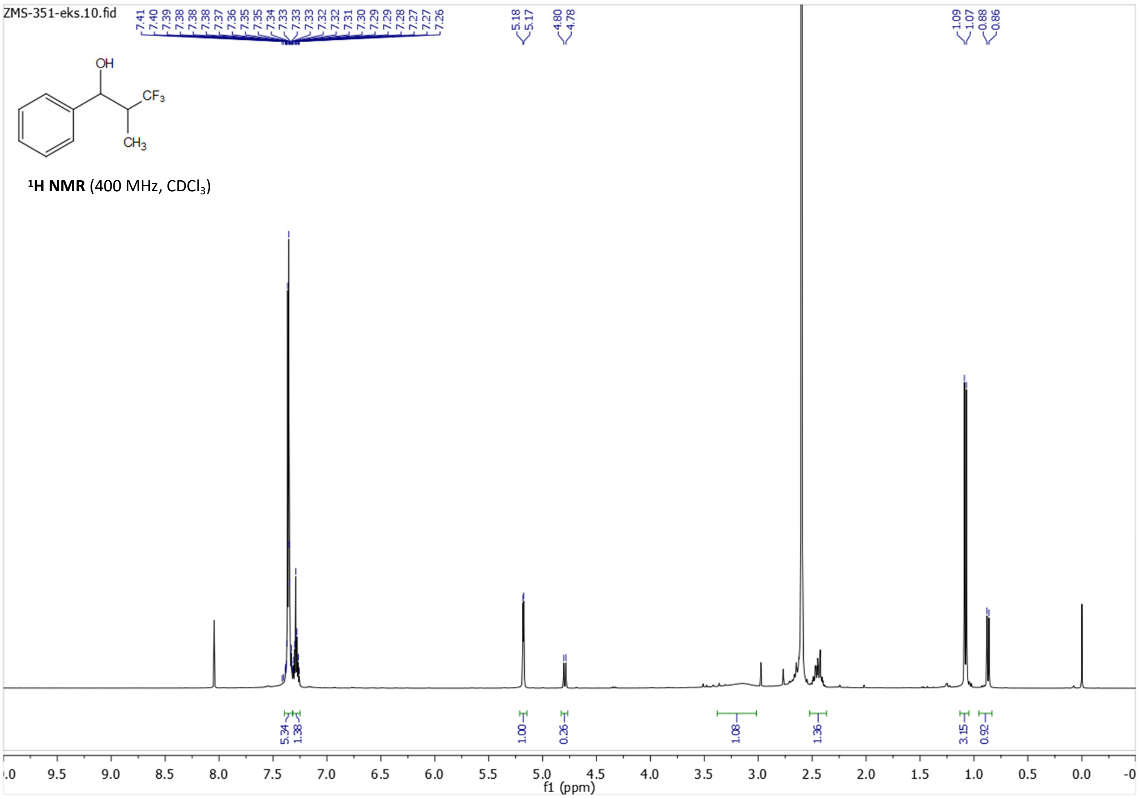

Compound **2r**, ^{1}H NMR:

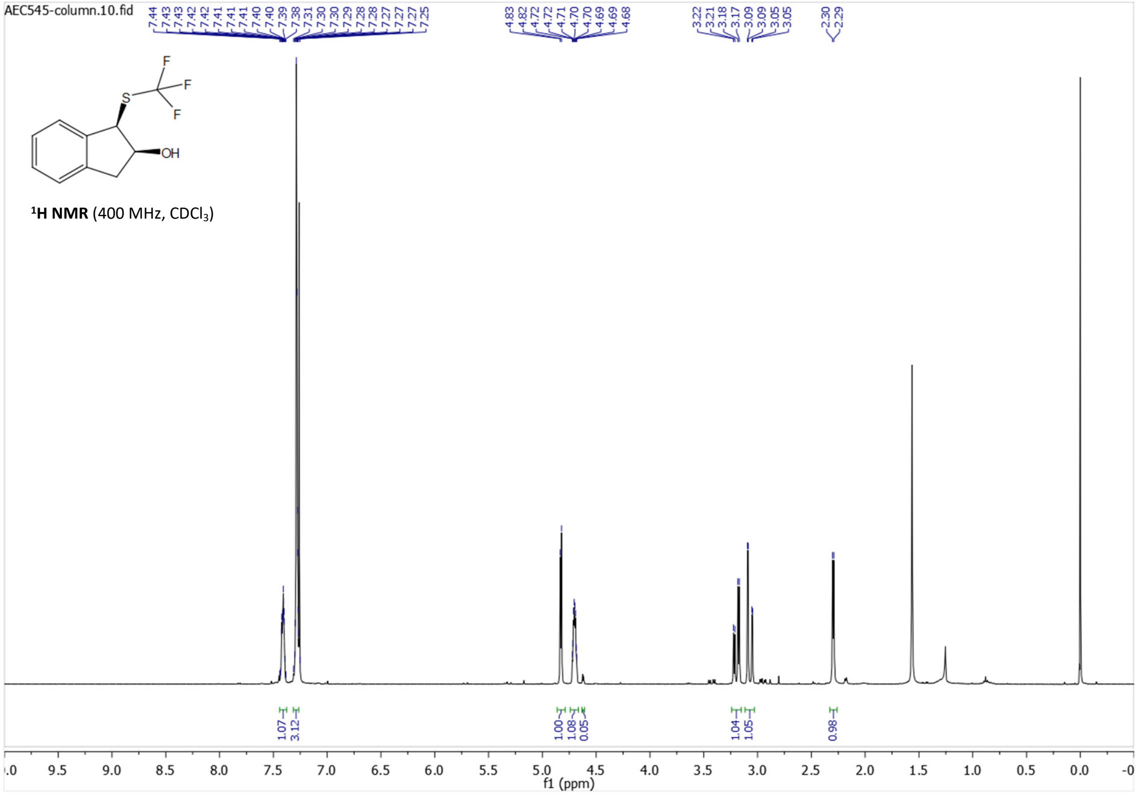

Compound **2r**, $^{13}C\{^{1}H\}$ NMR:

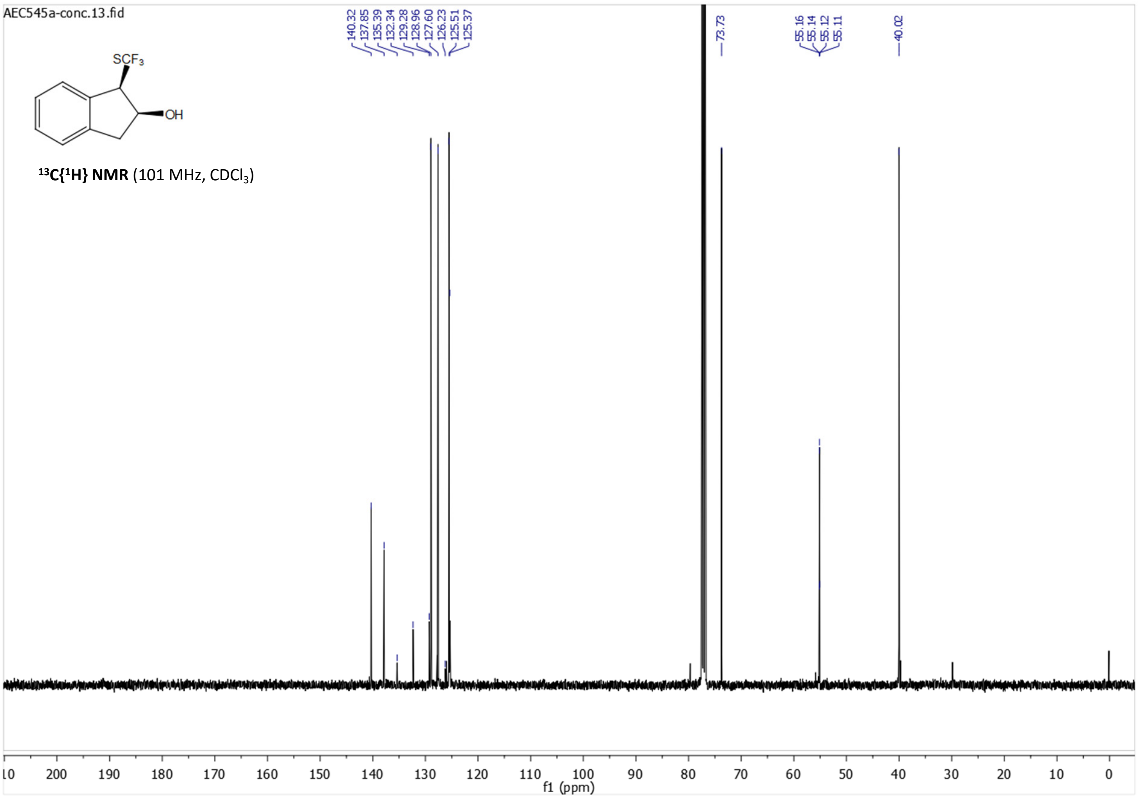

Compound **5**, ^{1}H NMR:

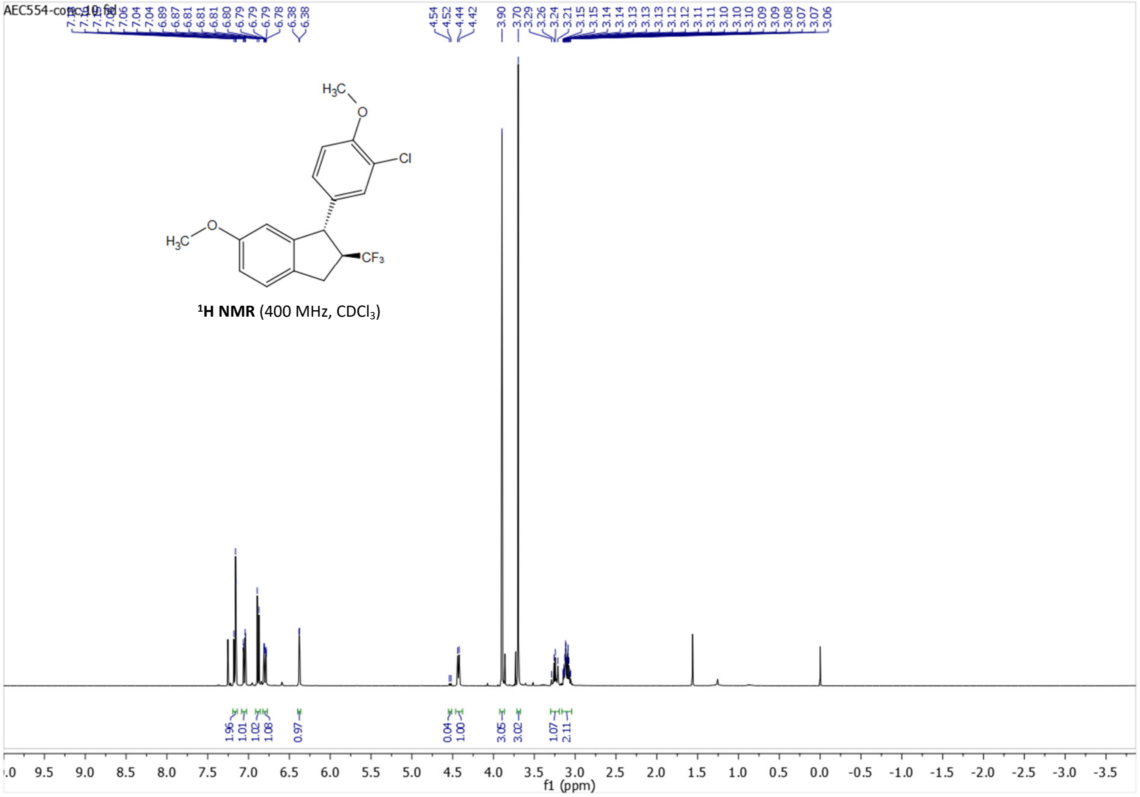

Compound **5**, $^{13}C\{^{1}H\}$ NMR:

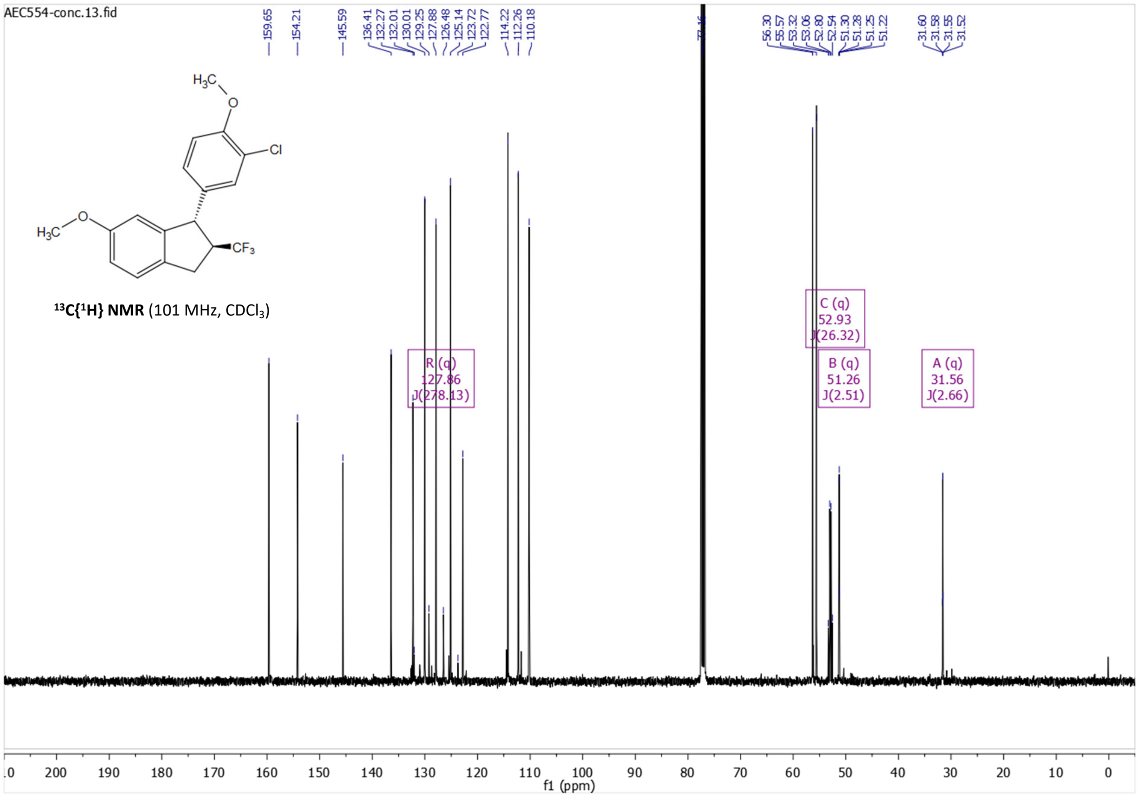

NOESY of Compound **5**:

Interpretation: This ^1H–^1H NOESY NMR experiment unambiguously confirms *trans*-disposition of H^1 and H^2: The nOe is expressed between H^2 and both $H^{3'}$ and $H^{5'}$.

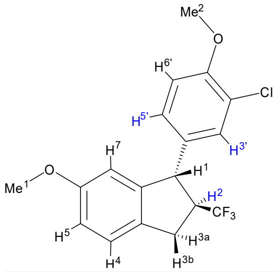

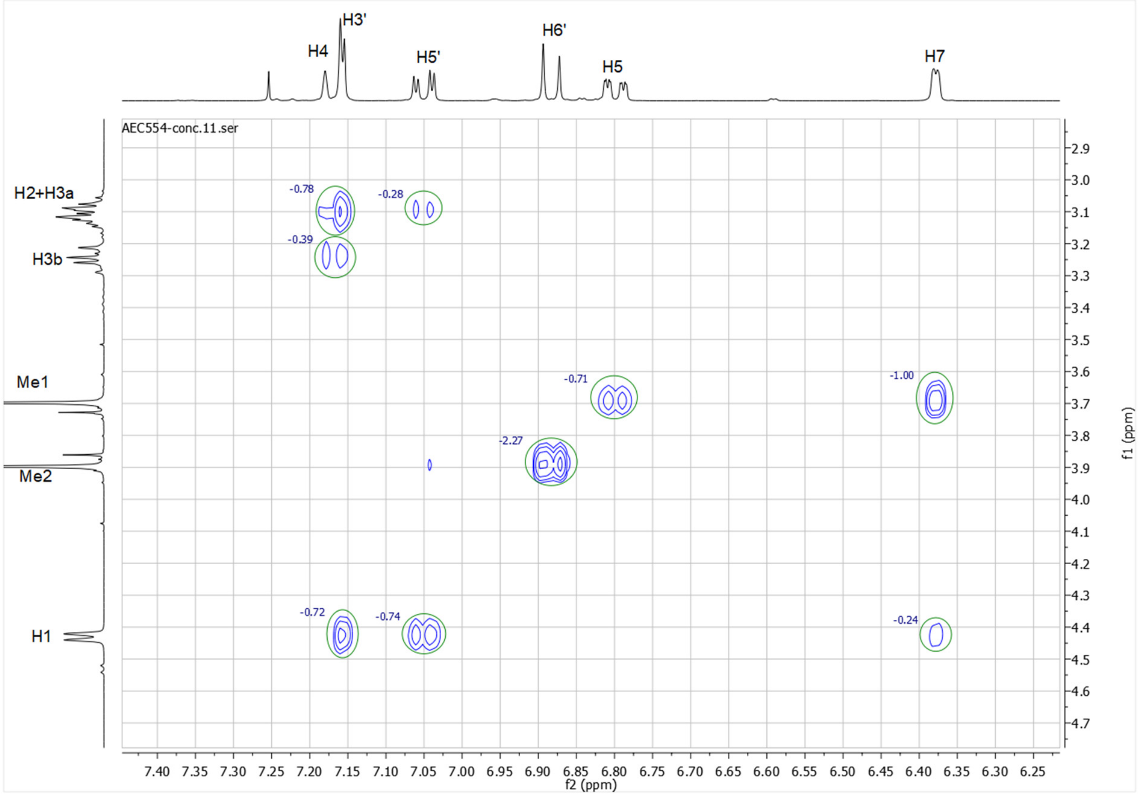

Compound **6**, ^{1}H NMR:

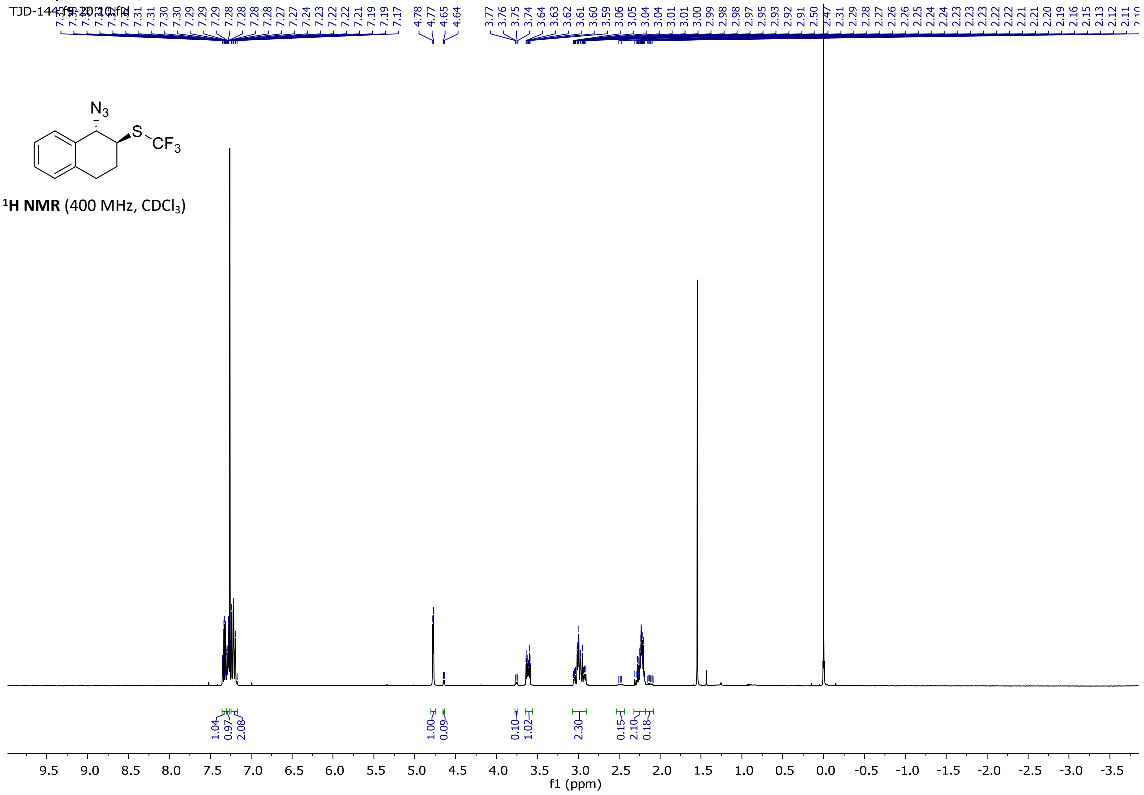

Compound **6**, $^{13}C\{^{1}H\}$ NMR:

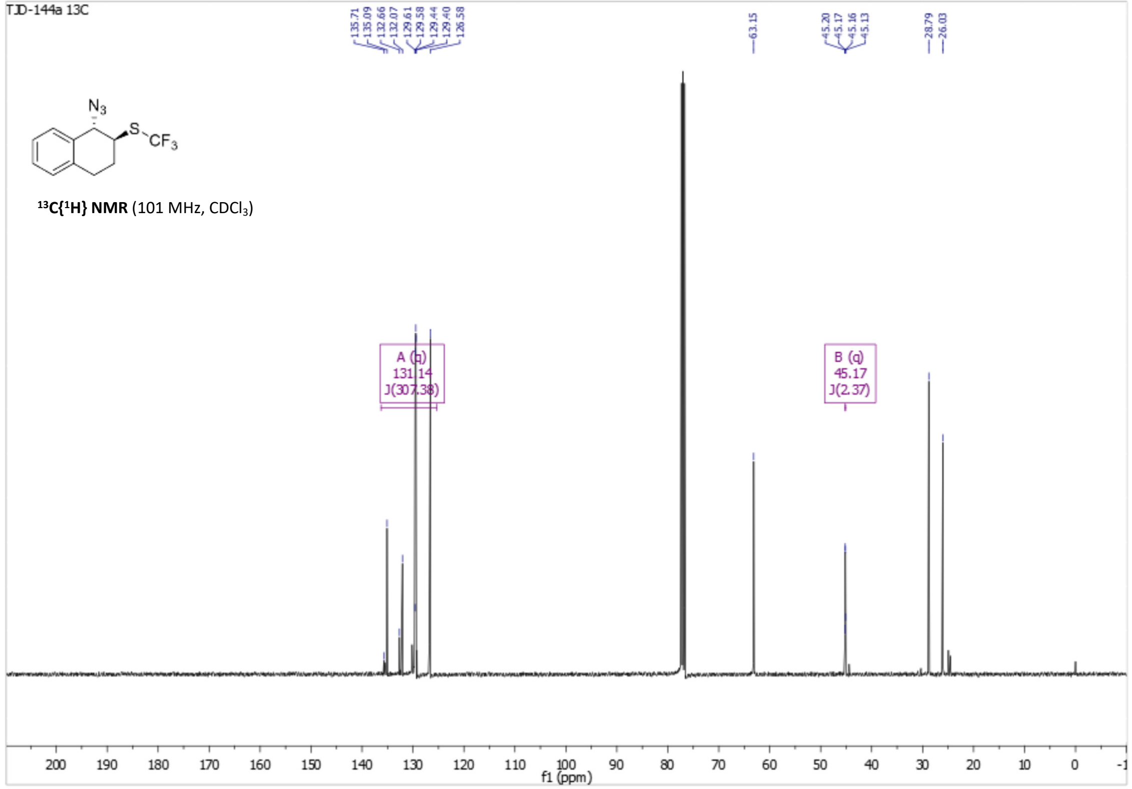

Compound **7**, ^{1}H NMR:

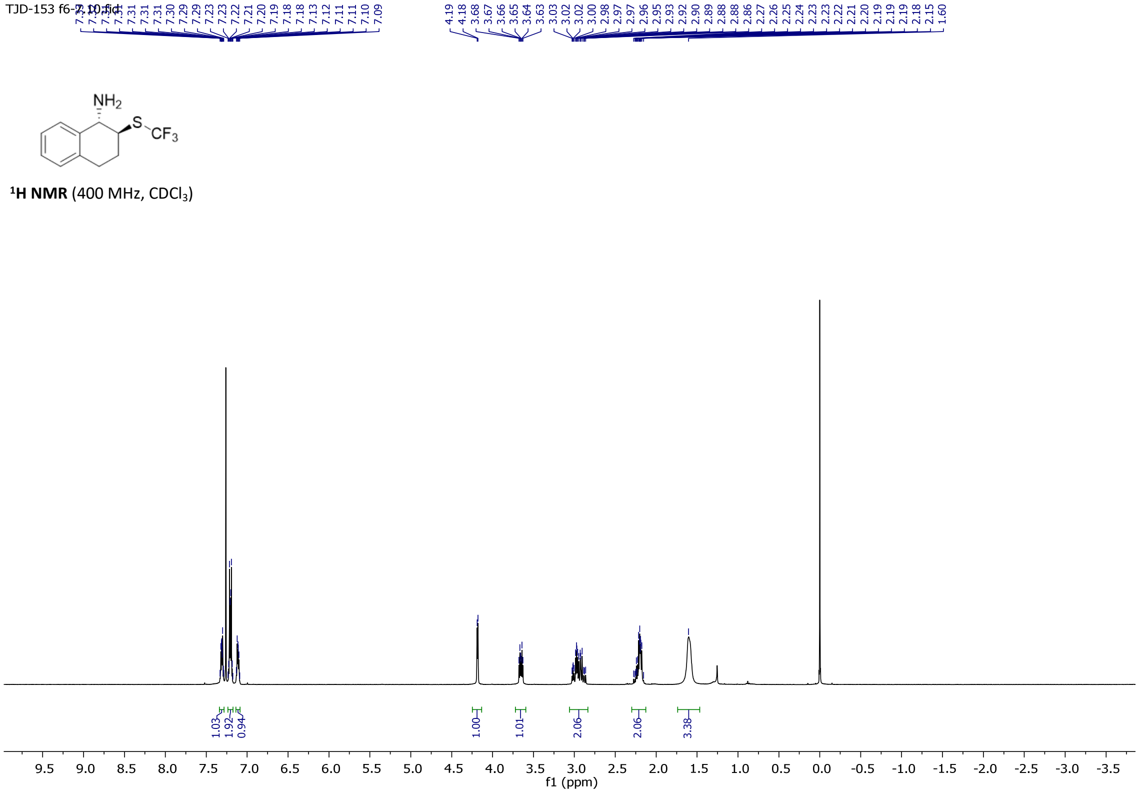

Compound **7**, $^{13}C\{^{1}H\}$ NMR:

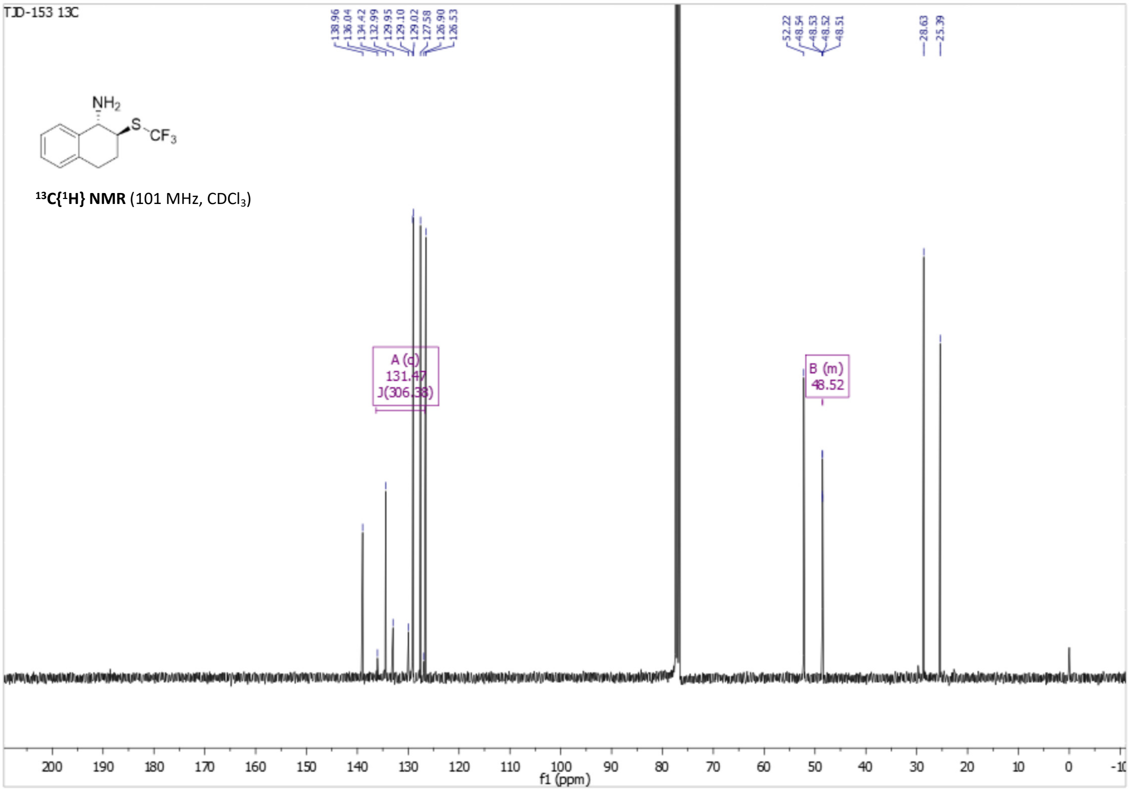

Compound **8**, ^{1}H NMR:

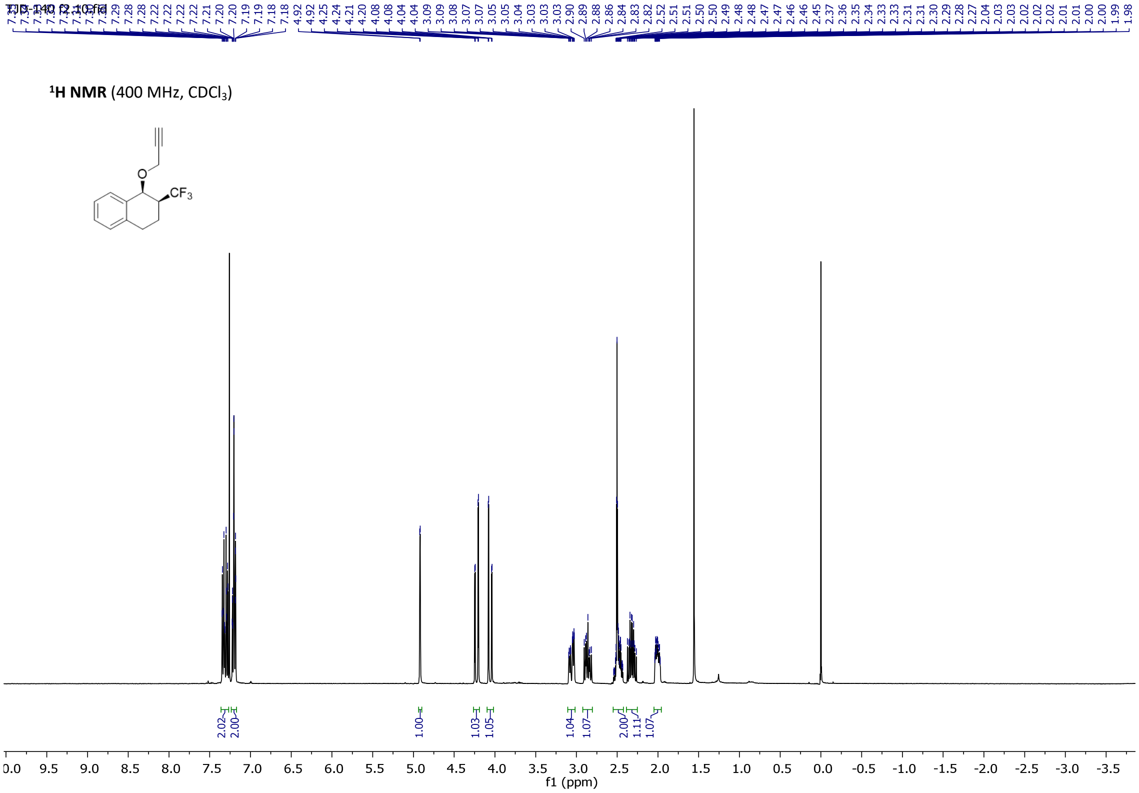

Compound **8**, $^{13}C\{^{1}H\}$ NMR:

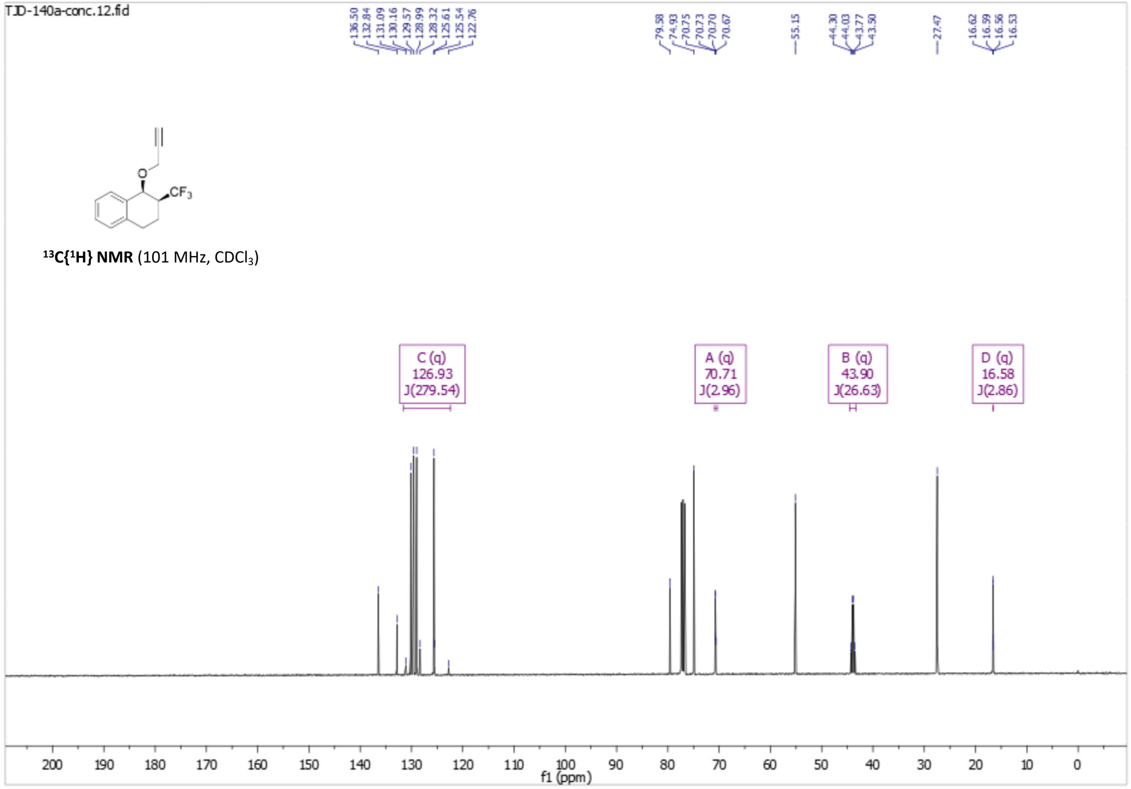

Compound **9**, ^1H NMR:

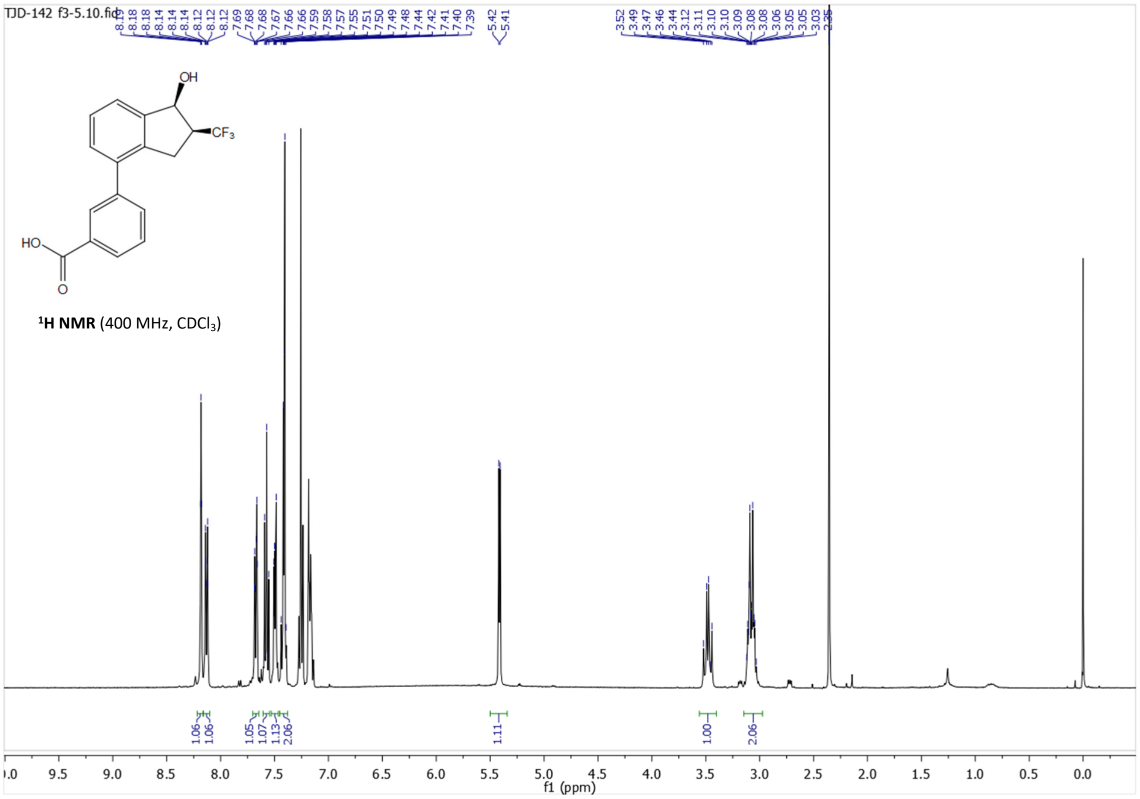

Compound **9**, $^{13}C\{^1H\}$ NMR:

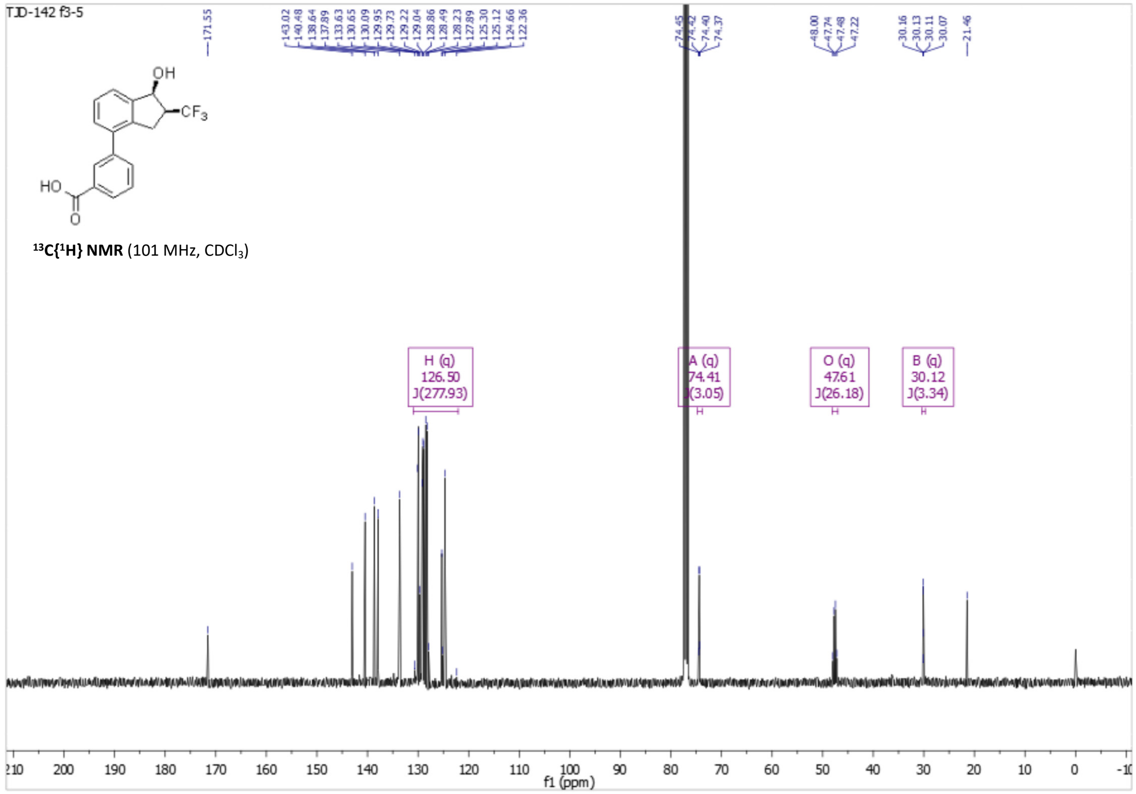

Compound (*S*)-**1a** (Scheme 1), ^{1}H NMR:

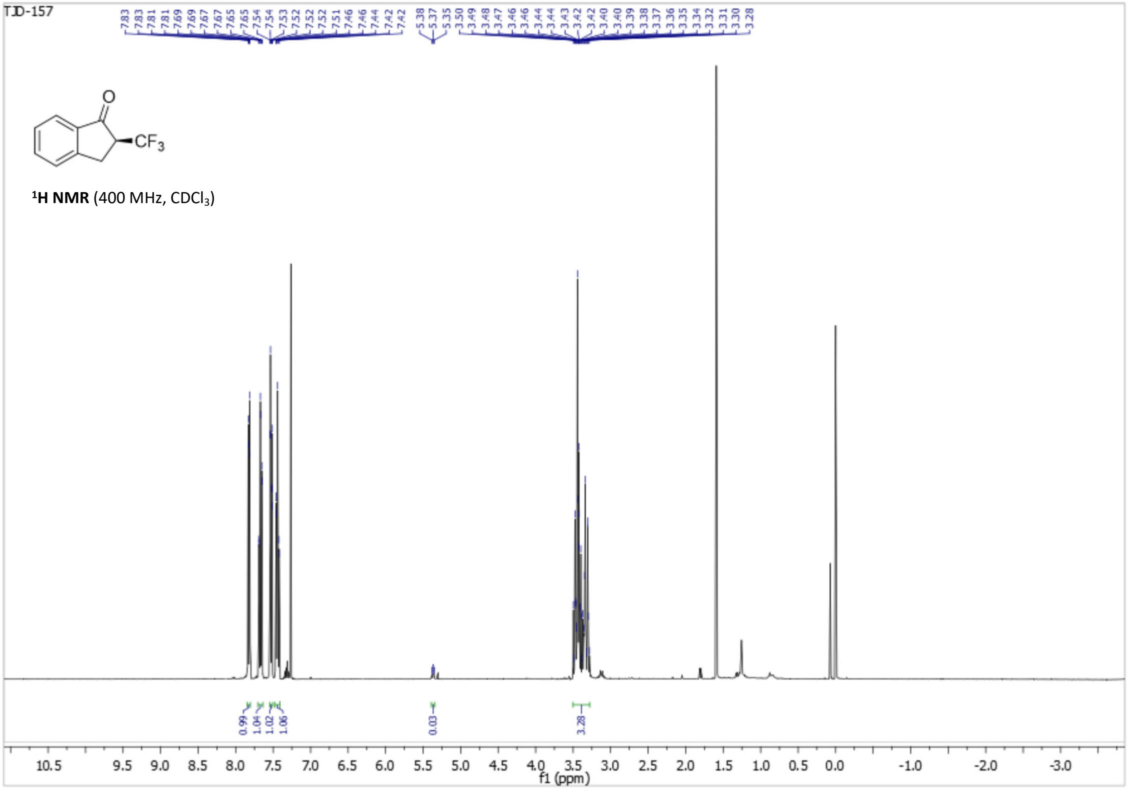

Compound (*S*)-**1c** (Scheme 1), ^{1}H NMR:

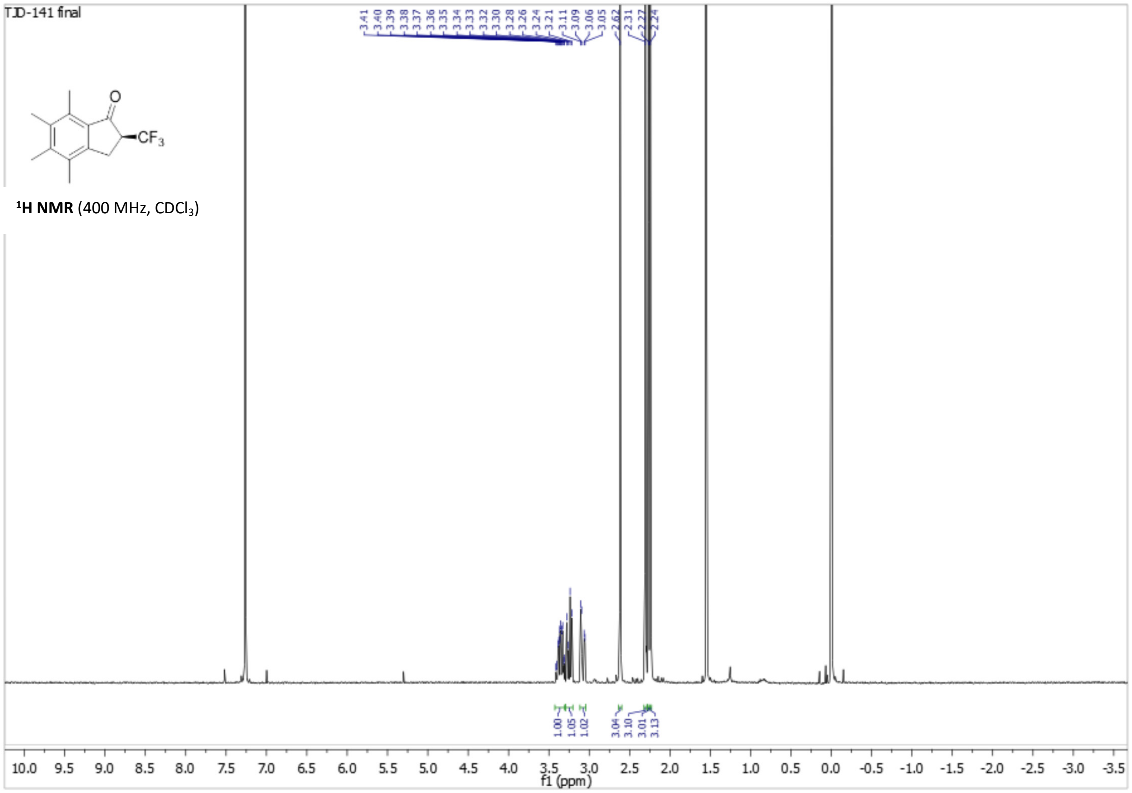

Compound **Boc-10**, ^{1}H NMR:

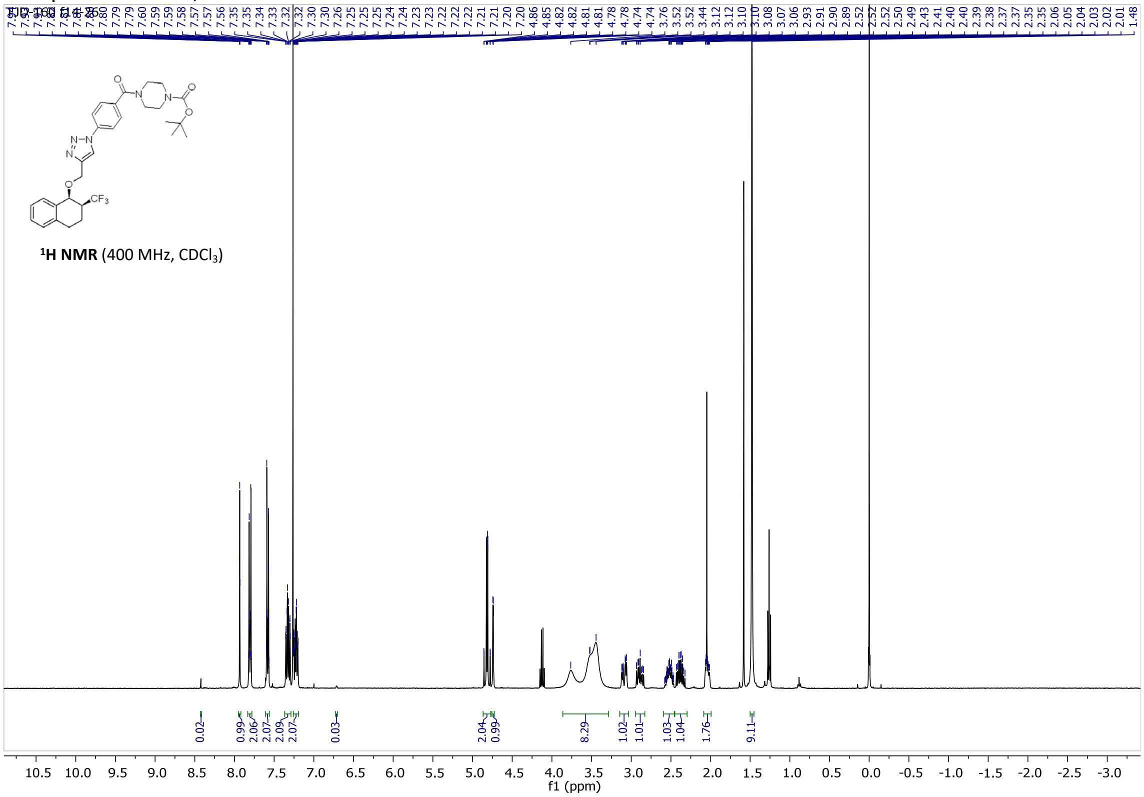

Compound **Boc-10**, $^{13}C\{^{1}H\}$ NMR:

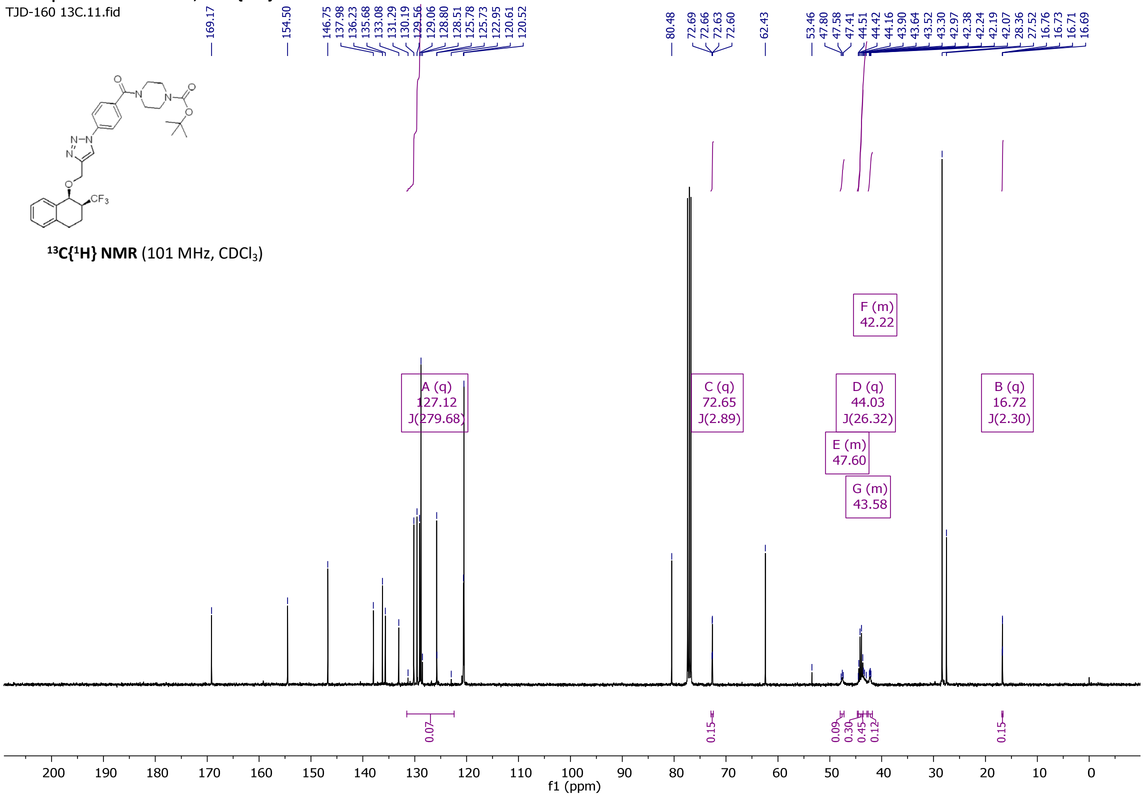

Compound **10**, ^{1}H NMR:

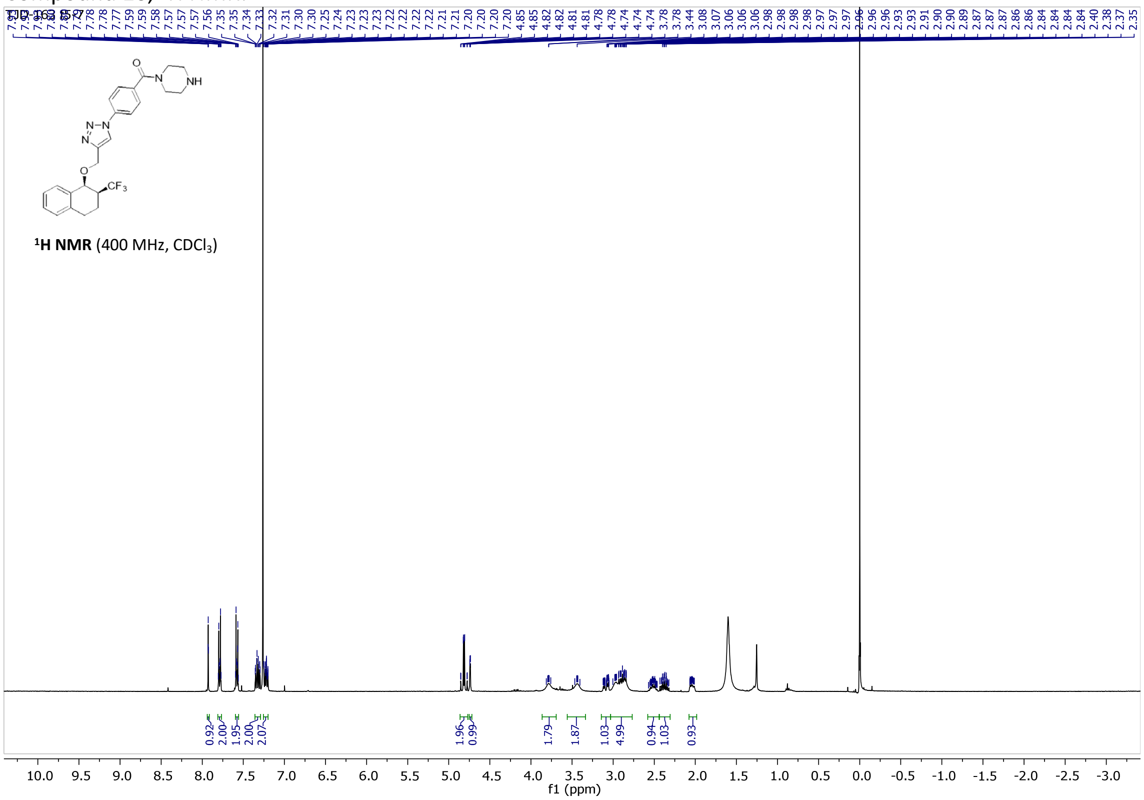

Compound **10**, ^{13}C{^{1}H} NMR:

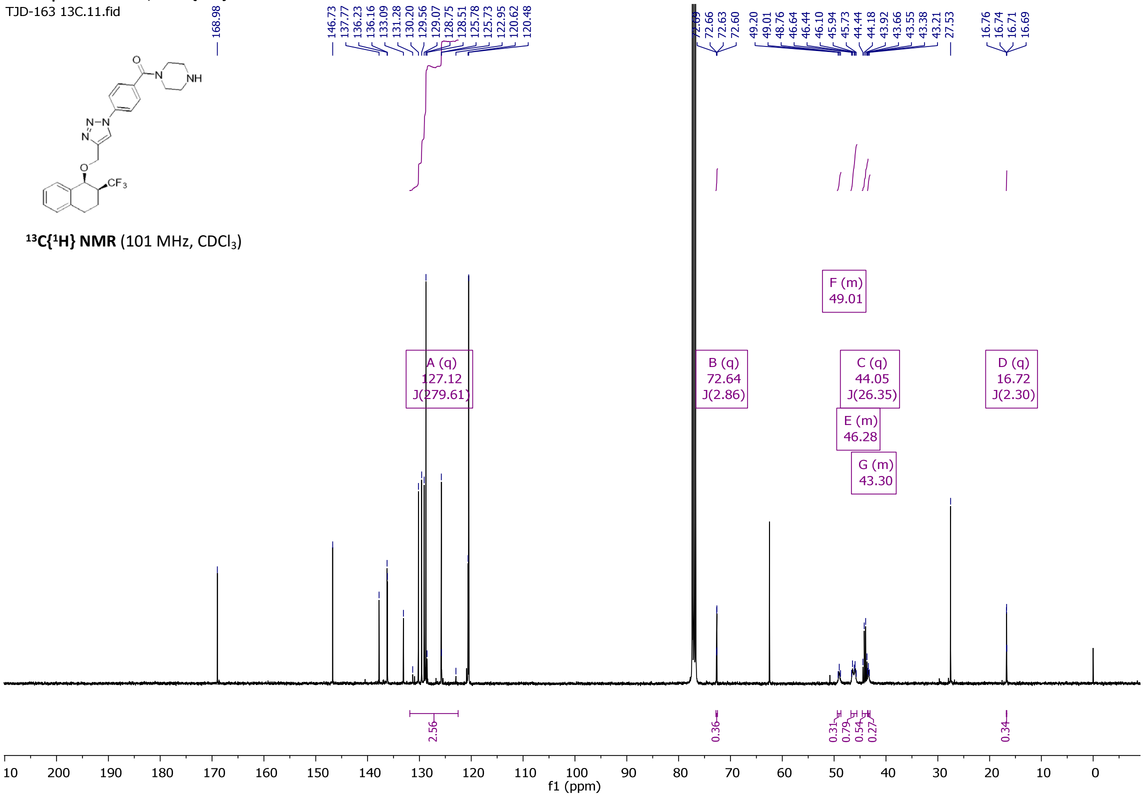

Computational analysis

All calculations were performed with Gaussian 16 (rev. C01) software.[17] Hybrid M06-2X[18] (with global 54% exchange) functional parametrized via D3 dispersion model[19] was employed. To introduce non-specific solvent effects of chlorobenzene in the geometry optimization steps, the Solvation Model based on Density (SMD),[20] a popular version of a polarizable continuum model, was used. All the geometries were optimized with the def2-svp and further refined with the def2-qzvp basis, respectively. The standard reaction Gibbs energies (1M, 298K) were calculated by combining the single-point def2-svp//def2-qzvp energies with the thermal corrections from frequency calculations under def2-SVP level and adjusted by 0.00301 Hartree. All the calculations were carried out on the conformers of the ruthenium complexes having λ-configured five-membered N–N ring with C–H hydrogen atoms located at the axial positions as found in X-ray molecular structures of (*S,S*)-**C2**.[21] Transition states were optimized by using Berny algorithm based on initial guess obtained from a constrained potential energy surface scan. Molecular graphics images were produced using the UCSF Chimera package.[22]

[17] Gaussian 16, Revision C.01, M. J. Frisch, G. W. Trucks, H. B. Schlegel, G. E. Scuseria, M. A. Robb, J. R. Cheeseman, G. Scalmani, V. Barone, G. A. Petersson, H. Nakatsuji, X. Li, M. Caricato, A. V. Marenich, J. Bloino, B. G. Janesko, R. Gomperts, B. Mennucci, H. P. Hratchian, J. V. Ortiz, A. F. Izmaylov, J. L. Sonnenberg, D. Williams-Young, F. Ding, F. Lipparini, F. Egidi, J. Goings, B. Peng, A. Petrone, T. Henderson, D. Ranasinghe, V. G. Zakrzewski, J. Gao, N. Rega, G. Zheng, W. Liang, M. Hada, M. Ehara, K. Toyota, R. Fukuda, J. Hasegawa, M. Ishida, T. Nakajima, Y. Honda, O. Kitao, H. Nakai, T. Vreven, K. Throssell, J. A. Montgomery, Jr., J. E. Peralta, F. Ogliaro, M. J. Bearpark, J. J. Heyd, E. N. Brothers, K. N. Kudin, V. N. Staroverov, T. A. Keith, R. Kobayashi, J. Normand, K. Raghavachari, A. P. Rendell, J. C. Burant, S. S. Iyengar, J. Tomasi, M. Cossi, J. M. Millam, M. Klene, C. Adamo, R. Cammi, J. W. Ochterski, R. L. Martin, K. Morokuma, O. Farkas, J. B. Foresman, and D. J. Fox, Gaussian, Inc., Wallingford CT, 2016.

[18] Zhao, Y.; Truhlar, D. G., *Theor. Chem. Acc.* **2008,** *120*, 215–241.

[19] Grimme, S.; Antony, J.; Ehrlich, S.; Krieg, H. H-Pu. *J. Chem. Phys.* **2010,** *132*, 154104.

[20] Marenich, A. V.; Cramer, C. J.; Truhlar, D. G. *J. Phys. Chem. B* **2009**, *113*, 6378–6396.

[21] Hayes, A. M.; Morris, D. J.; Clarkson, G. J.; Wills, M. *J. Am. Chem. Soc.* **2005**, *127*, 7318–7319.

[22] Pettersen, E. F.; Goddard, T. D.; Huang, C. C.; Couch, G. S.; Greenblatt, D. M.; Meng, E. C.; Ferrin, T. E. *J. Comp. Chem.* **2004**, *25*, 1605–1612.

Figure S1. Computed relative Free energy Profiles (1M, kcal·mol^{-1}) for the direct (top), enolate (middle) and enolate-anion (bottom) **1a**-epimerization pathways. For the enolate and enolate-anion pathways, the energy is relative to $HCO_2H \cdot HCO_2 \cdot HNEt_3$ optimized adduct.

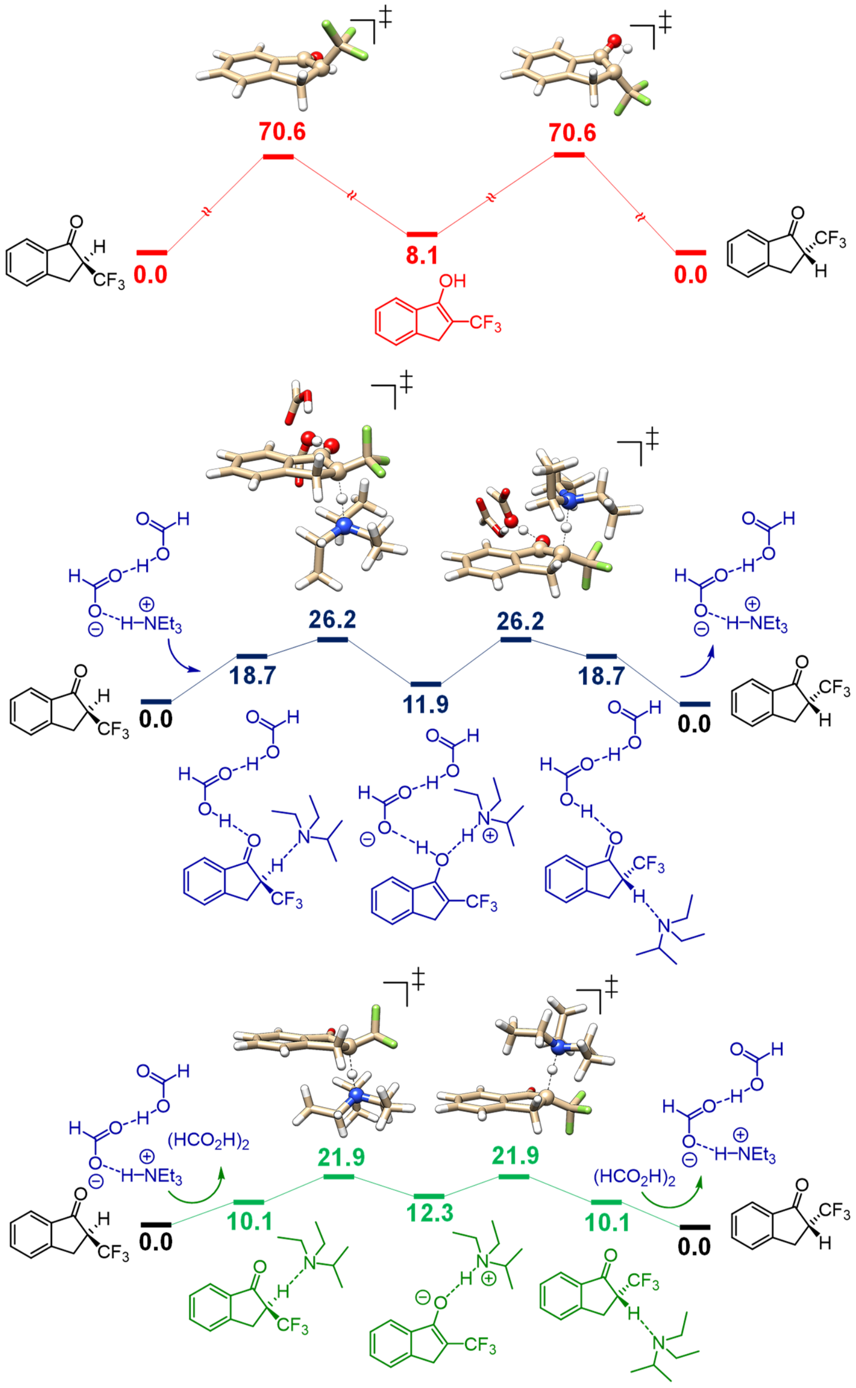

Section 2: Cartesian coordinates and energy data for the optimized structures

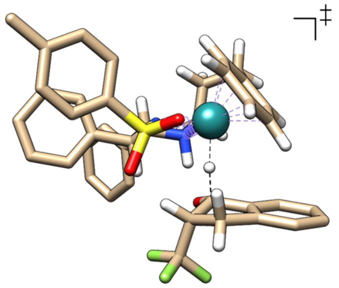

Thermal correction: 0.63541
E: -2675.696596
i295

44	-1.057418000	-0.966167000	1.219040000
8	1.040025000	-0.867022000	-2.381629000
6	7.072114000	-2.919917000	-0.519479000
1	7.232911000	-3.821171000	0.087812000
1	7.560305000	-3.060382000	-1.493235000
1	7.579509000	-2.083886000	-0.011583000
6	5.606109000	-2.622640000	-0.681169000
6	4.696208000	-2.932560000	0.341664000
1	5.056251000	-3.426714000	1.247119000
6	3.346690000	-2.620261000	0.219198000
1	2.648576000	-2.851098000	1.027190000
6	2.897983000	-1.982733000	-0.936513000
6	3.772839000	-1.678576000	-1.972797000
1	3.392217000	-1.180881000	-2.865619000
6	5.123013000	-2.000958000	-1.837781000
1	5.816162000	-1.757231000	-2.646271000
8	0.455670000	-2.868421000	-0.984925000
16	1.161150000	-1.587981000	-1.104545000
7	0.762239000	-0.649050000	0.159987000
6	1.075111000	0.777808000	-0.000222000
1	0.453258000	1.227724000	-0.798676000
6	0.684469000	1.465591000	1.315994000
1	1.321584000	1.049316000	2.111791000
7	-0.714550000	1.094785000	1.627401000
1	-1.330548000	1.550418000	0.897471000
6	-1.166449000	1.599548000	2.938898000
1	-0.850055000	2.649328000	3.036649000
1	-2.267943000	1.597361000	2.927267000
6	-0.658470000	0.798791000	4.135306000
1	0.392050000	0.506173000	3.974966000
1	-0.664803000	1.452415000	5.019286000
6	-1.509802000	-0.435217000	4.453816000
1	-2.525564000	-0.111452000	4.723142000
1	-1.088074000	-0.954896000	5.326817000
6	-1.595821000	-1.413506000	3.310980000
6	-0.460760000	-2.201462000	2.964098000
1	0.457012000	-2.123717000	3.548298000
6	-0.506171000	-3.016748000	1.805028000
1	0.392642000	-3.530530000	1.463885000
6	-1.673146000	-3.101924000	1.013147000
1	-1.665393000	-3.681828000	0.093369000
6	-2.795298000	-2.311851000	1.366496000
1	-3.669312000	-2.292363000	0.711918000
6	-2.778283000	-1.498016000	2.523789000
1	-3.641826000	-0.879229000	2.770549000
6	2.529390000	1.073983000	-0.324179000
6	2.867099000	1.734184000	-1.505900000
1	2.074106000	2.018170000	-2.201820000
6	4.201621000	2.019705000	-1.802617000
1	4.451648000	2.536955000	-2.730693000
6	5.210033000	1.645957000	-0.916179000
1	6.253947000	1.866474000	-1.147532000
6	4.879851000	0.985324000	0.270433000
1	5.665746000	0.685692000	0.966977000
6	3.547691000	0.702590000	0.562179000
1	3.294804000	0.168293000	1.481680000
6	0.909843000	2.964029000	1.247874000
6	1.914494000	3.556135000	2.018331000
1	2.513794000	2.934183000	2.688434000
6	2.164874000	4.925866000	1.929265000
1	2.954739000	5.374668000	2.534287000
6	1.406725000	5.717299000	1.067050000
1	1.600042000	6.789121000	0.994959000
6	0.399548000	5.133226000	0.297264000
1	-0.197962000	5.747876000	-0.378554000
6	0.150581000	3.763689000	0.382338000
1	-0.643077000	3.312339000	-0.220848000
1	-1.771311000	-0.226440000	-0.192558000
8	-2.325490000	1.849718000	-0.342177000
6	-2.486991000	0.699585000	-0.852342000
6	-3.818965000	-0.014246000	-0.807456000
6	-1.913280000	0.325852000	-2.254243000
6	-3.883449000	-0.988900000	-1.802995000
6	-4.866033000	0.206809000	0.077926000
6	-2.611396000	-0.994217000	-2.624361000
1	-0.821572000	0.216899000	-2.247962000
6	-5.015305000	-1.795358000	-1.906286000
6	-6.002242000	-0.599349000	-0.027817000
1	-4.788923000	0.996718000	0.829060000
6	-6.070505000	-1.595348000	-1.008757000
1	-5.087650000	-2.564187000	-2.678807000
1	-6.844860000	-0.449251000	0.649736000
1	-6.965414000	-2.216280000	-1.082321000
6	-2.204889000	1.432414000	-3.236490000
9	-1.496197000	2.534624000	-2.973949000
9	-1.887129000	1.067855000	-4.489615000
9	-3.496110000	1.788158000	-3.262296000
1	-2.797223000	-1.095922000	-3.702734000
1	-1.956530000	-1.831276000	-2.328558000

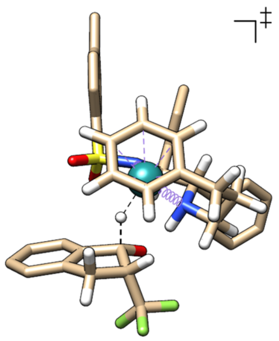

Thermal correction: 0.637742
E: -2675.686537
i330

44	-0.673159000	-0.357016000	1.502892000
8	1.238013000	-1.364554000	-2.207311000
6	7.176237000	-2.664165000	0.458325000
1	7.311570000	-3.205324000	1.403916000
1	7.676260000	-3.229435000	-0.341262000
1	7.692768000	-1.695308000	0.543822000
6	5.718694000	-2.458870000	0.146112000
6	4.725805000	-2.739846000	1.094421000
1	5.013236000	-3.144373000	2.067506000
6	3.379634000	-2.519181000	0.810343000
1	2.609026000	-2.755993000	1.546807000
6	3.018850000	-2.002553000	-0.431584000
6	3.983088000	-1.741169000	-1.401693000
1	3.672423000	-1.354580000	-2.372816000
6	5.325043000	-1.968363000	-1.106438000
1	6.085009000	-1.756950000	-1.862743000
8	0.549584000	-2.793934000	-0.238262000
16	1.293175000	-1.647428000	-0.766996000
7	0.902029000	-0.334781000	0.097788000
6	1.167330000	0.944299000	-0.563839000
1	0.693933000	0.961154000	-1.559802000
6	0.503965000	2.050104000	0.277421000
1	1.081436000	2.177594000	1.207484000
7	-0.850250000	1.574380000	0.639408000
1	-1.345126000	1.345401000	-0.279269000
6	-1.683660000	2.561411000	1.350452000
1	-1.623005000	3.523270000	0.819306000
1	-2.727560000	2.222814000	1.260924000
6	-1.336563000	2.782514000	2.820339000
1	-0.247380000	2.902254000	2.939142000
1	-1.780164000	3.740095000	3.128368000
6	-1.869244000	1.695882000	3.761657000
1	-2.965331000	1.651419000	3.681173000
1	-1.629182000	1.964867000	4.802062000
6	-1.304077000	0.329445000	3.485152000
6	0.117256000	0.136355000	3.507172000
1	0.777910000	0.965545000	3.764194000
6	0.661430000	-1.095446000	3.100475000
1	1.744448000	-1.193673000	3.018366000
6	-0.175185000	-2.178001000	2.704455000
1	0.255139000	-3.095362000	2.307451000
6	-1.567877000	-1.991812000	2.707539000
1	-2.214118000	-2.785641000	2.331116000
6	-2.140723000	-0.751799000	3.124296000
1	-3.221494000	-0.609381000	3.089224000
6	2.649198000	1.238373000	-0.728877000
6	3.173771000	1.592998000	-1.972400000
1	2.506604000	1.651986000	-2.835778000
6	4.538568000	1.854331000	-2.117857000
1	4.937308000	2.125988000	-3.097006000
6	5.389470000	1.763144000	-1.017443000
1	6.456685000	1.962534000	-1.131292000
6	4.870783000	1.412712000	0.232554000
1	5.533162000	1.334191000	1.097056000
6	3.510190000	1.151407000	0.372410000
1	3.105502000	0.850105000	1.342687000
6	0.501642000	3.360307000	-0.484412000
6	1.326797000	4.414347000	-0.086323000
1	1.957434000	4.298952000	0.798897000
6	1.359099000	5.604380000	-0.816051000
1	2.012064000	6.418999000	-0.497924000
6	0.558419000	5.749398000	-1.947672000
1	0.580479000	6.678981000	-2.519124000
6	-0.272894000	4.700516000	-2.348640000
1	-0.902546000	4.809355000	-3.233418000
6	-0.300317000	3.509957000	-1.624998000
1	-0.953071000	2.689781000	-1.940878000
1	-1.691014000	-0.791741000	0.097812000
8	-2.017641000	0.614588000	-1.490718000
6	-2.481020000	-0.413729000	-0.896625000
6	-2.576030000	-1.751904000	-1.596075000
6	-3.836909000	-0.318328000	-0.104194000
6	-3.633501000	-2.505127000	-1.086801000
6	-1.785044000	-2.215050000	-2.642009000
6	-4.342752000	-1.765483000	0.025732000
1	-3.703695000	0.174385000	0.867102000
6	-3.916222000	-3.761438000	-1.619321000
6	-2.069074000	-3.473610000	-3.174777000
1	-0.952667000	-1.612583000	-3.006696000
6	-3.124548000	-4.239573000	-2.667109000
1	-4.747867000	-4.357241000	-1.236523000
1	-1.464452000	-3.864914000	-3.995064000
1	-3.336697000	-5.219394000	-3.099476000
6	-4.807509000	0.555150000	-0.862840000
9	-4.438117000	1.839136000	-0.857640000
9	-4.962555000	0.184666000	-2.136294000
9	-6.031878000	0.517488000	-0.303944000
1	-4.053284000	-2.174604000	1.006885000
1	-5.437411000	-1.842765000	-0.041922000

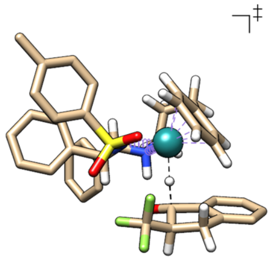

Thermal correction: 0.636765
E: -2675.684025
i543

44	1.127481000	-0.697942000	-1.005635000
8	-1.663852000	-1.514264000	2.205692000

6	-7.099565000	-2.251061000	-1.522462000
1	-7.097577000	-2.664849000	-2.539778000
1	-7.751818000	-2.871539000	-0.892550000
1	-7.549501000	-1.246428000	-1.572086000
6	-5.706496000	-2.175202000	-0.957972000
6	-4.589070000	-2.113221000	-1.803501000
1	-4.733466000	-2.155055000	-2.885639000
6	-3.303770000	-1.990802000	-1.283912000
1	-2.442788000	-1.911820000	-1.951751000
6	-3.125353000	-1.926527000	0.096327000
6	-4.214668000	-2.003655000	0.957680000
1	-4.048667000	-1.946238000	2.034230000
6	-5.497747000	-2.126840000	0.425445000
1	-6.356439000	-2.175458000	1.099524000
8	-0.798640000	-3.034484000	0.401891000
16	-1.469143000	-1.784633000	0.778745000
7	-0.756996000	-0.557789000	-0.003142000
6	-1.077616000	0.798053000	0.465797000
1	-0.517092000	1.021964000	1.391652000
6	-0.577722000	1.754644000	-0.626297000
1	-1.153016000	1.545780000	-1.540435000
7	0.836532000	1.422013000	-0.916317000
1	1.380288000	1.678653000	-0.052634000
6	1.396812000	2.212147000	-2.029996000
1	1.115180000	3.267014000	-1.889919000
1	2.493102000	2.159525000	-1.947454000
6	0.970090000	1.748428000	-3.420853000
1	-0.095212000	1.466657000	-3.419283000
1	1.062691000	2.597202000	-4.113353000
6	1.822144000	0.597414000	-3.964533000
1	2.866657000	0.931340000	-4.047269000
1	1.480554000	0.332263000	-4.976366000
6	1.768793000	-0.638968000	-3.106407000
6	0.563872000	-1.402406000	-3.038277000
1	-0.303709000	-1.106543000	-3.630194000
6	0.478588000	-2.486708000	-2.136772000
1	-0.470801000	-2.999627000	-1.987446000
6	1.575347000	-2.858222000	-1.322642000
1	1.456681000	-3.644229000	-0.580494000
6	2.768169000	-2.101619000	-1.409109000
1	3.597510000	-2.318178000	-0.734299000
6	2.888539000	-1.024765000	-2.322609000
1	3.804563000	-0.433956000	-2.351126000
6	-2.551654000	1.063859000	0.744263000
6	-2.987359000	1.242476000	2.058711000
1	-2.261059000	1.164023000	2.870172000
6	-4.333124000	1.487227000	2.337229000
1	-4.656780000	1.620648000	3.371165000
6	-5.260790000	1.558359000	1.298885000
1	-6.313881000	1.748745000	1.515018000
6	-4.835365000	1.384181000	-0.019972000
1	-5.554892000	1.437655000	-0.839847000
6	-3.491424000	1.138227000	-0.291717000
1	-3.176876000	0.987507000	-1.326956000
6	-0.776958000	3.209153000	-0.245404000
6	-1.632404000	4.022490000	-0.994671000
1	-2.143266000	3.604161000	-1.865769000
6	-1.842272000	5.354922000	-0.638446000
1	-2.513998000	5.976938000	-1.232874000
6	-1.194782000	5.887723000	0.475520000
1	-1.357621000	6.929485000	0.757696000
6	-0.336718000	5.083007000	1.227080000
1	0.173109000	5.495566000	2.099696000
6	-0.127551000	3.750637000	0.872400000
1	0.554896000	3.127587000	1.456591000
1	1.973873000	-0.319251000	0.507943000
8	2.217181000	1.664934000	1.375117000
6	2.755604000	0.525806000	1.314888000
6	4.109364000	0.333437000	0.661668000
6	2.847869000	-0.424033000	2.546890000
6	4.736723000	-0.813876000	1.146111000
6	4.739210000	1.184452000	-0.239592000
6	3.859639000	-1.512210000	2.159240000
1	3.258477000	0.210775000	3.348841000
6	6.004554000	-1.162607000	0.682947000
6	6.011469000	0.839501000	-0.701288000
1	4.246024000	2.107330000	-0.553727000
6	6.632038000	-0.331645000	-0.250218000
1	6.508635000	-2.058068000	1.053225000
1	6.533682000	1.490134000	-1.405224000
1	7.628819000	-0.587735000	-0.614146000
6	1.544971000	-0.966368000	3.065588000
9	1.049827000	-1.926269000	2.284517000
9	1.709729000	-1.517160000	4.282401000
9	0.622563000	-0.012830000	3.193216000
1	4.422111000	-1.900966000	3.018391000
1	3.337074000	-2.362629000	1.687807000

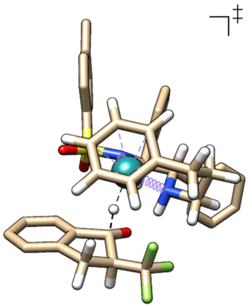

Thermal correction: 0.639093
E: -2675.680885
i319

44	-0.999349000	-0.448338000	1.239653000
8	1.536711000	-1.406501000	-2.166242000
6	7.226276000	-2.369300000	1.053611000
1	7.328301000	-3.113601000	1.854790000
1	7.876432000	-2.662177000	0.217729000
1	7.602345000	-1.408612000	1.440765000
6	5.795478000	-2.233557000	0.608079000
6	4.737280000	-2.565069000	1.465696000
1	4.955804000	-2.967814000	2.457513000
6	3.412513000	-2.404516000	1.066020000
1	2.596183000	-2.699361000	1.726756000
6	3.133465000	-1.891988000	-0.198674000
6	4.167086000	-1.588465000	-1.080882000
1	3.925258000	-1.216539000	-2.076647000
6	5.488182000	-1.758070000	-0.672891000
1	6.298805000	-1.514067000	-1.363882000
8	0.739997000	-2.882170000	-0.270112000

16	1.425745000	-1.667659000	-0.722567000
7	0.798134000	-0.391493000	0.051863000
6	1.096252000	0.899206000	-0.573862000
1	0.691955000	0.923897000	-1.599512000
6	0.388803000	1.996024000	0.238194000
1	0.895179000	2.085033000	1.211810000
7	-1.004993000	1.579088000	0.514720000
1	-1.453201000	1.474812000	-0.416217000
6	-1.752358000	2.588222000	1.297984000
1	-1.556540000	3.580952000	0.866072000
1	-2.820374000	2.389985000	1.164644000
6	-1.441352000	2.617401000	2.790569000
1	-0.353678000	2.590079000	2.964826000
1	-1.784667000	3.587575000	3.178012000
6	-2.152954000	1.516758000	3.585786000
1	-3.237655000	1.598732000	3.422960000
1	-1.969493000	1.665116000	4.661446000
6	-1.704716000	0.127652000	3.220491000
6	-0.319663000	-0.219555000	3.357513000
1	0.392033000	0.504217000	3.757050000
6	0.131223000	-1.458923000	2.879222000
1	1.200953000	-1.666054000	2.882567000
6	-0.764164000	-2.403630000	2.302287000
1	-0.382052000	-3.315854000	1.850198000
6	-2.129690000	-2.079359000	2.222998000
1	-2.815084000	-2.771634000	1.731896000
6	-2.613820000	-0.830479000	2.710091000
1	-3.667498000	-0.577437000	2.609941000
6	2.577098000	1.245489000	-0.622798000
6	3.177337000	1.634372000	-1.820521000
1	2.580302000	1.645124000	-2.735572000
6	4.525633000	1.997767000	-1.853241000
1	4.984019000	2.298385000	-2.797358000
6	5.283959000	1.977424000	-0.683440000
1	6.337875000	2.261298000	-0.707982000
6	4.689942000	1.588895000	0.520804000
1	5.280864000	1.564462000	1.438845000
6	3.346683000	1.222998000	0.547253000
1	2.885197000	0.894182000	1.483193000
6	0.478540000	3.323251000	-0.489695000
6	1.284431000	4.350341000	0.006353000
1	1.829597000	4.200741000	0.941912000
6	1.408022000	5.553801000	-0.689269000
1	2.046258000	6.346213000	-0.294361000
6	0.718338000	5.739982000	-1.886608000
1	0.812720000	6.680386000	-2.432587000
6	-0.093114000	4.718648000	-2.385596000
1	-0.633427000	4.859763000	-3.323464000
6	-0.211647000	3.513458000	-1.694934000
1	-0.842544000	2.711061000	-2.091649000
1	-1.987440000	-0.613083000	-0.186940000
8	-1.747229000	0.535751000	-1.986754000
6	-2.403128000	-0.407893000	-1.485116000
6	-2.303115000	-1.860960000	-1.918784000
6	-3.953858000	-0.287640000	-1.267555000
6	-3.436135000	-2.572786000	-1.509582000
6	-1.303730000	-2.459613000	-2.673962000
6	-4.414638000	-1.666970000	-0.796463000
1	-4.318844000	-0.115298000	-2.294165000
6	-3.562556000	-3.924188000	-1.817046000
6	-1.436249000	-3.814286000	-2.989606000
1	-0.432495000	-1.884089000	-2.984238000
6	-2.548121000	-4.541418000	-2.557157000
1	-4.445826000	-4.488324000	-1.508812000
1	-0.661531000	-4.310566000	-3.577214000
1	-2.635214000	-5.599302000	-2.812867000
6	-4.491333000	0.867240000	-0.479620000
9	-3.940259000	2.031982000	-0.839741000
9	-5.814548000	0.994354000	-0.654801000
9	-4.308043000	0.748042000	0.846809000
1	-4.304845000	-1.745579000	0.299233000
1	-5.461623000	-1.883980000	-1.047847000

NEt_3
Thermal correction: 0.172415
E: -292.4069757

7	0.178209000	0.002018000	0.381386000
6	-0.230519000	1.121485000	-0.454749000
1	0.633181000	1.794982000	-0.555401000
1	-0.480816000	0.790468000	-1.487513000
6	-1.393926000	1.915666000	0.119689000
1	-1.188094000	2.189879000	1.165402000
1	-2.338927000	1.354033000	0.090834000
1	-1.540961000	2.839558000	-0.457313000
6	-0.897636000	-0.925737000	0.709189000
1	-0.475939000	-1.688574000	1.382537000
1	-1.656242000	-0.388548000	1.299054000
6	-1.575497000	-1.616254000	-0.475256000
1	-0.853021000	-2.185599000	-1.079696000
1	-2.076334000	-0.893222000	-1.137024000
1	-2.339506000	-2.321681000	-0.116738000
6	1.366277000	-0.664439000	-0.132779000
1	1.484923000	-1.604642000	0.428209000
1	1.250770000	-0.950439000	-1.201317000
6	2.636660000	0.160719000	0.029848000
1	2.744325000	0.485046000	1.075422000
1	2.639983000	1.055110000	-0.609127000
1	3.517043000	-0.439133000	-0.242693000

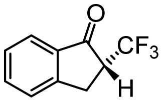

Thermal correction: 0.115095
E: -760.150365

8	-0.544397000	2.187701000	-0.233603000
6	-0.146255000	1.055735000	-0.318140000
6	1.235899000	0.563252000	-0.125937000
6	-1.010252000	-0.176057000	-0.661262000
6	1.256554000	-0.830879000	-0.184640000
6	2.389968000	1.317887000	0.096257000
6	-0.125243000	-1.406155000	-0.393496000
1	-1.279121000	-0.097517000	-1.726367000
6	2.465963000	-1.508657000	-0.027852000
6	3.593309000	0.638716000	0.250559000
1	2.334437000	2.407199000	0.140669000
6	3.625252000	-0.763305000	0.186647000
1	2.506833000	-2.598609000	-0.069491000
1	4.517987000	1.191983000	0.420671000

1	4.578723000	-1.280404000	0.309667000
6	-2.303647000	-0.184291000	0.110450000
9	-3.084775000	0.846992000	-0.202396000
9	-3.004913000	-1.299416000	-0.127442000
9	-2.087235000	-0.135904000	1.430906000
1	-0.449281000	-1.942727000	0.512084000
1	-0.151365000	-2.124055000	-1.223534000

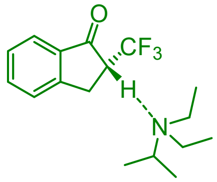

Thermal correction: 0.307017
E: -1052.564408

8	0.789580000	0.402994000	-2.244396000
6	1.125779000	0.339737000	-1.089336000
6	2.332229000	-0.317813000	-0.534218000
6	0.356392000	0.914473000	0.108851000
6	2.440432000	-0.033534000	0.828446000
6	3.266211000	-1.107548000	-1.207741000
6	1.327653000	0.873991000	1.300352000
1	-0.499407000	0.227719000	0.285109000
6	3.505841000	-0.560936000	1.558873000
6	4.326248000	-1.631666000	-0.475732000
1	3.151792000	-1.304491000	-2.275379000
6	4.439041000	-1.358832000	0.896484000
1	3.610602000	-0.354148000	2.625537000
1	5.073162000	-2.259276000	-0.963957000
1	5.276340000	-1.779964000	1.456163000
6	-0.223661000	2.271532000	-0.168149000
9	-1.123552000	2.249290000	-1.154820000
9	-0.843065000	2.767027000	0.912764000
9	0.715576000	3.161182000	-0.515442000
1	1.729819000	1.874674000	1.526111000
1	0.849278000	0.498024000	2.214924000
7	-2.288315000	-0.928389000	0.251128000
6	-2.516797000	-2.170726000	0.977407000
1	-2.454470000	-1.944702000	2.051576000
1	-3.544437000	-2.558093000	0.803666000
6	-1.500985000	-3.255825000	0.654080000
1	-0.475301000	-2.867129000	0.753209000
1	-1.620536000	-3.651659000	-0.364538000
1	-1.617268000	-4.097233000	1.351542000
6	-2.202493000	-1.081570000	-1.200109000
1	-1.944964000	-0.094416000	-1.616102000
1	-1.344087000	-1.732100000	-1.429091000
6	-3.451030000	-1.626304000	-1.891433000
1	-4.326820000	-0.984380000	-1.713910000
1	-3.696991000	-2.642526000	-1.547399000
1	-3.287644000	-1.673483000	-2.977819000
6	-3.198822000	0.138140000	0.651566000
1	-3.017416000	0.987642000	-0.025756000
1	-4.261238000	-0.154664000	0.511929000
6	-2.994064000	0.603662000	2.087609000
1	-1.932380000	0.821675000	2.277646000
1	-3.332032000	-0.139751000	2.823006000
1	-3.566918000	1.524877000	2.266595000

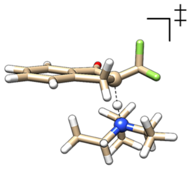

Thermal correction: 0.305802
E: -1052.544408
i914

8	0.555640000	0.603595000	-2.323808000
6	0.842159000	0.601389000	-1.135718000
6	2.133309000	0.098189000	-0.566160000
6	0.032728000	1.018431000	0.011510000
6	2.179312000	0.368007000	0.803170000
6	3.170458000	-0.559547000	-1.224392000
6	0.932869000	1.099603000	1.243230000
1	-0.891864000	-0.183268000	0.168646000
6	3.280144000	-0.045425000	1.553487000
6	4.272717000	-0.966570000	-0.474276000
1	3.102997000	-0.750044000	-2.297626000
6	4.321772000	-0.711994000	0.903601000
1	3.332664000	0.148752000	2.626993000
1	5.101765000	-1.488351000	-0.955533000
1	5.190954000	-1.039388000	1.477438000
6	-0.899278000	2.139991000	-0.211092000
9	-1.820346000	1.883052000	-1.160223000
9	-1.591758000	2.453455000	0.907721000
9	-0.311510000	3.298256000	-0.590186000
1	1.186791000	2.141723000	1.510119000
1	0.500249000	0.642100000	2.149014000
7	-1.709729000	-1.101458000	0.209780000
6	-1.118716000	-2.228369000	0.969749000
1	-0.806188000	-1.822672000	1.940808000
1	-1.908893000	-2.973093000	1.164399000
6	0.073894000	-2.869358000	0.282914000
1	0.832139000	-2.119948000	0.012195000
1	-0.210387000	-3.421943000	-0.622887000
1	0.540075000	-3.581724000	0.977019000
6	-1.949604000	-1.392902000	-1.227402000
1	-2.318537000	-0.456671000	-1.667766000
1	-0.969411000	-1.579863000	-1.684123000
6	-2.901818000	-2.544328000	-1.499166000
1	-3.911887000	-2.351727000	-1.112195000
1	-2.537422000	-3.489751000	-1.071513000
1	-2.986188000	-2.682847000	-2.585818000
6	-2.916146000	-0.543170000	0.865933000
1	-3.219652000	0.320859000	0.257562000
1	-3.724882000	-1.288821000	0.817188000
6	-2.680291000	-0.112240000	2.303231000
1	-1.791594000	0.529068000	2.384931000
1	-2.570401000	-0.967260000	2.983475000
1	-3.545877000	0.473189000	2.642152000

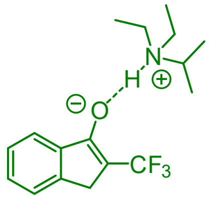

Thermal correction: 0.309692
E: -1052.563548

8	-0.109665000	-0.897299000	-0.427745000
6	0.930151000	-0.169085000	-0.284493000
6	2.289750000	-0.770628000	-0.163225000
6	1.104969000	1.201710000	-0.196357000
6	3.239313000	0.244498000	0.007289000
6	2.654762000	-2.113915000	-0.193332000
6	2.549941000	1.586476000	0.001424000
1	-1.444328000	-0.557202000	-0.146691000
6	4.585995000	-0.077627000	0.149351000
6	4.006503000	-2.434560000	-0.052110000
1	1.893841000	-2.885807000	-0.324436000
6	4.961621000	-1.425035000	0.117317000
1	5.338473000	0.702751000	0.283238000
1	4.324924000	-3.478561000	-0.072917000
1	6.014193000	-1.693489000	0.226191000
6	0.045866000	2.208111000	-0.283891000
9	-1.073526000	1.739850000	-0.874615000
9	-0.374032000	2.702143000	0.912570000
9	0.419708000	3.298416000	-0.981525000
1	2.936726000	2.229288000	-0.808175000
1	2.730768000	2.126640000	0.947739000
7	-2.535776000	-0.598759000	0.162145000
6	-2.698470000	-2.009852000	0.610671000
1	-1.939102000	-2.169482000	1.385634000
1	-3.689158000	-2.113692000	1.079432000
6	-2.493497000	-3.015351000	-0.506922000
1	-1.532945000	-2.829881000	-1.007287000
1	-3.301929000	-2.990597000	-1.250360000
1	-2.468206000	-4.024628000	-0.074180000
6	-3.353335000	-0.249916000	-1.031033000
1	-3.137663000	0.802357000	-1.251712000
1	-2.963261000	-0.841111000	-1.869770000
6	-4.845960000	-0.467516000	-0.859141000
1	-5.256571000	0.127065000	-0.031023000
1	-5.095946000	-1.524558000	-0.690587000
1	-5.355645000	-0.148776000	-1.778625000
6	-2.734739000	0.361756000	1.281470000
1	-2.709756000	1.364859000	0.836969000
1	-3.737575000	0.199357000	1.703955000
6	-1.666353000	0.247597000	2.355142000
1	-0.658289000	0.302969000	1.919413000
1	-1.752235000	-0.680096000	2.936477000
1	-1.777000000	1.088640000	3.052838000

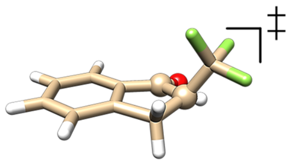

Thermal correction: 0.109514

E: -760.032224
i2146

8	-0.641305000	2.184379000	-0.718206000
6	-0.160170000	1.060242000	-0.397174000
6	1.178288000	0.609010000	-0.062962000
6	-1.051358000	-0.033387000	-0.708393000
6	1.194476000	-0.769160000	-0.353559000
6	2.316743000	1.303527000	0.359204000
6	-0.149145000	-1.260566000	-0.865630000
1	-1.419861000	1.322466000	-1.270294000
6	2.386772000	-1.477279000	-0.208940000
6	3.487916000	0.576515000	0.530253000
1	2.276260000	2.375726000	0.556362000
6	3.516953000	-0.798938000	0.247307000
1	2.434414000	-2.543124000	-0.438557000
1	4.393800000	1.074317000	0.878242000
1	4.450215000	-1.348368000	0.383976000
6	-2.188549000	-0.233181000	0.235770000
9	-2.806776000	0.908351000	0.565538000
9	-3.113704000	-1.052602000	-0.281407000
9	-1.817695000	-0.800866000	1.403956000
1	-0.501122000	-2.133518000	-0.296295000
1	-0.051242000	-1.577172000	-1.915822000

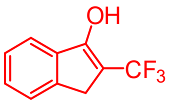

Thermal correction: 0.114562
E: -760.136975

8	0.426873000	2.228246000	-0.000001000
6	0.124159000	0.931601000	-0.000004000
6	-1.287312000	0.525044000	0.000000000
6	0.908246000	-0.176865000	-0.000009000
6	-1.333865000	-0.877249000	-0.000001000
6	-2.444257000	1.301508000	0.000001000
6	0.068580000	-1.428330000	-0.000003000
1	1.387445000	2.352695000	-0.000012000
6	-2.561588000	-1.530634000	-0.000001000
6	-3.672025000	0.638590000	0.000002000
1	-2.386685000	2.391099000	0.000002000
6	-3.728522000	-0.760335000	0.000001000
1	-2.616338000	-2.620890000	-0.000003000
1	-4.598636000	1.214793000	0.000004000
1	-4.700133000	-1.257370000	0.000001000
6	2.385439000	-0.220548000	0.000000000
9	2.921177000	1.018171000	-0.000021000
9	2.885106000	-0.854353000	1.069037000
9	2.885118000	-0.854396000	-1.069005000
1	0.261801000	-2.053898000	-0.887308000
1	0.261805000	-2.053885000	0.887311000

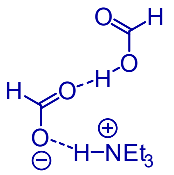

Thermal correction: 0.231398

E: -672.0315644

7	1.514069000	-0.071913000	-0.117248000
6	1.729806000	-1.362956000	-0.824950000
1	1.299513000	-1.240722000	-1.826397000
1	2.813278000	-1.513523000	-0.941796000
6	1.067428000	-2.533151000	-0.121983000
1	0.008413000	-2.318474000	0.084421000
1	1.566360000	-2.792499000	0.821512000
1	1.112169000	-3.412847000	-0.777622000
6	1.899417000	-0.088598000	1.324034000
1	1.632824000	0.906355000	1.702653000
1	1.236315000	-0.809229000	1.820200000
6	3.355478000	-0.425013000	1.578518000
1	4.036732000	0.289966000	1.096451000
1	3.611129000	-1.439723000	1.241051000
1	3.540641000	-0.379300000	2.660264000
6	2.106196000	1.090134000	-0.839591000
1	1.929374000	1.955038000	-0.188870000
1	3.188075000	0.920908000	-0.935768000
6	1.454768000	1.326353000	-2.190262000
1	0.359672000	1.356314000	-2.091006000
1	1.724012000	0.563381000	-2.932737000
1	1.788437000	2.298236000	-2.577849000
1	0.437973000	0.098296000	-0.110568000
8	-1.062489000	0.351023000	-0.032148000
8	-0.294224000	2.229880000	0.881801000
6	-1.209389000	1.493451000	0.546201000
1	-2.271043000	1.777468000	0.733581000
1	-2.237502000	-0.485975000	-0.364926000
8	-3.009468000	-1.140143000	-0.589541000
8	-4.272608000	0.261801000	0.619206000
6	-4.121623000	-0.740917000	-0.036421000
1	-4.957031000	-1.446584000	-0.239677000

$(HCO_2H)_2$
Thermal correction: 0.040806
E: -379.6027964

1	-4.784581000	-1.830303000	-1.227116000
8	-3.878819000	-1.853881000	-1.673999000
8	-3.267555000	-0.530495000	0.022055000
6	-3.026301000	-1.144258000	-1.001849000
1	-2.017005000	-1.140232000	-1.452172000
1	-4.666167000	-0.454395000	0.767541000
8	-5.572189000	-0.430108000	1.214205000
8	-6.183532000	-1.754957000	-0.480710000
6	-6.424763000	-1.140089000	0.542532000
1	-7.434149000	-1.143401000	0.992694000

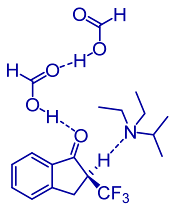

Thermal correction: 0.363268
E: -1432.165972

8	-0.792865000	0.479595000	1.501845000
6	0.047329000	1.023814000	0.807190000
6	-0.158127000	2.050783000	-0.223774000
6	1.531863000	0.710679000	0.844395000
6	1.084910000	2.506308000	-0.674071000
6	-1.363758000	2.553508000	-0.723410000
6	2.222536000	1.834492000	0.056148000
1	1.625951000	-0.267297000	0.306356000
6	1.140182000	3.482549000	-1.669190000
6	-1.301935000	3.527033000	-1.711800000
1	-2.320869000	2.185167000	-0.348685000
6	-0.057599000	3.981738000	-2.179014000
1	2.098161000	3.850640000	-2.040127000
1	-2.219658000	3.939749000	-2.132398000
1	-0.029835000	4.745127000	-2.958843000
6	2.067288000	0.498364000	2.232199000
9	1.537249000	-0.571437000	2.822657000
9	3.395859000	0.320542000	2.211568000
9	1.831841000	1.548865000	3.029462000
1	2.713353000	2.553964000	0.731117000
1	2.989773000	1.452255000	-0.631163000
7	1.745059000	-1.989405000	-0.847042000
6	3.041805000	-2.565936000	-0.514950000
1	2.983402000	-2.928712000	0.523241000
1	3.254353000	-3.460367000	-1.139393000
6	4.196475000	-1.582532000	-0.626022000
1	4.005132000	-0.682141000	-0.023105000
1	4.381130000	-1.273753000	-1.665317000
1	5.119580000	-2.047303000	-0.251581000
6	1.664133000	-1.378085000	-2.171327000
1	0.654118000	-0.948970000	-2.258658000
1	2.366903000	-0.529573000	-2.208592000
6	1.934176000	-2.312264000	-3.349367000
1	1.246222000	-3.171561000	-3.344038000
1	2.964172000	-2.699849000	-3.336597000
1	1.791646000	-1.776363000	-4.299107000
6	0.650805000	-2.919653000	-0.575076000
1	-0.206320000	-2.619209000	-1.193754000
1	0.924136000	-3.950799000	-0.881436000
6	0.219918000	-2.912573000	0.885193000
1	-0.147576000	-1.917018000	1.175464000
1	1.046268000	-3.180606000	1.560505000
1	-0.589550000	-3.640154000	1.047889000
1	-2.249811000	0.099410000	0.936096000
8	-3.073745000	-0.258545000	0.486932000
8	-1.660630000	-0.689862000	-1.192742000
6	-2.761726000	-0.737608000	-0.716413000
1	-3.650159000	-1.174371000	-1.209176000
1	-4.712656000	-0.402203000	1.180149000
8	-5.644000000	-0.505661000	1.482673000
8	-5.906605000	-1.388153000	-0.557288000
6	-6.343684000	-1.062800000	0.514912000
1	-7.402617000	-1.197843000	0.812169000

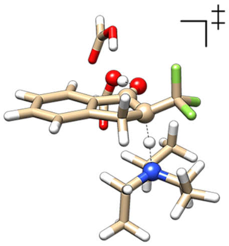

Thermal correction: 0.36297
E: -1432.153692
i1172

8	0.949176000	-0.508196000	-1.063483000
6	0.064112000	0.340920000	-0.903423000
6	0.250273000	1.728257000	-0.415498000
6	-1.363785000	0.154829000	-1.137437000
6	-0.958217000	2.421090000	-0.554987000
6	1.399982000	2.343381000	0.085026000
6	-2.009289000	1.541510000	-1.183125000
1	-1.783016000	-0.408053000	0.043970000
6	-1.045761000	3.751236000	-0.146101000
6	1.308892000	3.673163000	0.485176000
1	2.341738000	1.797652000	0.153090000
6	0.093631000	4.365019000	0.376300000
1	-1.981528000	4.305568000	-0.239537000
1	2.185799000	4.182419000	0.887276000
1	0.041900000	5.406452000	0.699243000
6	-1.746704000	-0.789228000	-2.220577000
9	-1.337959000	-2.045633000	-1.996295000
9	-3.083746000	-0.842419000	-2.371827000
9	-1.257441000	-0.448745000	-3.428010000
1	-2.190367000	1.867995000	-2.222233000
1	-2.973380000	1.602784000	-0.656580000
7	-2.188092000	-0.887175000	1.254670000
6	-3.214760000	-1.917797000	1.010666000
1	-2.800308000	-2.598087000	0.253299000
1	-3.369537000	-2.509602000	1.930764000
6	-4.532573000	-1.353406000	0.507857000
1	-4.374549000	-0.687956000	-0.352780000
1	-5.076519000	-0.801103000	1.286457000
1	-5.173654000	-2.181597000	0.176290000
6	-2.664270000	0.297634000	1.998191000
1	-1.822859000	1.007540000	2.020763000
1	-3.460429000	0.765252000	1.404166000
6	-3.167727000	0.025291000	3.408835000
1	-2.374218000	-0.346374000	4.070985000
1	-3.993537000	-0.701528000	3.411515000
1	-3.546804000	0.961867000	3.841120000
6	-0.952232000	-1.431543000	1.854540000
1	-0.224319000	-0.607009000	1.886495000
1	-1.157966000	-1.733640000	2.895274000
6	-0.342233000	-2.595154000	1.093224000
1	-0.176480000	-2.343077000	0.036963000
1	-0.952998000	-3.506746000	1.155734000
1	0.636882000	-2.817370000	1.540117000
1	2.284091000	-0.496871000	-0.433115000
8	3.214702000	-0.574541000	0.017247000
8	2.085717000	-0.379823000	1.937852000
6	3.114756000	-0.543168000	1.337585000
1	4.103239000	-0.678571000	1.817686000
1	4.734648000	-0.819887000	-0.784558000
8	5.623992000	-0.958689000	-1.194182000
8	6.304432000	-0.958619000	0.938678000
6	6.527267000	-1.041822000	-0.240872000
1	7.540799000	-1.200976000	-0.661075000

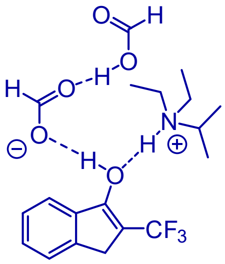

Thermal correction: 0.369318
E: -1432.182761

8	0.204837000	-0.236930000	0.583141000
6	-0.232598000	0.966923000	0.254287000
6	-1.540541000	1.231653000	-0.382627000
6	0.424595000	2.151178000	0.397801000
6	-1.654607000	2.616682000	-0.584612000
6	-2.543721000	0.351582000	-0.788076000
6	-0.406194000	3.305306000	-0.097808000
1	1.819945000	-0.703543000	-0.025890000
6	-2.795730000	3.144329000	-1.179439000
6	-3.687753000	0.888917000	-1.380291000
1	-2.452829000	-0.725269000	-0.643683000
6	-3.814347000	2.269366000	-1.570389000
1	-2.895393000	4.219450000	-1.341703000
1	-4.487788000	0.217595000	-1.698023000
1	-4.717613000	2.668427000	-2.035374000
6	1.741688000	2.359676000	1.032244000
9	2.426459000	1.214009000	1.205059000
9	2.532024000	3.168296000	0.301857000
9	1.660451000	2.935562000	2.242532000
1	-0.625053000	4.028127000	0.706506000
1	0.088544000	3.865680000	-0.908468000
7	2.514592000	-1.318550000	-0.501109000
6	3.723036000	-0.508690000	-0.838102000
1	4.103446000	-0.111542000	0.109122000
1	4.472888000	-1.203730000	-1.240026000
6	3.424521000	0.618856000	-1.805643000
1	2.558568000	1.211861000	-1.475488000
1	3.229774000	0.253557000	-2.822917000
1	4.292915000	1.289637000	-1.847241000
6	1.774830000	-1.841011000	-1.697685000
1	0.909643000	-2.377147000	-1.286514000
1	1.405219000	-0.964751000	-2.245606000
6	2.613500000	-2.735452000	-2.586398000
1	2.988835000	-3.615276000	-2.045155000
1	3.463135000	-2.203134000	-3.037004000
1	1.977693000	-3.099477000	-3.404581000
6	2.815904000	-2.404242000	0.489229000
1	1.941257000	-3.067461000	0.478182000
1	3.692184000	-2.951930000	0.117290000
6	3.038743000	-1.854638000	1.885213000
1	2.166277000	-1.273413000	2.216408000
1	3.935848000	-1.225374000	1.960718000

1	3.165466000	-2.699085000	2.575808000
1	-0.555940000	-0.962570000	0.811541000
8	-1.505887000	-1.890082000	1.176585000
8	-0.259713000	-3.501927000	0.262820000
6	-1.279843000	-3.099660000	0.795372000
1	-2.120938000	-3.803283000	0.998344000
1	-2.966230000	-1.573353000	1.531205000
8	-3.949433000	-1.365257000	1.712726000
8	-4.280946000	-2.322848000	-0.286984000
6	-4.681414000	-1.757263000	0.702429000
1	-5.753685000	-1.509656000	0.855946000

Design and molecular modeling of compound 10

Molecular docking

For the docking with the FRED software (OEDOCKING 3.3.0.2: OpenEye Scientific Software, Santa Fe, NM, USA. http://www.eyesopen.com),[23] the predicted Hsp90β CTD binding site[24] (PDB entry: 5FWK) was prepared using MAKE RECEPTOR (Release 3.2.0.2, OpenEye Scientific Software, Inc., Santa Fe, NM, USA; www.eyesopen.com). The grid box around the coumarin-based Hsp90 CTD inhibitor was generated automatically and was not adjusted. This resulted in a box with the following dimensions: 21.7 Å × 24.7 Å × 16.0 Å and the volume of 8551 Å^3. For "Cavity detection", the slow and effective "Molecular" method was used for detection of binding sites. Inner and outer contours of the grid box were also calculated automatically using "Balanced" settings for "Site Shape Potential" calculation. The inner contours were disabled. The library of ligand conformers was prepared by OMEGA (Release 3.3.1.2, OpenEye Scientific Software, Inc., Santa Fe, NM, USA; www.eyesopen.com). The ligand was then docked to the prepared Hsp90 CTD binding site using FRED (default settings). The results were visualized and analyzed with VIDA (version 4.3.0.4, OpenEye Scientific Software, Inc., Santa Fe, NM, USA, www.eyesopen.com).

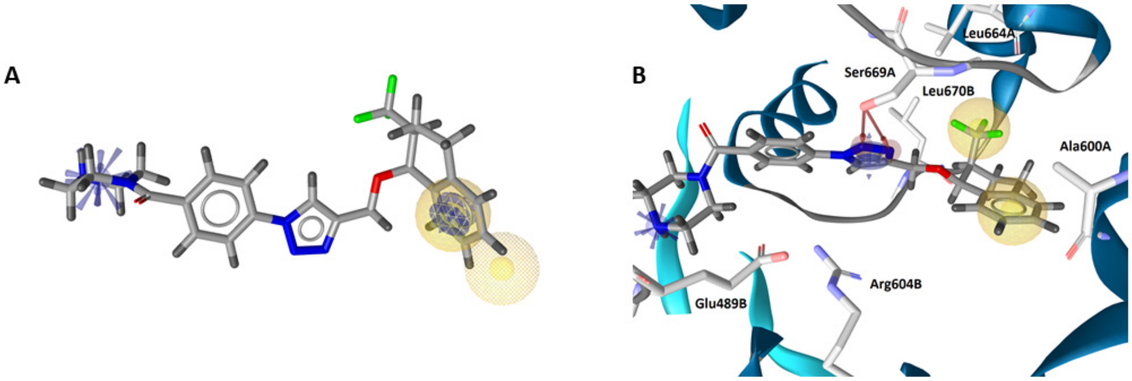

Figure S2. A) Alignment of compound **10** (grey sticks) with the three-dimensional structure-based pharmacophore model derived from molecular dynamics simulation of a coumarin-based Hsp90 CTD inhibitor in complex with Hsp90β.[25] The pharmacophore model included two with hydrophobic features (yellow spheres), one with an aromatic (blue disc), and a positive ionizable (blue star). **B)** Interactions of compound **10** with the Hsp90 C-terminal-domain binding site. The pharmacophore features are: hydrophobics (yellow spheres), hydrogen bond acceptors (red arrows), and positive ionizable (blue star). For clarity, only the amino acids that interact with **10** are shown. Compound **10** formed hydrogen bonds with Ser669A side chain, cation-π interaction with Arg604B and an ionic interaction with the Glu489A. Moreover, (1*S*,2*S*)-2-(trifluoromethyl)-1,2,3,4-tetrahydronaphthalene moiety of **10** formed a network of hydrophobic contacts with Ala600A, Leu664A and Leu670B.

[23] a) McGann, M. *J. Comput. Aided Mol. Des.* **2012**, *26*, 897–906. b) McGann, M. FRED *J. Chem. Inf. Model.* **2011**, *51*, 578–596.

[24] Tomašič, T.; Durcik, M.; Keegan, B. M.; Skledar, D. G.; Zajec, Ž.; Blagg, B. S. J.; Bryant, S. *Int. J. Mol. Sci.* **2020**, *21*, 6898.

[25] See previous footnote.

Cell-based assays

MTS Assay

The compounds were evaluated for their antiproliferative activities against the SKBr3 (ATCC HTB-30) breast cancer cell line using an MTS (Promega, Madison, WI, USA) assay according to the manufacturer's instructions. Cells were cultured in McCoy's 5A medium (Sigma - Aldrich, St. Louis, MO, USA) supplemented with 10% fetal bovine serum (Gibco, Thermo Fisher Scientific, Waltham, MA, USA), 100 U/mL penicillin (Sigma-Aldrich, St. Louis, MO, USA), 100 µg/mL streptomycin (Sigma-Aldrich, St. Louis, MO, USA). SKBr3 cells were incubated in a 5% CO_2 atmosphere at 37 °C. Cells were plated out in 96-well plates at a density of 4000 cells per well. Cells were allowed to attach to the plate and were then treated with the compounds, positive control (1 µM 17-DMAG) or vehicle control (0.5% DMSO). After the 72-hour incubation, CellTiter96 Aqueous One Solution Reagent (15 µL; Promega, Madison, WI, USA) was added to each well, and cells were incubated for an additional 3 hours. Absorbance was measured at 492 nm using a microplate reader (Synergy 4 Hybrid; BioTek, Winooski, VT, USA). Independent experiments were repeated twice, each time in triplicate. Statistically significant differences ($p < 0.05$) between treated groups and DMSO were calculated using two-tailed Welch's t-tests. IC50 values were determined using GraphPad Prism 8.0 software (San Diego, CA, USA) and represent the concentration at which a compound produced a half-maximal response; they are expressed as the mean values of the independent measurements.

Luciferase Refolding Assay

The compound was evaluated for its ability to inhibit Hsp90 in PC3MM2*luc*, metastatic prostate cancer cell line expressing firefly luciferase. Cells were cultured in Dulbecco's modified Eagle's medium, high glucose (Gibco, Thermo Fisher Scientific, Waltham, MA, USA) supplemented with 5 µg/mL puromycin (InvivoGen, San Diego, CA, USA), 100 U/mL penicillin (Sigma-Aldrich, St. Louis, MO, USA) and 100 µg/mL streptomycin (Sigma-Aldrich, St. Louis, MO, USA) and 10% fetal bovine serum (Gibco, Thermo Fisher Scientific, Waltham, MA, USA), at 37 °C and below 5% CO_2. Cell pellets were suspended in prewarmed medium (50°C) for 2 min to induce firefly luciferase unfolding. The cells were plated in 96-well plates at a density of 50,000 cells per well in the presence of selected compounds, vehicle control (1% DMSO) or positive control (50 µM geldanamycin). The plates were then incubated for 60 min at 37 °C to allow for luciferase refolding, 100 µL of ONE-Glo™ Luciferase Assay System (Promega, Madison, WI, USA) was then added to each well of the plate and incubated for another 5 min. Luciferase activity was determined by measuring luminescence with BioTek's Synergy™ 4 Hybrid Microplate Reader (Winooski, VT, USA). Independent experiments were repeated two times, each performed in triplicate. Statistical significance ($p < 0.05$) was calculated with two-tailed Welch's t-test between treated groups and DMSO. IC_{50} values (concentration of the inhibitor that gives a half-maximal response) are given as average values from the independent measurements, and were determined using GraphPad Prism 8.0 software (San Diego, CA, USA).

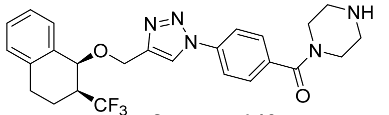

Compound 10

MTS assay	Luciferase refolding assay
**IC_{50} = 51 ± 2 µM**	**IC_{50} = 139 ± 10**
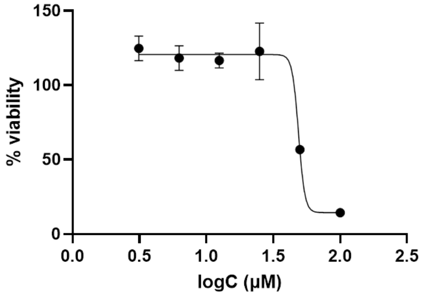	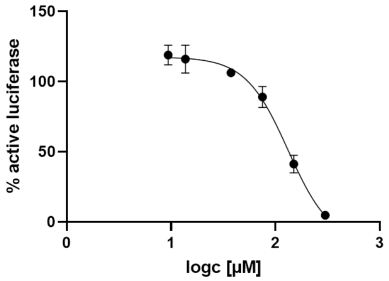

Single crystal X-ray analysis

Selected crystals attached to MiTeGen MicroLoops or MicroMounts with the aid of silicone grease (Bayer) were measured on a Rigaku OD XtaLAB Synergy-S dual source microfocus Ag/Cu PhotonJet-S diffractometer with a four-circle kappa-goniometer and an Eiger2 R CdTe 1M hybrid pixel array detector. During the measurement crystals were kept at 100 K by a cold nitrogen stream of an Oxford Cryosystems Cryostream 800 cooling device. *CrysAlis^{Pro}* software[26] was used for data collection and processing, which included an empirical absorption correction using spherical harmonics, implemented in *SCALE3 ABSPACK*, and a numerical absorption correction based on gaussian integration over a multifaceted crystal model. *Olex2* (v. 1.5)[27] was used for crystal structure solution and refinement (full-matrix least-squares minimization) employing *SHELXT*[28] and *SHELXL*[29] (v. 2019/2), respectively. Molecular graphics were created with *Diamond*[30] program.

Hydrogen atoms were refined freely including their isotropic thermal parameter *U*.[31] When free refinement resulted in either unrealistically small or large U_{iso} value, this was constrained to be equal to 1.2U_{eq} value of the atom to which the hydrogen atom is bonded. In the crystal structure of (*R*)-**4d** the free refinement of H1 atom resulted in a very short O1−H1 distance (0.66(6) Å), therefore a DFIX 0.84 Å restraint was employed. In the crystal structure of **2k** hydrogen atoms bonded to carbon atoms were included at calculated positions and refined using a riding model. This was necessary in order to reduce the number of refined parameters and improve the data-to-parameter ratio, because the asymmetric unit of the **2k** crystal structure (*P*1) contains 4 molecules. The hydrogen atoms of hydroxyl groups were refined freely with isotropic thermal parameters. Similarly, a riding model was used for carbon-bonded hydrogen atoms in crystal structure of **2o** ($P2_1$ polymorph, measured at room temperature), whereas the hydroxyl hydrogen atoms were refined semi-freely with O1−H1 distance restrained (DFIX) to 0.82 Å (free refinement yields 0.70(7) Å) and the U_{iso} value of the H2A atom was constrained to be equal to 1.2U_{eq} of the O2 atom.

The crystal structure of (*R*)-**4d**, (*R*)-4-bromo-1-indanol, was previously published[32a] (CSD refcode: VOFJEI; deposition number: 1897357) and the reported structural parameters ($P2_1$; *a* = 8.0451(4) Å; *b* = 5.1179(2)Å; *c* = 10.6130(6) Å; *β* = 107.945(2)°; *V* = 415.72(4) Å^3;) are in good agreement with the present data (cf. Table S2). The main differences between the two crystal structure determinations are in the measurement temperature (room temperature 293 K vs 100 K) and in the refined position of the hydroxyl hydrogen atom involved in the intermolecular hydrogen bonding (Figure S5, Table S4). Moreover, also the crystal structure of racemic **4d** has been reported[32b] (CSD refcode: VIYZAG; deposition number: 926556; $P2_1/c$; *a* = 12.7914(9) Å; *b* = 4.6949(4)Å; *c* = 27.864(2) Å; *β* = 94.707(1)°; *V* = 1667.7(2) Å^3; *Z* = 8; *Z′* = 2.

Details of single-crystal X-ray diffraction data collection and refinement pertaining to the compounds **2a**, **2d**, (*R*)-**4d**, **2f**, **2k**, **2n**, **2o** ($P2_12_12_1$), **2o** ($P2_1$), and **2p** are summarized in Table S2, bond distances are collected in Table S3, and hydrogen-bond geometries are given in Table S4. The molecular structures of asymmetric units are depicted in Figures S3–S11, the hydrogen-bonded chains in Figures S12–S20, and crystal packings in Figures S29–S21.

All crystals were also measured at room temperature and only the compound **2o**, crystallizing as plastically flexible needles, was found to exhibit temperature-induced single-crystal-to-single-crystal transition in the range 200–230 K with low-temperature orthorhombic $P2_12_12_1$ (*Z′* = 1) crystal structure and room-temperature monoclinic $P2_1$ (*Z′* = 2) polymorph (cf. Table S2, Figures S9, S10, S18, S19, S26, S27).

---

26 *CrysAlisPro* Software System, v. 171.41_64.120a/123a, Rigaku Oxford Diffraction, Rigaku Oxford Diffraction, Rigaku Corporation, The Woodlands, TX, USA, **1995–2022**.

27 Dolomanov, O. V.; Bourhis, L. J.; Gildea, R. J.; Howard, J. A. K.; Puschmann, H. *J. Appl. Cryst.* **2009**, *42*, 339–341.

28 Sheldrick, G. M. *Acta Crystallogr.* **2015**, *A71*, 3–8.

29 Sheldrick, G. M. *Acta Crystallogr.* **2015**, *C71*, 3–8.

30 Brandenburg, K. *Diamond* – Crystal and Molecular Structure Visualization, v. 3.1, Crystal Impact GbR, Bonn, Germany, **1997–2005**.

31 Cooper, R. I.; Thompson, A. L.; Watkin D. J. *J. Appl. Cryst.* **2010**, *43*, 1100–1107.

32 a) Zhang, L.; Tang, Y.; Han, Z.; Ding, K. *Angew. Chem. Int. Ed.* **2019**, *58*, 4973–4977. b) Aldeborgh, H.; George, K.; Howe, M.; Lowman, H.; Moustakas, H.; Strunsky, N.; Tanski, J. M. *J Chem Crystallogr* **2014**, *44*, 70–81.

Table S2. Summary of crystal data and structure refinements.

**Compound**	**2a**	**2d**	(*R*)-**4d**
Formula	$C_{10}H_9F_3O$	$C_{10}H_8BrF_3O$	C_9H_9BrO
F_w	202.17	281.07	213.07
T [K]	100.0(1)	100.0(1)	100.0(1)
Crystal system	Monoclinic	Orthorhombic	Monoclinic
Space group	$P2_1$	$P2_12_12_1$	$P2_1$
a [Å]	8.4643(2)	4.91090(10)	7.96702(7)
b [Å]	4.85610(10)	8.46270(10)	5.06354(4)
c [Å]	10.7964(2)	24.3005(3)	10.41269(9)
α [°]	90	90	90
β [°]	103.146(2)	90	106.7602(9)
γ [°]	90	90	90
V [Å^3]	432.140(16)	1009.92(3)	402.217(6)
Z	2	4	2
ρ_{calc} [g/cm^3]	1.554	1.849	1.759
Crystal size [mm]	0.664 × 0.060 × 0.035	0.759 × 0.047 × 0.027	0.092 × 0.066 × 0.060
Radiation type	Cu $K\alpha$	Cu $K\alpha$	Cu $K\alpha$
λ [Å]	1.54184	1.54184	1.54184
μ [mm^{-1}]	1.251	5.744	6.424
F(000)	208	552	212
ϑ_{max} [°]	76.285	76.223	76.106
Index ranges	$-10 \le h \le 10$	$-5 \le h \le 6$	$-10 \le h \le 10$
	$-6 \le k \le 6$	$-10 \le k \le 10$	$-6 \le k \le 6$
	$-13 \le l \le 13$	$-30 \le l \le 30$	$-13 \le l \le 13$
Reflections collected	24802 [a]	42924	38787
Independent reflections	3398	2086	1672
Reflections with [$I > 2\sigma(I)$]	3353	2058	1656
R_{int}	0.0450 [a]	0.0753	0.0377
R_{sigma}	0.0096	0.0184	0.0112
Data/restraints/parameters	3398/1/164	2086/0/167	1672/2/133
S	1.059	1.130	1.171
R_1, wR_2 [$I > 2\sigma(I)$]	0.0258, 0.0686	0.0398, 0.1019	0.0226, 0.0579
R_1, wR_2 [all data]	0.0261, 0.0688	0.0403, 0.1022	0.0228, 0.0580
$\Delta\rho_{min}$, $\Delta\rho_{max}$ [eÅ^{-3}]	− 0.184, 0.158	−0.748, 0.670	−0.488, 0.343
Flack x	0.03(8)	0.014(11)	−0.012(14)
CCDC deposition number [b]	2151748	2151749	2151750

[a] The crystals of compound **2a** were twinned (HKLF5 refinement, BASF 0.44)

[b] The supplementary crystallographic data for this paper can be obtained free of charge from The Cambridge Crystallographic Data Centre (CCDC) via www.ccdc.cam.ac.uk/structures.

Table S2. Continued …

Compound	2f	2k	2n
Formula	$C_{11}H_8F_6O$	$C_{12}H_{13}F_3O_2$	$C_{10}H_9F_3OS$
F_w	270.17	246.22	234.23
T [K]	100.0(1)	100.0(1)	100.0(1)
Crystal system	Orthorhombic	Triclinic	Orthorhombic
Space group	$P2_12_12_1$	$P1$	$P2_12_12_1$
a [Å]	4.89180(10)	8.32250(10)	4.84350(5)
b [Å]	12.0353(2)	12.15340(10)	9.96451(9)
c [Å]	17.8513(4)	12.28870(10)	21.0970(2)
α [°]	90	68.7720(10)	90
β [°]	90	70.5330(10)	90
γ [°]	90	89.4960(10)	90
V [Å^3]	1050.98(4)	1083.50(2)	1018.206(16)
Z	4	4	4
ρ_{calc} [g/cm^3]	1.707	1.509	1.528
Crystal size [mm]	0.235 × 0.033 × 0.029	0.067 × 0.056 × 0.030	0.484 × 0.151 × 0.097
Radiation type	Cu $K\alpha$	Cu $K\alpha$	Cu $K\alpha$
λ [Å]	1.54184	1.54184	1.54184
μ [mm^{-1}]	1.631	1.177	3.014
F(000)	544	512	480
ϑ_{max} [°]	76.300	76.248	76.049
Index ranges	$-5 \leq h \leq 6$	$-10 \leq h \leq 10$	$-6 \leq h \leq 6$
	$-15 \leq k \leq 15$	$-15 \leq k \leq 15$	$-12 \leq k \leq 12$
	$-22 \leq l \leq 22$	$-14 \leq l \leq 15$	$-26 \leq l \leq 26$
Reflections collected	25297	67652	30809
Independent reflections	2177	8663	2114
Reflections with [$I > 2\sigma(I)$]	2132	8388	2102
R_{int}	0.0652	0.0380	0.0456
R_{sigma}	0.0224	0.0217	0.0137
Data/restraints/parameters	2177/0/194	8663/3/634	2114/0/173
S	1.087	1.026	1.056
R_1, wR_2 [$I > 2\sigma(I)$]	0.0310, 0.0766	0.0280, 0.0711	0.0219, 0.0563
R_1, wR_2 [all data]	0.0323, 0.0775	0.0292, 0.0716	0.0220, 0.0564
$\Delta\rho_{min}$, $\Delta\rho_{max}$ [eÅ^{-3}]	−0.194, 0.291	−0.185, 0.250	−0.185, 0.219
Flack x	0.05(6)	0.02(3)	−0.010(5)
CCDC deposition number [b]	2151751	2151752	2151753

Table S2. Continued …

**Compound**	**2o** ($P2_12_12_1$)	**2o** ($P2_1$)	**2p**
Formula	$C_{11}H_{11}F_3OS$	$C_{11}H_{11}F_3OS$	$C_{10}H_9F_3O_2$
F_w	248.26	248.26	218.17
T [K]	100.0(1)	296(2)	100.0(1)
Crystal system	Orthorhombic	Monoclinic	Monoclinic
Space group	$P2_12_12_1$	$P2_1$	$P2_1$
a [Å]	5.12413(3)	5.16870(10)	10.1028(2)
b [Å]	10.11484(6)	21.8616(6)	4.75350(10)
c [Å]	20.63850(14)	10.1340(2)	10.2459(2)
α [°]	90	90	90
β [°]	90	90.687(2)	104.328(2)
γ [°]	90	90	90
V [Å^3]	1069.689(11)	1145.02(4)	476.740(17)
Z	4	4	2
ρ_{calc} [g/cm^3]	1.542	1.440	1.520
Crystal size [mm]	0.611 × 0.061 × 0.039	1.074 × 0.077 × 0.066	0.497 × 0.040 × 0.036
Radiation type	Cu $K\alpha$	Cu $K\alpha$	Cu $K\alpha$
λ [Å]	1.54184	1.54184	1.54184
μ [mm^{-1}]	2.903	2.712	1.261
F(000)	512	512	224
ϑ_{max} [°]	76.216	76.161	76.022
Index ranges	$-6 \le h \le 6$	$-6 \le h \le 5$	$-12 \le h \le 12$
	$-12 \le k \le 12$	$-27 \le k \le 26$	$-5 \le k \le 5$
	$-25 \le l \le 23$	$-12 \le l \le 12$	$-12 \le l \le 12$
Reflections collected	90181	12515	17644
Independent reflections	2236	4429	1956
Reflections with [$I > 2\sigma(I)$]	2223	3830	1922
R_{int}	0.0659	0.0368	0.0370
R_{sigma}	0.0114	0.0395	0.0131
Data/restraints/parameters	2236/0/189	4429/2/296	1956/1/173
S	1.054	1.064	1.034
R_1, wR_2 [$I > 2\sigma(I)$]	0.0226, 0.0574	0.0534, 0.1552	0.0273, 0.0719
R_1, wR_2 [all data]	0.0227, 0.0575	0.0597, 0.1614	0.0277, 0.0722
$\Delta\rho_{min}$, $\Delta\rho_{max}$ [eÅ^{-3}]	−0.212, 0.233	−0.278, 0.323	−0.160, 0.182
Flack x	−0.007(5)	−0.04(2)	−0.09(5)
CCDC deposition number [b]	2151754	2155509	2151755

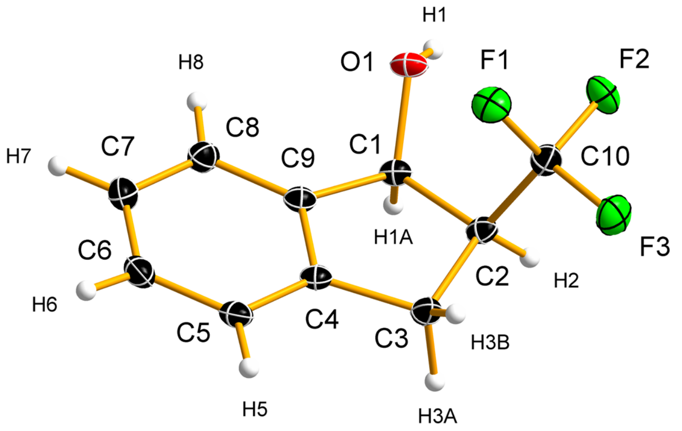

Figure S3. The atom numbering scheme and the asymmetric unit of the crystal structure of compound **2a**. Thermal ellipsoids are plotted at the 50% probability level and hydrogen atoms are depicted as small spheres of arbitrary radius.

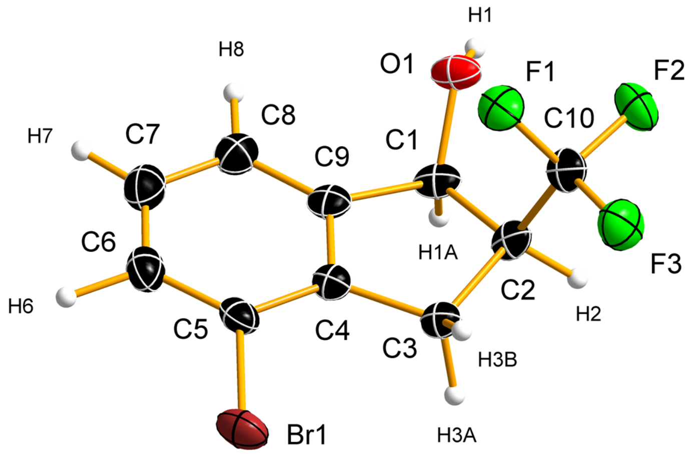

Figure S4. The atom numbering scheme and the asymmetric unit of the crystal structure of compound **2d**. Thermal ellipsoids are plotted at the 50% probability level and hydrogen atoms are depicted as small spheres of arbitrary radius.

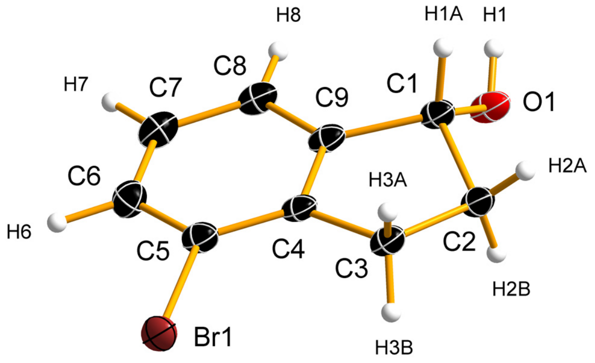

Figure S5. The atom numbering scheme and the asymmetric unit of the crystal structure of compound (*R*)-**4d**. Thermal ellipsoids are plotted at the 50% probability level and hydrogen atoms are depicted as small spheres of arbitrary radius.

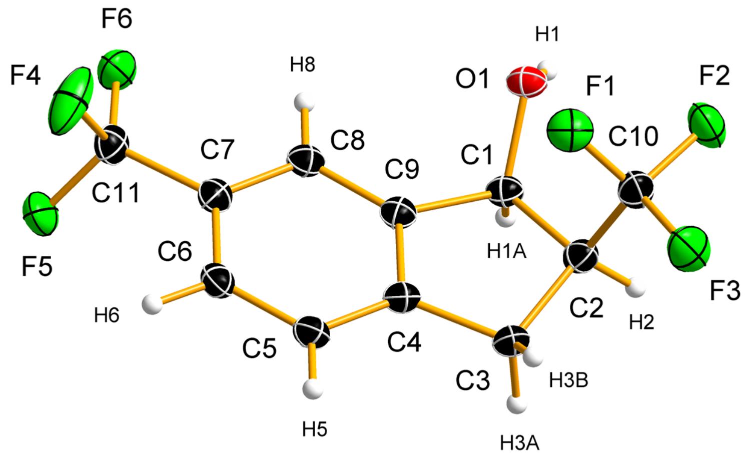

Figure S6. The atom numbering scheme and the asymmetric unit of the crystal structure of compound **2f**. Thermal ellipsoids are plotted at the 50% probability level and hydrogen atoms are depicted as small spheres of arbitrary radius.

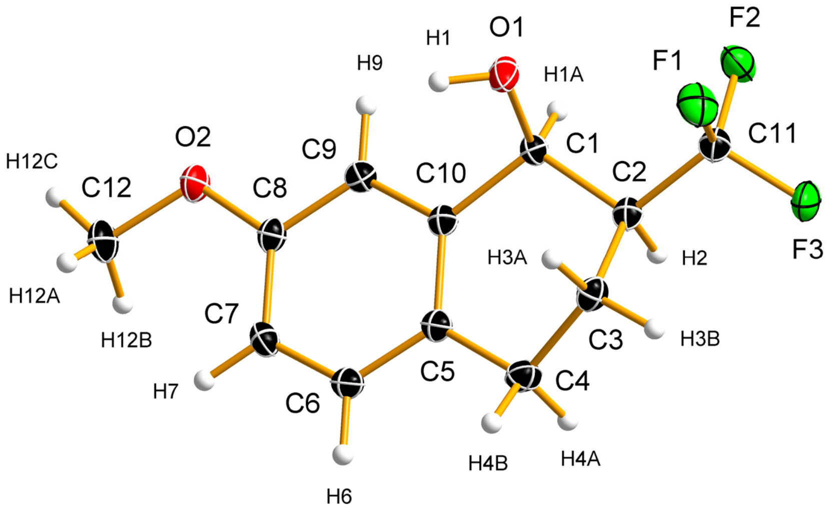

Figure S7. One of the four molecules of the asymmetric unit ($P1$, $Z = 4$) of the crystal structure of compound **2k**. Thermal ellipsoids are plotted at the 50% probability level and hydrogen atoms are depicted as small spheres of arbitrary radius.

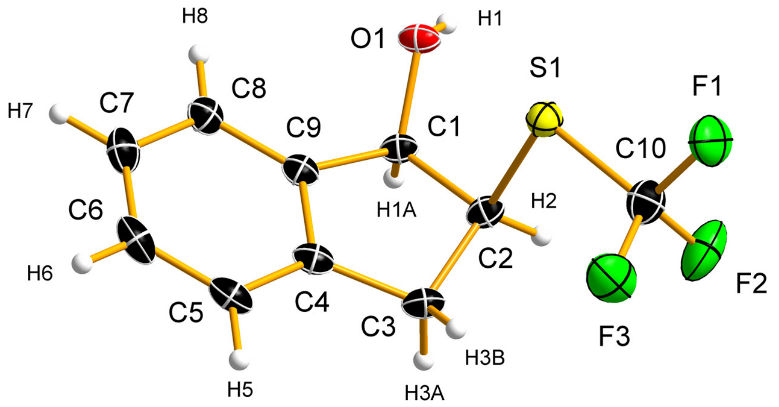

Figure S8. The atom numbering scheme and the asymmetric unit of the crystal structure of compound **2n**. Thermal ellipsoids are plotted at the 50% probability level and hydrogen atoms are depicted as small spheres of arbitrary radius

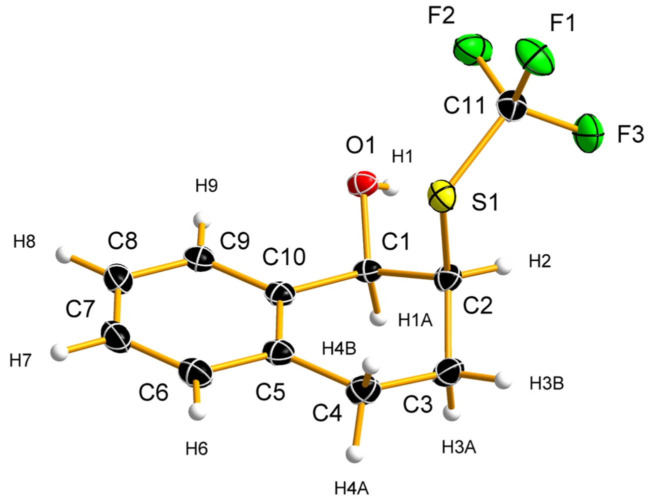

Figure S9. The atom numbering scheme and the asymmetric unit of the crystal structure of compound **2o** ($P2_12_12_1$). Thermal ellipsoids are plotted at the 50% probability level and hydrogen atoms are depicted as small spheres of arbitrary radius.

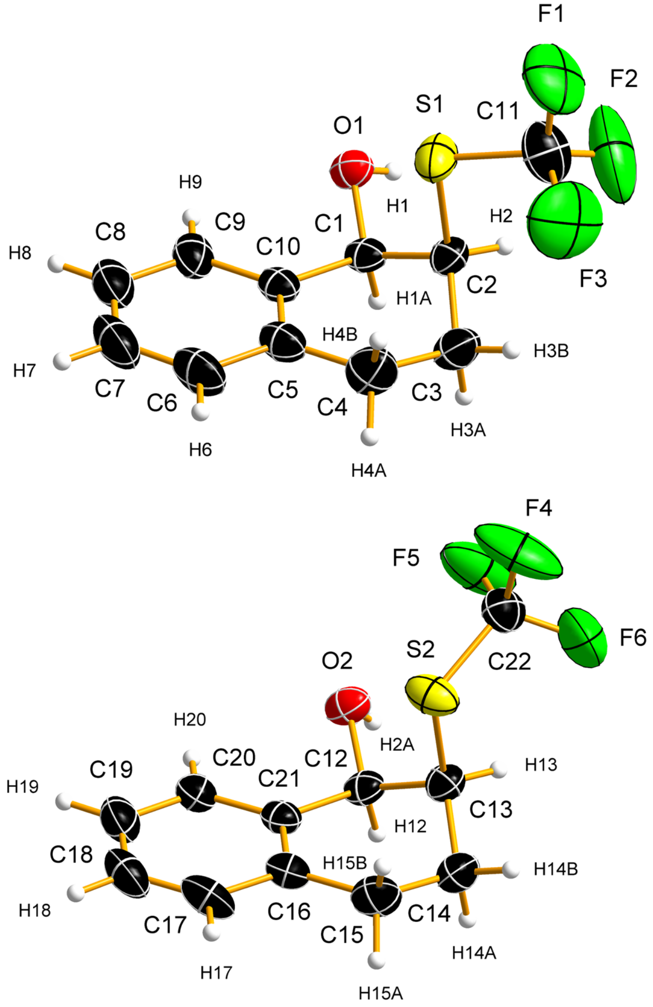

Figure S10. The atom numbering scheme and the asymmetric unit of the crystal structure of compound **2o** ($P2_1$) is comprised of two molecules (Z' = 2). Thermal ellipsoids are plotted at the 30% probability level and hydrogen atoms are depicted as small spheres of arbitrary radius. The conformational difference between the two molecules is the orientation of the S−CF_3 group (cf. Figure S9).

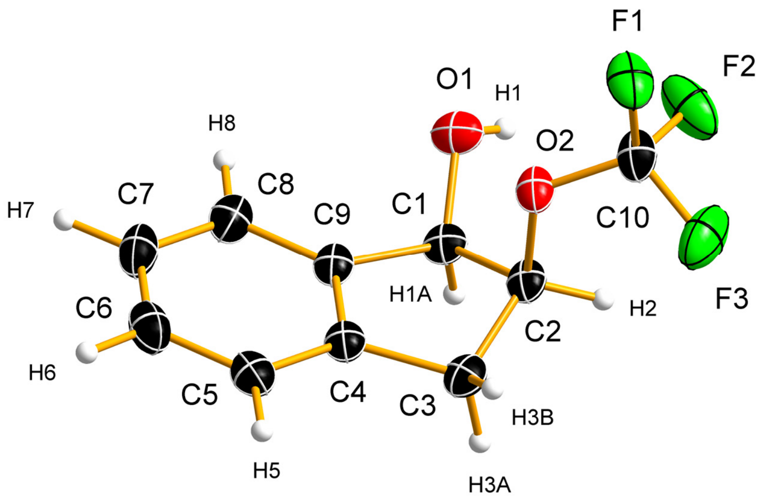

Figure S11. The atom numbering scheme and the asymmetric unit of the crystal structure of compound **2p**. Thermal ellipsoids are plotted at the 50% probability level and hydrogen atoms are depicted as small spheres of arbitrary radius.

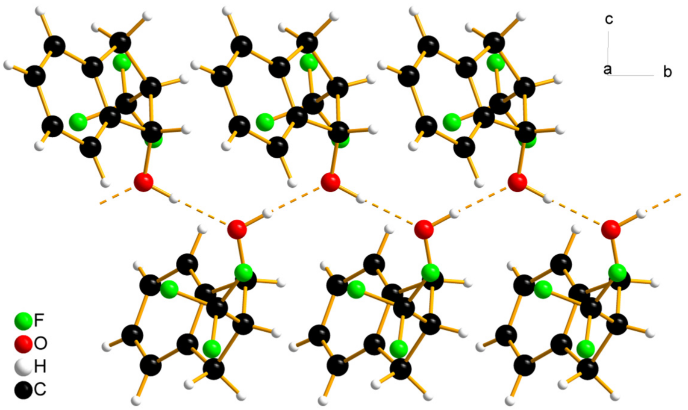

Figure S12. The O−H···O hydrogen-bonded chains in the crystal structure of **2a**, connect the molecules along the *b*-crystallographic axis.

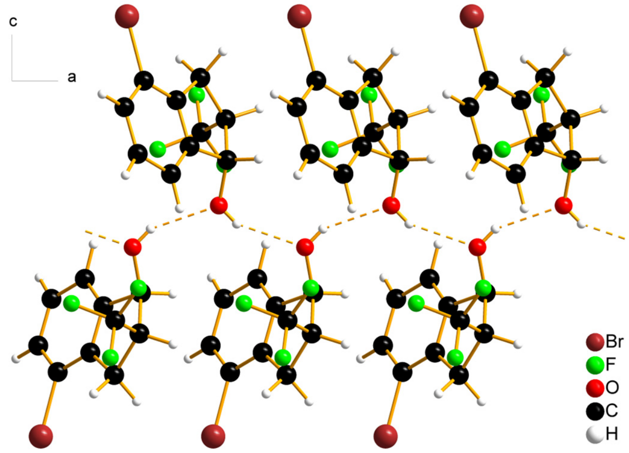

Figure S13. The O−H···O hydrogen-bonded chains in the crystal structure of **2d**, connect the molecules along the *a*-crystallographic axis.

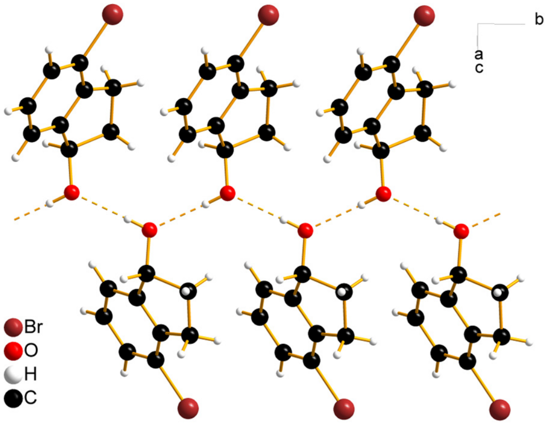

Figure S14. The O−H···O hydrogen-bonded chains in the crystal structure of (*R*)-**4d**, connect the molecules along the *b*-crystallographic axis.

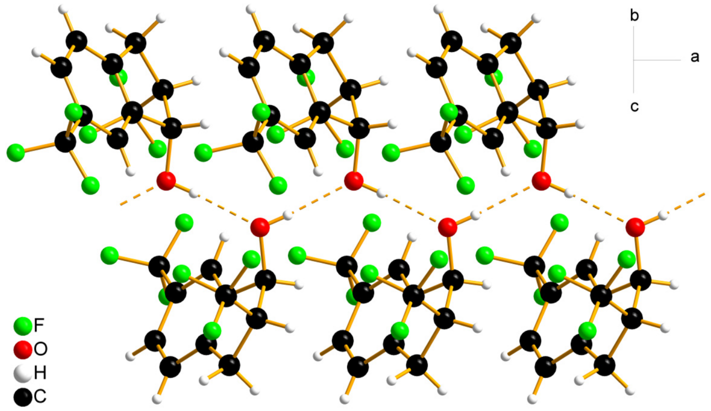

Figure S15. The O−H···O hydrogen-bonded chains in the crystal structure of **2f**, connect the molecules along the *a*-crystallographic axis.

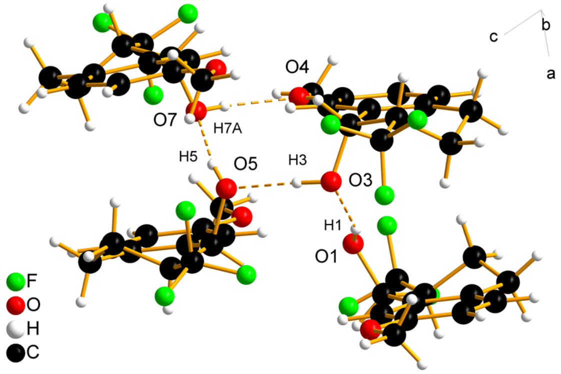

Figure S16. The molecules of the asymmetric unit (*P*1, *Z* = 4) in the crystal structure of **2k** form an O−H···O hydrogen-bonded tetramer. Atoms involved in hydrogen bonds are labelled.

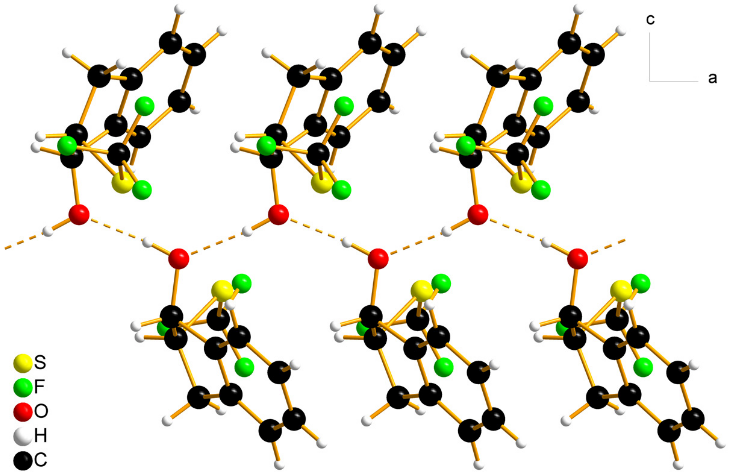

Figure S17. The O−H···O hydrogen-bonded chains in the crystal structure of **2n**, connect the molecules along the *a*-crystallographic axis.

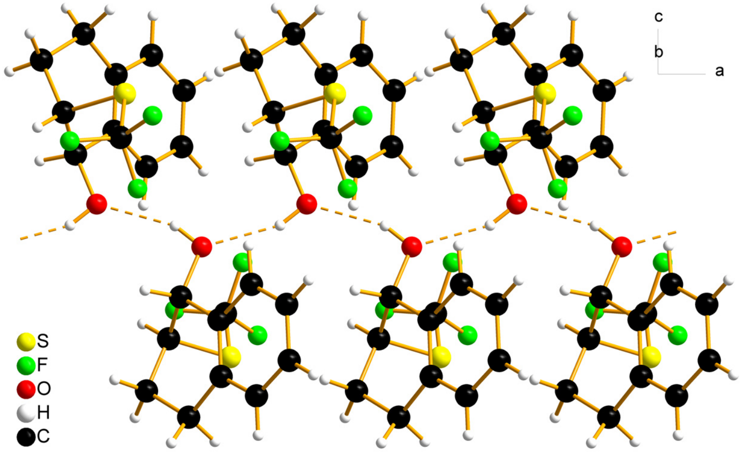

Figure S18. The O−H···O hydrogen-bonded chains in the crystal structure of **2o** ($P2_12_12_1$), connect the molecules along the *a*-crystallographic axis.

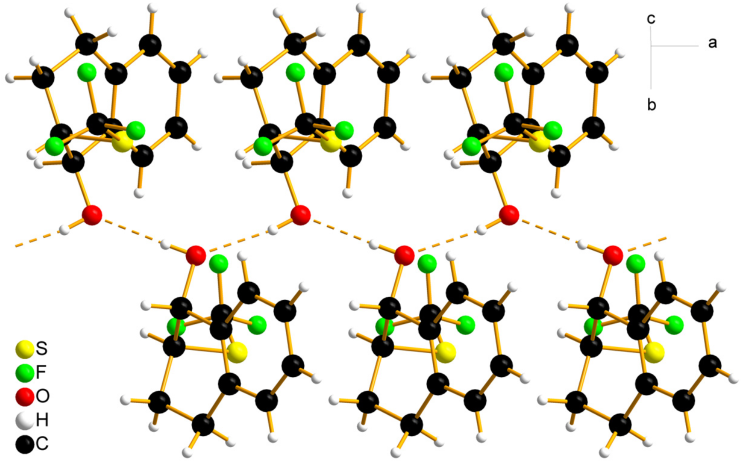

Figure S19. The O−H···O hydrogen-bonded chains in the crystal structure of **2o** ($P2_1$), connect the molecules along the *a*-crystallographic axis.

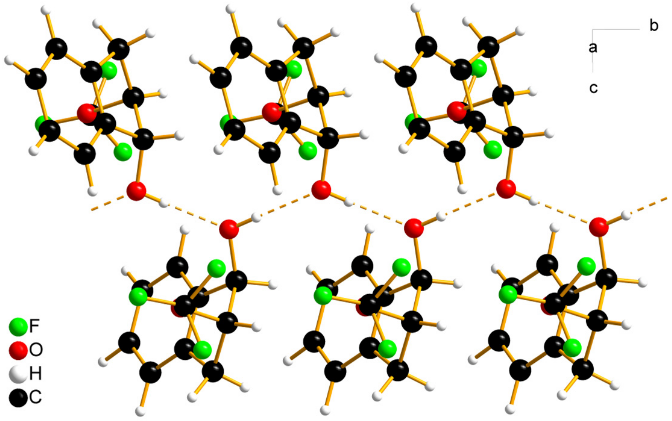

Figure S20. The O−H···O hydrogen-bonded chains in the crystal structure of **2p**, connect the molecules along the *b*-crystallographic axis.

Table S3. Selected bond distances (Å) in crystal structures **2a**, **2d**, (*R*)-**4d**, **2f**, **2k**, **2n**, **2o** ($P2_12_12_1$), **2o** ($P2_1$), **2p**

**Bond Lengths** [Å]							
**2a**							
F1−C10	1.344(3)	C1−C2	1.569(2)	C3−H3B	0.98(3)	C6−H6	0.91(3)
F2−C10	1.338(2)	C1−C9	1.506(3)	C3−C4	1.508(3)	C6−C7	1.398(3)
F3−C10	1.361(2)	C2−H2	0.93(3)	C4−C5	1.391(3)	C7−H7	0.94(3)
O1−H1	0.90(4)	C2−C3	1.557(3)	C4−C9	1.391(3)	C7−C8	1.389(3)
O1−C1	1.421(2)	C2−C10	1.508(3)	C5−H5	1.01(3)	C8−H8	1.00(3)
C1−H1A	1.00(3)	C3−H3A	1.04(3)	C5−C6	1.392(3)	C8−C9	1.392(3)
**2d**							
Br1−C5	1.906(5)	C1−C2	1.565(8)	C3−H3B	1.00(7)	C6−H6	1.12(9)
F1−C10	1.349(7)	C1−H1A	1.04(8)	C3−H3A	1.02(9)	C6−C7	1.391(9)
F2−C10	1.341(6)	C1−C9	1.517(8)	C3−C4	1.507(7)	C7−H7	0.99(8)
F3−C10	1.344(7)	C2−H2	1.08(7)	C4−C5	1.387(7)	C7−C8	1.391(9)
O1−C1	1.418(6)	C2−C3	1.554(7)	C4−C9	1.380(7)	C8−H8	1.02(7)
O1−H1	0.74(11)	C2−C10	1.498(8)	C5−C6	1.378(8)	C8−C9	1.384(8)
(*R*)-**4d**							
Br1−C5	1.906(4)	C2−H2A	0.93(4)	C3−C4	1.510(6)	C6−C7	1.386(6)
O1−H1	0.81(3)	C2−H2B	0.92(6)	C4−C5	1.382(6)	C7−H7	0.93(6)
O1−C1	1.431(5)	C2−C3	1.544(4)	C4−C9	1.388(5)	C7−C8	1.395(6)
C1−C2	1.535(8)	C3−H3A	0.93(5)	C5−C6	1.388(6)	C8−H8	0.95(6)
C1−H1A	1.00(6)	C3−H3B	0.96(7)	C6−H6	1.02(6)	C8−C9	1.387(6)
C1−C9	1.521(6)						
**2f**							
F1−C10	1.345(3)	O1−C1	1.423(3)	C3−H3B	0.98(3)	C6−C7	1.398(3)
F2−C10	1.335(3)	C1−H1A	0.95(3)	C3−C4	1.509(3)	C7−C8	1.391(3)
F3−C10	1.346(3)	C1−C2	1.559(3)	C4−C5	1.392(3)	C7−C11	1.500(3)
F4−C11	1.325(3)	C1−C9	1.511(3)	C4−C9	1.386(3)	C8−H8	0.94(3)
F5−C11	1.343(3)	C2−H2	0.98(3)	C5−H5	0.93(3)	C8−C9	1.384(3)
F6−C11	1.338(3)	C2−C3	1.551(3)	C5−C6	1.390(3)	C2−C10	1.509(3)
O1−H1	0.79(4)	C3−H3A	0.95(3)	C6−H6	0.98(3)		
**2k**							
F1−C11	1.340(3)	C1−C2	1.534(3)	C4−H4A	0.99	C7−C8	1.393(3)
F2−C11	1.342(3)	C1−C10	1.514(3)	C4−H4B	0.99	C8−C9	1.391(3)
F3−C11	1.362(3)	C2−H2	1.00	C4−C5	1.512(3)	C9−H9	0.95
O1−H1	0.85(4)	C2−C3	1.526(3)	C5−C6	1.389(3)	C9−C10	1.394(3)
O1−C1	1.427(3)	C2−C11	1.506(3)	C5−C10	1.408(3)	C12−H12A	0.98
O2−C8	1.371(3)	C3−H3A	0.99	C6−H6	0.95	C12−H12B	0.98
O2−C12	1.422(3)	C3−H3B	0.99	C6−C7	1.390(3)	C12−H12C	0.98
C1−H1A	1.00	C3−C4	1.526(3)	C7−H7	0.95		
F4−C23	1.337(3)	C13−C14	1.534(3)	C16−H16A	0.99	C19−C20	1.397(3)
F5−C23	1.340(3)	C13−C22	1.516(3)	C16−H16B	0.99	C20−C21	1.389(3)
F6−C23	1.361(3)	C14−H14	1.00	C16−C17	1.519(3)	C21−H21	0.95
O3−H3	0.88(4)	C14−C15	1.525(3)	C17−C18	1.398(3)	C21−C22	1.395(3)
O3−C13	1.433(3)	C14−C23	1.511(3)	C17−C22	1.398(3)	C24−H24A	0.98
O4−C20	1.371(3)	C15−H15A	0.99	C18−H18	0.95	C24−H24B	0.98
O4−C24	1.431(3)	C15−H15B	0.99	C18−C19	1.387(3)	C24−H24C	0.98
C13−H13	1.00	C15−C16	1.529(4)	C19−H19	0.95		

Table S3. Continued …

**Bond Lengths** [Å]							
**2k**							
F7–C35	1.338(3)	C25–C26	1.532(3)	C28–H28A	0.99	C31–C32	1.397(3)
F8–C35	1.342(3)	C25–C34	1.515(3)	C28–H28B	0.99	C32–C33	1.391(3)
F9–C35	1.362(3)	C26–H26	1.00	C28–C29	1.513(3)	C33–H33	0.95
O5–H5	0.90(3)	C26–C27	1.528(3)	C29–C30	1.394(3)	C33–C34	1.396(3)
O5–C25	1.442(3)	C26–C35	1.511(3)	C29–C34	1.408(3)	C36–H36A	0.98
O6–C32	1.369(3)	C27–H27A	0.99	C30–H30	0.95	C36–H36B	0.98
O6–C36	1.431(3)	C27–H27B	0.99	C30–C31	1.387(4)	C36–H36C	0.98
C25–H25	1.00	C27–C28	1.524(3)	C31–H31	0.95		
F10–C47	1.349(3)	C37–C38	1.533(3)	C40–H40A	0.99	C43–C44	1.394(3)
F11–C47	1.342(3)	C37–C46	1.514(3)	C40–H40B	0.99	C44–C45	1.388(3)
F12–C47	1.350(3)	C38–H38	1.00	C40–C41	1.513(3)	C45–H45	0.95
O7–H7A	0.88(4)	C38–C39	1.526(3)	C41–C42	1.392(3)	C45–C46	1.387(3)
O7–C37	1.441(3)	C38–C47	1.504(3)	C41–C46	1.403(3)	C48–H48A	0.98
O8–C44	1.364(3)	C39–H39A	0.99	C42–H42	0.95	C48–H48B	0.98
O8–C48	1.429(3)	C39–H39B	0.99	C42–C43	1.391(3)	C48–H48C	0.98
C37–H37	1.00	C39–C40	1.524(3)	C43–H43	0.95		
**2n**							
S1–C2	1.8334(18)	C1–H1A	0.96(2)	C3–H3B	1.00(2)	C6–H6	0.95(3)
S1–C10	1.794(2)	C1–C2	1.561(2)	C3–C4	1.509(3)	C6–C7	1.391(3)
F1–C10	1.336(2)	C1–C9	1.503(2)	C4–C5	1.391(3)	C7–H7	0.94(3)
F2–C10	1.333(3)	C2–H2	0.98(3)	C4–C9	1.388(2)	C7–C8	1.391(3)
F3–C10	1.336(2)	C2–C3	1.544(2)	C5–H5	0.98(2)	C8–H8	0.93(2)
O1–H1	0.86(3)	C3–H3A	0.96(3)	C5–C6	1.388(3)	C8–C9	1.391(3)
O1–C1	1.4188(19)						
**2o** ($P2_12_12_1$)							
S1–C2	1.8416(19)	C1–H1A	0.99(2)	C3–C4	1.530(3)	C6–C7	1.387(3)
S1–C11	1.793(2)	C1–C2	1.529(3)	C4–H4A	0.97(3)	C7–H7	0.97(2)
F1–C11	1.342(2)	C1–C10	1.515(3)	C4–H4B	1.02(2)	C7–C8	1.392(3)
F2–C11	1.328(2)	C2–H2	0.96(2)	C4–C5	1.510(3)	C8–H8	0.94(3)
F3–C11	1.340(2)	C2–C3	1.527(3)	C5–C6	1.401(3)	C8–C9	1.385(3)
O1–H1	0.84(3)	C3–H3A	0.95(3)	C5–C10	1.403(2)	C9–H9	0.96(2)
O1–C1	1.426(2)	C3–H3B	1.00(2)	C6–H6	0.97(2)	C9–C10	1.398(3)
**2o** ($P2_1$)							
S1–C2	1.834(5)	C1–H1A	0.98	C3–C4	1.536(9)	C6–C7	1.376(13)
S1–C11	1.743(7)	C1–C2	1.516(7)	C4–H4A	0.97	C7–H7	0.93
F1–C11	1.330(11)	C1–C10	1.507(6)	C4–H4B	0.97	C7–C8	1.380(12)
F2–C11	1.260(10)	C2–H2	0.98	C4–C5	1.484(10)	C8–H8	0.93
F3–C11	1.309(12)	C2–C3	1.510(7)	C5–C6	1.407(8)	C8–C9	1.370(9)
O1–H1	0.80(3)	C3–H3A	0.97	C5–C10	1.390(7)	C9–H9	0.93
O1–C1	1.412(5)	C3–H3B	0.97	C6–H6	0.93	C9–C10	1.409(8)
S2–C13	1.842(4)	C12–H12	0.98	C14–C15	1.533(9)	C17–C18	1.340(11)
S2–C22	1.752(6)	C12–C13	1.507(7)	C15–H15A	0.97	C18–H18	0.93
F4–C22	1.298(7)	C12–C21	1.511(6)	C15–H15B	0.97	C18–C19	1.378(10)
F5–C22	1.288(8)	C13–H13	0.98	C15–C16	1.493(9)	C19–H19	0.93
F6–C22	1.287(9)	C13–C14	1.530(7)	C16–C17	1.411(8)	C19–C20	1.376(8)
O2–H2A	0.81(7)	C14–H14A	0.97	C16–C21	1.387(7)	C20–H20	0.93
O2–C12	1.419(5)	C14–H14B	0.97	C17–H17	0.93	C20–C21	1.400(7)

Table S3. Continued …

Bond Lengths [Å]							
**2p**							
F1–C10	1.327(3)	C1–H1A	0.99(3)	C3–H3B	1.02(3)	C6–H6	1.02(3)
F2–C10	1.332(3)	C1–C2	1.540(3)	C3–C4	1.508(3)	C6–C7	1.389(4)
F3–C10	1.337(3)	C1–C9	1.509(3)	C4–C5	1.391(3)	C7–H7	0.98(3)
O1–H1	0.83(5)	C2–H2	1.00(3)	C4–C9	1.390(3)	C7–C8	1.393(3)
O1–C1	1.409(2)	C2–C3	1.527(3)	C5–H5	1.04(2)	C8–H8	0.98(3)
O2–C2	1.466(2)	C3–H3A	0.95(3)	C5–C6	1.393(3)	C8–C9	1.388(3)
O2–C10	1.323(2)						

Table S4. Comparison of intermolecular hydrogen-bond geometry (Å, °) in compounds **2a**, **2d**, (*R*)-**4d**, **2f**, **2k**, **2n**, **2o** ($P2_12_12_1$), **2o** ($P2_1$), and **2p**.

**Compound**	***D*–H···*A***	***D*–H**	**H···*A***	***D*···*A***	***D*–H···*A***
**2a**	O1–H1···O1^{i}	0.90(4)	1.83(4)	2.7219(12)	171(3)
**2d**	O1–H1···O1^{ii}	0.74(11)	2.05(12)	2.734(3)	153(10)
(*R*)-**4d**	O1–H1···O1^{iii}	0.81(3)[a]	2.02(3)	2.818(3)	170(6)
**2f**	O1–H1···O1^{iv}	0.79(4)	1.96(4)	2.7437(14)	175(4)
**2k**	O1–H1···O3	0.85(4)	2.06(4)	2.857(2)	156(3)
	O3–H3···O5	0.88(4)	1.97(4)	2.839(2)	170(4)
	O5–H5···O7	0.90(3)	1.97(4)	2.855(2)	170(3)
	O7–H7A···O4	0.88(4)	1.96(4)	2.808(2)	163(3)
**2n**	O1–H1···O1^{v}	0.86(3)	1.82(3)	2.6751(10)	171(3)
**2o** ($P2_12_12_1$)	O1–H1···O1^{vi}	0.84(3)	2.01(3)	2.8074(10)	157(3)
**2o** ($P2_1$)	O1–H1···O2^{vii}	0.80(3)	2.04(3)	2.832(5)	169(6)
	O2–H2A···O1	0.81(7)	2.04(7)	2.814(5)	161(7)
**2p**	O1–H1···O1^{i}	0.83(5)	1.86(5)	2.6782(13)	168(4)

Symmetry codes:
(i) −*x*+1, *y*+1/2, −*z*+1
(ii) *x*+1/2, −*y*+1/2, −*z*+1
(iii) −*x*+2, *y*−1/2, −*z*+1
(iv) *x*+1/2, −*y*+3/2, −*z*+1
(v) *x*−1/2, −*y*+1/2, −*z*+1
(vi) *x*−1/2, −*y*+3/2, −*z*+1
(vii) *x*−1, *y*, *z*

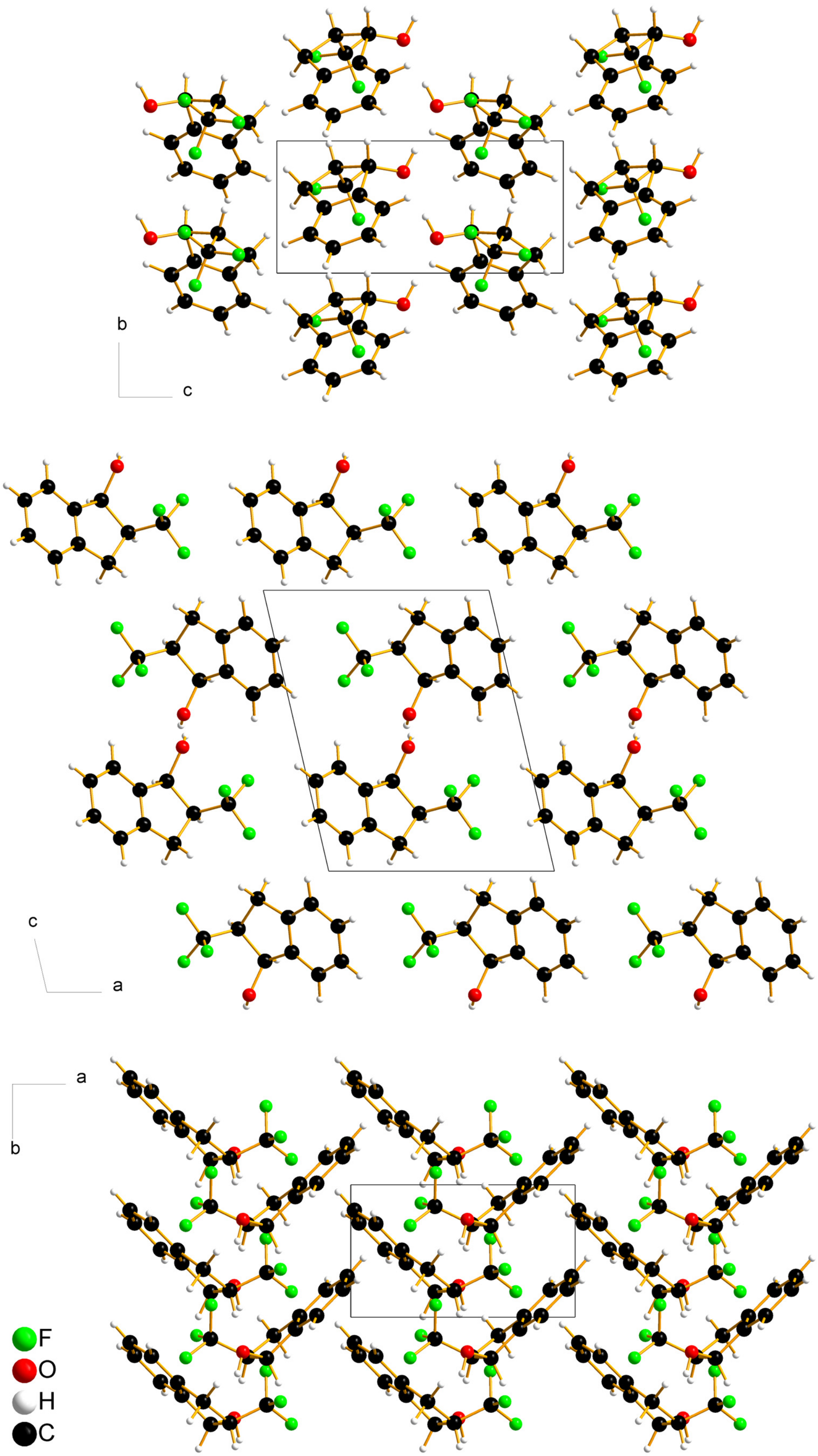

Figure S21. The crystal packing and the unit cell of **2a** crystal structure viewed along the *a*-crystallographic axis (top), *b*-crystallographic axis (middle), and *c*-crystallographic axis (bottom).

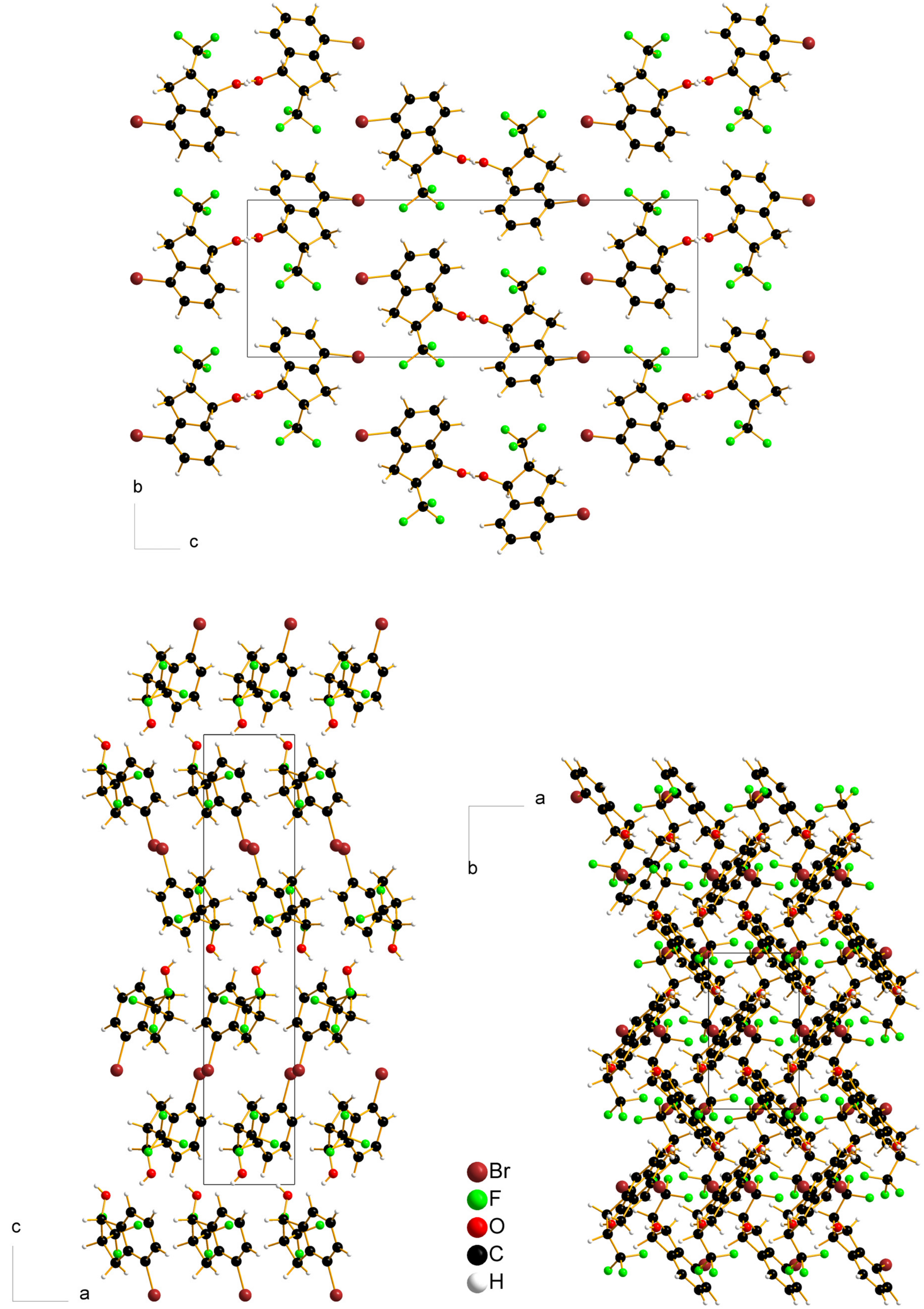

Figure S22. The crystal packing and the unit cell of **2d** crystal structure viewed along the *a*-crystallographic axis (top), *b*-crystallographic axis (middle), and *c*-crystallographic axis (bottom).

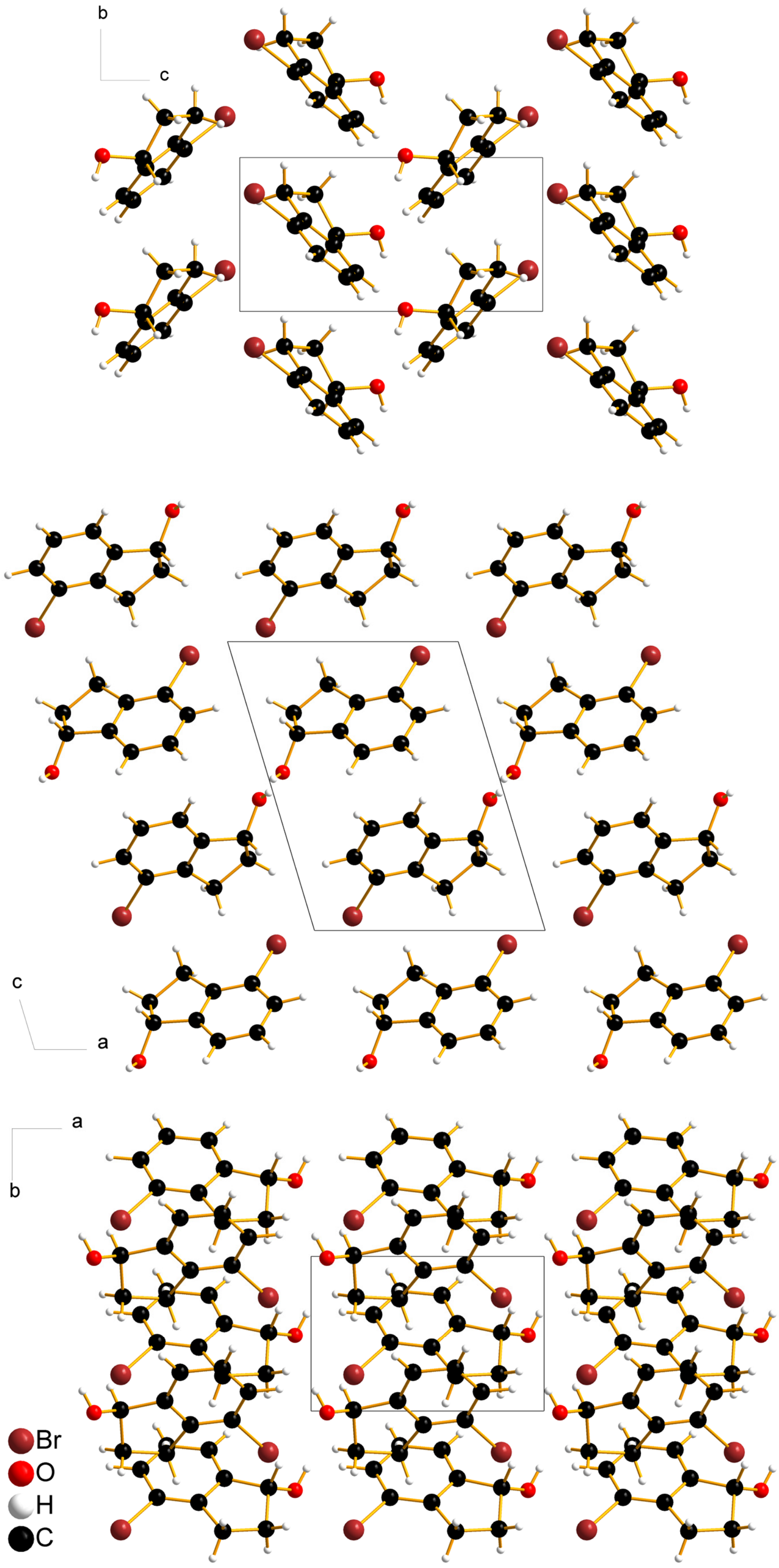

Figure S23. The crystal packing and the unit cell of (*R*)-**4d** crystal structure viewed along the *a*-crystallographic axis (top), *b*-crystallographic axis (middle), and *c*-crystallographic axis (bottom).

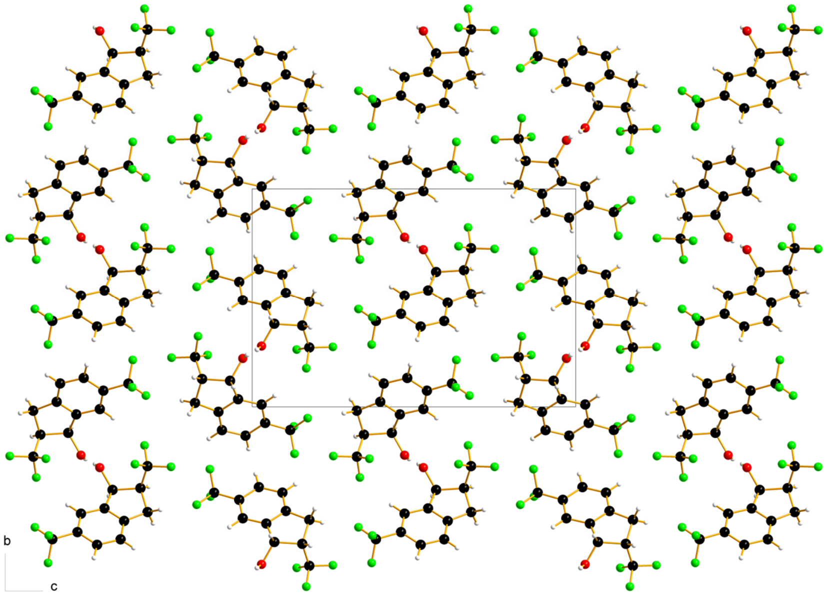

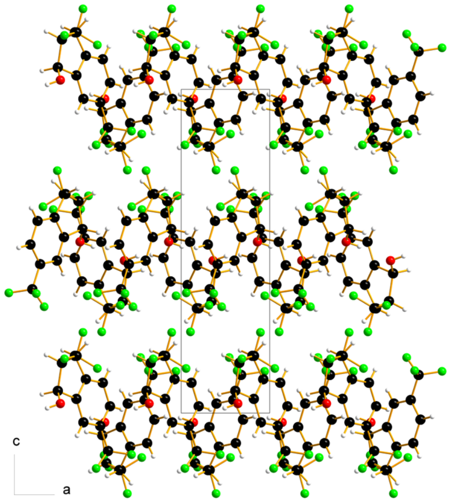

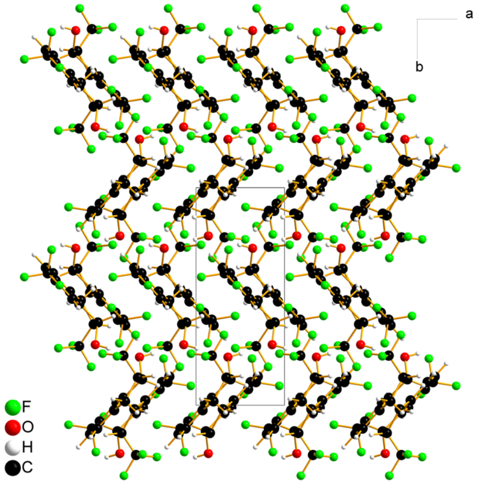

Figure S24. The crystal packing and the unit cell of **2f** crystal structure viewed along the *a*-crystallographic axis (top), *b*-crystallographic axis (middle), and *c*-crystallographic axis (bottom).

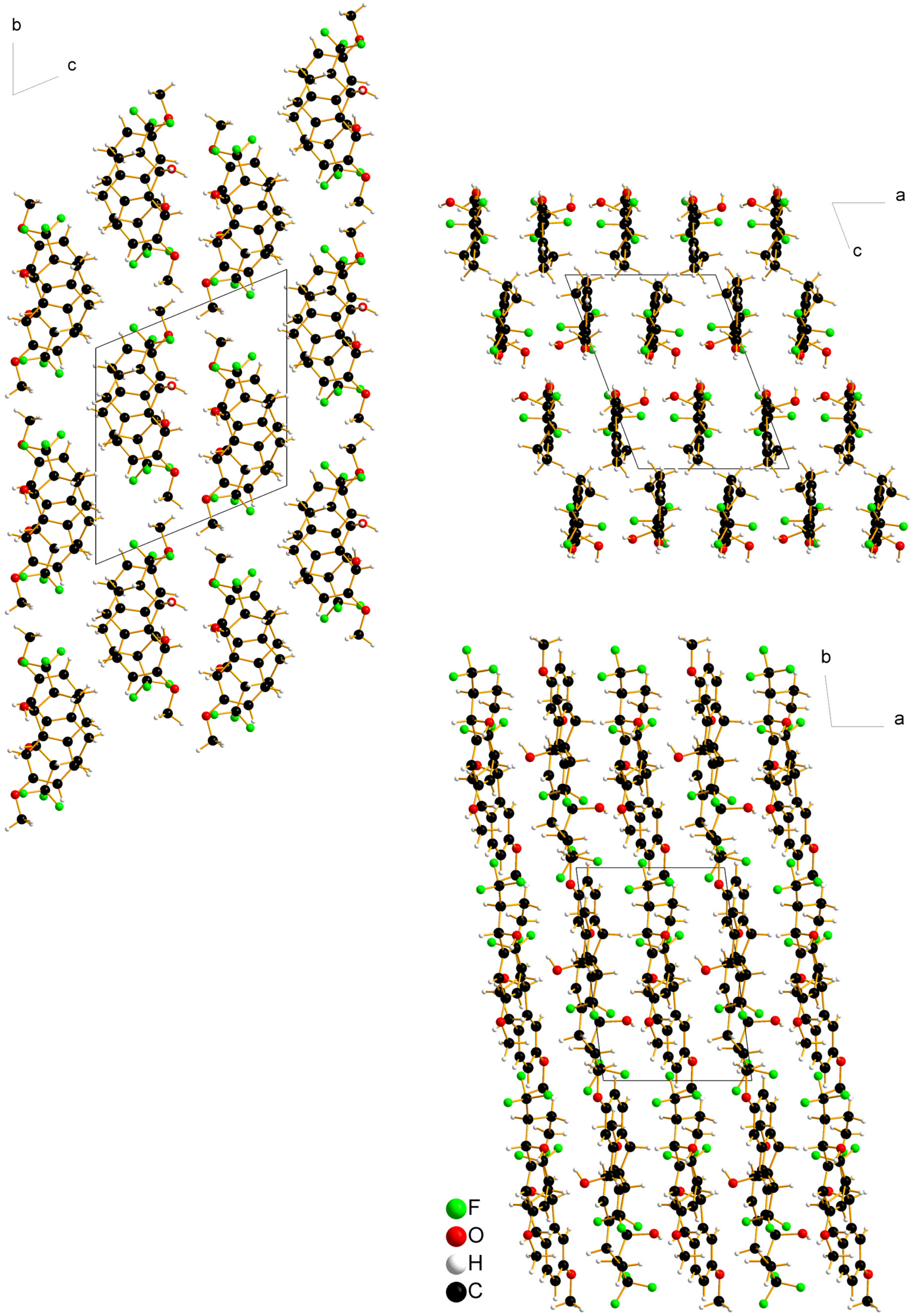

Figure S25. The crystal packing and the unit cell of **2k** crystal structure viewed along the *a*-crystallographic axis (top left), *b*-crystallographic axis (top right), and *c*-crystallographic axis (bottom).

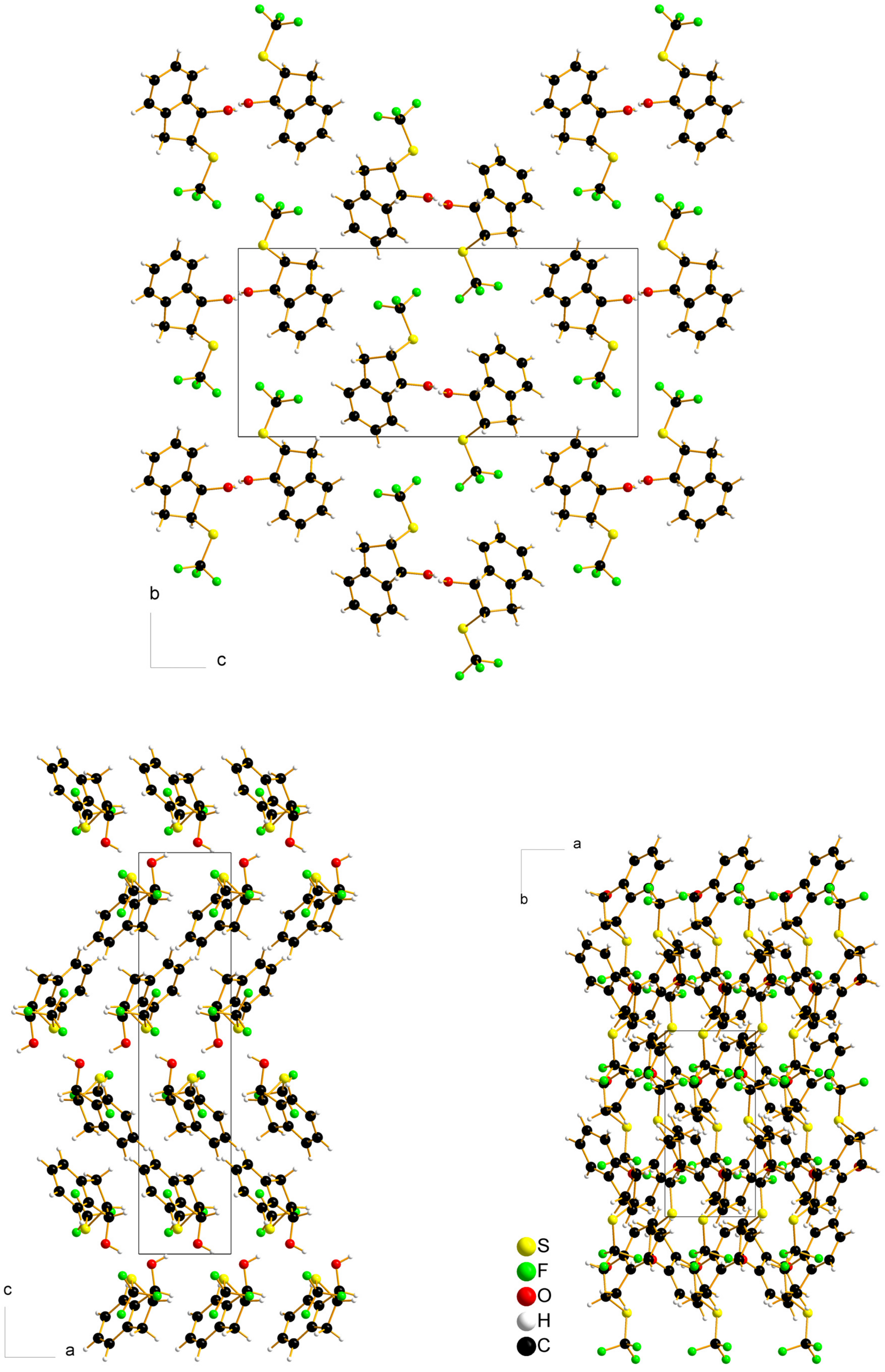

Figure S26. The crystal packing and the unit cell of **2n** crystal structure viewed along the *a*-crystallographic axis (top), *b*-crystallographic axis (bottom left), and *c*-crystallographic axis (bottom right).

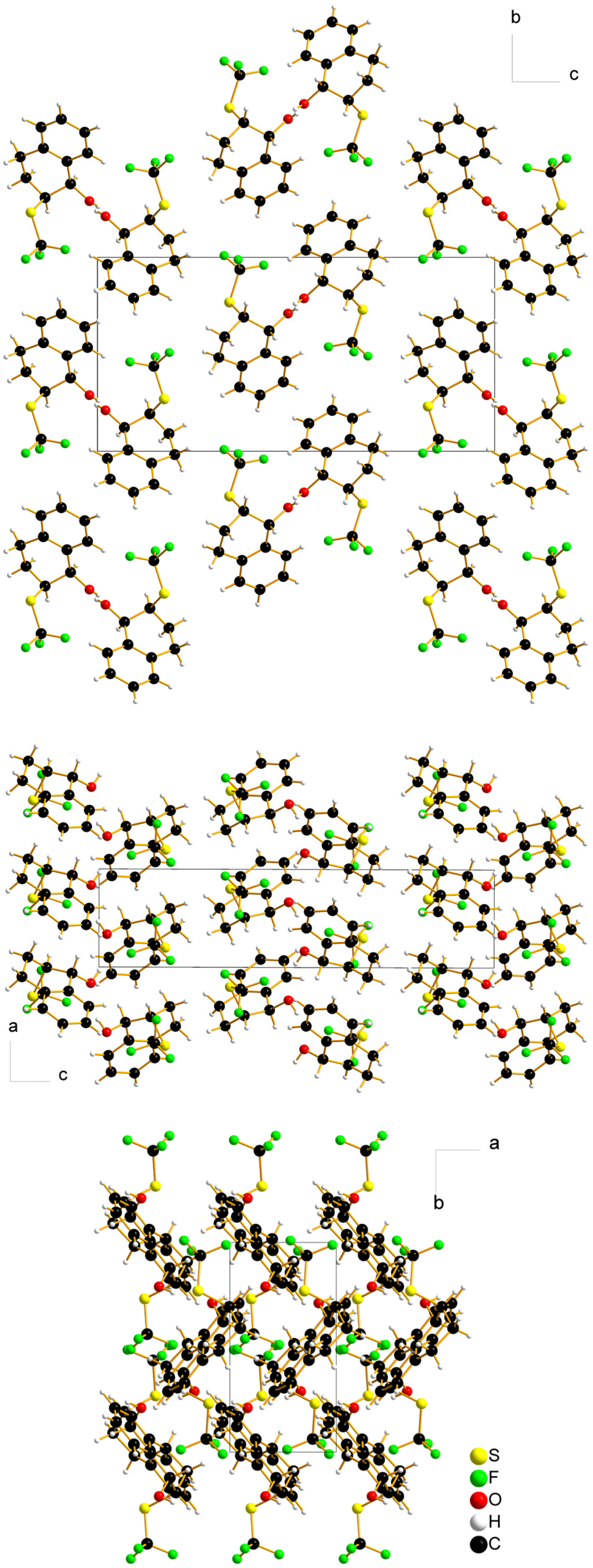

Figure S27. The crystal packing and the unit cell of **2o** crystal structure viewed along the *a*-crystallographic axis (top), *b*-crystallographic axis (middle), and *c*-crystallographic axis (bottom).

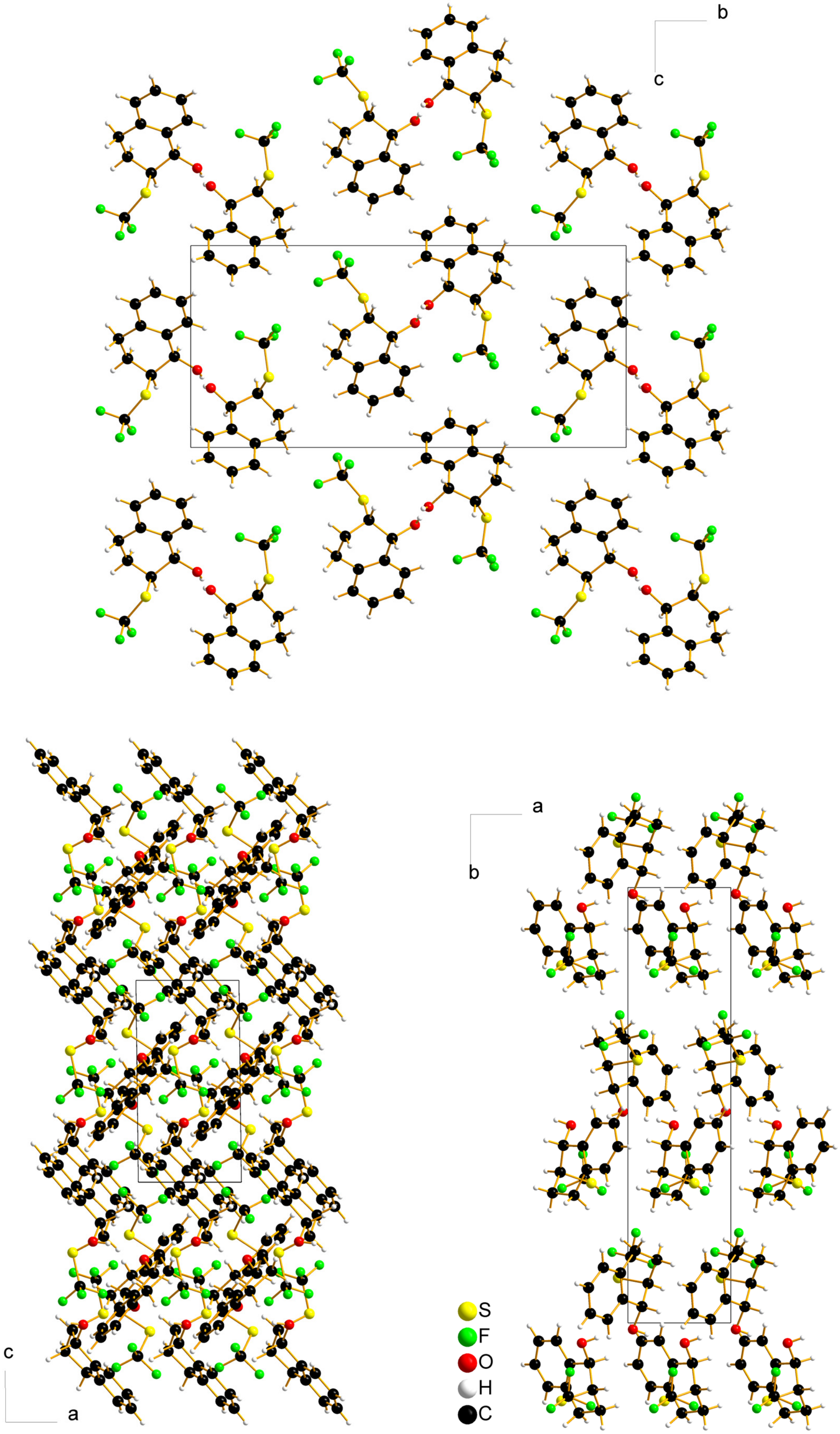

Figure S28. The crystal packing and the unit cell of **2o** ($P2_1$) crystal structure viewed along the *a*-crystallographic axis (top), *b*-crystallographic axis (bottom left), and *c*-crystallographic axis (bottom right).

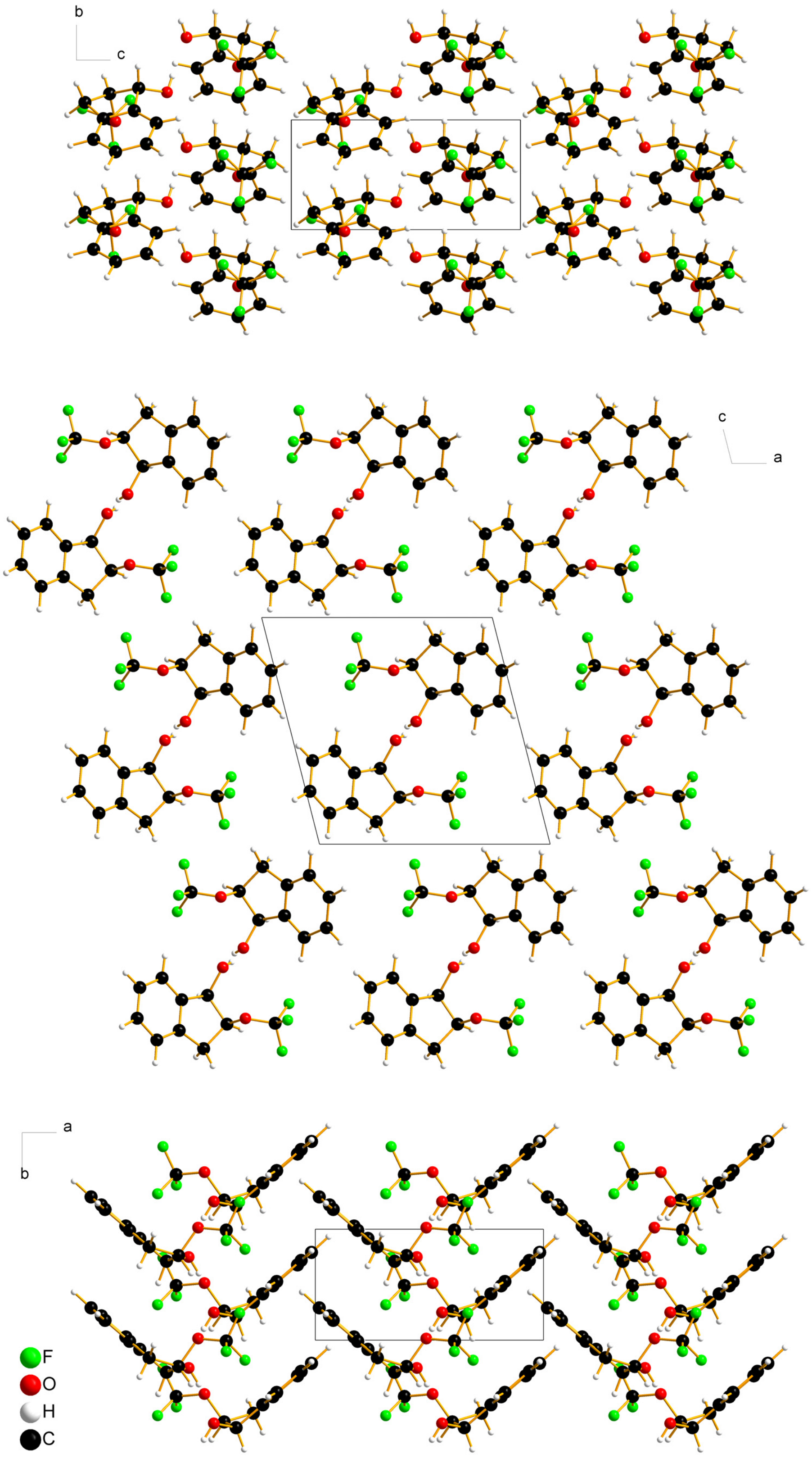

Figure S29. The crystal packing and the unit cell of **2p** crystal structure viewed along the *a*-crystallographic axis (top), *b*-crystallographic axis (middle), and *c*-crystallographic axis (bottom).

Photos of mechanically responsive behavior

Figure S30. Mechanically responsive elastic flexible behavior of **2a**

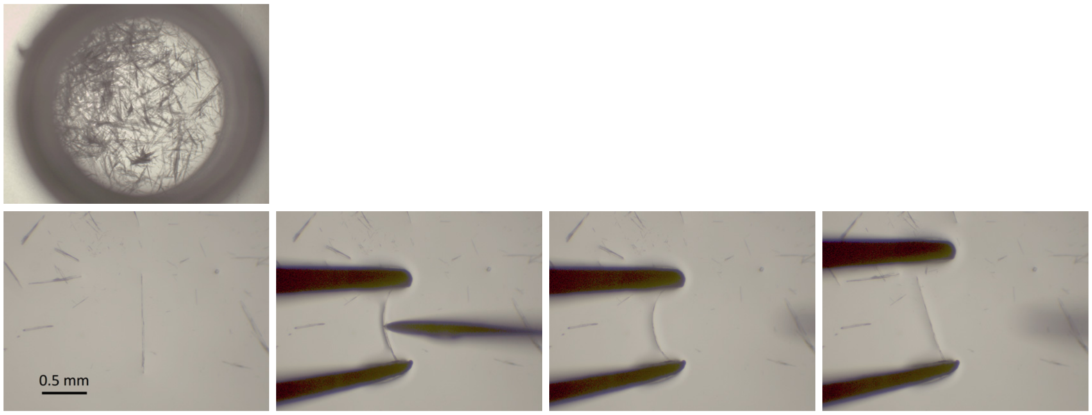

Top: Crystalline sample of **2a** (the inner diameter of vial neck is 10 mm).
Sequence: Three-point bending experiment using metal tweezers and a metal needle (glass slide, crystal immersed in a thin layer of LV CryoOil, MiTeGen).

Figure S31. Mechanically responsive elastic flexible behavior of **2d** (see also "Movie 2d")

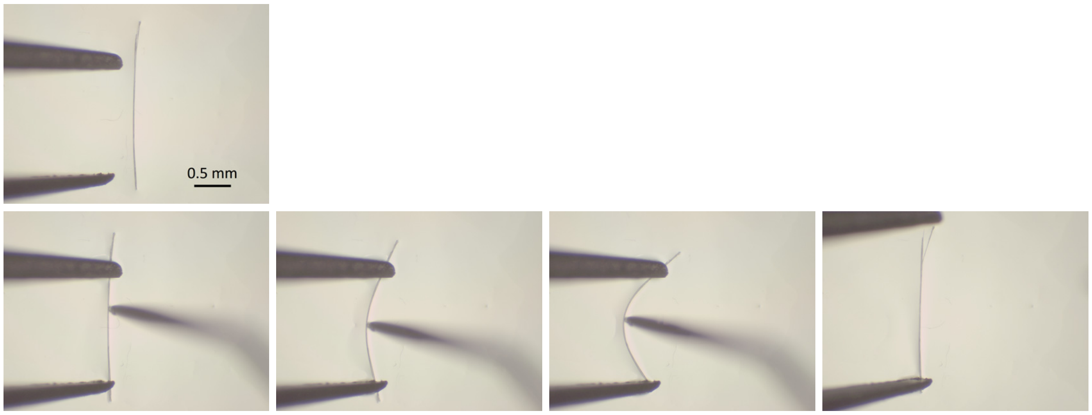

Sequence: Three-point bending experiment using metal tweezers and a metal needle (glass slide, crystal immersed in a thin layer of LV CryoOil, MiTeGen).

Figure S32. Mechanically responsive elastic flexible behavior of (*R*)-**4d**, a sample obtained from DKR-ATH run with (*R*,*R*)-**C2** (se also "Movie 4d")

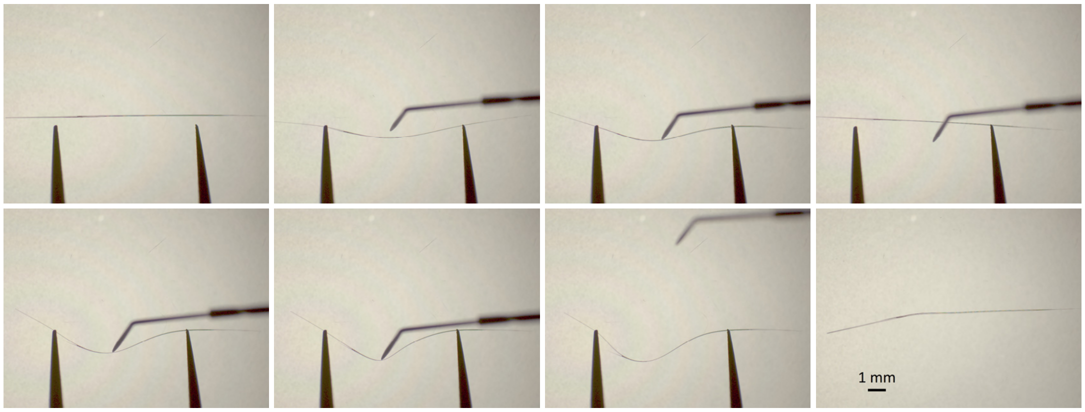

Sequence: Three-point bending experiment using metal tweezers and a metal needle.

Figure S33. Crystalline samples of **2k** (left) and **2n** (right) that exhibit typical brittle behavior (the inner diameter of vial neck is 13 mm)

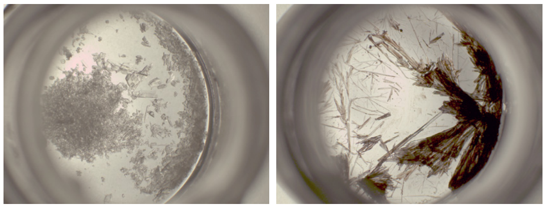

Figure S34. Mechanically responsive plastic flexible behavior of **2o**

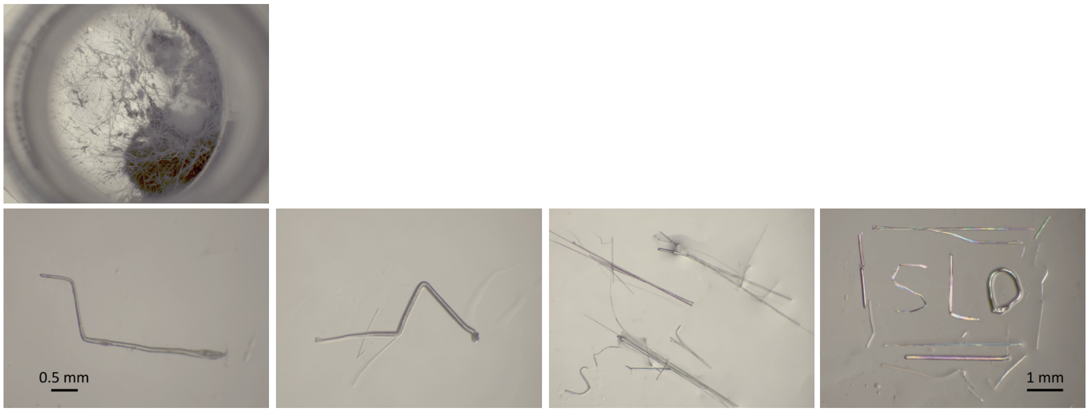

Top left: Crystalline sample of **2o** (the inner diameter of vial neck is 13 mm).
Bottom: Needle-shaped crystals of **2o** can be bent to various shapes without delamination or shattering (glass slide, crystals immersed in a thin layer of LV CryoOil, MiTeGen).

Figure S35. Mechanically responsive elastic flexible behavior of **2p**

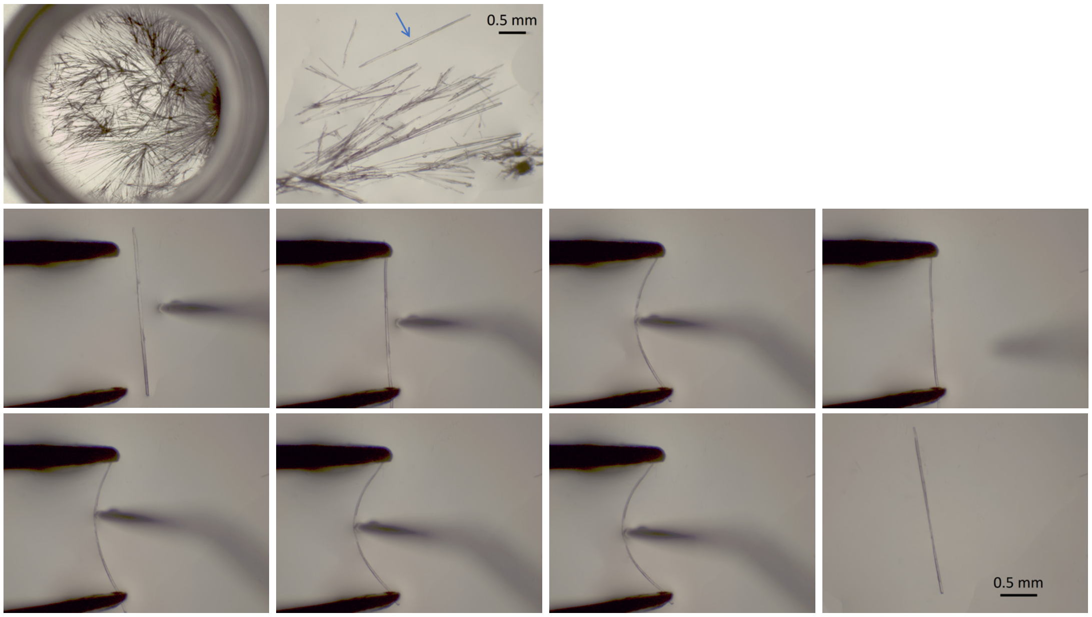

Top left: Crystalline sample of **2p** (the inner diameter of vial neck is 13 mm).
Top right: Needle-shaped crystals on a glass slide, immersed in a thin layer of LV CryoOil (MiTeGen); the crystal used for bending experiment is marked with a blue arrow.
Sequence: Three-point bending experiment using metal tweezers and a metal needle (glass slide, crystal immersed in a thin layer of LV CryoOil, MiTeGen).
